# Global, regional, and national sepsis incidence and mortality, 1990–2017: analysis for the Global Burden of Disease Study

**DOI:** 10.1016/S0140-6736(19)32989-7

**Published:** 2020-01-18

**Authors:** Kristina E Rudd, Sarah Charlotte Johnson, Kareha M Agesa, Katya Anne Shackelford, Derrick Tsoi, Daniel Rhodes Kievlan, Danny V Colombara, Kevin S Ikuta, Niranjan Kissoon, Simon Finfer, Carolin Fleischmann-Struzek, Flavia R Machado, Konrad K Reinhart, Kathryn Rowan, Christopher W Seymour, R Scott Watson, T Eoin West, Fatima Marinho, Simon I Hay, Rafael Lozano, Alan D Lopez, Derek C Angus, Christopher J L Murray, Mohsen Naghavi

**Affiliations:** a Department of Critical Care Medicine, University of Pittsburgh, Pittsburgh, PA, USA; b Division of Pulmonary, Critical Care, and Sleep Medicine, University of Washington, Seattle, WA, USA; c Institute for Health Metrics and Evaluation, University of Washington, Seattle, WA, USA; d Division of Allergy and Infectious Diseases, University of Washington, Seattle, WA, USA; e Department of Pediatrics, University of Washington, Seattle, WA, USA; f Department of Health Metrics Sciences, School of Medicine, University of Washington, Seattle, WA, USA; g Department of Pediatrics, University of British Columbia, Vancouver, BC, Canada; h The George Institute for Global Health, University of New South Wales, Newtown, NSW, Australia; i Center for Sepsis Control and Care, Jena University Hospital, Jena, Germany; j Anesthesiology, Pain and Intensive Care Department, Federal University of São Paulo, São Paulo, Brazil; k Anästhesiologie mit Sp operative Intensivmeidzin, Charité University Medical Center Berlin, Berlin, Germany; l Clinical Trials Unit, Intensive Care National Audit & Research Centre (ICNARC), London, UK; m Faculty of Public Health & Policy linked to the Department of Health Services Research & Policy, London School of Hygiene & Tropical Medicine, London, UK; n Pediatric Critical Care Medicine, Seattle Children's Hospital, Seattle, WA, USA; o Institute of Advanced Studies, University of São Paulo, São Paulo, Brazil; p University of Melbourne, Melbourne, QLD, Australia

## Abstract

**Background:**

Sepsis is life-threatening organ dysfunction due to a dysregulated host response to infection. It is considered a major cause of health loss, but data for the global burden of sepsis are limited. As a syndrome caused by underlying infection, sepsis is not part of standard Global Burden of Diseases, Injuries, and Risk Factors Study (GBD) estimates. Accurate estimates are important to inform and monitor health policy interventions, allocation of resources, and clinical treatment initiatives. We estimated the global, regional, and national incidence of sepsis and mortality from this disorder using data from GBD 2017.

**Methods:**

We used multiple cause-of-death data from 109 million individual death records to calculate mortality related to sepsis among each of the 282 underlying causes of death in GBD 2017. The percentage of sepsis-related deaths by underlying GBD cause in each location worldwide was modelled using mixed-effects linear regression. Sepsis-related mortality for each age group, sex, location, GBD cause, and year (1990–2017) was estimated by applying modelled cause-specific fractions to GBD 2017 cause-of-death estimates. We used data for 8·7 million individual hospital records to calculate in-hospital sepsis-associated case-fatality, stratified by underlying GBD cause. In-hospital sepsis-associated case-fatality was modelled for each location using linear regression, and sepsis incidence was estimated by applying modelled case-fatality to sepsis-related mortality estimates.

**Findings:**

In 2017, an estimated 48·9 million (95% uncertainty interval [UI] 38·9–62·9) incident cases of sepsis were recorded worldwide and 11·0 million (10·1–12·0) sepsis-related deaths were reported, representing 19·7% (18·2–21·4) of all global deaths. Age-standardised sepsis incidence fell by 37·0% (95% UI 11·8–54·5) and mortality decreased by 52·8% (47·7–57·5) from 1990 to 2017. Sepsis incidence and mortality varied substantially across regions, with the highest burden in sub-Saharan Africa, Oceania, south Asia, east Asia, and southeast Asia.

**Interpretation:**

Despite declining age-standardised incidence and mortality, sepsis remains a major cause of health loss worldwide and has an especially high health-related burden in sub-Saharan Africa.

**Funding:**

The Bill & Melinda Gates Foundation, the National Institutes of Health, the University of Pittsburgh, the British Columbia Children's Hospital Foundation, the Wellcome Trust, and the Fleming Fund.

## Introduction

Sepsis is life-threatening organ dysfunction due to a dysregulated host response to infection^1^ and is an important global health problem. In the USA, for example, sepsis is the most common cause of in-hospital deaths and costs more than US$24 billion annually.^2^, ^3^ Infection-prevention efforts, including those targeting both community-acquired and health-care-associated infections, can reduce sepsis incidence.^4^, ^5^ Sepsis is treatable, and timely implementation of targeted interventions improves outcomes.^6^, ^7^, ^8^, ^9^ The World Health Assembly has urged member states to strengthen efforts to identify, document, prevent, and treat sepsis.^10^ Accurate quantification of sepsis incidence and mortality, while important for public health leaders, researchers, and funding agencies, remains a formidable challenge.^11^, ^12^, ^13^

Most previous estimates of sepsis incidence and mortality have relied on hospital administrative databases, excluding patients who were never admitted to hospital,^14^, ^15^, ^16^ and were restricted to national or subnational locations in a selected group of middle-income or high-income countries.^17^, ^18^ A few additional studies have used electronic health record data^19^ or death certificates.^20^ These studies used various methods, thereby hampering comparability over time or by location.^21^ Additionally, many studies were restricted to adults, with a paucity of data for children.^22^, ^23^, ^24^, ^25^, ^26^

The most recent global estimates for sepsis incidence and mortality were based on data for adults admitted to hospital in seven high-income countries^11^ and reported 19·4 million sepsis (formerly, severe sepsis) incident cases and 5·3 million sepsis-related deaths annually. No estimates are available for the global incidence of sepsis and mortality from this disorder according to underlying cause, although these data are vital to understand the clinical context of sepsis. In this study, we used data obtained from the Global Burden of Diseases, Injuries, and Risk Factors Study (GBD) 2017 to estimate the global, regional, and national incidence of sepsis and mortality from this disorder across 195 countries and territories, 282 underlying causes, both sexes, and 23 age groups, for the years 1990 to 2017.

**Research in context**

**Evidence before this study**

Although sepsis is recognised as a major global health problem, few estimates of the global incidence of sepsis or mortality from this disorder exist. Current estimates have been extrapolated from data for adults with sepsis treated in hospital in high-income countries. Most national estimates rely on potentially inaccurate hospital administrative databases and use varying case definitions, leading to disparate estimates even within the same population and hampering comparability over time or by location. Most studies are restricted to patients admitted to hospital and exclude children, ignore the underlying cause of illness, and assess data for only 1 year or a few years.

**Added value of this study**

We assessed the global, regional, and national incidence of sepsis and mortality from this disorder from 1990 to 2017, providing new and robust evidence of the burden of sepsis worldwide. By using vital statistics and hospital admission data for more than 100 million individuals and by incorporating Global Burden of Diseases, Injuries, and Risk Factors Study (GBD) 2017 estimates for causes of death and disease for 195 locations, 282 underlying causes, both sexes, and all ages, we have provided more detailed and evidence-based estimates of the cause and burden of sepsis than have been available previously. Our study complies with the Guidelines for Accurate and Transparent Health Estimates Reporting recommendations.

**Implications of all the available evidence**

The estimated burden of sepsis in 2017 (48·9 million [95% uncertainty interval 38·9–62·9] incident cases and 11·0 million [10·1–12·0] deaths worldwide) is twice that thought previously. This striking increase is largely attributable to the far higher burden among people living in areas with a lower Socio-demographic Index (SDI), for whom data had previously been lacking. Nearly half of all sepsis-related deaths occur secondary to sepsis complicating an underlying injury or non-communicable disease. Our results, using GBD 2017 data, highlight the need for greater prevention and treatment of sepsis, particularly in areas of the world with the lowest SDI.

## Methods

### Study design and data collection

We used GBD 2017 data to produce sepsis estimates that were consistent with other GBD estimates.^27^, ^28^, ^29^, ^30^, ^31^ By contrast with previous hospital-based approaches, vital registration death records were the primary basis for our estimates because they represent deaths in and out of hospital. Use of these records is an essential feature for global sepsis estimates, because much of the sepsis burden could be incurred outside of the hospital, particularly in low-income or middle-income countries. We first estimated sepsis-related mortality using multiple cause-of-death vital registration data and age-specific, sex-specific, location-specific, and cause-specific GBD 2017 estimates for all causes of death worldwide, from 1990 to 2017 (appendix p 2).^31^ We estimated the incidence of sepsis by applying modelled sepsis-related case-fatality from hospital administrative data to mortality estimates (appendix p 3). We followed the Guidelines for Accurate and Transparent Health Estimates Reporting (GATHER) recommendations.^32^

### Defining sepsis

Sepsis is diagnosed clinically by the presence of acute infection and new organ dysfunction.^1^ Unlike the previous idea of septicaemia, which was a non-specific term describing an individual who appeared unwell and had a bloodstream infection, the modern notion of sepsis extends across bacterial, fungal, viral, and parasitic pathogens, focuses on the host response as the major source of morbidity and mortality, and requires only that infection be suspected rather than proven, in recognition that many cases do not have such confirmation.^33^ Since sepsis is presumed to result from underlying infection, it is inherently an intermediate cause of health loss. In some cases, another condition might contribute to the infection (eg, diabetes mellitus). According to the principles of the International Classification of Diseases (ICD), causes of death are assigned based on the underlying disorder that triggers the chain of events leading to death. Therefore, intermediate conditions reported as the cause of death are considered miscoded.^31^ In our analysis, we did not replace GBD methods for handling sepsis-related ICD codes; rather, our methodological approach should be considered a complement to the existing GBD estimation process.

Since sepsis is an intermediate cause of health loss, estimation of its mortality and incidence requires individual-level data with multiple ICD codes specifying the underlying and intermediate causes of death or admission to hospital. For deaths, these data are reported as underlying, intermediate, and immediate causes, according to the International Form of Medical Certificate of Cause of Death recommended by WHO.^34^ Using both underlying and chain (intermediate or immediate) causes of death is a multiple cause-of-death analysis. For admission to hospital, these data are classified as the primary admission diagnosis and secondary admission diagnoses (comorbid disorders or other new conditions identified at admission), as reported by each data source.

Following the approach used in previous studies of sepsis epidemiology,^14^, ^16^, ^35^ we classified sepsis cases within two mutually exclusive groups: explicit and implicit. Explicit sepsis cases were those with an ICD 9th (ICD-9) or 10th (ICD-10) revision code referencing sepsis explicitly (eg, ICD-10 code A41.0 [sepsis due to *Staphylococcus aureus*]; appendix p 6). Implicit sepsis cases were those with both an infection code (eg, ICD-10 code K35 [acute appendicitis]) listed as the underlying cause of death or primary admission diagnosis and an organ dysfunction code listed as a chain cause of death or secondary admission diagnosis (eg, ICD-10 code J96 [acute respiratory failure]; appendix p 6). ICD codes, adapted from the modified Angus criteria,^14^, ^35^ were classified by the study team with input from collaborators with expertise in sepsis epidemiology, critical care, infectious diseases, and paediatrics. A case was eligible to be classified as implicit only if it did not meet criteria for explicit sepsis. Total sepsis estimates are based on both explicit and implicit sepsis cases.

Sepsis cases and deaths are reported according to the underlying GBD cause. Causes have been categorised as infections, injuries, or non-communicable diseases for the unique purposes of this analysis. This custom categorisation and the GBD 2017 cause hierarchy are presented in the appendix (pp 7–14).

### Categorisation of locations

We categorised locations worldwide using the Socio-demographic Index (SDI).^31^ SDI is a summary measure that identifies where countries or other geographical areas sit on the spectrum of development. Expressed on a scale of 0–1, SDI is a composite average of the ranking of the income per capita, average educational attainment, and fertility rates of all areas in the GBD study. Locations are then categorised within SDI quintiles, termed low, low-middle, middle, high-middle, and high. SDI is specific by country and year and, thus, the categorisation of a specific country can change over time.

### Estimating mortality due to sepsis

#### Death certificate data extraction

We included all nationally representative sources of multiple cause-of-death data available in the GBD database, including Brazil, Mexico, Taiwan (province of China), and the USA (appendix pp 4, 15–19). Individual death certificates contained three-digit or four-digit ICD codes. Demographic information was extracted, including age, sex, year, and location of death. Data from Brazil, Mexico, and the USA were extracted at the state level. In total, our analysis included 109 million individual death records, with 9·33 million sepsis-related deaths.

#### Data processing, mapping, and redistribution

ICD codes on a death certificate were classified as explicit sepsis, infectious disease, or organ dysfunction, denoting individual records with an explicit, implicit, or no-sepsis status. ICD codes listed as the underlying cause of death were mapped to one of the 282 diseases reported in the GBD 2017 cause list.^31^ Occasionally, data sources included deaths by a cause for which there is medical consensus that death is impossible for the sex and age (eg, deaths due to cervical cancer in males; appendix pp 20–26). When deaths violated these restrictions, they were redistributed proportionally among all causes. Those deaths with non-specific ICD codes (eg, unspecified stroke) or ICD codes that could not be underlying causes of death (eg, senility or explicit sepsis) were redistributed by age, sex, location, year, and sepsis status to the most likely cause of death (appendix p 4). Methods for redistribution have been described previously.^31^ Records were aggregated by underlying cause, age group, sex, year, location, and sepsis status to generate cause-specific sepsis deaths. Sepsis fractions were calculated for each underlying cause by dividing sepsis deaths by the total number of cause-specific deaths within each stratum.

#### Model and covariate selection

We used mixed-effects linear regression to estimate the fraction of sepsis-related deaths by underlying GBD cause. Covariates included age group, sex, and Healthcare Access and Quality Index (HAQ Index). The HAQ Index uses 32 diseases that would not be fatal with effective health infrastructure to generate a 0–100 score for each location, from 1990 to 2017.^36^ HAQ Index scores in input data ranged from 46·9 to 92·8. Because the dependent variable is a proportion, we modelled the logit of the sepsis fraction.

$$logit(sepsisfraction)=\beta_{HAQIndex}\times X_{HAQIndex}+\beta_{\mathrm{se}x}\times X_{sex}+\pi_{age}+\pi_{level1,level2}+ɛ$$

The model used a nested random-effects structure on the underlying cause of death, allowing prediction of sepsis fractions for diseases with limited input data by borrowing information from diseases within the same group. All underlying causes of death were categorised into 17 groups according to physiological relatedness (appendix pp 27–33).

Model covariates, primarily HAQ Index, were used to extrapolate to standard GBD locations from 1990 to 2017, even when we did not have multiple cause of death data. Predictions and uncertainty intervals (UIs) were generated for the fraction of sepsis-related deaths by drawing 1000 times from the normal distribution of the fixed and random coefficients (separately) for each GBD location, age group, sex, and cause from 1990 to 2017. Point estimates were derived from the mean of the draws, and 95% UIs were derived from the 2·5th and 97·5th percentiles. Uncertainty is attributable to sample size variability between data sources, data availability, and model specifications. The use of UIs instead of CIs allows propagation of uncertainty to the final estimates.^31^ To capture differences in approach for identifying sepsis cases using ICD codes, we did an additional analysis of only explicit sepsis-related deaths.

#### Applying sepsis fractions to GBD causes of death estimates

We multiplied predicted cause-specific, age group-specific, sex-specific, year-specific, and location-specific sepsis fractions by GBD 2017 death estimates to calculate the number of sepsis-related deaths. GBD 2017 provided a comprehensive estimation of cause-specific mortality for 282 causes in 195 countries and territories from 1980 to 2017.^31^ The causes-of-death database included vital registration, verbal autopsy, registry, survey, police, and surveillance data. Statistical modelling tools developed for GBD, including the Cause of Death Ensemble model (CODEm), were used to estimate mortality for each location, year, age group, and sex. We then aggregated the results to arrive at national, regional, and global sepsis-related mortality. The GBD 2017 location hierarchy is included in the appendix (pp 34–50).

### Estimating sepsis incidence

Global sepsis incidence was assessed by dividing estimated sepsis deaths by modelled in-hospital sepsis case-fatality, which was established using individual-level hospital admission or discharge data.

#### Hospital data extraction

Input data included all nationally representative sources of individual-level hospital admission or discharge data with multiple diagnoses available within the GBD database, including data from Austria, Brazil, Canada, Chile, Georgia, Italy, Mexico, New Zealand, Philippines, and the USA (appendix p 51). ICD codes ranged from three to six digits. We extracted age group, sex, year, and location for each record. Data at the subnational level were available for Brazil, Mexico, New Zealand, and the USA. In total, our analysis included 309 million individual hospital records, of which 8·7 million were sepsis-related and served as the basis for case-fatality estimates.

#### Data processing, mapping, and redistribution

We mapped each primary admission diagnosis to a GBD cause, and tagged admissions as either explicit or implicit sepsis. Garbage-coded hospital admissions were redistributed by age, sex, location, year, sepsis type, and fatality. We aggregated records by GBD cause, age group, sex, year, location, and sepsis status to generate the number of cause-specific sepsis cases and deaths. Sepsis case-fatality was defined as the number of sepsis deaths divided by the number of sepsis cases within each stratum.

#### Model and covariate selection

Case-fatality was modelled using a mixed-effects linear regression model. Similar to the model for sepsis mortality, this model included sex, age group, and HAQ Index as covariates and used the nested random-effects structure. HAQ Index values ranged from 48·6 to 94·8. To capture differences in approach for identifying sepsis cases using ICD codes, we did an additional analysis wherein we modelled explicit sepsis case-fatality only. Predictions and UIs were generated for sepsis case-fatality, using the same methods as those used to estimate sepsis mortality, for each standard GBD location, age group, sex, and cause from 1990 to 2017 (appendix p 79).

$$logit(casefatality)=\beta_{HAQIndex}\times X_{HAQIndex}+\beta_{sex}\times X_{sex}+\pi_{age}+\pi_{level1,level2}+ɛ$$

#### Calculating incidence

We calculated sepsis incidence by dividing sepsis deaths by in-hospital case-fatality for each cause, age group, year, sex, and location after enforcing age-sex cause restrictions.

### Role of the funding source

The funders had no role in study design, data collection, data analysis, data interpretation, or writing of the report. The corresponding author had full access to all data in the study and had final responsibility for the decision to submit for publication.

## Results

### Sepsis incidence

**Figure 1:**
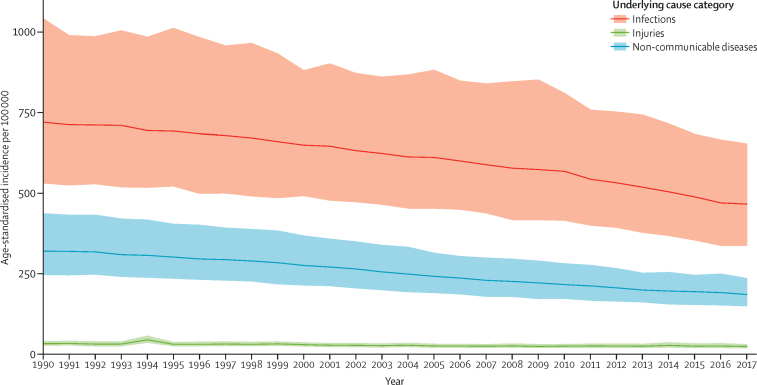
Age-standardised global sepsis incidence per 100 000 population, for both sexes and by underlying cause category, 1990–2017 Shaded areas represent 95% uncertainty intervals.

Globally, there were an estimated 60·2 million (95% UI 47·2–79·7) cases of sepsis in 1990 and 48·9 million (38·9–62·9) cases of sepsis in 2017 (table 1). This change represents a decrease of 18·8% (95% UI 5·9–42·2). Of all incident cases of sepsis in 2017, 33·1 million (95% UI 24·1–45·9 [67·4%, 95% UI 59·1–75·7]) occurred in people with an underlying infectious cause of health loss, and 15·8 million (12·7–20·0 [32·6%, 24·3–40·9]) occurred in individuals with underlying injuries or non-communicable diseases (table 1). The global age-standardised incidence of sepsis fell from 1074·7 (95% UI 861·4–1397·5) cases per 100 000 in 1990 to 677·5 (535·7–876·1) cases per 100 000 in 2017, a decrease of 37·0% (95% UI 11·8–54·5; figure 1). This declining incidence was seen in nearly every location worldwide (appendix pp 52–62).

**Table 1:** Incident cases of sepsis and age-standardised incidence of sepsis, for all ages, both sexes, and all locations, according to category of underlying cause, 2017

	**Male**		**Female**		**Both sexes**	
	Incident cases (95% UI)	Age-standardised incidence per 100 000 population (95% UI)	Incident cases (95% UI)	Age-standardised incidence per 100 000 population (95% UI)	Incident cases (95% UI)	Age-standardised incidence per 100 000 population (95% UI)
Infections	15 961 632 (11 416 679–22 490 150)	453·5 (323·5–641·6)	17 165 460 (12 324 759–24 539 248)	482·4 (344·1–695·4)	33 127 159 (24 112 267–45 885 664)	466·8 (337·4–654·8)
Injuries	1 202 056 (916 529–1 548 161)	31·7 (24·2–40·8)	663 329 (494 773–850 850)	17·8 (13·2–23·1)	1 865 358 (1 421 131–2 392 774)	24·7 (18·8–31·7)
Non-communicable diseases	5 567 578 (4 499 826–7 157 847)	157·6 (126·8–203·8)	8 349 730 (6 520 440–11 096 832)	216·4 (167·6–290·8)	13 917 451 (11 313 974–17 629 415)	186·0 (150·0–237·0)
All causes	22 731 266 (18 037 098–29 410 723)	642·8 (507·7–834·8)	26 178 518 (20 630 286–33 702 305)	716·5 (560·2–925·1)	48 909 968 (38 929 606–62 859 320)	677·5 (535·7–876·1)

Data are n (95% UI), unless otherwise stated. UI=uncertainty interval.

**Figure 2:**
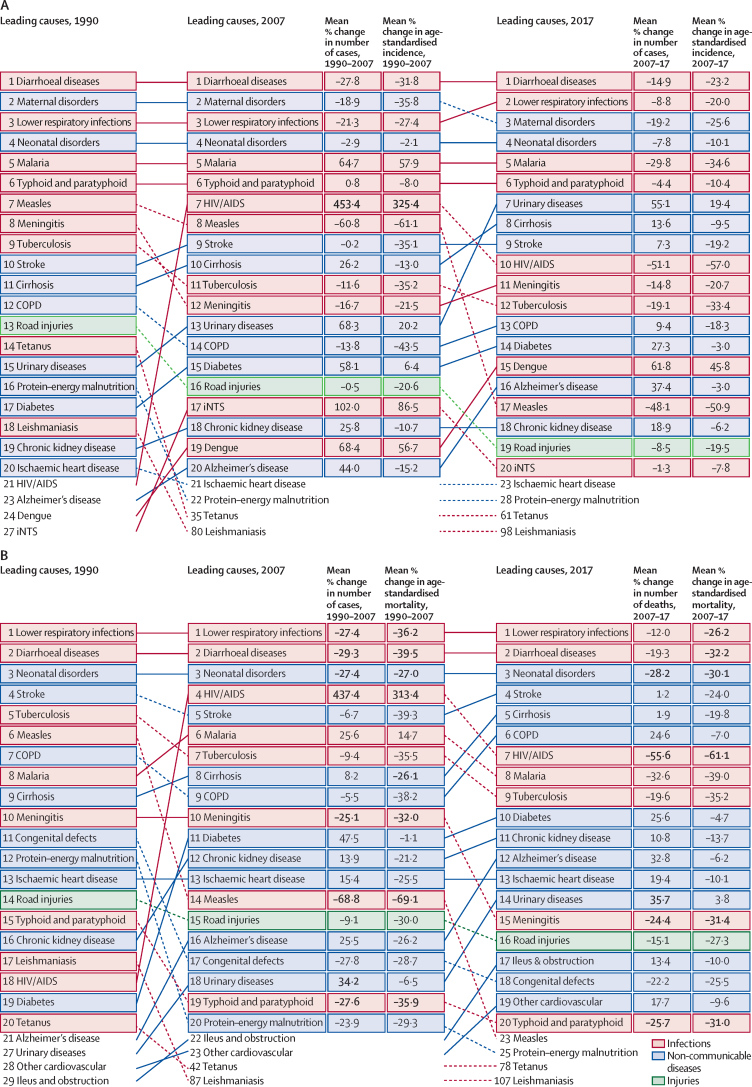
Leading 20 Level 3 causes of global incident sepsis (A) and sepsis-related deaths (B) for both sexes and all ages combined, in 1990, 2007, and 2017 Causes are connected by lines between periods (1990, 2007, and 2017); solid lines are ranked increases (or no change in rank) and dashed lines are ranked decreases. Numbers in bold highlight statistically significant changes between periods. COPD=chronic obstructive pulmonary disease. iNTS=invasive non-typhoidal salmonella.

Among all age groups, both sexes, and all locations, the most common underlying cause of sepsis was diarrhoeal disease, in every year from 1990 to 2017, with 15·0 million (95% UI 6·34–32·0) cases of sepsis attributable to diarrhoeal diseases in 1990 and 9·21 million (3·56–20·9) in 2017 (figure 2A; appendix pp 63–68). In 2017, the most common underlying injury to cause sepsis was road traffic injury (457 495 [95% UI 282 177–715 774] cases of sepsis), and maternal disorders were the most common non-communicable disease complicated by sepsis (5·7 million [3·4–9·2] cases of sepsis; appendix pp 63–68). Among children younger than 5 years, the most common causes of sepsis in 2017 were diarrhoeal diseases (5·9 million [95% UI 2·1–14·2] cases of sepsis [27·9%, 95% UI 12·0–50·8]), neonatal disorders (5·1 million [2·9–8·9] cases of sepsis [25·7%, 13·7–40·9]), and lower respiratory infections (3·3 million [1·8–6·3] cases of sepsis [16·5%, 0·1–29·3]; data not shown).

Global age-standardised sepsis incidence in 2017 was higher among females than males (716·5 [95% UI 560·2–925·1] cases per 100 000 *vs* 642·8 [507·7–834·8] cases per 100 000; table 1). Overall, sepsis incidence peaked in early childhood, with a second peak in incidence among older adults (appendix p 80). In 2017, there were an estimated 20·3 million (95% UI 14·0–29·7) incident sepsis cases worldwide among children younger than 5 years, 4·9 million (3·5–7·0) incident sepsis cases among children and adolescents aged 5–19 years, and 23·7 million (20·1–28·8) incident sepsis cases among adults 20 years and older (data not shown).

**Figure 3:**
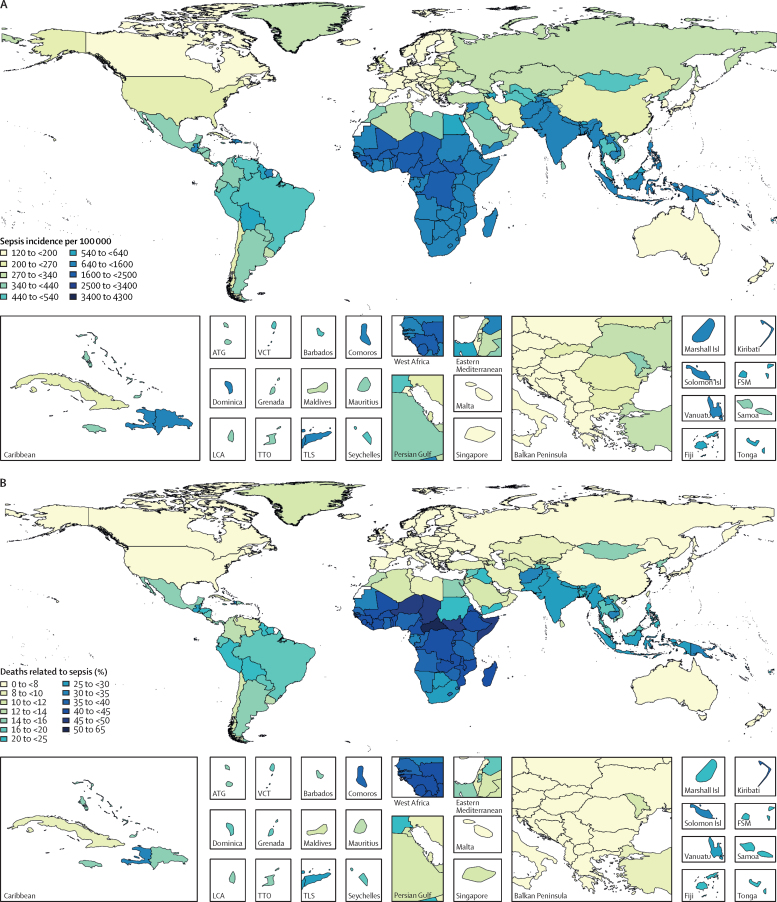
Age-standardised sepsis incidence per 100 000 population for both sexes, in 2017 (A), and percentage of all deaths related to sepsis, age-standardised for both sexes, in 2017 (B) ATG=Antigua and Barbuda. FSM=Federated States of Micronesia. LCA=Saint Lucia. TLS=Timor-Leste. TTO=Trinidad and Tobago. VCT=Saint Vincent and the Grenadines.

**Figure 4:**
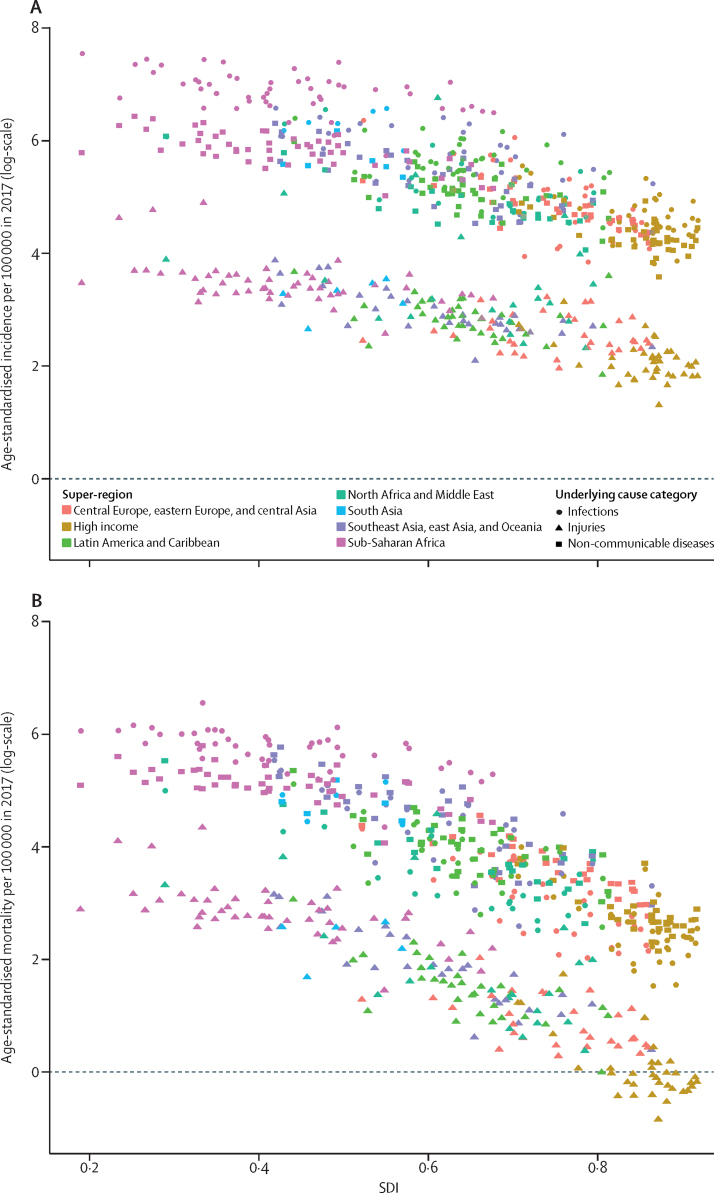
Age-standardised sepsis incidence (A) and mortality (B) per 100 000 population and SDI, by location and underlying cause category for both sexes, in 2017 Every point represents one country or territory. 195 countries and territories worldwide are categorised according to SDI. SDI=Socio-demographic Index.

Patterns of sepsis incidence varied substantially according to location (figure 3A; appendix pp 52–62, 81, 82). The highest age-standardised incidence of sepsis occurred in areas with the lowest SDI (figure 4A). Among all ages, both sexes, and all underlying causes, an estimated 52·2 million (95% UI 40·5–70·9) incident cases of sepsis in 1990 (87·0% [95% UI 84·9–89·2] of total) and 41·5 million (32·1–54·5) incident sepsis cases in 2017 (85·0% [82·2–87·4] of total) occurred in countries with a low, low-middle, or middle SDI.

In the sensitivity analysis in which only explicit sepsis ICD codes were modelled, there were an estimated 37·0 million (95% UI 30·9–44·6) incident cases of explicit sepsis worldwide in 2017, with an age-standardised explicit sepsis incidence of 508·4 (95% UI 421·8–612·3) cases per 100 000.

### Sepsis-related mortality

There were an estimated 11·0 million (95% UI 10·1–12·0) total sepsis-related deaths worldwide in 2017, representing 19·7% (18·2–21·4) of deaths that year (figure 3B; table 2). Global age-standardised mortality for sepsis in 2017 was 148·1 (95% UI 136·4–161·0) deaths per 100 000 population. Sepsis-related deaths were identified across the full spectrum of underlying causes of death, including non-communicable diseases, injuries, and infections. Of all sepsis deaths in 2017, 5·11 million (95% UI 4·54–5·78) deaths, representing 46·4% (95% UI 42·2–50·8) of the total, occurred in individuals with a non-infectious underlying cause of death.

**Table 2:** Sepsis-related deaths, percentage of total deaths related to sepsis, and age-standardised mortality related to sepsis, for all ages, both sexes, and all locations, according to category of underlying cause of death, 2017

	**Male**			**Female**			**Both sexes**		
	Sepsis-related deaths (95% UI)	Percentage of total global deaths (95% UI)	Age-standardised mortality per 100 000 population (95% UI)	Sepsis-related deaths (95% UI)	Percentage of total global deaths (95% UI)	Age-standardised mortality per 100 000 population (95% UI)	Sepsis-related deaths (95% UI)	Percentage of total global deaths (95% UI)	Age-standardised mortality per 100 000 population (95% UI)
Infections	3 161 020 (2 787 158–3 623 313)	52·2% (46·6–57·6)	89·4 (78·5–102·4)	2 894 883 (2 574 910–3 285 846)	57·1% (51·7–62·5)	76·6 (68·5–86·2)	6 055 890 (5 414 160–6 776 079)	54·4% (48·9–59·7)	82·5 (73·8–92·1)
Injuries	339 309 (282 155–404 432)	5·5% (4·6–6·5)	9·0 (7·4–10·7)	186 537 (152 681–227 604)	7·3% (6·0–8·9)	4·7 (3·9–5·8)	525 838 (437 501–626 696)	6·1% (5·1–7·2)	6·8 (5·7–8·1)
Non-communicable diseases	2 395 358 (2 083 336–2 768 606)	11·1% (9·7–12·8)	67·8 (58·8–78·6)	2 186 969 (1 917 124–2 506 269)	11·3% (9·9–13·0)	54·5 (47·9–62·3)	4 582 316 (4 021 478–5 245 002)	11·2% (9·8–12·9)	60·6 (53·4–69·6)
All causes	5 826 339 (5 334 433–6 372 795)	19·2% (17·6–21·0)	164·2 (150·1–180·1)	5 194 467 (4 777 508–5 691 003)	20·3% (18·7–22·2)	134·1 (123·6–146·1)	11 020 776 (10 145 212–11 994 113)	19·7% (18·2–21·4)	148·1 (136·4–161·0)

Data are n (95% UI), unless otherwise stated. Denominators for calculating percentage of global deaths were taken from reference 28. UI=uncertainty interval.

Globally, for both sexes and all age groups combined, the most common underlying cause of sepsis-related death was lower respiratory infection in every year from 1990 to 2017, with 2·8 million (95% UI 2·3–3·2) sepsis-related deaths in 1990 and 1·8 million (1·3–2·1) sepsis-related deaths in 2017 attributable to lower respiratory infections (figure 2B; appendix pp 63–68). Of the most common underlying causes of sepsis-related deaths in 2017, road injuries were the most common injury-related cause with 145 520 (95% UI 100 480–200 090) sepsis-related deaths, and neonatal disorders were the most common non-communicable disease with 801 615 (627 191–996 840) sepsis-related deaths (appendix pp 63–68). Globally, among children younger than 5 years, the three most common causes of sepsis-related deaths in 2017 were neonatal disorders (801 615 [95% UI 627 191–996 840] deaths), lower respiratory infections (641 682 [508 331–748 106] deaths), and diarrhoeal diseases (447 783 [340 224–532 225] deaths; data not shown).

**Figure 5:**
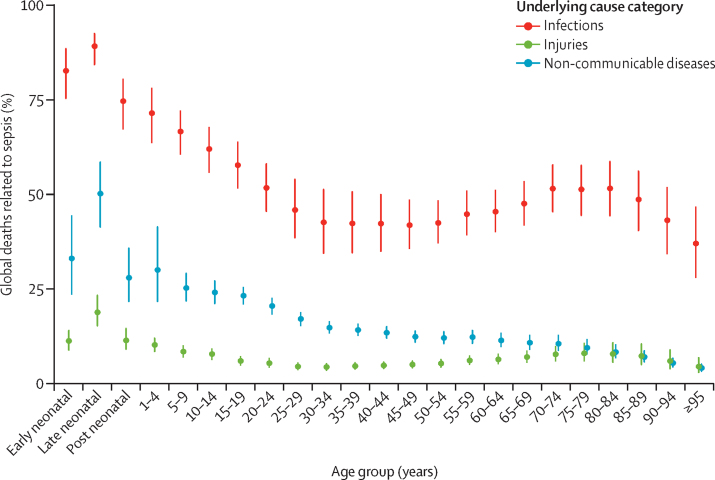
Percentage of all sepsis-related deaths in each underlying cause category, by age group and for both sexes, in 2017 Bars represent 95% uncertainty intervals.

Global age-standardised sepsis-related mortality in 2017 was higher among males than females (164·2 [95% UI 150·1–180·1] per 100 000 *vs* 134·1 [123·6–146·1] per 100 000; table 2). The percentage of all global deaths (from any cause) which were related to sepsis in 2017 peaked in early childhood, declined through early adulthood, and rose among older adults (figure 5). In 2017, there were an estimated 2·9 million (95% UI 2·6–3·2) deaths related to sepsis worldwide among children younger than 5 years, 454 000 (418 000–493 000) among children and adolescents aged 5–19 years, and 7·7 million (6·9–8·5) among adults 20 years and older (data not shown).

There were an estimated 15·7 million (95% UI 14·7–16·7) deaths related to sepsis in 1990, versus 11·0 million (10·1–12·0) deaths in 2017, a decrease of 29·7% (95% UI 22·1–36·6). The age-standardised percentage of all global deaths related to sepsis declined from 29·1% (95% UI 27·2–31·4) in 1990 to 20·1% (18·5–21·8) in 2017, a decrease of 31·0% (23·7–37·9; figure 3B; appendix pp 81–84).

Patterns of sepsis-related mortality varied substantially according to location (figure 3B; appendix pp 69–78, 81, 82, 85). The highest age-standardised sepsis-related mortality occurred in areas with the lowest SDI (figure 4B). This inverse relation with SDI was stronger for mortality than for incidence (figure 4), reflecting further disparities in sepsis-associated case-fatality according to SDI. In low SDI locations, most sepsis-related deaths were due to infection, whereas most sepsis-related deaths in high SDI locations were attributable to non-communicable diseases (appendix p 85). Among all ages, both sexes, and all underlying causes of death, an estimated 13·6 million (95% UI 12·7–14·5) sepsis-related deaths (87·1% [95% UI 86·0–88·0] of total) in 1990 and 8·2 million (7·6–8·9) sepsis-related deaths in 2017 (84·8% [83·6–85·8] of total) occurred in countries with low, low-middle, or middle SDIs.

In the sensitivity analysis in which only explicit sepsis ICD codes were modelled, there were an estimated 9·2 million (95% UI 8·4–10·1) total sepsis-related deaths worldwide in 2017, representing 16·5% (14·9–18·1) of all deaths that year.

## Discussion

Our study is the first to produce global estimates of sepsis incidence and mortality across 195 countries and territories, 282 underlying causes of death, both sexes, and 23 age groups for the years 1990 to 2017. Our findings indicate that there were an estimated 48·9 million (95% UI 38·9–62·9) incident cases of sepsis and 11·0 million (10·1–12·0) sepsis-related deaths in 2017. These estimates are more than double previous global figures, which is probably attributable to inclusion of more data from low-income and middle-income countries, locations where sepsis incidence and mortality are considerably higher and for which data were previously under-represented. Furthermore, the difference between these current estimates and previous global estimates was especially striking among children, such that more than half of all sepsis cases worldwide in 2017 occurred among children, many of them neonates.

These findings have several key implications for health policy makers, clinicians, and researchers. First, the global burden of sepsis is larger than previously appreciated, requiring urgent attention. Second, there is substantial variation in sepsis incidence and mortality according to HAQ Index, with the highest burden in locations that are least equipped to prevent, identify, or treat sepsis. Further research to understand these disparities, and development of policies and practices targeting their amelioration, is crucial.^37^ Third, more robust infection-prevention measures should be assessed and implemented in areas with the highest incidence of sepsis and among populations on which sepsis will have the greatest impact, such as neonates. In addition to continued public health work targeting common infections such as diarrhoeal diseases, there could be important opportunities for sepsis prevention in locations with a high incidence of sepsis attributable to non-communicable diseases or injuries. Although robust data are scarce, many of these cases of sepsis are suspected to be due to nosocomial infections; patients admitted to hospital for non-infectious conditions could be exposed to infection risk either from invasive devices such as central venous or urinary catheters or through inadequate handwashing practices among health-care workers.^38^ Research and policy interventions targeting antimicrobial resistance, an important driver of sepsis (particularly in health-care settings), are imperative. Fourth, clinicians and public health policy makers must implement cost-effective measures proven to improve sepsis outcomes in the locations and patient groups with disparately high sepsis-related mortality.^7^, ^39^ Patients with sepsis frequently present for urgent medical care with undifferentiated infection. All sepsis patients, regardless of underlying source, have a shared need for access to basic acute care services such as timely and appropriate antibiotic administration, microbiology facilities, and capacity for organ support.

Our study is the first to use multinational individual-level data to produce global sepsis estimates. The most recent estimate of the global burden of sepsis, by Fleischmann and colleagues,^11^ suggested that there were 19·4 million cases of hospital-treated sepsis a year (previously termed severe sepsis) and 5·3 million sepsis-related deaths annually. As a systematic review, that study was limited by necessary extrapolation from population-level data inputs, preventing age-standardisation of estimates.^11^ Additionally, the study was restricted to data from high-income countries.^11^ The only previous estimate of global paediatric and neonatal sepsis incidence (4·2 million cases among children younger than 20 years old in 2018)^13^ is substantially lower than our estimate of 25·2 million (95% UI 17·9–35·9) cases in 2017, a difference that could be partly explained by our study's inclusion of data from low-income countries and adjustment for health-care access and quality.

Although our study's global estimates are substantially higher than those previously published, our finding that about 20% of all global deaths in 2017 were related to sepsis is lower than previous estimates of the proportion of deaths among patients admitted to hospital that were related to sepsis (approximately 30–50%).^2^, ^19^ Additionally, our estimates for many locations with a high SDI are lower than previously published.^17^, ^19^ Rhee and colleagues^19^ used electronic health record data to estimate that there were 1·7 million incident adult sepsis cases requiring admission to hospital in the USA in 2014, with 270 000 sepsis-related deaths. This is higher than our corresponding estimate of 903 000 incident cases and 174 000 sepsis-related deaths among adults aged 20 years and older. One potential reason for this discrepancy is that we used death certificates as the base data source rather than electronic health record data, and death certificates might be less likely to contain explicit sepsis or organ dysfunction ICD codes, even when those conditions were present. Further efforts are needed to establish robust population-level sepsis surveillance, because current ICD-based strategies might overestimate or underestimate the true burden in some locations.

The 282 causes of death in the GBD 2017 study, classified according to underlying cause of health loss, are collectively exhaustive and mutually exclusive. Our study identified incident cases of sepsis and deaths from this disorder among all GBD causes, irrespective of whether the underlying cause was an infection, non-communicable disease, or injury. These incident cases and deaths can be considered to be dually labelled, first, with one of the 282 underlying causes and, second, with sepsis as an intermediate cause. Therefore, it is impossible to directly compare the burden of sepsis to any of the underlying causes (appendix p 5).

Our study has several strengths. First, we used extensive, multinational, individual-level death certificate and hospitalisation data, allowing for a uniquely granular assessment of the burden of sepsis according to specific strata of age group, sex, location, year, and underlying cause of illness or death. Inclusion of data from areas that differ substantially in infection profile, comorbidity pattern, medical coding practices, and HAQ Index enhanced the ability of our study to make national-level and regional-level estimates, even for locations without data. Second, use of vital registration data allowed for mortality estimates that were not restricted to patients admitted to hospital. Third, use of neonatal, paediatric, and adult data permitted estimation of subpopulation age-based estimates and age-standardised population-level estimates. Fourth, assessment of the burden of sepsis within the framework of all 282 underlying causes of death in the GBD 2017 study, rather than solely communicable diseases, allowed us to identify a large number of incident cases of sepsis and deaths with non-infectious underlying causes leading to acute infection then to sepsis. These cases might have been excluded from previous estimates based on extrapolation of sepsis incidence from that of infectious diseases, and, importantly, stratification of sepsis cases according to underlying cause might inform targeted sepsis prevention efforts within specific patient populations.

Our study has several limitations. First, input data were restricted to sources available at the time of analysis, either individual-level vital registration or hospitalisation data with multiple ICD codes. Although 109 million death certificates and 309 million individual hospital records were available for analysis, these were limited by range of HAQ Index and underlying cause, restricting model accuracy for locations or subpopulations without input data. It is unclear in which direction this limitation would be most likely to bias estimates. Improved availability of high-quality data sources with multiple causes of death or hospitalisation, particularly in low-income and middle-income countries, is vital to improve future estimates. Second, although most studies of sepsis epidemiology have used an ICD code-based approach, this strategy has imperfect correlation with clinician chart review for the identification of patients with sepsis.^19^, ^35^ Third, the ICD code approach for implicit sepsis was novel in that we required infection codes to be listed as the underlying cause of death, and organ dysfunction codes to be listed in the chain of death. This method was used because of the ordered nature of death certificates, to best ensure that the underlying infection caused the organ dysfunction and, thus, represented sepsis. This approach restricted the identification of implicit sepsis deaths to those with infection as the underlying cause and, therefore, might have led to an underestimate of the burden. Fourth, although the ICD codes were based on modified Angus criteria,^14^, ^35^ they were substantially further modified to reflect the most current definition of sepsis,^1^ modern understanding of sepsis pathophysiology, and global infection patterns. As with any ICD code-based method, there is a risk of misclassification, potentially leading to overestimation or underestimation. There is some disagreement about which infections, when identified in association with acute organ dysfunction, should be considered as sources of sepsis. Additionally, because of changing sepsis definitions over time, there could be additional misclassification, particularly in older data sources. The specific ICD code approach used in our study should be further validated. Fifth, our study was not designed to distinguish between hospital-acquired and community-acquired sepsis, an important differentiation that could inform future sepsis prevention initiatives. Sixth, although the data used for sepsis mortality estimates are representative of the whole population, case-fatality data used to estimate incidence are drawn from hospitalisation records. There are no reliable data to inform the comparison of in-hospital versus out-of-hospital sepsis-associated case-fatality, although it is possible that these two differ substantially.

Using GBD 2017 cause of death results^31^ and multiple cause-of-death data, we have produced global sepsis estimates that are more than double previous calculations, with 11 million sepsis deaths and 48·9 million incident sepsis cases in 2017. We have shown a global trend of decreasing sepsis burden but, importantly, substantial differences between regions remain, in total number of sepsis deaths, age distribution of sepsis deaths, and case-fatality. These differences by location are alarming and deserve urgent attention from the global health, research, and policy communities.
