# Supplementary material for: Global, regional, and national sepsis incidence and mortality, 1990–2017: analysis for the Global Burden of Disease Study
Source: Lancet. 2020 Jan 18;395(10219):200–11. doi: 10.1016/S0140-6736(19)32989-7 (PMC6970225; doi:10.1016/S0140-6736(19)32989-7)
Supplement: Supplementary appendix [file mmc1.pdf]

THE LANCET

Supplementary appendix

This appendix formed part of the original submission and has been peer reviewed. We post it as supplied by the authors.

Supplement to: Rudd KE, Johnson SC, Agesa KM, et al. Global, regional, and national sepsis incidence and mortality, 1990–2017: analysis for the Global Burden of Disease Study. *Lancet* 2020; published online Jan 16. http://dx.doi.org/10.1016/S0140-6736(19)32989-7.

Global, regional, and national sepsis incidence and mortality, 1990–2017: online supplement

Contents

I. Supplemental methods

eMethods 1. Conceptual diagram of sepsis mortality estimation process **Page 2**
eMethods 2. Conceptual diagram of sepsis incidence estimation process **Page 3**
eMethods 3. Data sources, processing, mapping, and redistribution **Page 4**
eMethods 4. Conceptual diagram of sepsis as intermediate cause of death **Page 5**

II. Supplemental tables and figures

eTable 1. International Classification of Diseases 9th and 10th Revision codes for the identification of sepsis **Page 6**
eTable 2. Custom cause groups for infections, injuries, and non-communicable diseases **Page 7**
eTable 3. Data sources for modelling sepsis mortality **Page 15**
eTable 4. Data quality rating from 0 to 5 stars for multiple-cause vital registration data used in modelling sepsis mortality **Page 16**
eTable 5. Global Burden of Disease 2017 age-sex restrictions **Page 20**
eTable 6. Cause of death nesting hierarchy **Page 27**
eTable 7. Global Burden of Disease 2017 location hierarchy with levels **Page 34**
eTable 8. Data sources for modelling sepsis incidence **Page 51**
eTable 9. Sepsis incidence by location for all ages, both sexes, and all underlying causes, 1990 and 2017 **Page 52**
eTable 10. Global sepsis-related mortality and incidence by underlying cause for all ages and both sexes, 2017 **Page 63**
eTable 11. Sepsis-related mortality by location for all ages, both sexes, and all underlying causes, 1990 and 2017 **Page 69**
eFigure 1. Age-standardised sepsis-related in-hospital case fatality by year and region, both sexes, 1990–2017 **Page 79**
eFigure 2. Incident sepsis cases by age group and underlying cause category, both sexes, all locations, 2017 **Page 80**
eFigure 3. Age-standardised sepsis incidence rate per 100,000 population, both sexes, 1990 (panel A) and percentage of all deaths related to sepsis, age-standardised, both sexes, 1990 (panel B) **Page 81**
eFigure 4. Ranking of sepsis-related mortality for each year 1990–2017, by Global Burden of Disease region **Page 83**
eFigure 5. Percentage of global deaths related to sepsis, age-standardised, both sexes, by underlying cause category, 1990–2017 **Page 84**
eFigure 6. Percentage of sepsis deaths by underlying cause category and quintile of Socio-demographic Index, age-standardised, both sexes, 2017 **Page 85**

eMethods 1. Conceptual diagram of sepsis mortality estimation process

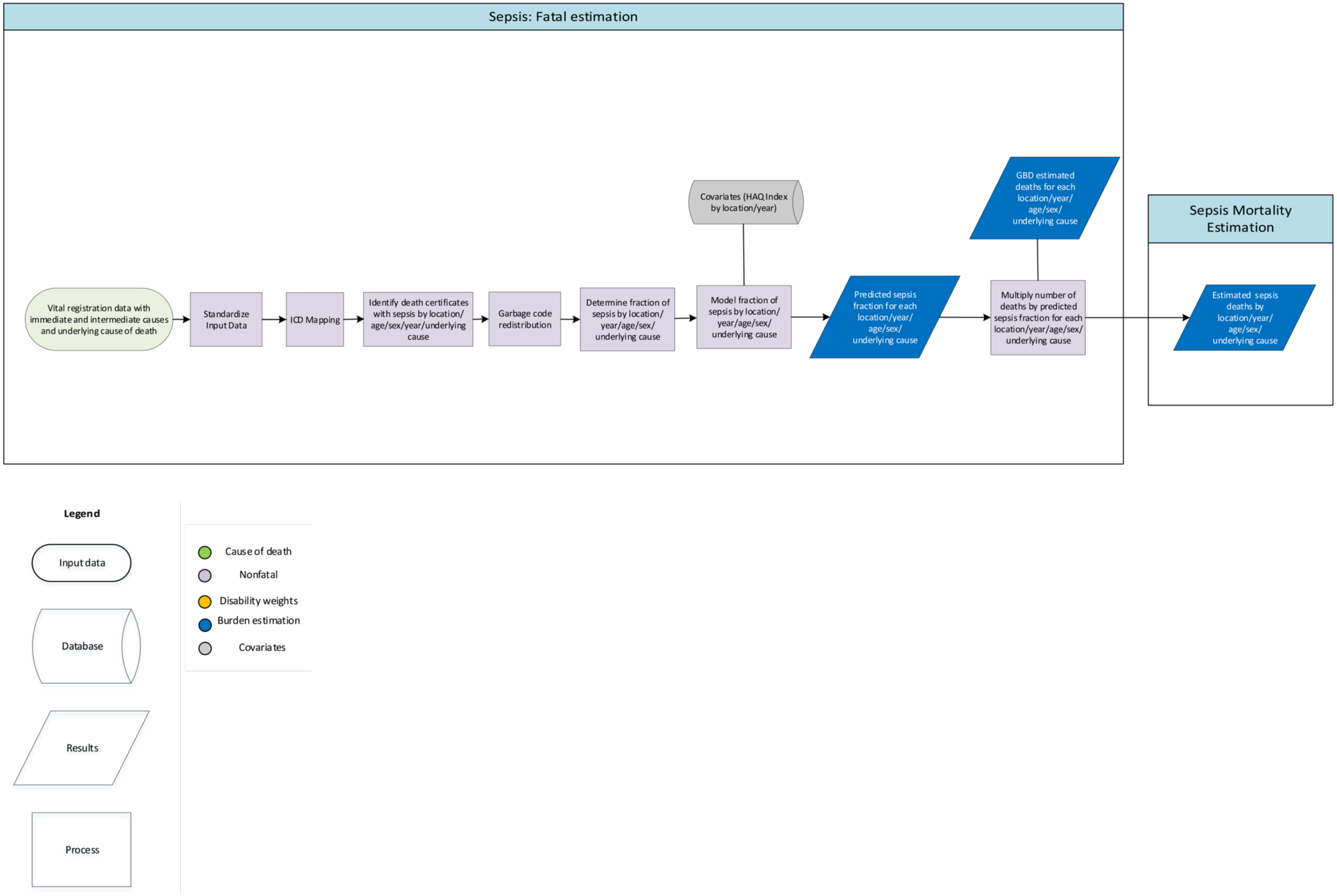

eMethods 2. Conceptual diagram of sepsis incidence estimation process

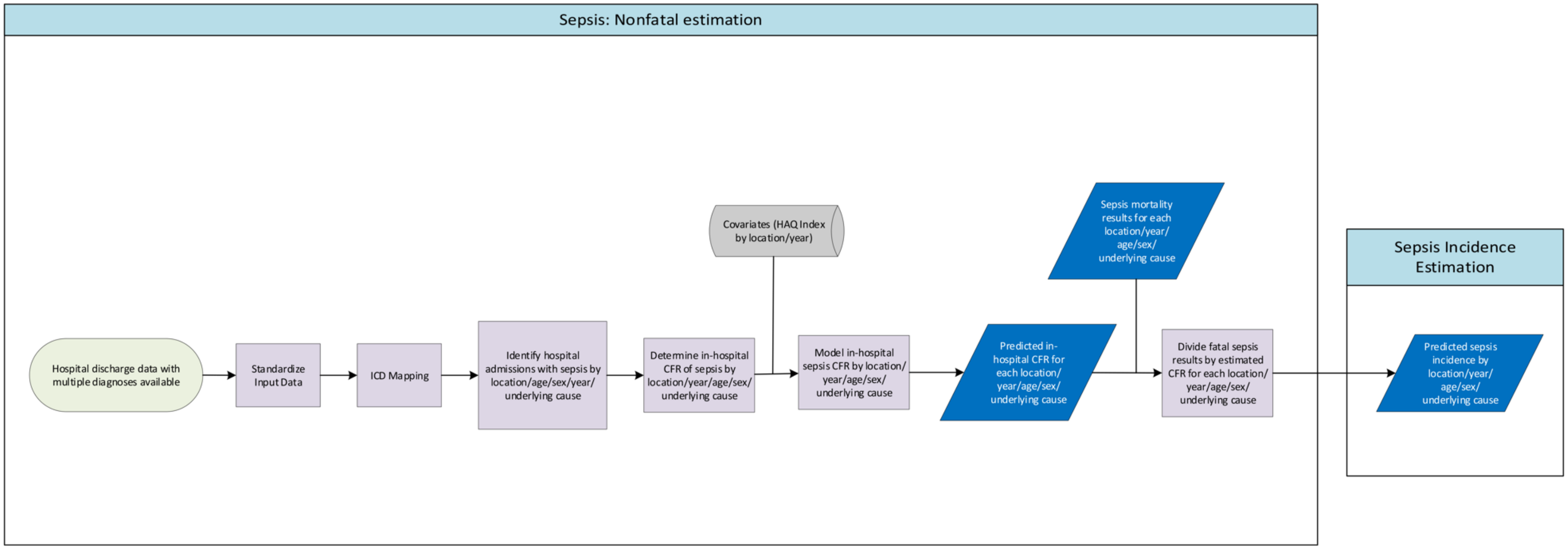

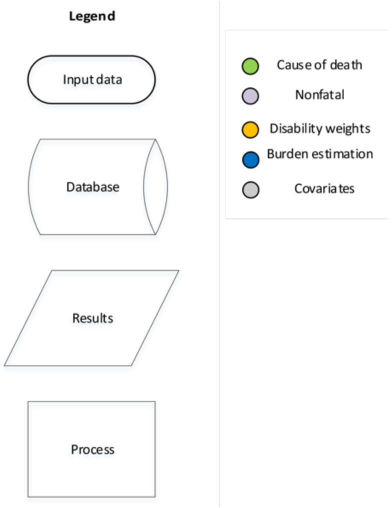

eMethods 3. Data quality

As of GBD 2016, a star rating from 0 to 5 is used to quantify the quality of data available in a given location for the time interval estimated by GBD, with 5 stars representing the highest-quality data. Locations improve in the star rating as they increase availability, completeness, and detail of their mortality data and reduce the percentage of deaths coded to ill-defined garbage codes or highly aggregated causes. The exact methods for calculating stars can be found in Roth et al *Lancet* 2018.[31] The star rating for the vital registration systems where multiple cause of death data were available for sepsis-specific analyses is shown in eTable 4. Complete star ratings by country for the 1980–2017 time interval can be found in Roth et al *Lancet* 2018.[31]

eMethods 4. Conceptual diagram of sepsis as intermediate cause of death

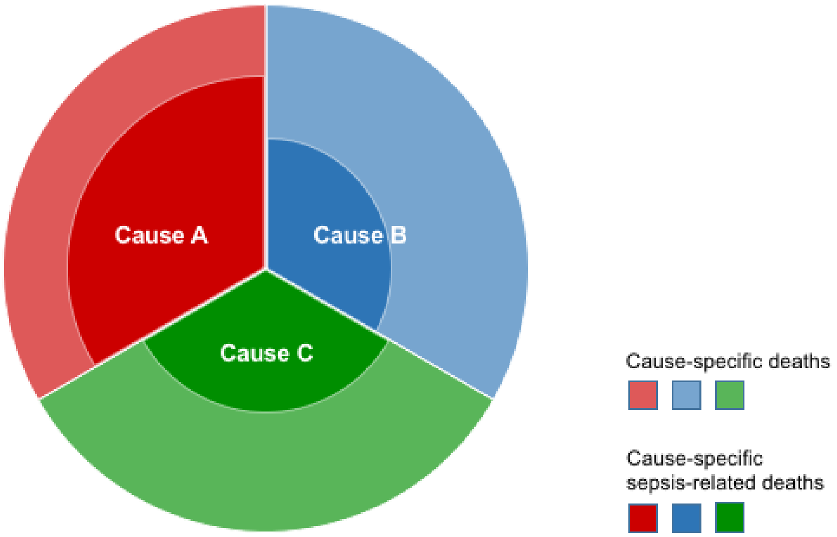

The 282 causes in the GBD 2017 study, classified according to underlying cause of health loss, are collectively exhaustive and mutually exclusive. This study identified sepsis cases and deaths from among all GBD causes, irrespective of whether the underlying cause was an infection, non-communicable disease, or injury. These cases and deaths can be considered to be dually labeled – first with one of the 282 underlying causes, and second with sepsis as an intermediate cause. Therefore, it is impossible to directly compare the burden of sepsis to any of the underlying causes.

eTable 1. International Classification of Diseases (ICD) 9th and 10th Revision codes for the identification of sepsis

	Explicit sepsis	Implicit sepsis	
ICD Revision	Explicit codes	Infection codes	Organ dysfunction codes
ICD-9	038-038.9, 090-097.9, 286.6, 635-639.9, 646.5-646.64, 658.4-658.93, 659.2-659.33, 670-670.9, 672-672.04, 674.1-674.34, 675-675.94, 771, 771.4-771.89, 800-801.99, 803-804.99, 905.0, 995.9-995.94	002-004.9, 006-009.9, 020-029, 032-033.9, 035-036.9, 040.0, 045-045.93, 047-049.9, 052-053.9, 054.72, 055-055.9, 060-066.9, 072-073, 073.7-073.9, 074.2, 074.21-074.23, 075-075.9, 078.3, 079.82, 080-084.9, 087-088.9, 100-100.9, 102-103.9, 121-125, 125.4-125.9, 127, 127.1-127.2, 127.4-129.0, 138, 310.89, 320-320.3, 320.5-326.9, 417-417.9, 420-424, 424.4-424.99, 429-429.9, 442-442.9, 443.21-443.29, 447-450, 456, 456.3, 457, 457.1-457.9, 458.0-458.1, 459-459.0, 459.2-459.9, 466-469, 470.0, 480-490.9, 510-511.9, 513.0-513.9, 540-542.9, 574-576.9, 590-590.9, 595-595.9, 597-597.9, 599.0, 601-601.9, 604-604.99, 680-689, 711-711.99, 728.86, 770.0, 785.4, V01.71-V01.79, V01.82, V01.84, V02.4, V03.5-V03.6, V04.2, V04.4, V04.6-V04.7, V04.81, V05.0-V05.1, V05.4, V12.03, V12.61, V73.2, V73.4-V73.5, V74.3, V75.1, V75.6, V75.8	293-294, 295.4-295.45, 295.80-295.95, 296.82-298.9, 300.5-302.9, 306-307.0, 307.2-307.49, 307.6-307.7, 307.9, 310-310.1, 313-313.9, 316-316.9, 327-327.19, 327.3-327.8, 347-347.9, 584-584.9, 780-780.2, 780.93, 780.97, 797-797.9, 799.2-799.29, V11.0-V11.2, V11.4-V12.0, V17-V17.0, V40-V41.9, V79-V79.9
ICD-10	A02.1-A02.9, A20.7-A20.9, A21.7-A21.9, A22.7-A22.9, A24.1-A24.9, A26.7-A26.9, A28.2-A28.9, A32.7-A32.9, A39.0, A39.4-A41.9, A42.7-A42.9, A50-A50.9, A54.86, B00.7-B00.9, B37.7-B37.9, N98.0, O03.0, O03.38, O03.5, O03.88, O04.5, O04.88, O07.38, O08.0, O08.83, O23-O23.93, O41.1-O41.93, O75.3, O85-O86.89, O88.3-O88.32, O91-O91.23, O98, O98.2-O98.93, P00.2, P22-P23.9, P29.12, P29.81, P35-P37, P37.1-P39.9, R65.2-R65.21, R68.13	A01-A02.0, A03-A09.9, A19-A20.3, A21-A21.3, A22-A22.2, A23-A24.0, A25-A26.0, A27-A28.1, A31-A32.12, A36-A39, A39.1-A39.3, A42-A42.2, A43-A46.0, A48-A49.9, A59-A59.9, A65-A65.0, A69-A69.1, A74, A74.8-A75.9, A77-A81.9, A83-A96.9, A98-B00.59, B01-B10.89, B25-B27.99, B29.4, B33-B34.9, B37-B37.6, B38-B50.9, B54-B55, B55.1-B55.9, B58-B60.8, B64, B67-B67.99, B91, B95-B99.9, G00-G08.0, G14-G14.6, H05.01-H05.039, H60.2-H60.23, H70.0-H70.009, I00, I02, I02.9, I26.01-I26.09, I26.90-I26.99, I33-I33.9, I38-I39.9, I40.0-I40.9, I76, I96-I96.9, I98.1, J01-J06.9, J09-J22.9, J36-J36.0, J39.0-J39.1, J85-J86.9, K35-K37.9, K57-K57.93, K61-K61.4, K63.0-K63.1, K65-K65.9, K67.8, K75.0-K75.1, K75.3, K76.3, K77.0, K81.0, K81.2, K83.0, K95.01, K95.81, L02-L08.9, M00-M02.9, M86-M86.9, M89.6-M89.69, N10-N10.9, N15.1-N15.9, N30-N30.91, N39.0, N41.0, N41.2-N41.3, N45-N45.9, N70-N77.8, R78.81, T80.2-T80.29, T81.4, T82.6-T82.7, T83.5, T83.6, T84.5-T84.7, T85.7, T88.0, U04	D65-D65.9, D69.5-D69.59, E87.2-E87.99, G93.4-G93.49, I46-I46.9, I95.1-I95.9, J80-J80.9, J95.2-J95.3, J96-J96.92, K72-K72.91, N00-N01.9, N17-N17.9, R09.02, R09.2, R40.0-R40.4, R41.82, R55-R55.0, R57-R57.9

Abbreviations: ICD=International Classification of Diseases.
Sepsis cases were identified using codes from the ICD 9th (ICD-9) and 10th (ICD-10) Revisions. Cases were classified within two mutually exclusive groups, “explicit” and “implicit.” Explicit sepsis cases were those with an ICD code explicitly referencing sepsis (“explicit code”) listed as a chain cause of death or secondary admission diagnosis. Implicit sepsis cases were those with both an “infection code” listed as the underlying cause of death or primary admission diagnosis and an “organ dysfunction code” listed as a chain cause of death or secondary admission diagnosis. ICD codes were adapted from the Angus criteria,[1] and were classified by the study team and validated by an expert panel. If a case met criteria for both case definitions, it was classified as explicit. Total sepsis estimates are based upon both explicit and implicit sepsis cases.

eTable 2. Custom cause groups for infections, injuries, and non-communicable diseases

GBD cause	Custom cause group	GBD cause level
HIV/AIDS	Infections	3
HIV/AIDS - Drug-susceptible tuberculosis	Infections	4
HIV/AIDS - Multidrug-resistant tuberculosis without extensive drug resistance	Infections	4
HIV/AIDS - Extensively drug-resistant tuberculosis	Infections	4
HIV/AIDS resulting in other diseases	Infections	4
Sexually transmitted infections excluding HIV	Infections	3
Syphilis	Infections	4
Chlamydial infection	Infections	4
Gonococcal infection	Infections	4
Other sexually transmitted infections	Infections	4
Tuberculosis	Infections	3
Drug-susceptible tuberculosis	Infections	4
Multidrug-resistant tuberculosis without extensive drug resistance	Infections	4
Extensively drug-resistant tuberculosis	Infections	4
Lower respiratory infections	Infections	3
Upper respiratory infections	Infections	3
Otitis media	Infections	3
Diarrhoeal diseases	Infections	3
Typhoid fever	Infections	4
Paratyphoid fever	Infections	4
Invasive non-typhoidal *Salmonella*	Infections	3
Other intestinal infectious diseases	Infections	3
Malaria	Infections	3
Chagas disease	Infections	3
Leishmaniasis	Infections	3
Visceral leishmaniasis	Infections	4
African trypanosomiasis	Infections	3
Schistosomiasis	Infections	3
Cysticercosis	Infections	3
Cystic echinococcosis	Infections	3
Dengue	Infections	3
Yellow fever	Infections	3
Rabies	Infections	3
Intestinal nematode infections	Infections	3
Ascariasis	Infections	4

Ebola virus disease	Infections	3
Zika virus disease	Infections	3
Other neglected tropical diseases	Infections	3
Meningitis	Infections	3
Pneumococcal meningitis	Infections	4
H influenzae type B meningitis	Infections	4
Meningococcal infection	Infections	4
Other meningitis	Infections	4
Encephalitis	Infections	3
Diphtheria	Infections	3
Whooping cough	Infections	3
Tetanus	Infections	3
Measles	Infections	3
Varicella and herpes zoster	Infections	3
Acute hepatitis	Infections	3
Acute hepatitis A	Infections	4
Acute hepatitis B	Infections	4
Acute hepatitis C	Infections	4
Acute hepatitis E	Infections	4
Other unspecified infectious diseases	Infections	3
Maternal disorders	Non-Communicable Diseases	3
Maternal haemorrhage	Non-Communicable Diseases	4
Maternal sepsis and other pregnancy-related infections	Infections	4
Maternal hypertensive disorders	Non-Communicable Diseases	4
Maternal obstructed labour and uterine rupture	Non-Communicable Diseases	4
Maternal abortive outcome	Non-Communicable Diseases	4
Ectopic pregnancy	Non-Communicable Diseases	4
Indirect maternal deaths	Non-Communicable Diseases	4
Late maternal deaths	Non-Communicable Diseases	4
Maternal deaths aggravated by HIV/AIDS	Infections	4
Other maternal disorders	Non-Communicable Diseases	4
Other maternal disorders	Infections	4
Neonatal disorders	Non-Communicable Diseases	3
Neonatal preterm birth	Non-Communicable Diseases	4
Neonatal encephalopathy due to birth asphyxia and trauma	Non-Communicable Diseases	4
Neonatal sepsis and other neonatal infections	Infections	4
Haemolytic disease and other neonatal jaundice	Non-Communicable Diseases	4
Other neonatal disorders	Non-Communicable Diseases	4
Nutritional deficiencies	Non-Communicable Diseases	2

Protein-energy malnutrition	Non-Communicable Diseases	3
Other nutritional deficiencies	Non-Communicable Diseases	3
Lip and oral cavity cancer	Non-Communicable Diseases	3
Nasopharynx cancer	Non-Communicable Diseases	3
Other pharynx cancer	Non-Communicable Diseases	3
Oesophageal cancer	Non-Communicable Diseases	3
Stomach cancer	Non-Communicable Diseases	3
Colon and rectum cancer	Non-Communicable Diseases	3
Liver cancer	Non-Communicable Diseases	3
Liver cancer due to hepatitis B	Infections	4
Liver cancer due to hepatitis C	Infections	4
Liver cancer due to alcohol use	Non-Communicable Diseases	4
Liver cancer due to NASH	Non-Communicable Diseases	4
Liver cancer due to other causes	Non-Communicable Diseases	4
Gallbladder and biliary tract cancer	Non-Communicable Diseases	3
Pancreatic cancer	Non-Communicable Diseases	3
Larynx cancer	Non-Communicable Diseases	3
Tracheal, bronchus, and lung cancer	Non-Communicable Diseases	3
Malignant skin melanoma	Non-Communicable Diseases	3
Non-melanoma skin cancer (squamous-cell carcinoma)	Non-Communicable Diseases	4
Breast cancer	Non-Communicable Diseases	3
Cervical cancer	Non-Communicable Diseases	3
Uterine cancer	Non-Communicable Diseases	3
Ovarian cancer	Non-Communicable Diseases	3
Prostate cancer	Non-Communicable Diseases	3
Testicular cancer	Non-Communicable Diseases	3
Kidney cancer	Non-Communicable Diseases	3
Bladder cancer	Non-Communicable Diseases	3
Brain and nervous system cancer	Non-Communicable Diseases	3
Thyroid cancer	Non-Communicable Diseases	3
Mesothelioma	Non-Communicable Diseases	3
Hodgkin lymphoma	Non-Communicable Diseases	3
Non-Hodgkin lymphoma	Non-Communicable Diseases	3
Multiple myeloma	Non-Communicable Diseases	3
Leukaemia	Non-Communicable Diseases	3
Acute lymphoid leukaemia	Non-Communicable Diseases	4
Chronic lymphoid leukaemia	Non-Communicable Diseases	4
Acute myeloid leukaemia	Non-Communicable Diseases	4
Chronic myeloid leukaemia	Non-Communicable Diseases	4

Other leukaemia	Non-Communicable Diseases	4
Other malignant cancers	Non-Communicable Diseases	3
Other neoplasms	Non-Communicable Diseases	3
Myelodysplastic, myeloproliferative, and other haemopoietic neoplasms	Non-Communicable Diseases	4
Other benign and in situ neoplasms	Non-Communicable Diseases	4
Cardiovascular diseases	Non-Communicable Diseases	2
Rheumatic heart disease	Infections	3
Ischaemic heart disease	Non-Communicable Diseases	3
Stroke	Non-Communicable Diseases	3
Ischaemic stroke	Non-Communicable Diseases	4
Intracerebral haemorrhage	Non-Communicable Diseases	4
Subarachnoid haemorrhage	Non-Communicable Diseases	4
Hypertensive heart disease	Non-Communicable Diseases	3
Non-rheumatic valvular heart disease	Non-Communicable Diseases	3
Non-rheumatic calcific aortic valve disease	Non-Communicable Diseases	4
Non-rheumatic degenerative mitral valve disease	Non-Communicable Diseases	4
Other non-rheumatic valve diseases	Non-Communicable Diseases	4
Cardiomyopathy and myocarditis	Non-Communicable Diseases	3
Myocarditis	Infections	4
Alcoholic cardiomyopathy	Non-Communicable Diseases	4
Other cardiomyopathy	Non-Communicable Diseases	4
Atrial fibrillation and flutter	Non-Communicable Diseases	3
Aortic aneurysm	Non-Communicable Diseases	3
Peripheral vascular disease	Non-Communicable Diseases	3
Endocarditis	Infections	3
Other cardiovascular and circulatory diseases	Infections	3
Other cardiovascular and circulatory diseases	Non-Communicable Diseases	3
Chronic respiratory diseases	Non-Communicable Diseases	2
Chronic obstructive pulmonary disease	Non-Communicable Diseases	3
Pneumoconiosis	Non-Communicable Diseases	3
Silicosis	Non-Communicable Diseases	4
Asbestosis	Non-Communicable Diseases	4
Coal workers pneumoconiosis	Non-Communicable Diseases	4
Other pneumoconiosis	Non-Communicable Diseases	4
Asthma	Non-Communicable Diseases	3
Interstitial lung disease and pulmonary sarcoidosis	Non-Communicable Diseases	3
Other chronic respiratory diseases	Non-Communicable Diseases	3
Digestive diseases	Non-Communicable Diseases	2
Cirrhosis and other chronic liver diseases	Non-Communicable Diseases	3

Cirrhosis and other chronic liver diseases due to hepatitis B	Infections	4
Cirrhosis and other chronic liver diseases due to hepatitis C	Infections	4
Cirrhosis and other chronic liver diseases due to alcohol use	Non-Communicable Diseases	4
Cirrhosis due to NASH	Non-Communicable Diseases	4
Cirrhosis and other chronic liver diseases due to other causes	Non-Communicable Diseases	4
Upper digestive system diseases	Non-Communicable Diseases	3
Peptic ulcer disease	Non-Communicable Diseases	4
Gastritis and duodenitis	Non-Communicable Diseases	4
Appendicitis	Infections	3
Paralytic ileus and intestinal obstruction	Non-Communicable Diseases	3
Inguinal, femoral, and abdominal hernia	Non-Communicable Diseases	3
Inflammatory bowel disease	Non-Communicable Diseases	3
Vascular intestinal disorders	Non-Communicable Diseases	3
Gallbladder and biliary diseases	Non-Communicable Diseases	3
Pancreatitis	Non-Communicable Diseases	3
Other digestive diseases	Non-Communicable Diseases	3
Alzheimer's disease and other dementias	Non-Communicable Diseases	3
Parkinson's disease	Non-Communicable Diseases	3
Epilepsy	Non-Communicable Diseases	3
Multiple sclerosis	Non-Communicable Diseases	3
Motor neuron disease	Non-Communicable Diseases	3
Other neurological disorders	Non-Communicable Diseases	3
Eating disorders	Non-Communicable Diseases	3
Anorexia nervosa	Non-Communicable Diseases	4
Bulimia nervosa	Non-Communicable Diseases	4
Alcohol use disorders	Non-Communicable Diseases	3
Drug use disorders	Non-Communicable Diseases	3
Opioid use disorders	Non-Communicable Diseases	4
Cocaine use disorders	Non-Communicable Diseases	4
Amphetamine use disorders	Non-Communicable Diseases	4
Other drug use disorders	Non-Communicable Diseases	4
Diabetes mellitus	Non-Communicable Diseases	3
Diabetes mellitus type 1	Non-Communicable Diseases	4
Diabetes mellitus type 2	Non-Communicable Diseases	4
Chronic kidney disease	Non-Communicable Diseases	3
Chronic kidney disease due to diabetes mellitus type 1	Non-Communicable Diseases	4
Chronic kidney disease due to diabetes mellitus type 2	Non-Communicable Diseases	4
Chronic kidney disease due to hypertension	Non-Communicable Diseases	4
Chronic kidney disease due to glomerulonephritis	Non-Communicable Diseases	4

Chronic kidney disease due to other and unspecified causes	Non-Communicable Diseases	4
Acute glomerulonephritis	Non-Communicable Diseases	3
Skin and subcutaneous diseases	Non-Communicable Diseases	2
Bacterial skin diseases	Infections	3
Cellulitis	Infections	4
Pyoderma	Infections	4
Decubitus ulcer	Infections	3
Other skin and subcutaneous diseases	Non-Communicable Diseases	3
Musculoskeletal disorders	Non-Communicable Diseases	2
Rheumatoid arthritis	Non-Communicable Diseases	3
Other musculoskeletal disorders	Non-Communicable Diseases	3
Congenital anomalies	Non-Communicable Diseases	3
Neural tube defects	Non-Communicable Diseases	4
Congenital heart anomalies	Non-Communicable Diseases	4
Orofacial clefts	Non-Communicable Diseases	4
Down's syndrome	Non-Communicable Diseases	4
Other chromosomal abnormalities	Non-Communicable Diseases	4
Congenital musculoskeletal and limb anomalies	Non-Communicable Diseases	4
Urogenital congenital anomalies	Non-Communicable Diseases	4
Digestive congenital anomalies	Non-Communicable Diseases	4
Other congenital anomalies	Non-Communicable Diseases	4
Urinary diseases and male infertility	Non-Communicable Diseases	3
Urinary tract infections	Infections	4
Urolithiasis	Non-Communicable Diseases	4
Other urinary diseases	Non-Communicable Diseases	4
Gynaecological diseases	Non-Communicable Diseases	3
Uterine fibroids	Non-Communicable Diseases	4
Polycystic ovarian syndrome	Non-Communicable Diseases	4
Endometriosis	Non-Communicable Diseases	4
Genital prolapse	Non-Communicable Diseases	4
Other gynaecological diseases	Non-Communicable Diseases	4
Haemoglobinopathies and haemolytic anaemias	Non-Communicable Diseases	3
Thalassemias	Non-Communicable Diseases	4
Sickle cell disorders	Non-Communicable Diseases	4
G6PD deficiency	Non-Communicable Diseases	4
Other haemoglobinopathies and haemolytic anaemias	Non-Communicable Diseases	4
Endocrine, metabolic, blood, and immune disorders	Non-Communicable Diseases	3
Sudden infant death syndrome	Non-Communicable Diseases	3
Transport injuries	Injuries	2

Road injuries	Injuries	3
Pedestrian road injuries	Injuries	4
Cyclist road injuries	Injuries	4
Motorcyclist road injuries	Injuries	4
Motor vehicle road injuries	Injuries	4
Other road injuries	Injuries	4
Other transport injuries	Injuries	3
Falls	Injuries	3
Drowning	Injuries	3
Fire, heat, and hot substances	Injuries	3
Poisonings	Injuries	3
Poisoning by carbon monoxide	Injuries	4
Poisoning by other means	Injuries	4
Exposure to mechanical forces	Injuries	3
Unintentional firearm injuries	Injuries	4
Other exposure to mechanical forces	Injuries	4
Adverse effects of medical treatment	Injuries	3
Animal contact	Injuries	3
Venomous animal contact	Injuries	4
Non-venomous animal contact	Injuries	4
Foreign body	Injuries	3
Pulmonary aspiration and foreign body in airway	Injuries	4
Foreign body in other body part	Injuries	4
Environmental heat and cold exposure	Injuries	3
Exposure to forces of nature	Injuries	3
Other unintentional injuries	Injuries	3
Self-harm	Injuries	3
Self-harm by firearm	Injuries	4
Self-harm by other specified means	Injuries	4
Interpersonal violence	Injuries	3
Assault by firearm	Injuries	4
Assault by sharp object	Injuries	4
Assault by other means	Injuries	4
Conflict and terrorism	Injuries	3
Executions and police conflict	Injuries	3

Abbreviations: COPD=chronic obstructive pulmonary disease. HIV/AIDS=human immunodeficiency virus/acquired immune deficiency syndrome. NASH=non-alcoholic steatohepatitis.
Sepsis cases and deaths are reported according to the underlying GBD cause. The GBD cause list is structured as a hierarchy, with each level composed of causes that are mutually exclusive and collectively exhaustive. GBD Level 1 causes are three overarching categories (eg, non-communicable diseases). Level 2 causes contain 21 broad cause groups (eg, digestive diseases). Individual causes are primarily recorded at Level 3 (eg, appendicitis, diabetes mellitus, and pancreatitis). A subset of Level 3 causes are further disaggregated to Level 4 causes (eg, diabetes mellitus type 1 and type 2). Level 2, 3, and 4 causes have been categorised

into three custom Level 1 groups (infections, injuries, and non-communicable diseases) for the unique purposes of this analysis, solely to facilitate communication of the results. These custom cause groups were not used to model estimates – they are used only for the presentation of results – and differ from the cause levels used throughout the GBD project.[31]

eTable 3. Data sources for modelling sepsis mortality

Location	Data source	Location-years	Number of locations	Years	ICD classification	Individual records
Brazil	Mortality Information System (SIM)	485	27	1999-2016	ICD-10	19,284,410
Taiwan (province of China)	Cause of Death Data Statistics and Management System (CDDSM)	10	1	2008-2017	ICD-10	1,554,360
United States	National Vital Statistics System (NVSS)	1,836	51	1980-2015	ICD-9, ICD-10	82,897,520
Mexico	National Institute of Statistics and Geography (INEGI)	256	32	2009-2016	ICD-10	4,850,422

Abbreviations: ICD=International Classification of Diseases.
All data sources represent national population-level data, with no restrictions as to age, sex, or cause of death. We included all national population-level sources of multiple cause of death (MCOD) death certificate data available within the GBD database. Data from Brazil, the USA, and Mexico were extracted at the state level.

eTable 4. Data quality rating from 0 to 5 stars for multiple-cause vital registration data used in modelling sepsis mortality

Location	1980	1981	1982	1983	1984	1985	1986	1987	1988	1989	1990	1991	1992	1993	1994	1995	1996	1997	1998	1999	2000	2001	2002	2003	2004	2005	2006	2007	2008	2009	2010	2011	2012	2013	2014	2015	2016	2017
USA	5	5	5	5	5	5	5	5	5	5	5	5	5	5	5	5	5	5	5	5	5	5	5	5	5	5	5	5	5	5	5	5	5	5	5	5		
Alabama	4	4	5	4	5	4	4	4	4	4	4	4	4	4	4	4	4	4	4	4	4	4	4	4	4	4	4	4	4	4	4	4	4	4	4	4		
Alaska	5	4	5	5	5	5	5	5	5	5	5	5	5	5	5	5	5	5	5	5	5	5	5	5	5	5	5	5	5	5	5	5	4	4	4	4		
Arizona	5	5	5	5	5	5	5	5	5	5	5	5	5	5	5	5	5	5	5	5	5	5	5	5	5	5	5	5	5	5	5	5	5	5	5	5		
Arkansas	4	4	4	4	4	4	4	4	4	5	5	5	5	5	5	5	5	5	4	4	4	5	5	5	5	5	5	5	5	5	5	5	5	5	5	5		
California	5	5	5	5	5	5	5	5	5	5	5	5	5	5	5	5	5	5	5	5	5	5	5	5	5	5	5	5	5	5	5	5	5	5	5	5		
Colorado	5	5	5	5	5	5	5	5	5	5	5	5	5	5	5	5	5	5	5	5	5	5	5	5	5	5	5	5	5	5	5	5	5	5	5	5		
Connecticut	5	5	5	5	5	5	5	5	5	5	5	5	5	5	5	5	5	5	5	5	5	5	5	5	5	4	5	4	4	4	4	4	4	4	4	4		
Delaware	5	5	5	5	5	5	5	5	5	5	5	5	5	5	5	5	5	5	5	5	5	5	5	5	5	5	5	5	5	5	5	5	5	5	5	5		
Washington, DC	5	4	4	4	4	4	4	4	4	4	4	4	4	4	4	5	4	4	5	4	5	5	5	4	5	4	5	4	4	4	4	4	4	4	4	4		
Florida	5	5	5	5	5	5	5	5	5	5	5	5	5	5	5	5	5	5	5	5	5	5	5	5	5	5	5	5	5	5	5	5	5	5	5	5		
Georgia	5	5	5	5	5	5	5	5	5	5	5	5	5	5	5	5	5	5	4	5	5	4	4	4	4	4	4	4	4	4	4	4	4	4	4	4		
Hawaii	5	5	5	5	5	5	5	5	5	5	5	5	5	5	5	5	5	4	4	4	4	4	4	4	4	4	4	4	4	4	4	4	4	4	4	4		
Idaho	5	5	5	5	5	5	5	5	5	5	5	5	5	5	5	5	5	5	5	5	5	5	5	5	5	5	5	5	5	5	5	5	5	5	5	5		
Illinois	5	5	5	5	5	5	5	5	5	5	5	5	5	5	5	5	5	5	5	5	5	5	5	5	5	5	5	5	5	5	5	5	4	5	5	4		
Indiana	5	5	5	5	5	5	5	5	5	5	5	5	5	5	5	5	5	5	5	5	5	5	5	5	5	5	5	4	5	4	5	5	5	5	4	5		
Iowa	5	5	5	5	5	5	5	5	5	5	5	5	5	5	5	5	5	5	5	5	5	5	5	5	5	5	5	5	5	5	5	5	5	5	5	5		
Kansas	5	5	5	5	5	5	5	5	5	5	5	5	5	5	5	5	5	5	5	5	5	5	5	5	5	5	5	5	5	4	4	5	4	4	4	5		
Kentucky	5	5	5	5	5	5	5	5	5	5	5	5	5	5	5	5	5	5	5	5	5	5	5	5	4	5	4	4	4	4	4	4	4	4	4	4		
Louisiana	5	5	5	5	5	5	5	5	5	5	5	5	5	5	5	5	5	5	5	5	5	5	5	5	5	4	4	4	4	4	4	4	4	4	4	4		
Maine	5	5	5	5	5	5	5	5	5	5	5	5	5	5	5	5	5	5	5	5	5	5	5	5	5	5	5	5	5	5	5	5	5	5	5	5		
Maryland	5	5	5	5	5	5	5	5	5	5	5	5	5	5	5	5	5	5	5	5	5	5	5	5	5	5	5	5	5	5	5	5	5	5	5	5		
Massachusetts	5	5	5	5	5	5	5	5	5	5	5	5	5	5	5	5	5	5	4	5	5	5	4	4	5	4	4	4	5	4	5	5	4	4	4	4		
Michigan	5	5	5	5	5	5	5	5	5	5	5	5	5	5	5	5	5	5	5	5	5	5	5	5	5	5	5	5	5	5	5	5	5	5	5	5		
Minnesota	5	5	5	5	5	5	5	5	5	5	5	5	5	5	5	5	5	5	5	5	5	5	5	5	5	5	5	5	5	5	5	5	5	5	5	5		
Mississippi	4	4	4	4	4	4	5	5	5	5	5	5	5	5	5	5	5	5	5	5	5	5	5	4	4	4	4	4	4	4	4	4	4	4	4	4		
Missouri	5	5	5	5	5	5	5	5	5	5	5	5	5	5	5	5	5	5	5	5	5	5	5	5	5	5	5	5	5	5	5	5	5	5	5	5		
Montana	5	5	5	5	5	5	5	5	5	5	5	5	5	5	5	5	5	5	5	5	5	5	5	5	5	5	5	5	5	5	5	5	4	5	5	4		
Nebraska	5	5	5	5	5	5	5	5	5	5	5	5	5	4	5	5	4	4	4	5	5	4	5	5	4	5	5	5	5	5	5	5	5	5	5	5		
Nevada	5	5	5	5	5	5	5	5	5	5	5	5	5	5	5	5	5	5	5	5	5	4	4	4	4	4	4	4	4	4	4	4	4	4	5	5		
New Hampshire	5	5	5	5	5	5	5	5	5	5	5	5	5	5	5	5	5	5	5	5	5	5	5	5	5	5	5	5	5	5	5	5	5	5	5	5		

New Jersey	5	5	5	5	5	5	5	5	5	5	5	5	5	5	5	5	5	5	5	5	5	5	5	5	5	5	4	4	5	4	5	5	4	4	4	4		
New Mexico	5	4	5	5	5	5	5	5	5	5	5	5	5	5	5	5	5	5	5	5	5	5	5	5	5	5	5	5	5	5	5	5	5	5	5	5		
New York	5	5	5	5	5	5	5	5	5	5	5	5	5	5	5	5	5	5	5	5	5	5	5	5	5	5	5	5	5	5	5	5	5	5	5	5		
North Carolina	5	5	5	5	5	5	5	5	5	5	5	5	5	5	5	5	5	5	5	5	5	5	5	5	5	5	5	5	5	5	5	5	5	5	5	5		
North Dakota	5	5	5	5	5	5	5	5	5	5	5	5	5	5	5	5	5	5	5	5	5	5	5	5	5	5	5	5	5	5	5	4	4	4	4	4		
Ohio	5	5	5	5	5	5	5	5	5	5	5	5	5	5	5	5	5	5	5	5	5	5	5	5	5	5	5	5	5	4	5	5	5	5	5	4		
Oklahoma	5	5	5	5	5	5	5	5	5	5	5	5	5	5	5	5	5	5	5	5	5	5	5	5	5	5	5	5	4	4	5	5	5	5	5	5		
Oregon	5	5	5	5	5	5	5	5	5	5	5	5	5	5	5	5	5	5	5	5	5	5	5	5	5	5	5	5	5	5	5	5	5	5	5	5		
Pennsylvania	5	5	5	5	5	5	5	5	5	5	5	5	5	5	5	5	5	5	5	5	5	4	4	5	5	4	4	4	5	4	4	4	4	4	4	4		
Rhode Island	5	5	5	5	5	5	5	5	5	5	5	5	5	5	5	5	5	5	5	5	5	5	5	5	5	5	5	5	5	5	5	5	5	5	5	5		
South Carolina	5	5	5	5	5	5	5	5	5	5	5	5	5	5	5	5	5	5	5	5	5	5	5	5	5	5	5	5	5	5	5	5	5	5	5	5		
South Dakota	5	5	5	5	5	5	5	5	5	5	5	5	5	5	5	5	5	5	5	5	5	5	5	5	5	5	5	5	5	5	5	5	5	5	5	5		
Tennessee	5	5	5	5	5	5	5	5	5	5	5	5	5	5	5	5	5	5	5	5	5	5	5	5	5	5	5	5	5	5	5	5	5	5	5	5		
Texas	5	5	4	4	4	4	4	4	4	5	5	5	5	5	5	5	5	5	5	5	5	5	5	5	5	5	5	5	5	5	5	5	5	5	5	5		
Utah	5	5	5	5	5	5	5	5	5	5	5	5	5	5	5	5	5	5	5	5	5	4	4	4	4	4	4	4	4	4	4	4	4	4	4	4		
Vermont	5	5	5	5	5	5	5	5	5	5	5	5	5	5	5	5	5	5	5	5	5	5	5	5	5	5	5	5	5	5	5	5	5	5	5	5		
Virginia	5	5	5	5	5	5	5	5	5	5	5	5	5	5	5	5	5	5	5	5	5	5	5	5	5	5	5	5	4	5	4	5	5	5	5	5		
Washington	5	5	5	5	5	5	5	5	5	5	5	5	5	5	5	5	5	5	5	5	5	5	5	5	5	5	5	5	5	5	5	5	5	5	5	5		
West Virginia	5	5	5	5	5	5	5	5	5	5	5	5	5	5	5	5	5	5	5	5	5	5	5	5	5	4	4	4	4	4	4	4	4	4	4	4		
Wisconsin	5	5	5	5	5	5	5	5	5	5	5	5	5	5	5	5	5	5	5	5	5	5	5	5	5	5	5	5	5	5	5	5	5	5	5	5		
Wyoming	5	5	5	5	5	5	5	5	5	5	5	5	5	5	5	5	5	5	5	5	5	5	5	5	5	5	5	5	5	5	5	5	4	4	4	4		
Mexico																														4	5	4	5	5	5	5	5	
Aguascalientes																														5	5	4	5	5	5	5	5	
Baja California																														5	5	5	5	4	5	5	5	
Baja California Sur																														5	4	5	5	5	5	5	5	
Campeche																														4	4	4	5	5	5	5	5	
Chiapas																														4	4	4	4	4	4	4	4	
Chihuahua																														5	5	5	5	5	5	5	5	
Coahuila																														4	5	5	5	5	5	5	5	
Colima																														5	5	5	5	5	5	5	5	
Durango																														4	4	4	4	4	4	4	4	
Guanajuato																														5	5	5	5	5	5	5	5	
Guerrero																														4	4	4	4	4	4	4	4	
Hidalgo																														4	4	4	5	4	4	5	5	

Jalisco																														5	5	5	5	5	5	5	5	
México																														4	5	5	5	5	5	5	5	
Mexico City																														4	5	5	5	5	5	5	5	
Michoacán de Ocampo																														4	4	4	5	5	4	5	5	
Morelos																														4	5	4	5	5	5	5	5	
Nayarit																														4	5	5	5	5	5	5	5	
Nuevo León																														4	5	5	5	5	5	5	5	
Oaxaca																														4	4	4	4	4	4	4	5	
Puebla																														4	4	4	5	5	5	5	5	
Querétaro																														4	5	4	5	5	5	5	5	
Quintana Roo																														4	4	4	5	5	5	5	5	
San Luis Potosí																														4	4	4	5	5	5	4	5	
Sinaloa																														5	5	5	5	5	5	5	5	
Sonora																														4	5	5	5	5	5	5	5	
Tabasco																														4	5	4	5	5	5	5	5	
Tamaulipas																														4	5	5	5	5	5	5	5	
Tlaxcala																														4	4	4	5	4	5	5	5	
Veracruz de Ignacio de la Llave																														4	4	4	5	5	5	5	5	
Yucatán																														4	4	4	5	5	5	5	5	
Zacatecas																														4	4	4	5	5	5	5	5	
Brazil																				4	4	4	4	4	4	4	4	4	4	4	4	4	4	4	4	4	4	
Acre																				3	3	3	3	3	3	4	4	4	4	4	4	4	4	4	4	4	4	
Alagoas																				3	3	3	3	3	3	3	3	4	4	4	4	4	4	4	4	4	4	
Amapá																				4	3	4	4	4	4	4	3	3	4	4	4	4	4	4	4	4	4	
Amazonas																				3	3	3	3	3	4	4	4	4	4	4	4	4	4	4	4	4	4	
Bahia																				3	3	3	3	3	3	3	3	3	3	3	3	3	3	4	4	3	3	
Ceará																				3	3	3	3	3	3	3	3	3	3	4	3	4	4	4	4	4	4	
Distrito Federal																				5	5	5	5	5	5	5	5	5	5	5	5	5	5	5	5	5	5	
Espírito Santo																				4	4	4	4	4	4	4	5	5	5	5	5	5	5	5	5	5	5	
Goiás																				4	4	4	4	4	4	4	4	4	4	4	4	4	5	5	5	5	5	
Maranhão																				2	2	2	2	2	2	3	3	3	3	3	3	3	3	3	3	3	3	
Mato Grosso																				4	4	4	4	4	4	4	4	4	4	4	4	4	4	4	4	4	4	
Mato Grosso do Sul																				4	4	4	4	5	5	5	5	5	5	5	5	5	5	5	5	5	5	

Minas Gerais																				4	4	4	4	4	4	4	4	4	4	4	4	4	4	4	4	4	4	
Pará																				3	3	3	3	3	3	3	3	3	3	3	3	4	4	4	4	4	4	
Paraíba																				2	2	3	3	3	3	3	3	4	4	4	4	4	4	4	4	4	4	
Paraná																				4	4	4	4	4	4	4	4	4	4	4	5	4	4	5	5	5	5	
Pernambuco																				3	3	3	3	4	4	4	4	4	4	4	4	4	4	4	4	4	4	
Piauí																				2	3	3	3	3	3	3	3	3	4	4	4	4	4	4	4	4	4	
Rio de Janeiro																				4	4	4	4	4	4	4	4	4	4	4	4	4	4	4	4	4	4	
Rio Grande do Norte																				3	3	3	3	3	3	4	4	4	4	4	4	4	4	4	4	4	4	
Rio Grande do Sul																				4	4	4	4	4	4	5	5	5	5	5	5	5	5	5	5	5	5	
Rondônia																				4	4	4	4	4	4	4	4	4	4	4	4	4	4	4	4	4	4	
Roraima																				4	4	4	4	4	4	4	4	4	4	4	4	4	4	4	4	4	4	
Santa Catarina																				4	4	4	4	4	4	4	4	4	4	4	4	5	4	5	5	5	5	
São Paulo																				4	4	4	4	4	4	4	4	4	4	4	4	4	4	4	4	4	4	
Sergipe																				3	3	3	3	3	4	4	4	4	4	4	4	4	4	4	4	4	4	
Tocantins																				3	3	3	3	3	3	3	3	4	4	4	4	4	4	4	4	4	4	
Taiwan (province of China)																													4	4	4	4	4	4	5	5	5	5

GBD 2017 star ratings are displayed.[31] A star rating from 0 to 5 is used to quantify the quality of data available in a given location for the time interval estimated by GBD, with 5 stars representing the highest-quality data. Locations improve in the star rating as they increase availability, completeness, and detail of their mortality data and reduce the percentage of deaths coded to ill-defined garbage codes or highly aggregated causes. The exact methods for calculating stars can be found in Roth et al *Lancet* 2018.[31]

eTable 5. Global Burden of Disease 2017 age-sex restrictions

Cause	Minimum age	Maximum age	Sex restrictions
HIV/AIDS and sexually transmitted infections	7 days		
HIV/AIDS	28 days		
HIV/AIDS - Drug-susceptible tuberculosis	28 days		
HIV/AIDS - Multidrug-resistant tuberculosis without extensive drug resistance	28 days		
HIV/AIDS - Extensively drug-resistant tuberculosis	28 days		
HIV/AIDS resulting in other diseases	28 days		
Sexually transmitted infections excluding HIV			
Syphilis			
Chlamydial infection	10 years		
Gonococcal infection	10 years		
Other sexually transmitted infections	10 years		
Respiratory infections and tuberculosis	7 days		
Tuberculosis	28 days		
Drug-susceptible tuberculosis	28 days		
Multidrug-resistant tuberculosis without extensive drug resistance	28 days		
Extensively drug-resistant tuberculosis	28 days		
Lower respiratory infections			
Upper respiratory infections			
Otitis media			
Enteric infections			
Diarrhoeal diseases			
Typhoid and paratyphoid	7 days		
Typhoid fever	28 days		
Paratyphoid fever	28 days		
Invasive non-typhoidal salmonella	7 days		
Other intestinal infectious diseases	28 days		
Neglected tropical diseases and malaria			
Malaria	7 days		
Chagas disease	28 days		
Leishmaniasis	28 days		
Visceral leishmaniasis	28 days		
African trypanosomiasis	1 year		
Schistosomiasis	28 days		
Cysticercosis			
Cystic echinococcosis	1 year		
Dengue	28 days		
Yellow fever	7 days		
Rabies	28 days		

Intestinal nematode infections	28 days		
Ascariasis	28 days		
Ebola virus disease			
Zika virus disease			
Other neglected tropical diseases			
Other infectious diseases			
Meningitis			
Pneumococcal meningitis			
H influenzae type B meningitis			
Meningococcal infection			
Other meningitis			
Encephalitis			
Diphtheria	28 days	59 years	
Whooping cough	28 days	59 years	
Tetanus			
Measles	28 days	59 years	
Varicella and herpes zoster			
Acute hepatitis	28 days		
Acute hepatitis A	28 days		
Acute hepatitis B	28 days		
Acute hepatitis C	28 days		
Acute hepatitis E	28 days		
Other unspecified infectious diseases			
Maternal and neonatal disorders		54 years	
Maternal disorders	10 years	54 years	Females only
Maternal haemorrhage	10 years	54 years	Females only
Maternal sepsis and other pregnancy-related infections	10 years	54 years	Females only
Maternal hypertensive disorders	10 years	54 years	Females only
Maternal obstructed labour and uterine rupture	10 years	54 years	Females only
Maternal abortive outcome	10 years	54 years	Females only
Ectopic pregnancy	10 years	54 years	Females only
Indirect maternal deaths	10 years	54 years	Females only
Late maternal deaths	10 years	54 years	Females only
Maternal deaths aggravated by HIV/AIDS	10 years	54 years	Females only
Other maternal disorders	10 years	54 years	Females only
Neonatal disorders		4 years	
Neonatal preterm birth		4 years	
Neonatal encephalopathy due to birth asphyxia and trauma		4 years	
Neonatal sepsis and other neonatal infections		4 years	
Haemolytic disease and other neonatal jaundice		4 years	
Other neonatal disorders		4 years	

Nutritional deficiencies	28 days		
Protein-energy malnutrition	28 days		
Other nutritional deficiencies	28 days		
Neoplasms			
Lip and oral cavity cancer	15 years		
Nasopharynx cancer	5 years		
Other pharynx cancer	15 years		
Oesophageal cancer	15 years		
Stomach cancer	15 years		
Colon and rectum cancer	15 years		
Liver cancer	5 years		
Liver cancer due to hepatitis B	5 years		
Liver cancer due to hepatitis C	5 years		
Liver cancer due to alcohol use	15 years		
Liver cancer due to NASH	15 years		
Liver cancer due to other causes	5 years		
Gallbladder and biliary tract cancer	15 years		
Pancreatic cancer	15 years		
Larynx cancer	15 years		
Tracheal, bronchus, and lung cancer	15 years		
Malignant skin melanoma	15 years		
Non-melanoma skin cancer	15 years		
Non-melanoma skin cancer (squamous-cell carcinoma)	15 years		
Breast cancer	15 years		
Cervical cancer	15 years		Females only
Uterine cancer	15 years		Females only
Ovarian cancer	15 years		Females only
Prostate cancer	15 years		Males only
Testicular cancer	15 years		Males only
Kidney cancer			
Bladder cancer	15 years		
Brain and nervous system cancer			
Thyroid cancer	10 years		
Mesothelioma	15 years		
Hodgkin lymphoma			
Non-Hodgkin lymphoma			
Multiple myeloma	15 years		
Leukaemia			
Acute lymphoid leukaemia			
Chronic lymphoid leukaemia	15 years		
Acute myeloid leukaemia			

Chronic myeloid leukaemia	15 years		
Other leukaemia			
Other malignant cancers			
Other neoplasms			
Myelodysplastic, myeloproliferative, and other haemopoietic neoplasms			
Other benign and in situ neoplasms			
Cardiovascular diseases			
Rheumatic heart disease	1 year		
Ischaemic heart disease	15 years		
Stroke			
Ischaemic stroke			
Intracerebral haemorrhage			
Subarachnoid haemorrhage			
Hypertensive heart disease	15 years		
Non-rheumatic valvular heart disease	15 years		
Non-rheumatic calcific aortic valve disease	15 years		
Non-rheumatic degenerative mitral valve disease	15 years		
Other non-rheumatic valve diseases	15 years		
Cardiomyopathy and myocarditis			
Myocarditis			
Alcoholic cardiomyopathy	15 years		
Other cardiomyopathy			
Atrial fibrillation and flutter	30 years		
Aortic aneurysm	15 years		
Peripheral vascular disease	40 years		
Endocarditis			
Other cardiovascular and circulatory diseases			
Chronic respiratory diseases			
Chronic obstructive pulmonary disease	28 days		
Pneumoconiosis	15 years		
Silicosis	15 years		
Asbestosis	15 years		
Coal workers pneumoconiosis	15 years		
Other pneumoconiosis	15 years		
Asthma	1 year		
Interstitial lung disease and pulmonary sarcoidosis	1 year		
Other chronic respiratory diseases			
Digestive diseases			
Cirrhosis and other chronic liver diseases	1 year		
Cirrhosis and other chronic liver diseases due to hepatitis B	1 year		
Cirrhosis and other chronic liver diseases due to hepatitis C	1 year		

Cirrhosis and other chronic liver diseases due to alcohol use	15 years		
Cirrhosis due to NASH	15 years		
Cirrhosis and other chronic liver diseases due to other causes	1 year		
Upper digestive system diseases	1 year		
Peptic ulcer disease	1 year		
Gastritis and duodenitis	1 year		
Appendicitis	1 year		
Paralytic ileus and intestinal obstruction			
Inguinal, femoral, and abdominal hernia	1 year		
Inflammatory bowel disease	1 year		
Vascular intestinal disorders	1 year		
Gallbladder and biliary diseases	1 year		
Pancreatitis	1 year		
Other digestive diseases	1 year		
Neurological disorders	28 days		
Alzheimer's disease and other dementias	40 years		
Parkinson's disease	20 years		
Epilepsy	28 days		
Multiple sclerosis	20 years		
Motor neuron disease			
Other neurological disorders	28 days		
Mental disorders			
Eating disorders	5 years	49 years	
Anorexia nervosa	5 years	49 years	
Bulimia nervosa	5 years	49 years	
Substance use disorders			
Alcohol use disorders	15 years		
Drug use disorders			
Opioid use disorders			
Cocaine use disorders	15 years		
Amphetamine use disorders	15 years		
Other drug use disorders	15 years		
Diabetes and kidney diseases			
Diabetes mellitus			
Diabetes mellitus type 1			
Diabetes mellitus type 2	15 years		
Chronic kidney disease	28 days		
Chronic kidney disease due to diabetes mellitus type 1	28 days		
Chronic kidney disease due to diabetes mellitus type 2	28 days		
Chronic kidney disease due to hypertension	28 days		
Chronic kidney disease due to glomerulonephritis	28 days		

Chronic kidney disease due to other and unspecified causes	28 days		
Acute glomerulonephritis	28 days		
Skin and subcutaneous diseases	28 days		
Bacterial skin diseases	7 days		
Cellulitis	28 days		
Pyoderma	28 days		
Decubitus ulcer	1 years		
Other skin and subcutaneous diseases	28 days		
Musculoskeletal disorders	5 years		
Rheumatoid arthritis	5 years		
Other musculoskeletal disorders	5 years		
Other non-communicable diseases			
Congenital anomalies		69 years	
Neural tube defects		69 years	
Congenital heart anomalies		69 years	
Orofacial clefts		4 years	
Down's syndrome		69 years	
Other chromosomal abnormalities		69 years	
Congenital musculoskeletal and limb anomalies		69 years	
Urogenital congenital anomalies		69 years	
Digestive congenital anomalies		69 years	
Other congenital anomalies		69 years	
Urinary diseases and male infertility			
Urinary tract infections			
Urolithiasis	5 years		
Other urinary diseases			
Gynaecological diseases	15 years		Females only
Uterine fibroids	15 years		Females only
Polycystic ovarian syndrome	15 years	54 years	Females only
Endometriosis	15 years	54 years	Females only
Genital prolapse	15 years		Females only
Other gynaecological diseases	15 years		Females only
Haemoglobinopathies and haemolytic anaemias			
Thalassemias			
Sickle cell disorders			
G6PD deficiency			
Other haemoglobinopathies and haemolytic anaemias			
Endocrine, metabolic, blood, and immune disorders			
Sudden infant death syndrome	7 days	364 days	
Transport injuries			
Road injuries			

Pedestrian road injuries			
Cyclist road injuries	1 year		
Motorcyclist road injuries			
Motor vehicle road injuries			
Other road injuries			
Other transport injuries			
Unintentional injuries			
Falls			
Drowning			
Fire, heat, and hot substances			
Poisonings			
Poisoning by carbon monoxide			
Poisoning by other means			
Exposure to mechanical forces			
Unintentional firearm injuries			
Other exposure to mechanical forces			
Adverse effects of medical treatment			
Animal contact			
Venomous animal contact			
Non-venomous animal contact			
Foreign body			
Pulmonary aspiration and foreign body in airway			
Foreign body in other body part			
Environmental heat and cold exposure			
Exposure to forces of nature			
Other unintentional injuries			
Self-harm and interpersonal violence			
Self-harm	10 years		
Self-harm by firearm	10 years		
Self-harm by other specified means	10 years		
Interpersonal violence			
Assault by firearm			
Assault by sharp object			
Assault by other means			
Conflict and terrorism			
Executions and police conflict	28 days		

Abbreviations: COPD=chronic obstructive pulmonary disease. HIV/AIDS=human immunodeficiency virus/acquired immune deficiency syndrome. NASH=non-alcoholic steatohepatitis.
Age-sex restrictions are per the Global Burden of Disease study 2017.[31] When deaths violated these restrictions, they were redistributed proportionally among all causes.

eTable 6. Cause of death nesting hierarchy

GBD cause	Nesting group
HIV/AIDS	Infectious (b)
HIV/AIDS - Drug-susceptible tuberculosis	Infectious (b)
HIV/AIDS - Multidrug-resistant tuberculosis without extensive drug resistance	Infectious (b)
HIV/AIDS - Extensively drug-resistant tuberculosis	Infectious (b)
HIV/AIDS resulting in other diseases	Infectious (b)
Sexually transmitted infections excluding HIV	Infectious (a)
Syphilis	Infectious (a)
Chlamydial infection	Infectious (a)
Gonococcal infection	Infectious (a)
Other sexually transmitted infections	Infectious (a)
Tuberculosis	Infectious (b)
Drug-susceptible tuberculosis	Infectious (b)
Multidrug-resistant tuberculosis without extensive drug resistance	Infectious (b)
Extensively drug-resistant tuberculosis	Infectious (b)
Lower respiratory infections	Infectious (c)
Upper respiratory infections	Infectious (c)
Otitis media	Infectious (c)
Diarrhoeal diseases	Infectious (b)
Typhoid fever	Infectious (c)
Paratyphoid fever	Infectious (c)
Invasive non-typhoidal salmonella	Infectious (c)
Other intestinal infectious diseases	Infectious (b)
Other intestinal infectious diseases	Infectious (c)
Malaria	Neglected tropical diseases
Chagas disease	Neglected tropical diseases
Leishmaniasis	Neglected tropical diseases
Visceral leishmaniasis	Neglected tropical diseases
African trypanosomiasis	Neglected tropical diseases
Schistosomiasis	Neglected tropical diseases
Cysticercosis	Neglected tropical diseases
Cystic echinococcosis	Neglected tropical diseases
Dengue	Infectious (c)
Yellow fever	Neglected tropical diseases
Rabies	Neglected tropical diseases
Intestinal nematode infections	Neglected tropical diseases
Ascariasis	Neglected tropical diseases
Ebola virus disease	Infectious (c)
Zika virus disease	Neglected tropical diseases
Other neglected tropical diseases	Neglected tropical diseases

Meningitis	Infectious (c)
Pneumococcal meningitis	Infectious (c)
H influenzae type B meningitis	Infectious (c)
Meningococcal infection	Infectious (c)
Other meningitis	Infectious (c)
Encephalitis	Infectious (a)
Diphtheria	Infectious (a)
Whooping cough	Infectious (b)
Tetanus	Infectious (b)
Measles	Infectious (b)
Varicella and herpes zoster	Infectious (c)
Acute hepatitis	Infectious (a)
Acute hepatitis A	Infectious (a)
Acute hepatitis B	Infectious (a)
Acute hepatitis C	Infectious (a)
Acute hepatitis E	Infectious (a)
Other unspecified infectious diseases	Infectious (a)
Other unspecified infectious diseases	Infectious (c)
Maternal disorders	Maternal and neonatal disorders
Maternal haemorrhage	Maternal and neonatal disorders
Maternal sepsis and other pregnancy-related infections	Maternal and neonatal disorders
Maternal hypertensive disorders	Maternal and neonatal disorders
Maternal obstructed labour and uterine rupture	Maternal and neonatal disorders
Maternal abortive outcome	Maternal and neonatal disorders
Ectopic pregnancy	Maternal and neonatal disorders
Indirect maternal deaths	Maternal and neonatal disorders
Late maternal deaths	Maternal and neonatal disorders
Maternal deaths aggravated by HIV/AIDS	Maternal and neonatal disorders
Other maternal disorders	Maternal and neonatal disorders
Neonatal disorders	Maternal and neonatal disorders
Neonatal preterm birth	Maternal and neonatal disorders
Neonatal encephalopathy due to birth asphyxia and trauma	Maternal and neonatal disorders
Neonatal sepsis and other neonatal infections	Maternal and neonatal disorders
Haemolytic disease and other neonatal jaundice	Maternal and neonatal disorders
Other neonatal disorders	Maternal and neonatal disorders
Protein-energy malnutrition	Nutritional deficiencies
Other nutritional deficiencies	Nutritional deficiencies
Lip and oral cavity cancer	Neoplasms (non-blood)
Nasopharynx cancer	Neoplasms (non-blood)
Other pharynx cancer	Neoplasms (non-blood)
Oesophageal cancer	Neoplasms (non-blood)

Stomach cancer	Neoplasms (non-blood)
Colon and rectum cancer	Neoplasms (non-blood)
Liver cancer	Neoplasms (non-blood)
Liver cancer due to hepatitis B	Neoplasms (non-blood)
Liver cancer due to hepatitis C	Neoplasms (non-blood)
Liver cancer due to alcohol use	Neoplasms (non-blood)
Liver cancer due to NASH	Neoplasms (non-blood)
Liver cancer due to other causes	Neoplasms (non-blood)
Gallbladder and biliary tract cancer	Neoplasms (non-blood)
Pancreatic cancer	Neoplasms (non-blood)
Larynx cancer	Neoplasms (non-blood)
Tracheal, bronchus, and lung cancer	Neoplasms (non-blood)
Malignant skin melanoma	Neoplasms (non-blood)
Non-melanoma skin cancer (squamous-cell carcinoma)	Neoplasms (non-blood)
Breast cancer	Neoplasms (non-blood)
Cervical cancer	Neoplasms (non-blood)
Uterine cancer	Neoplasms (non-blood)
Ovarian cancer	Neoplasms (non-blood)
Prostate cancer	Neoplasms (non-blood)
Testicular cancer	Neoplasms (non-blood)
Kidney cancer	Neoplasms (non-blood)
Bladder cancer	Neoplasms (non-blood)
Brain and nervous system cancer	Neoplasms (non-blood)
Thyroid cancer	Neoplasms (non-blood)
Mesothelioma	Neoplasms (non-blood)
Hodgkin lymphoma	Neoplasms (blood)
Non-Hodgkin lymphoma	Neoplasms (blood)
Multiple myeloma	Neoplasms (blood)
Leukaemia	Neoplasms (blood)
Acute lymphoid leukaemia	Neoplasms (blood)
Chronic lymphoid leukaemia	Neoplasms (blood)
Acute myeloid leukaemia	Neoplasms (blood)
Chronic myeloid leukaemia	Neoplasms (blood)
Other leukaemia	Neoplasms (blood)
Other malignant cancers	Neoplasms (non-blood)
Other neoplasms	Neoplasms (non-blood)
Myelodysplastic, myeloproliferative, and other haemopoietic neoplasms	Neoplasms (non-blood)
Other benign and in situ neoplasms	Neoplasms (non-blood)
Rheumatic heart disease	Cardiovascular diseases
Ischaemic heart disease	Cardiovascular diseases
Stroke	Cardiovascular diseases

Ischaemic stroke	Cardiovascular diseases
Intracerebral haemorrhage	Cardiovascular diseases
Subarachnoid haemorrhage	Cardiovascular diseases
Hypertensive heart disease	Cardiovascular diseases
Non-rheumatic valvular heart disease	Cardiovascular diseases
Non-rheumatic calcific aortic valve disease	Cardiovascular diseases
Non-rheumatic degenerative mitral valve disease	Cardiovascular diseases
Other non-rheumatic valve diseases	Cardiovascular diseases
Cardiomyopathy and myocarditis	Cardiovascular diseases
Myocarditis	Cardiovascular diseases
Alcoholic cardiomyopathy	Cardiovascular diseases
Other cardiomyopathy	Cardiovascular diseases
Atrial fibrillation and flutter	Cardiovascular diseases
Aortic aneurysm	Cardiovascular diseases
Peripheral vascular disease	Cardiovascular diseases
Endocarditis	Infectious (c)
Other cardiovascular and circulatory diseases	Infectious (a)
Other cardiovascular and circulatory diseases	Cardiovascular diseases
Chronic obstructive pulmonary disease	Respiratory diseases
Pneumoconiosis	Respiratory diseases
Silicosis	Respiratory diseases
Asbestosis	Respiratory diseases
Coal workers pneumoconiosis	Respiratory diseases
Other pneumoconiosis	Respiratory diseases
Asthma	Respiratory diseases
Interstitial lung disease and pulmonary sarcoidosis	Respiratory diseases
Other chronic respiratory diseases	Respiratory diseases
Cirrhosis and other chronic liver diseases	Digestive diseases
Cirrhosis and other chronic liver diseases due to hepatitis B	Digestive diseases
Cirrhosis and other chronic liver diseases due to hepatitis C	Digestive diseases
Cirrhosis and other chronic liver diseases due to alcohol use	Digestive diseases
Cirrhosis due to NASH	Digestive diseases
Cirrhosis and other chronic liver diseases due to other causes	Digestive diseases
Upper digestive system diseases	Digestive diseases
Peptic ulcer disease	Digestive diseases
Gastritis and duodenitis	Digestive diseases
Appendicitis	Infectious (a)
Paralytic ileus and intestinal obstruction	Infectious (a)
Inguinal, femoral, and abdominal hernia	Infectious (a)
Inflammatory bowel disease	Digestive diseases
Vascular intestinal disorders	Digestive diseases

Gallbladder and biliary diseases	Digestive diseases
Pancreatitis	Digestive diseases
Other digestive diseases	Digestive diseases
Alzheimer's disease and other dementias	Other non-communicable diseases
Parkinson's disease	Other non-communicable diseases
Epilepsy	Other non-communicable diseases
Multiple sclerosis	Other non-communicable diseases
Motor neuron disease	Other non-communicable diseases
Other neurological disorders	Other non-communicable diseases
Eating disorders	Other non-communicable diseases
Anorexia nervosa	Other non-communicable diseases
Bulimia nervosa	Other non-communicable diseases
Alcohol use disorders	Mental disorders
Drug use disorders	Mental disorders
Opioid use disorders	Mental disorders
Cocaine use disorders	Mental disorders
Amphetamine use disorders	Mental disorders
Other drug use disorders	Mental disorders
Diabetes mellitus	Diabetes and kidney diseases
Diabetes mellitus type 1	Diabetes and kidney diseases
Diabetes mellitus type 2	Diabetes and kidney diseases
Chronic kidney disease	Diabetes and kidney diseases
Chronic kidney disease due to diabetes mellitus type 1	Diabetes and kidney diseases
Chronic kidney disease due to diabetes mellitus type 2	Diabetes and kidney diseases
Chronic kidney disease due to hypertension	Diabetes and kidney diseases
Chronic kidney disease due to glomerulonephritis	Diabetes and kidney diseases
Chronic kidney disease due to other and unspecified causes	Diabetes and kidney diseases
Acute glomerulonephritis	Diabetes and kidney diseases
Skin and subcutaneous diseases	Other non-communicable diseases
Bacterial skin diseases	Infectious (c)
Cellulitis	Infectious (c)
Pyoderma	Infectious (c)
Decubitus ulcer	Infectious (c)
Other skin and subcutaneous diseases	Other non-communicable diseases
Musculoskeletal disorders	Other non-communicable diseases
Rheumatoid arthritis	Other non-communicable diseases
Other musculoskeletal disorders	Other non-communicable diseases
Congenital anomalies	Other non-communicable diseases
Neural tube defects	Other non-communicable diseases
Congenital heart anomalies	Other non-communicable diseases
Orofacial clefts	Other non-communicable diseases

Down's syndrome	Other non-communicable diseases
Other chromosomal abnormalities	Other non-communicable diseases
Congenital musculoskeletal and limb anomalies	Other non-communicable diseases
Urogenital congenital anomalies	Other non-communicable diseases
Digestive congenital anomalies	Other non-communicable diseases
Other congenital anomalies	Other non-communicable diseases
Urinary diseases and male infertility	Urinary and gynaecological diseases
Urinary tract infections	Infectious (c)
Urolithiasis	Infectious (c)
Other urinary diseases	Urinary and gynaecological diseases
Gynaecological diseases	Urinary and gynaecological diseases
Uterine fibroids	Urinary and gynaecological diseases
Polycystic ovarian syndrome	Other non-communicable diseases
Endometriosis	Urinary and gynaecological diseases
Genital prolapse	Urinary and gynaecological diseases
Other gynaecological diseases	Urinary and gynaecological diseases
Haemoglobinopathies and haemolytic anaemias	Other non-communicable diseases
Thalassemias	Other non-communicable diseases
Sickle cell disorders	Other non-communicable diseases
G6PD deficiency	Other non-communicable diseases
Other haemoglobinopathies and haemolytic anaemias	Other non-communicable diseases
Endocrine, metabolic, blood, and immune disorders	Other non-communicable diseases
Sudden infant death syndrome	Other non-communicable diseases
Transport injuries	Injuries (b)
Road injuries	Injuries (b)
Pedestrian road injuries	Injuries (b)
Cyclist road injuries	Injuries (b)
Motorcyclist road injuries	Injuries (b)
Motor vehicle road injuries	Injuries (b)
Other road injuries	Injuries (b)
Other transport injuries	Injuries (b)
Falls	Injuries (b)
Drowning	Injuries (a)
Fire, heat, and hot substances	Injuries (b)
Poisonings	Injuries (a)
Poisoning by carbon monoxide	Injuries (a)
Poisoning by other means	Injuries (a)
Exposure to mechanical forces	Injuries (b)
Unintentional firearm injuries	Injuries (b)
Other exposure to mechanical forces	Injuries (b)
Adverse effects of medical treatment	Injuries (b)

Animal contact	Injuries (b)
Venomous animal contact	Injuries (b)
Non-venomous animal contact	Injuries (b)
Foreign body	Injuries (a)
Pulmonary aspiration and foreign body in airway	Injuries (a)
Foreign body in other body part	Injuries (a)
Environmental heat and cold exposure	Injuries (a)
Exposure to forces of nature	Injuries (b)
Other unintentional injuries	Injuries (a)
Other unintentional injuries	Injuries (b)
Self-harm	Injuries (b)
Self-harm by firearm	Injuries (b)
Self-harm by other specified means	Injuries (a)
Self-harm by other specified means	Injuries (b)
Interpersonal violence	Injuries (b)
Assault by firearm	Injuries (b)
Assault by sharp object	Injuries (b)
Assault by other means	Injuries (b)
Conflict and terrorism	Injuries (b)
Executions and police conflict	Injuries (b)

Abbreviations: COPD=chronic obstructive pulmonary disease. HIV/AIDS=human immunodeficiency virus/acquired immune deficiency syndrome. NASH=non-alcoholic steatohepatitis.
The models used to estimate mortality and incidence used a nested random effects structure on the underlying cause. The hierarchal nesting allowed the model to make predictions sepsis fraction (mortality estimates) or case fatality rate (incidence estimates) for diseases not represented in the input data through borrowing information from diseases within the same group. All underlying causes were categorised into 17 groups according to physiological relatedness.

eTable 7. Global Burden of Disease 2017 location hierarchy with levels

Geography	Level
Global	0
Low SDI	1
Low-middle SDI	1
Middle SDI	1
High-middle SDI	1
High SDI	1
Central Europe, eastern Europe, and central Asia	1
Central Asia	2
Armenia	3
Azerbaijan	3
Georgia	3
Kazakhstan	3
Kyrgyzstan	3
Mongolia	3
Tajikistan	3
Turkmenistan	3
Uzbekistan	3
Central Europe	2
Albania	3
Bosnia and Herzegovina	3
Bulgaria	3
Croatia	3
Czech Republic	3
Hungary	3
Montenegro	3
North Macedonia	3
Poland	3
Romania	3
Serbia	3
Slovakia	3
Slovenia	3
Eastern Europe	2
Belarus	3
Estonia	3
Latvia	3
Lithuania	3

Moldova	3
Russia	3
Ukraine	3
High-income	1
Australasia	2
Australia	3
New Zealand	3
High-income Asia Pacific	2
Brunei	3
Japan	3
Aichi	4
Akita	4
Aomori	4
Chiba	4
Ehime	4
Fukui	4
Fukuoka	4
Fukushima	4
Gifu	4
Gunma	4
Hiroshima	4
Hokkaidō	4
Hyōgo	4
Ibaraki	4
Ishikawa	4
Iwate	4
Kagawa	4
Kagoshima	4
Kanagawa	4
Kōchi	4
Kumamoto	4
Kyōto	4
Mie	4
Miyagi	4
Miyazaki	4
Nagano	4
Nagasaki	4
Nara	4
Niigata	4

Ōita	4
Okayama	4
Okinawa	4
Ōsaka	4
Saga	4
Saitama	4
Shiga	4
Shimane	4
Shizuoka	4
Tochigi	4
Tokushima	4
Tōkyō	4
Tottori	4
Toyama	4
Wakayama	4
Yamagata	4
Yamaguchi	4
Yamanashi	4
South Korea	3
Singapore	3
High-income North America	2
Canada	3
Greenland	3
United States	3
Alabama	4
Alaska	4
Arizona	4
Arkansas	4
California	4
Colorado	4
Connecticut	4
Delaware	4
District of Columbia	4
Florida	4
Georgia	4
Hawaii	4
Idaho	4
Illinois	4
Indiana	4

Iowa	4
Kansas	4
Kentucky	4
Louisiana	4
Maine	4
Maryland	4
Massachusetts	4
Michigan	4
Minnesota	4
Mississippi	4
Missouri	4
Montana	4
Nebraska	4
Nevada	4
New Hampshire	4
New Jersey	4
New Mexico	4
New York	4
North Carolina	4
North Dakota	4
Ohio	4
Oklahoma	4
Oregon	4
Pennsylvania	4
Rhode Island	4
South Carolina	4
South Dakota	4
Tennessee	4
Texas	4
Utah	4
Vermont	4
Virginia	4
Washington	4
West Virginia	4
Wisconsin	4
Wyoming	4
Southern Latin America	2
Argentina	3
Chile	3

Uruguay	3
Western Europe	2
Andorra	3
Austria	3
Belgium	3
Cyprus	3
Denmark	3
Finland	3
France	3
Germany	3
Greece	3
Iceland	3
Ireland	3
Israel	3
Italy	3
Luxembourg	3
Malta	3
Netherlands	3
Norway	3
Portugal	3
Spain	3
Sweden	3
Stockholm	4
Sweden except Stockholm	4
Switzerland	3
United Kingdom	3
England	4
East Midlands	5
Derby	6
Derbyshire	6
Leicester	6
Leicestershire	6
Lincolnshire	6
Northamptonshire	6
Nottingham	6
Nottinghamshire	6
Rutland	6
East of England	5
Bedford	6

Cambridgeshire	6
Central Bedfordshire	6
Essex	6
Hertfordshire	6
Luton	6
Norfolk	6
Peterborough	6
Southend-on-Sea	6
Suffolk	6
Thurrock	6
Greater London	5
Barking and Dagenham	6
Barnet	6
Bexley	6
Brent	6
Bromley	6
Camden	6
Croydon	6
Ealing	6
Enfield	6
Greenwich	6
Hackney	6
Hammersmith and Fulham	6
Haringey	6
Harrow	6
Havering	6
Hillingdon	6
Hounslow	6
Islington	6
Kensington and Chelsea	6
Kingston upon Thames	6
Lambeth	6
Lewisham	6
Merton	6
Newham	6
Redbridge	6
Richmond upon Thames	6
Southwark	6
Sutton	6

Tower Hamlets	6
Waltham Forest	6
Wandsworth	6
Westminster	6
North East England	5
County Durham	6
Darlington	6
Gateshead	6
Hartlepool	6
Middlesbrough	6
Newcastle upon Tyne	6
North Tyneside	6
Northumberland	6
Redcar and Cleveland	6
South Tyneside	6
Stockton-on-Tees	6
Sunderland	6
North West England	5
Blackburn with Darwen	6
Blackpool	6
Bolton	6
Bury	6
Cheshire East	6
Cheshire West and Chester	6
Cumbria	6
Halton	6
Knowsley	6
Lancashire	6
Liverpool	6
Manchester	6
Oldham	6
Rochdale	6
Salford	6
Sefton	6
St Helens	6
Stockport	6
Tameside	6
Trafford	6
Warrington	6

Wigan	6
Wirral	6
South East England	5
Bracknell Forest	6
Brighton and Hove	6
Buckinghamshire	6
East Sussex	6
Hampshire	6
Isle of Wight	6
Kent	6
Medway	6
Milton Keynes	6
Oxfordshire	6
Portsmouth	6
Reading	6
Slough	6
Southampton	6
Surrey	6
West Berkshire	6
West Sussex	6
Windsor and Maidenhead	6
Wokingham	6
South West England	5
Bath and North East Somerset	6
Bournemouth	6
Bristol, City of	6
Cornwall	6
Devon	6
Dorset	6
Gloucestershire	6
North Somerset	6
Plymouth	6
Poole	6
Somerset	6
South Gloucestershire	6
Swindon	6
Torbay	6
Wiltshire	6
West Midlands	5

Birmingham	6
Coventry	6
Dudley	6
Herefordshire, County of	6
Sandwell	6
Shropshire	6
Solihull	6
Staffordshire	6
Stoke-on-Trent	6
Telford and Wrekin	6
Walsall	6
Warwickshire	6
Wolverhampton	6
Worcestershire	6
Yorkshire and the Humber	5
Barnsley	6
Bradford	6
Calderdale	6
Doncaster	6
East Riding of Yorkshire	6
Kingston upon Hull, City of	6
Kirklees	6
Leeds	6
North East Lincolnshire	6
North Lincolnshire	6
North Yorkshire	6
Rotherham	6
Sheffield	6
Wakefield	6
York	6
Northern Ireland	4
Scotland	4
Wales	4
Latin America and Caribbean	1
Andean Latin America	2
Bolivia	3
Ecuador	3
Peru	3
Caribbean	2

Antigua and Barbuda	3
The Bahamas	3
Barbados	3
Belize	3
Bermuda	3
Cuba	3
Dominica	3
Dominican Republic	3
Grenada	3
Guyana	3
Haiti	3
Jamaica	3
Puerto Rico	3
Saint Lucia	3
Saint Vincent and the Grenadines	3
Suriname	3
Trinidad and Tobago	3
Virgin Islands	3
Central Latin America	2
Colombia	3
Costa Rica	3
El Salvador	3
Guatemala	3
Honduras	3
Mexico	3
Aguascalientes	4
Baja California	4
Baja California Sur	4
Campeche	4
Chiapas	4
Chihuahua	4
Coahuila	4
Colima	4
Mexico City	4
Durango	4
Guanajuato	4
Guerrero	4
Hidalgo	4
Jalisco	4

México	4
Michoacán de Ocampo	4
Morelos	4
Nayarit	4
Nuevo León	4
Oaxaca	4
Puebla	4
Querétaro	4
Quintana Roo	4
San Luis Potosí	4
Sinaloa	4
Sonora	4
Tabasco	4
Tamaulipas	4
Tlaxcala	4
Veracruz de Ignacio de la Llave	4
Yucatán	4
Zacatecas	4
Nicaragua	3
Panama	3
Venezuela	3
Tropical Latin America	2
Brazil	3
Acre	4
Alagoas	4
Amapá	4
Amazonas	4
Bahia	4
Ceará	4
Distrito Federal	4
Espírito Santo	4
Goiás	4
Maranhão	4
Mato Grosso	4
Mato Grosso do Sul	4
Minas Gerais	4
Pará	4
Paraíba	4
Paraná	4

Pernambuco	4
Piaui	4
Rio de Janeiro	4
Rio Grande do Norte	4
Rio Grande do Sul	4
Rondônia	4
Roraima	4
Santa Catarina	4
São Paulo	4
Sergipe	4
Tocantins	4
Paraguay	3
North Africa and Middle East	1
North Africa and Middle East	2
Afghanistan	3
Algeria	3
Bahrain	3
Egypt	3
Iran	3
Iraq	3
Jordan	3
Kuwait	3
Lebanon	3
Libya	3
Morocco	3
Palestine	3
Oman	3
Qatar	3
Saudi Arabia	3
Sudan	3
Syria	3
Tunisia	3
Turkey	3
United Arab Emirates	3
Yemen	3
South Asia	1
South Asia	2
Bangladesh	3
Bhutan	3

India	3
Andhra Pradesh	4
Arunachal Pradesh	4
Assam	4
Bihar	4
Chhattisgarh	4
Delhi	4
Goa	4
Gujarat	4
Haryana	4
Himachal Pradesh	4
Jammu and Kashmir	4
Jharkhand	4
Karnataka	4
Kerala	4
Madhya Pradesh	4
Maharashtra	4
Manipur	4
Meghalaya	4
Mizoram	4
Nagaland	4
Odisha	4
Punjab	4
Rajasthan	4
Sikkim	4
Tamil Nadu	4
Telangana	4
Tripura	4
Uttar Pradesh	4
Uttarakhand	4
West Bengal	4
Union Territories other than Delhi	4
Nepal	3
Pakistan	3
Southeast Asia, east Asia, and Oceania	1
East Asia	2
China	3
North Korea	3
Taiwan (province of China)	3

Oceania	2
American Samoa	3
Federated States of Micronesia	3
Fiji	3
Guam	3
Kiribati	3
Marshall Islands	3
Northern Mariana Islands	3
Papua New Guinea	3
Samoa	3
Solomon Islands	3
Tonga	3
Vanuatu	3
Southeast Asia	2
Cambodia	3
Indonesia	3
Laos	3
Malaysia	3
Maldives	3
Mauritius	3
Myanmar	3
Philippines	3
Sri Lanka	3
Seychelles	3
Thailand	3
Timor-Leste	3
Vietnam	3
Sub-Saharan Africa	1
Central sub-Saharan Africa	2
Angola	3
Central African Republic	3
Congo (Brazzaville)	3
Democratic Republic of the Congo	3
Equatorial Guinea	3
Gabon	3
Eastern sub-Saharan Africa	2
Burundi	3
Comoros	3
Djibouti	3

Eritrea	3
Ethiopia	3
Kenya	3
Baringo	4
Bomet	4
Bungoma	4
Busia	4
Elgeyo-Marakwet	4
Embu	4
Garissa	4
HomaBay	4
Isiolo	4
Kajiado	4
Kakamega	4
Kericho	4
Kiambu	4
Kilifi	4
Kirinyaga	4
Kisii	4
Kisumu	4
Kitui	4
Kwale	4
Laikipia	4
Lamu	4
Machakos	4
Makueni	4
Mandera	4
Marsabit	4
Meru	4
Migori	4
Mombasa	4
Murang’a	4
Nairobi	4
Nakuru	4
Nandi	4
Narok	4
Nyamira	4
Nyandarua	4
Nyeri	4

Samburu	4
Siaya	4
TaitaTaveta	4
TanaRiver	4
TharakaNithi	4
TransNzoia	4
Turkana	4
UasinGishu	4
Vihiga	4
Wajir	4
WestPokot	4
Madagascar	3
Malawi	3
Mozambique	3
Rwanda	3
Somalia	3
South Sudan	3
Tanzania	3
Uganda	3
Zambia	3
Southern sub-Saharan Africa	2
Botswana	3
Lesotho	3
Namibia	3
South Africa	3
Swaziland (eSwatini)	3
Zimbabwe	3
Western sub-Saharan Africa	2
Benin	3
Burkina Faso	3
Cameroon	3
Cape Verde	3
Chad	3
Côte d’Ivoire	3
The Gambia	3
Ghana	3
Guinea	3
Guinea-Bissau	3
Liberia	3

Mali	3
Mauritania	3
Niger	3
Nigeria	3
São Tomé and Príncipe	3
Senegal	3
Sierra Leone	3
Togo	3

The Global Burden of Disease Study 2017 location hierarchy lists geographical super-regions (level 1), regions (level 2), national locations (level 3), and subnational locations (levels 4, 5, and 6).[31] GBD created regions based on epidemiological similarity and geographical closeness. There are seven super-regions, which group the 21 regions on the basis of cause of death patterns.

eTable 8. Data sources for modelling sepsis incidence

Country	Data source	Location-years	Number of locations	Years	ICD classification	Individual records	Hospitalisations with sepsis
Austria	Austria Hospital Inpatient Discharges	14	1	2001–2014	ICD9, ICD10	37,632,608	357,772
Brazil	Brazil Hospital Information System	52	26	2015–2016	ICD10	21,792,143	602,753
Canada	Canada Discharge Abstract Database	16	1	1994–2009	ICD9, ICD10	2,390,381	22,312
Chile	Chile Hospital Discharge Information System	11	1	2001–2004, 2006–2012	ICD10	16,355,550	208,088
Georgia	Georgia Hospital Data	1	1	2014	ICD10	398,822	3,797
Italy	Italy – Hospital Inpatient Discharges	12	1	2005–2016	ICD9	110,811,752	2,002,710
Mexico	Mexico Automated Hospital Discharge System	160	32	2003, 2005, 2007–2009	ICD10	9,549,237	389,761
New Zealand	New Zealand National Minimum Dataset	32	2	2000–2015	ICD9, ICD10	8,159,218	256,072
Philippines	Philippines Health Insurance Corporation Claims	2	1	2013–2014	ICD10	7,742,328	329,417
United States	National Hospital Discharge Survey	31	1	1980–2010	ICD9	7,730,286	315,079
United States	States Inpatient Databases	78	13	2003–2008	ICD9	86,661,803	4,185,230

Abbreviations: ICD=International Classification of Diseases.
All data sources represent national population-level data, with no restrictions as to age, sex, or cause of death. We included all population-level sources of individual-level hospital admission or discharge data with multiple diagnoses available within the GBD database, with no ad-hoc exclusions. Data from Brazil, the United States, New Zealand, and Mexico were extracted at the subnational level.

eTable 9. Sepsis incidence by location for all ages, both sexes, and all underlying causes, 1990 and 2017

	All underlying causes			Underlying infection			Underlying non-communicable disease			Underlying injury		
**Location**	**Incident sepsis cases (95% UI), 2017**	**Percentage change in sepsis ASIR (95% UI), 1990-2017**	**Sepsis ASIR per 100,000 population (95% UI), 2017**	**Incident sepsis cases (95% UI), 2017**	**Percentage change in sepsis ASIR (95% UI), 1990-2017**	**Sepsis ASIR per 100,000 population (95% UI), 2017**	**Incident sepsis cases (95% UI), 2017**	**Percentage change in sepsis ASIR (95% UI), 1990-2017**	**Sepsis ASIR per 100,000 population (95% UI), 2017**	**Incident sepsis cases (95% UI), 2017**	**Percentage change in sepsis ASIR (95% UI), 1990-2017**	**Sepsis ASIR per 100,000 population (95% UI), 2017**
Central Europe, eastern Europe, and central Asia	1,539,623 (1,282,185 - 1,872,799)	-31.4 (-51.7 - -8.3)	349.1 (283.6 - 439.2)	771,325 (557,899 - 1,079,309)	-29.4 (-60.3 - 12.5)	200.4 (141.1 - 283.2)	687,816 (563,632 - 843,088)	-32.0 (-51.0 - -6.2)	130.8 (105.7 - 164.8)	80,483 (58,256 - 111,444)	-31.6 (-57.1 - 0.9)	17.9 (13.0 - 24.5)
Central Asia	418,614 (321,644 - 557,943)	-40.4 (-61.7 - -14.8)	491.1 (382.8 - 645.9)	272,448 (184,615 - 398,685)	-46.1 (-71.8 - -11.0)	306.1 (207.9 - 445.0)	133,093 (103,074 - 175,738)	-22.5 (-45.7 - 10.1)	170.6 (134.6 - 223.7)	13,073 (9,605 - 17,604)	-41.9 (-62.0 - -16.2)	14.4 (10.6 - 19.3)
Armenia	10,777 (8,628 - 13,629)	-51.3 (-68.3 - -26.7)	346.3 (267.3 - 468.6)	5,663 (3,883 - 8,342)	-53.6 (-76.7 - -16.6)	205.3 (135.5 - 324.3)	4,819 (3,829 - 6,282)	-39.1 (-61.1 - -13.3)	131.8 (104.0 - 171.6)	295 (221 - 395)	-75.7 (-84.7 - -63.4)	9.2 (6.9 - 12.5)
Azerbaijan	51,825 (37,112 - 75,031)	-38.0 (-64.8 - -1.8)	624.6 (441.2 - 913.9)	34,544 (21,490 - 55,090)	-45.6 (-74.3 - 0.7)	417.7 (256.3 - 675.7)	16,224 (11,826 - 23,017)	-2.3 (-37.9 - 53.4)	196.1 (143.0 - 283.1)	1,057 (787 - 1,450)	-57.3 (-71.8 - -35.4)	10.8 (8.0 - 14.9)
Georgia	12,705 (10,806 - 15,212)	-50.1 (-67.9 - -26.9)	294.5 (242.5 - 363.2)	4,960 (3,668 - 6,841)	-61.2 (-80.5 - -33.7)	137.0 (95.8 - 203.2)	7,014 (5,787 - 8,523)	-31.6 (-56.9 - -0.4)	139.1 (113.6 - 171.2)	730 (534 - 983)	-9.7 (-46.4 - 38.5)	18.3 (13.3 - 25.1)
Kazakhstan	56,695 (45,561 - 71,584)	-49.9 (-66.8 - -30.3)	330.4 (263.6 - 415.4)	28,942 (20,208 - 42,758)	-57.8 (-77.4 - -28.5)	165.0 (114.6 - 245.3)	24,139 (19,068 - 30,739)	-36.3 (-56.6 - -11.5)	145.6 (115.4 - 186.2)	3,615 (2,545 - 5,046)	-31.9 (-58.6 - 3.0)	19.7 (13.9 - 27.5)
Kyrgyzstan	24,521 (19,112 - 32,077)	-56.7 (-71.0 - -39.4)	398.4 (318.5 - 505.8)	13,360 (9,453 - 18,921)	-65.7 (-81.4 - -44.6)	202.7 (146.9 - 280.5)	10,301 (7,224 - 15,987)	-36.5 (-58.8 - -3.2)	182.0 (135.0 - 263.6)	859 (636 - 1,158)	-52.3 (-69.1 - -31.0)	13.7 (10.1 - 18.4)
Mongolia	13,468 (10,743 - 17,376)	-57.7 (-70.7 - -43.1)	451.5 (369.5 - 563.9)	7,073 (4,956 - 10,448)	-66.0 (-80.3 - -45.5)	215.9 (156.7 - 309.4)	5,594 (4,257 - 7,831)	-45.5 (-62.1 - -24.3)	211.8 (168.8 - 276.1)	801 (573 - 1,093)	-24.1 (-52.0 - 13.2)	23.8 (17.1 - 32.3)
Tajikistan	83,593 (53,162 - 133,671)	-35.4 (-63.7 - 6.3)	778.9 (534.2 - 1,178.6)	65,570 (38,358 - 114,535)	-37.9 (-71.0 - 17.8)	569.1 (349.2 - 961.8)	16,919 (10,714 - 28,192)	-21.3 (-51.8 - 23.8)	198.2 (146.1 - 288.0)	1,104 (804 - 1,523)	-39.9 (-62.2 - -9.7)	11.6 (8.5 - 15.9)
Turkmenistan	22,468 (16,486 - 32,264)	-50.5 (-68.4 - -25.8)	470.9 (353.8 - 661.3)	14,246 (9,283 - 22,398)	-58.0 (-78.7 - -28.8)	281.9 (186.7 - 437.9)	7,649 (5,691 - 10,702)	-27.8 (-51.0 - 5.2)	177.6 (135.8 - 239.3)	573 (415 - 793)	-46.0 (-66.2 - -19.3)	11.4 (8.3 - 15.7)
Uzbekistan	142,563 (105,118 - 198,513)	-35.1 (-60.0 - -3.1)	493.9 (372.8 - 679.3)	98,090 (64,335 - 151,652)	-42.4 (-70.0 - -0.7)	313.8 (207.3 - 481.8)	40,434 (31,306 - 54,423)	-10.9 (-39.1 - 26.5)	167.4 (132.1 - 221.0)	4,039 (2,882 - 5,750)	-36.3 (-59.9 - -2.8)	12.6 (9.0 - 17.9)
Central Europe	317,902 (266,895 - 389,351)	-49.1 (-62.5 - -33.2)	197.2 (162.6 - 246.4)	126,904 (88,944 - 187,950)	-49.8 (-70.9 - -17.4)	90.6 (62.3 - 135.2)	175,990 (144,556 - 217,637)	-47.0 (-62.5 - -27.7)	95.9 (77.9 - 120.7)	15,008 (10,526 - 21,328)	-49.4 (-69.7 - -21.3)	10.7 (7.6 - 15.2)
Albania	5,278 (4,161 - 6,892)	-65.1 (-78.4 - -47.1)	186.2 (140.4 - 261.4)	2,175 (1,436 - 3,468)	-73.6 (-87.3 - -50.5)	91.9 (57.5 - 154.2)	2,843 (2,172 - 3,761)	-47.5 (-66.2 - -23.1)	85.0 (63.7 - 113.7)	260 (177 - 359)	-34.5 (-59.6 - -3.3)	9.3 (6.3 - 12.7)
Bosnia and Herzegovina	7,569 (6,278 - 9,442)	-39.0 (-54.9 - -20.7)	164.4 (136.8 - 201.7)	1,694 (1,280 - 2,294)	-41.4 (-62.7 - -11.0)	50.6 (36.8 - 71.2)	5,534 (4,321 - 7,341)	-37.1 (-58.5 - -13.4)	105.1 (82.3 - 134.9)	341 (239 - 491)	-35.6 (-61.0 - -0.2)	8.7 (6.2 - 12.3)
Bulgaria	21,325 (17,887 - 26,205)	-39.6 (-57.4 - -17.6)	220.9 (179.3 - 276.6)	7,445 (5,320 - 10,769)	-46.6 (-71.2 - -11.8)	101.7 (69.8 - 150.7)	13,005 (10,501 - 16,581)	-28.8 (-51.0 - -1.8)	108.1 (86.5 - 140.1)	874 (622 - 1,212)	-36.5 (-61.2 - -3.5)	11.2 (7.9 - 15.5)
Croatia	11,792 (9,761 - 14,621)	-29.9 (-48.0 - -6.4)	172.5 (140.3 - 216.2)	4,690 (3,124 - 6,949)	-7.8 (-48.7 - 47.4)	79.6 (53.0 - 121.4)	6,501 (5,278 - 8,199)	-40.1 (-58.0 - -17.3)	83.1 (67.2 - 102.9)	600 (390 - 954)	-45.8 (-68.7 - -13.7)	9.8 (6.7 - 14.2)

Czech Republic	30,736 (24,553 - 39,381)	-38.1 (-55.2 - -18.5)	178.0 (143.6 - 225.8)	15,028 (9,869 - 22,414)	-8.6 (-47.4 - 47.7)	92.4 (62.7 - 135.6)	14,346 (11,430 - 18,306)	-53.1 (-67.5 - -35.3)	75.6 (60.7 - 95.4)	1,362 (934 - 1,935)	-51.7 (-74.1 - -20.9)	10.0 (6.9 - 14.0)
Hungary	26,575 (22,309 - 32,454)	-48.4 (-61.0 - -34.2)	172.3 (143.6 - 210.7)	8,663 (6,221 - 12,388)	-35.6 (-59.9 - -2.5)	65.4 (46.5 - 92.2)	16,639 (13,349 - 20,981)	-52.0 (-66.0 - -33.5)	97.6 (79.0 - 121.6)	1,273 (840 - 1,916)	-63.7 (-79.9 - -40.3)	9.3 (6.4 - 13.3)
Montenegro	1,226 (1,004 - 1,539)	-36.8 (-56.7 - -13.2)	155.2 (126.9 - 192.1)	291 (202 - 441)	-43.6 (-70.5 - -0.2)	45.9 (31.0 - 70.0)	864 (678 - 1,127)	-31.2 (-56.3 - -0.1)	99.1 (77.3 - 129.0)	71 (51 - 96)	-35.6 (-58.1 - -5.9)	10.2 (7.3 - 13.7)
North Macedonia	4,135 (3,382 - 5,136)	-64.1 (-79.6 - -42.7)	180.0 (140.9 - 241.8)	1,039 (737 - 1,453)	-79.7 (-91.4 - -60.9)	60.5 (41.0 - 88.8)	2,930 (2,261 - 3,791)	-36.4 (-63.7 - 3.4)	112.4 (81.2 - 166.9)	166 (116 - 235)	-31.5 (-59.1 - 5.2)	7.1 (5.0 - 10.2)
Poland	100,478 (82,825 - 126,495)	-47.4 (-61.5 - -29.1)	181.3 (147.3 - 229.7)	41,790 (28,394 - 64,322)	-44.2 (-70.1 - -5.5)	80.4 (54.1 - 120.8)	53,365 (43,119 - 67,116)	-48.3 (-64.9 - -28.5)	89.6 (70.9 - 116.9)	5,323 (3,646 - 7,673)	-48.3 (-70.4 - -18.6)	11.3 (7.8 - 16.3)
Romania	65,281 (53,229 - 82,686)	-51.4 (-68.3 - -30.3)	270.0 (207.9 - 363.5)	29,071 (19,530 - 44,905)	-51.0 (-75.4 - -10.9)	148.6 (94.5 - 244.1)	33,446 (27,090 - 41,591)	-49.8 (-67.5 - -29.4)	108.7 (87.3 - 136.2)	2,764 (1,999 - 3,798)	-48.0 (-68.3 - -19.7)	12.6 (9.1 - 17.5)
Serbia	24,146 (20,119 - 29,858)	-45.5 (-60.5 - -27.8)	186.1 (154.6 - 226.8)	6,494 (4,748 - 9,183)	-53.8 (-73.1 - -25.8)	57.9 (42.5 - 82.1)	16,750 (13,448 - 21,570)	-38.1 (-59.8 - -10.7)	120.0 (95.3 - 154.0)	902 (647 - 1,235)	-50.3 (-69.4 - -25.9)	8.2 (5.8 - 11.3)
Slovakia	14,461 (11,680 - 18,244)	-37.8 (-56.4 - -14.9)	201.9 (161.5 - 261.3)	6,572 (4,434 - 10,134)	-31.9 (-64.3 - 16.4)	96.7 (65.1 - 147.9)	7,149 (5,799 - 8,933)	-39.4 (-59.0 - -13.0)	93.6 (73.9 - 123.3)	741 (509 - 1,087)	-47.4 (-69.2 - -14.1)	11.6 (8.1 - 16.5)
Slovenia	4,900 (3,984 - 6,327)	-44.8 (-59.3 - -26.4)	134.9 (109.8 - 169.9)	1,951 (1,283 - 3,154)	-33.2 (-63.3 - 9.1)	58.1 (38.4 - 88.1)	2,617 (2,044 - 3,415)	-49.7 (-65.6 - -29.3)	65.8 (51.7 - 84.4)	332 (207 - 517)	-52.0 (-74.9 - -21.5)	11.0 (7.1 - 16.2)
Eastern Europe	803,107 (672,469 - 958,729)	-17.9 (-38.6 - 6.7)	320.6 (263.0 - 394.8)	371,973 (273,043 - 512,316)	-1.9 (-40.7 - 48.4)	170.6 (123.7 - 244.6)	378,733 (307,431 - 473,313)	-30.6 (-49.7 - -5.3)	127.1 (102.9 - 158.7)	52,402 (37,806 - 72,197)	-20.4 (-51.1 - 20.0)	22.9 (16.5 - 31.3)
Belarus	26,034 (21,460 - 31,897)	-34.2 (-51.4 - -12.2)	218.4 (175.1 - 273.0)	9,332 (6,616 - 13,216)	-34.6 (-63.2 - 4.1)	93.2 (62.7 - 135.8)	14,835 (11,730 - 19,220)	-32.2 (-52.9 - -4.9)	108.4 (85.4 - 139.1)	1,866 (1,300 - 2,683)	-32.8 (-60.8 - 8.6)	16.7 (11.8 - 24.0)
Estonia	3,308 (2,667 - 4,111)	-51.3 (-64.4 - -34.9)	164.8 (132.7 - 203.9)	1,388 (948 - 2,039)	-47.0 (-68.1 - -11.8)	78.2 (54.2 - 112.9)	1,740 (1,366 - 2,231)	-50.3 (-66.0 - -31.0)	75.0 (59.2 - 95.1)	180 (122 - 261)	-66.5 (-80.6 - -44.7)	11.6 (7.9 - 16.5)
Latvia	6,586 (5,374 - 8,098)	-32.6 (-48.4 - -13.4)	220.3 (179.6 - 270.8)	2,635 (1,859 - 3,792)	-18.5 (-49.2 - 26.1)	101.9 (71.9 - 147.2)	3,565 (2,791 - 4,617)	-36.7 (-55.1 - -12.6)	102.1 (80.6 - 129.6)	386 (266 - 548)	-55.6 (-74.0 - -31.0)	16.4 (11.4 - 22.9)
Lithuania	9,847 (8,111 - 11,954)	-16.3 (-36.9 - 9.4)	234.2 (190.8 - 287.3)	4,196 (3,041 - 5,931)	15.9 (-29.0 - 77.2)	117.2 (82.9 - 163.8)	5,024 (4,046 - 6,343)	-31.8 (-52.2 - -5.9)	99.5 (80.0 - 125.6)	627 (428 - 921)	-40.2 (-65.7 - -1.7)	17.5 (12.1 - 25.8)
Moldova	15,055 (11,994 - 19,167)	-30.1 (-53.9 - 8.8)	432.6 (293.5 - 626.3)	8,094 (5,195 - 12,055)	-20.9 (-59.8 - 44.9)	281.6 (140.8 - 477.3)	6,292 (5,116 - 7,729)	-39.9 (-57.2 - -18.0)	134.8 (107.8 - 172.1)	669 (492 - 931)	-49.6 (-67.8 - -22.1)	16.3 (11.9 - 22.7)
Russia	573,487 (481,452 - 682,786)	-17.1 (-38.1 - 6.9)	328.8 (270.2 - 405.2)	273,831 (199,975 - 380,012)	0.1 (-39.2 - 52.0)	177.4 (127.7 - 256.2)	262,312 (212,284 - 330,505)	-30.7 (-49.5 - -5.1)	128.2 (103.6 - 160.0)	37,343 (27,095 - 51,698)	-22.8 (-52.2 - 15.6)	23.2 (16.6 - 31.9)
Ukraine	168,791 (140,054 - 202,794)	-13.5 (-36.8 - 17.2)	324.6 (264.8 - 411.5)	72,496 (52,915 - 97,560)	4.0 (-39.7 - 69.0)	166.5 (117.7 - 248.6)	84,964 (67,745 - 107,414)	-28.1 (-49.6 - -0.1)	133.1 (106.5 - 167.2)	11,331 (8,043 - 15,418)	1.1 (-40.0 - 55.5)	25.1 (17.7 - 34.4)
High-income	3,405,288 (2,762,325 - 4,376,855)	-17.8 (-39.5 - 8.1)	204.1 (165.7 - 256.7)	1,885,892 (1,306,086 - 2,790,861)	-3.1 (-42.2 - 46.4)	115.0 (81.6 - 164.9)	1,399,475 (1,128,469 - 1,747,081)	-27.8 (-50.7 - 2.2)	80.0 (62.8 - 103.8)	119,922 (82,808 - 172,469)	-42.2 (-67.4 - -10.9)	9.2 (6.4 - 13.2)
Australasia	65,390 (52,787 - 82,558)	-22.0 (-44.4 - 5.2)	159.7 (127.1 - 214.0)	33,950 (22,965 - 49,219)	-3.6 (-44.4 - 51.9)	85.3 (58.1 - 134.6)	28,667 (22,510 - 36,459)	-32.7 (-54.2 - -4.0)	66.2 (50.7 - 85.9)	2,773 (1,814 - 4,149)	-44.3 (-71.6 - -6.5)	8.3 (5.5 - 12.5)
Australia	55,251 (44,428 - 70,778)	-21.3 (-44.3 - 7.8)	158.8 (125.3 - 215.0)	28,863 (19,468 - 43,211)	-0.1 (-44.2 - 59.8)	85.2 (57.6 - 140.4)	24,084 (18,821 - 30,776)	-33.9 (-55.5 - -5.7)	65.6 (50.2 - 85.4)	2,304 (1,505 - 3,497)	-42.8 (-70.9 - -3.7)	8.0 (5.3 - 12.1)
New Zealand	10,140 (8,247 - 12,731)	-25.7 (-47.9 - 0.2)	164.2 (131.4 - 210.9)	5,088 (3,422 - 7,351)	-17.6 (-55.7 - 31.0)	85.1 (56.9 - 123.4)	4,583 (3,617 - 5,834)	-27.0 (-50.0 - 5.7)	69.3 (52.9 - 91.9)	469 (308 - 706)	-48.4 (-74.0 - -12.7)	9.8 (6.5 - 14.8)
High-income Asia Pacific	588,812 (451,988 - 827,629)	-33.7 (-53.8 - -7.3)	148.3 (116.4 - 200.8)	359,233 (224,738 - 578,500)	-23.1 (-57.9 - 28.4)	91.6 (60.6 - 142.3)	212,554 (163,540 - 285,622)	-42.7 (-58.7 - -20.9)	50.0 (39.7 - 64.3)	17,025 (11,387 - 25,412)	-53.6 (-74.1 - -25.1)	6.7 (4.5 - 10.0)

Brunei	1,157 (924 - 1,485)	-7.2 (-30.9 - 24.9)	356.5 (284.4 - 460.9)	633 (452 - 909)	26.6 (-21.1 - 99.0)	203.0 (143.9 - 291.6)	462 (353 - 628)	-29.3 (-51.9 - 1.9)	138.6 (105.1 - 187.2)	62 (44 - 85)	-35.4 (-62.1 - -5.1)	15.0 (10.8 - 20.3)
Japan	470,031 (352,015 - 674,092)	-27.4 (-50.9 - 4.9)	143.6 (111.9 - 195.3)	295,847 (181,915 - 484,069)	-16.8 (-56.2 - 44.0)	91.3 (59.6 - 143.2)	163,064 (124,283 - 224,863)	-38.3 (-56.9 - -14.2)	46.3 (36.8 - 59.6)	11,120 (7,424 - 16,889)	-41.2 (-67.3 - -2.2)	6.0 (4.0 - 9.1)
South Korea	105,582 (84,630 - 139,010)	-48.4 (-61.5 - -29.3)	160.1 (127.5 - 218.2)	53,202 (35,629 - 80,675)	-32.6 (-60.1 - 11.8)	86.6 (58.1 - 143.0)	46,764 (35,601 - 61,921)	-57.5 (-69.6 - -41.0)	64.7 (49.7 - 86.5)	5,616 (3,793 - 8,493)	-66.7 (-81.1 - -47.0)	8.8 (6.0 - 13.2)
Singapore	12,041 (8,416 - 17,936)	-24.4 (-53.9 - 20.1)	191.9 (134.3 - 285.4)	9,551 (5,929 - 15,336)	2.7 (-48.6 - 85.4)	152.3 (94.9 - 243.7)	2,265 (1,810 - 2,937)	-61.7 (-73.0 - -46.3)	35.9 (28.5 - 46.5)	226 (148 - 349)	-53.5 (-73.9 - -24.3)	3.7 (2.4 - 5.7)
High-income North America	1,182,106 (963,659 - 1,473,480)	-0.4 (-28.9 - 32.3)	247.8 (198.4 - 311.1)	652,213 (456,632 - 921,637)	13.5 (-32.6 - 79.5)	138.3 (98.0 - 193.3)	480,673 (386,219 - 599,307)	-8.7 (-41.0 - 32.8)	97.2 (74.9 - 130.4)	49,221 (34,013 - 71,812)	-26.9 (-61.9 - 20.3)	12.3 (8.4 - 18.3)
Canada	98,916 (78,504 - 127,026)	-2.0 (-31.6 - 32.4)	183.8 (144.4 - 234.8)	54,760 (35,883 - 80,473)	32.6 (-26.7 - 114.6)	102.1 (69.2 - 147.7)	40,133 (32,111 - 50,680)	-22.1 (-48.2 - 14.2)	72.3 (55.3 - 96.7)	4,023 (2,678 - 5,980)	-35.3 (-66.4 - 5.3)	9.5 (6.4 - 14.0)
Greenland	162 (134 - 200)	-34.5 (-52.1 - -13.5)	300.2 (243.4 - 376.8)	72 (53 - 100)	-16.0 (-46.5 - 29.4)	138.2 (100.7 - 194.2)	77 (62 - 100)	-40.8 (-61.1 - -11.7)	138.9 (106.6 - 188.0)	13 (9 - 19)	-56.7 (-73.9 - -34.8)	23.1 (16.5 - 33.4)
USA	1,083,007 (884,243 - 1,342,025)	-0.1 (-28.9 - 32.5)	254.9 (204.8 - 318.9)	597,370 (418,706 - 850,648)	12.6 (-33.1 - 77.0)	142.3 (100.6 - 199.1)	440,454 (353,594 - 551,017)	-7.3 (-40.7 - 34.7)	100.0 (77.0 - 134.3)	45,184 (31,223 - 66,328)	-26.1 (-61.4 - 22.6)	12.7 (8.6 - 18.8)
Southern Latin America	257,999 (209,344 - 323,462)	-26.1 (-47.6 - -1.7)	359.4 (288.1 - 455.4)	157,499 (114,794 - 219,369)	-13.3 (-48.7 - 32.4)	221.6 (160.8 - 310.5)	91,249 (72,467 - 114,876)	-39.0 (-59.0 - -13.9)	124.5 (96.9 - 161.0)	9,251 (6,708 - 12,738)	-35.3 (-58.7 - -5.4)	13.4 (9.6 - 18.5)
Argentina	191,581 (152,645 - 244,495)	-21.8 (-46.7 - 6.7)	400.5 (317.0 - 511.8)	120,419 (86,749 - 167,282)	-5.5 (-45.6 - 51.4)	252.9 (180.5 - 352.3)	64,573 (50,370 - 83,407)	-38.5 (-59.4 - -13.2)	133.4 (101.4 - 175.6)	6,589 (4,770 - 9,243)	-29.5 (-55.7 - 3.2)	14.3 (10.4 - 20.0)
Chile	51,711 (41,802 - 64,332)	-43.9 (-59.2 - -24.7)	260.8 (207.0 - 339.5)	28,432 (20,238 - 39,492)	-42.9 (-65.5 - -12.1)	146.7 (102.9 - 214.9)	21,210 (16,778 - 26,550)	-43.1 (-60.8 - -20.2)	103.4 (80.9 - 134.7)	2,070 (1,471 - 2,888)	-51.9 (-71.2 - -26.5)	10.7 (7.6 - 15.0)
Uruguay	14,696 (11,812 - 18,359)	-14.1 (-39.3 - 16.8)	335.6 (264.7 - 433.1)	8,642 (6,252 - 12,083)	7.2 (-36.0 - 65.8)	206.3 (145.0 - 298.3)	5,461 (4,409 - 6,778)	-34.9 (-54.8 - -9.1)	114.0 (88.9 - 144.3)	593 (428 - 804)	-17.4 (-50.0 - 23.7)	15.3 (11.0 - 21.1)
Western Europe	1,310,922 (1,069,548 - 1,682,660)	-25.3 (-44.6 - -2.6)	167.8 (137.5 - 210.2)	682,920 (466,754 - 1,030,675)	-7.8 (-44.7 - 42.1)	89.3 (62.8 - 128.5)	586,357 (471,753 - 745,327)	-34.9 (-54.5 - -9.5)	71.7 (56.6 - 90.3)	41,645 (27,548 - 62,125)	-54.5 (-75.3 - -26.2)	6.8 (4.7 - 9.8)
Andorra	197 (148 - 267)	-20.2 (-45.6 - 14.3)	157.5 (121.6 - 208.7)	114 (71 - 181)	-9.5 (-52.1 - 53.4)	88.2 (59.3 - 133.8)	78 (61 - 101)	-26.7 (-51.6 - 3.3)	63.0 (49.2 - 81.2)	6 (4 - 10)	-39.6 (-68.0 - 2.9)	6.4 (4.2 - 9.3)
Austria	17,764 (14,990 - 21,748)	-38.5 (-54.4 - -20.0)	121.3 (100.9 - 149.0)	5,841 (4,019 - 8,487)	-31.3 (-60.0 - 8.2)	44.4 (30.4 - 63.8)	10,962 (8,786 - 13,706)	-39.2 (-57.6 - -14.9)	69.0 (54.1 - 88.0)	961 (613 - 1,473)	-52.0 (-75.5 - -20.9)	7.9 (5.2 - 11.7)
Belgium	40,952 (31,938 - 54,451)	-13.2 (-38.3 - 14.9)	195.1 (153.8 - 253.7)	24,762 (16,023 - 37,055)	27.8 (-30.1 - 108.9)	116.1 (77.8 - 173.8)	14,687 (11,744 - 19,003)	-38.7 (-58.2 - -15.7)	69.5 (55.7 - 87.6)	1,504 (992 - 2,266)	-44.1 (-70.1 - -5.5)	9.5 (6.4 - 14.0)
Cyprus	2,597 (2,009 - 3,347)	-35.9 (-54.0 - -13.7)	152.3 (118.9 - 195.6)	1,301 (823 - 1,983)	-3.4 (-44.4 - 55.5)	76.3 (49.6 - 113.9)	1,174 (913 - 1,581)	-51.1 (-67.4 - -29.4)	67.5 (52.1 - 89.1)	122 (83 - 179)	-51.3 (-73.1 - -22.0)	8.5 (5.8 - 12.6)
Denmark	18,999 (14,727 - 24,746)	-9.7 (-35.5 - 19.7)	187.9 (146.2 - 245.2)	10,141 (6,322 - 15,468)	17.0 (-34.7 - 95.6)	95.8 (62.1 - 144.6)	8,365 (6,643 - 10,899)	-20.8 (-45.3 - 11.1)	85.9 (66.5 - 112.9)	493 (324 - 734)	-58.6 (-77.9 - -32.8)	6.2 (4.2 - 8.9)
Finland	12,580 (10,173 - 15,929)	-46.7 (-60.4 - -30.3)	124.1 (101.4 - 154.2)	4,443 (3,006 - 6,508)	-57.6 (-76.5 - -30.0)	46.7 (31.2 - 68.0)	7,455 (5,752 - 9,910)	-31.6 (-53.5 - -5.1)	68.8 (53.4 - 89.0)	683 (423 - 1,137)	-52.8 (-74.2 - -20.7)	8.6 (5.6 - 13.0)
France	176,908 (146,143 - 221,269)	-34.0 (-50.9 - -9.2)	154.5 (125.3 - 202.3)	87,728 (60,866 - 129,591)	-25.5 (-56.1 - 24.2)	81.8 (55.6 - 128.0)	80,706 (64,637 - 102,294)	-36.4 (-53.7 - -14.3)	64.0 (51.3 - 79.9)	8,474 (5,510 - 12,769)	-57.4 (-76.6 - -30.1)	8.7 (5.9 - 12.8)
Germany	278,978 (223,434 - 361,735)	-22.2 (-43.5 - 5.4)	175.9 (139.2 - 224.7)	136,622 (90,890 - 205,699)	-6.4 (-47.7 - 49.2)	87.4 (59.7 - 134.2)	133,680 (106,173 - 168,518)	-29.4 (-51.4 - -1.7)	81.4 (63.2 - 105.3)	8,676 (5,550 - 13,531)	-49.8 (-74.5 - -18.5)	7.0 (4.6 - 10.3)

Greece	28,023 (22,342 - 36,414)	-25.0 (-49.4 - 2.3)	140.2 (111.9 - 180.9)	12,340 (7,809 - 19,736)	-7.2 (-48.7 - 60.8)	61.4 (40.2 - 93.7)	14,607 (11,692 - 18,855)	-31.2 (-58.2 - 2.8)	70.3 (54.8 - 92.9)	1,076 (710 - 1,531)	-42.8 (-71.4 - -4.3)	8.5 (5.7 - 12.7)
Iceland	646 (504 - 870)	-34.3 (-55.6 - -5.9)	134.0 (104.0 - 179.0)	350 (218 - 556)	-23.8 (-62.6 - 32.8)	74.6 (47.0 - 116.8)	267 (212 - 346)	-40.8 (-60.7 - -17.7)	52.0 (41.4 - 65.9)	30 (20 - 44)	-47.2 (-71.4 - -11.4)	7.5 (5.1 - 11.0)
Ireland	10,202 (8,209 - 13,508)	-31.6 (-51.9 - -6.0)	157.0 (127.3 - 203.0)	5,520 (3,651 - 8,387)	-19.4 (-57.4 - 35.5)	84.4 (56.9 - 124.9)	4,393 (3,483 - 5,482)	-38.0 (-58.1 - -14.5)	67.3 (52.6 - 86.1)	290 (189 - 438)	-57.0 (-77.0 - -28.8)	5.3 (3.5 - 7.9)
Israel	21,030 (16,805 - 27,015)	-23.9 (-46.3 - 2.9)	189.0 (151.8 - 239.5)	12,641 (8,610 - 17,955)	3.2 (-42.4 - 64.4)	112.8 (77.7 - 159.8)	7,691 (6,106 - 9,731)	-38.7 (-58.7 - -14.5)	68.9 (55.2 - 86.4)	698 (481 - 969)	-67.5 (-80.9 - -48.9)	7.3 (5.0 - 10.0)
Italy	150,140 (123,456 - 189,417)	-36.9 (-55.9 - -13.8)	129.0 (104.7 - 163.7)	61,977 (43,614 - 91,224)	-12.1 (-48.7 - 43.2)	62.2 (42.5 - 91.5)	82,876 (64,788 - 108,785)	-48.3 (-66.9 - -24.9)	61.1 (48.0 - 78.6)	5,288 (3,571 - 7,746)	-55.0 (-76.5 - -28.4)	5.7 (3.9 - 8.2)
Luxembourg	1,362 (1,084 - 1,779)	-30.1 (-48.9 - -6.5)	156.3 (125.8 - 202.7)	665 (441 - 1,008)	-10.6 (-48.9 - 48.3)	79.6 (52.5 - 122.0)	639 (505 - 825)	-39.6 (-56.4 - -16.9)	68.8 (54.5 - 87.3)	59 (38 - 90)	-56.1 (-77.3 - -28.5)	7.9 (5.2 - 11.7)
Malta	1,349 (1,074 - 1,773)	-11.1 (-38.1 - 20.9)	194.4 (153.7 - 257.0)	748 (507 - 1,119)	25.2 (-27.4 - 102.6)	104.8 (71.3 - 152.8)	565 (450 - 711)	-32.2 (-57.7 - 3.0)	83.2 (63.0 - 116.6)	36 (24 - 53)	-31.3 (-60.8 - 10.9)	6.3 (4.3 - 9.1)
Netherlands	58,707 (46,160 - 77,794)	-7.1 (-35.4 - 31.9)	215.3 (163.5 - 289.8)	35,208 (23,315 - 53,354)	22.1 (-32.7 - 112.3)	135.2 (86.6 - 206.7)	21,970 (17,525 - 28,479)	-31.0 (-52.7 - -3.7)	74.0 (58.2 - 94.5)	1,529 (957 - 2,555)	-41.9 (-68.8 - -0.0)	6.1 (4.1 - 9.5)
Norway	15,500 (11,959 - 20,648)	-24.8 (-46.6 - 2.6)	168.8 (133.9 - 216.5)	9,480 (6,110 - 14,377)	-5.2 (-47.5 - 59.6)	99.9 (67.1 - 145.0)	5,512 (4,324 - 7,146)	-36.0 (-55.2 - -12.0)	61.6 (48.8 - 78.2)	509 (331 - 807)	-61.3 (-79.6 - -34.9)	7.3 (4.9 - 10.8)
Portugal	41,681 (33,038 - 55,205)	-35.0 (-52.8 - -11.0)	201.2 (159.2 - 260.8)	23,915 (15,797 - 37,314)	-6.5 (-46.4 - 48.3)	118.7 (80.7 - 177.2)	16,726 (13,321 - 21,243)	-51.9 (-66.5 - -34.0)	75.1 (60.0 - 94.0)	1,040 (737 - 1,424)	-66.9 (-80.7 - -49.2)	7.4 (5.3 - 10.1)
Spain	137,938 (111,276 - 178,469)	-31.3 (-49.9 - -7.3)	162.2 (130.2 - 214.6)	73,359 (50,575 - 107,696)	-10.3 (-47.0 - 40.1)	93.2 (64.1 - 143.8)	61,271 (47,420 - 82,841)	-43.7 (-61.4 - -21.4)	63.7 (50.3 - 82.6)	3,309 (2,177 - 4,889)	-66.6 (-82.9 - -46.3)	5.3 (3.6 - 7.6)
Sweden	28,476 (22,242 - 37,586)	-22.3 (-44.7 - 6.2)	149.1 (116.6 - 191.6)	16,045 (10,315 - 24,492)	-9.1 (-50.0 - 48.8)	84.4 (55.9 - 123.6)	11,491 (9,158 - 14,775)	-31.1 (-51.5 - -4.2)	58.4 (47.1 - 73.2)	940 (612 - 1,446)	-44.1 (-70.2 - -8.5)	6.3 (4.2 - 9.3)
Switzerland	19,749 (15,789 - 25,171)	-30.8 (-52.3 - -1.2)	144.5 (110.4 - 192.9)	10,466 (7,005 - 15,429)	-24.1 (-60.0 - 28.6)	80.7 (50.1 - 124.6)	8,510 (6,717 - 11,025)	-30.0 (-53.6 - 2.1)	57.7 (44.5 - 77.1)	773 (464 - 1,344)	-63.2 (-80.7 - -36.6)	6.1 (4.0 - 9.6)
United Kingdom	245,783 (191,983 - 330,996)	-2.2 (-32.3 - 36.1)	216.2 (173.2 - 280.5)	148,548 (96,687 - 227,079)	22.3 (-34.9 - 105.2)	124.4 (83.3 - 184.5)	92,125 (73,299 - 117,720)	-18.5 (-44.4 - 16.6)	86.0 (66.1 - 114.0)	5,109 (3,327 - 7,674)	-43.4 (-68.1 - -10.4)	5.8 (3.9 - 8.5)
Latin America and Caribbean	2,479,512 (2,022,964 - 3,082,183)	-44.7 (-63.6 - -23.5)	454.9 (366.2 - 571.0)	1,512,060 (1,093,850 - 2,082,708)	-45.1 (-70.6 - -11.0)	282.5 (202.0 - 393.6)	863,259 (694,343 - 1,065,868)	-41.5 (-57.5 - -20.3)	154.4 (124.3 - 191.9)	104,194 (79,923 - 136,243)	-45.5 (-61.5 - -24.2)	17.9 (13.8 - 23.3)
Andean Latin America	300,256 (229,721 - 394,106)	-51.4 (-67.1 - -31.7)	496.8 (383.8 - 649.1)	197,233 (133,843 - 292,440)	-48.1 (-70.6 - -16.9)	321.6 (221.3 - 472.4)	92,220 (71,489 - 119,201)	-53.5 (-69.7 - -34.8)	157.4 (123.5 - 200.4)	10,804 (7,849 - 15,031)	-64.4 (-77.3 - -46.7)	17.7 (12.9 - 24.6)
Bolivia	63,172 (47,765 - 84,705)	-57.8 (-71.6 - -41.3)	582.6 (450.2 - 758.0)	34,999 (23,989 - 52,984)	-58.7 (-76.1 - -34.3)	306.7 (217.9 - 442.9)	25,866 (18,506 - 35,732)	-54.8 (-74.4 - -31.7)	254.8 (188.1 - 338.8)	2,307 (1,586 - 3,341)	-58.2 (-74.1 - -38.0)	21.0 (14.8 - 29.9)
Ecuador	65,078 (51,793 - 83,704)	-41.5 (-60.4 - -17.6)	420.2 (336.4 - 538.7)	36,285 (25,024 - 52,484)	-45.4 (-70.3 - -9.7)	233.6 (161.4 - 338.5)	25,321 (19,819 - 32,749)	-34.6 (-54.2 - -9.8)	165.5 (130.6 - 212.8)	3,472 (2,547 - 4,708)	-22.9 (-47.7 - 10.9)	21.1 (15.5 - 28.7)
Peru	172,007 (123,848 - 243,388)	-51.5 (-69.0 - -27.6)	509.7 (370.2 - 716.6)	125,949 (80,892 - 194,313)	-44.2 (-70.8 - -5.9)	369.6 (240.1 - 565.5)	41,033 (31,197 - 54,539)	-60.3 (-74.4 - -42.7)	125.0 (95.6 - 164.5)	5,025 (3,503 - 7,306)	-74.7 (-85.2 - -60.8)	15.1 (10.6 - 21.8)
Caribbean	280,526 (219,270 - 365,898)	-20.0 (-48.4 - 15.7)	639.4 (488.6 - 847.3)	173,384 (120,257 - 254,406)	-15.3 (-54.0 - 45.3)	410.6 (280.1 - 613.5)	95,929 (75,281 - 129,282)	-24.6 (-49.3 - 8.1)	204.7 (159.3 - 279.3)	11,213 (8,561 - 14,658)	-24.7 (-47.8 - 4.9)	24.1 (18.4 - 31.5)
Antigua and Barbuda	327 (262 - 414)	-3.5 (-32.1 - 29.9)	392.7 (305.2 - 516.1)	188 (134 - 263)	13.3 (-32.5 - 78.8)	235.7 (163.4 - 341.5)	128 (100 - 166)	-19.4 (-44.5 - 12.9)	144.1 (111.1 - 191.6)	11 (9 - 15)	-19.3 (-46.2 - 15.4)	12.9 (9.8 - 17.2)
The Bahamas	1,454 (1,185 - 1,809)	-12.4 (-37.6 - 21.3)	440.1 (352.5 - 575.5)	840 (606 - 1,184)	-2.4 (-39.9 - 53.9)	268.0 (188.0 - 395.2)	548 (436 - 688)	-22.9 (-45.5 - 6.8)	154.2 (122.8 - 195.7)	67 (49 - 90)	-20.1 (-49.0 - 16.3)	17.9 (13.0 - 24.4)

Appendix: Global Burden of Sepsis

Barbados	1,669 (1,342 - 2,138)	9.5 (-23.5 - 54.9)	530.4 (399.5 - 711.9)	1,084 (783 - 1,500)	33.9 (-18.6 - 118.6)	368.1 (250.6 - 546.9)	551 (432 - 729)	-20.1 (-45.9 - 15.1)	151.9 (116.2 - 203.4)	34 (25 - 47)	-26.4 (-53.9 - 8.0)	10.3 (7.4 - 14.3)
Belize	1,581 (1,254 - 2,051)	-20.4 (-43.0 - 7.5)	473.1 (381.4 - 605.4)	937 (660 - 1,393)	-22.4 (-53.4 - 19.8)	272.9 (196.4 - 397.3)	556 (444 - 709)	-14.9 (-38.1 - 14.5)	176.4 (143.5 - 218.8)	88 (67 - 116)	-17.5 (-44.0 - 16.3)	23.8 (18.3 - 31.1)
Bermuda	145 (118 - 184)	-39.2 (-54.9 - -19.4)	158.3 (126.8 - 201.8)	69 (48 - 100)	-25.9 (-55.1 - 14.3)	84.2 (59.0 - 121.9)	72 (57 - 92)	-48.5 (-62.4 - -31.6)	67.8 (54.3 - 85.4)	5 (4 - 7)	-50.7 (-69.4 - -24.1)	6.3 (4.4 - 8.8)
Cuba	35,780 (29,242 - 45,496)	-32.5 (-51.7 - -8.2)	249.8 (200.1 - 320.6)	18,912 (13,326 - 27,885)	-29.9 (-59.3 - 13.3)	144.1 (99.3 - 211.9)	15,137 (12,333 - 18,558)	-31.7 (-51.7 - -10.3)	93.8 (76.7 - 115.6)	1,731 (1,225 - 2,531)	-47.7 (-66.5 - -20.3)	11.9 (8.5 - 17.0)
Dominica	449 (351 - 602)	52.7 (1.6 - 140.5)	747.8 (537.7 - 1,101.1)	253 (172 - 391)	78.7 (-3.8 - 220.4)	464.5 (289.8 - 798.1)	184 (144 - 245)	28.9 (-14.4 - 91.4)	265.1 (198.3 - 366.6)	13 (9 - 17)	-1.0 (-34.2 - 42.2)	18.2 (13.5 - 24.3)
Dominican Republic	71,859 (49,368 - 112,131)	-10.0 (-49.4 - 50.6)	715.2 (493.1 - 1,111.5)	49,491 (29,765 - 87,546)	-2.6 (-57.2 - 95.8)	492.6 (296.6 - 875.4)	19,947 (14,378 - 28,543)	-19.9 (-48.2 - 21.0)	199.4 (145.4 - 284.1)	2,421 (1,620 - 3,714)	-11.4 (-46.5 - 39.9)	23.2 (15.7 - 35.3)
Grenada	543 (451 - 679)	-16.1 (-38.5 - 12.3)	431.4 (346.6 - 562.7)	281 (207 - 394)	-1.9 (-40.2 - 49.2)	232.6 (162.8 - 347.2)	244 (198 - 307)	-26.3 (-45.6 - -1.9)	183.8 (147.3 - 234.4)	19 (14 - 24)	-37.1 (-56.9 - -10.7)	15.0 (11.3 - 19.5)
Guyana	4,267 (3,322 - 5,618)	-24.0 (-45.7 - 7.3)	628.6 (493.0 - 820.8)	2,217 (1,502 - 3,398)	-20.0 (-55.4 - 36.5)	323.4 (223.7 - 487.9)	1,856 (1,434 - 2,518)	-26.6 (-48.6 - 2.8)	277.8 (215.7 - 372.3)	193 (145 - 254)	-16.2 (-41.3 - 13.3)	27.5 (20.7 - 35.6)
Haiti	115,365 (81,511 - 166,828)	-42.1 (-63.1 - -13.8)	1,028.4 (767.3 - 1,405.5)	73,722 (46,815 - 118,806)	-38.9 (-68.3 - 9.7)	590.0 (392.3 - 914.4)	37,455 (25,566 - 60,825)	-42.8 (-68.5 - -6.4)	399.1 (295.3 - 584.9)	4,187 (3,082 - 5,517)	-46.5 (-62.7 - -28.1)	39.3 (29.3 - 51.4)
Jamaica	10,467 (8,069 - 14,211)	-8.9 (-41.5 - 36.8)	422.0 (313.9 - 607.0)	4,809 (3,207 - 7,729)	-19.7 (-61.3 - 49.9)	206.5 (130.4 - 362.9)	5,340 (3,973 - 7,479)	7.0 (-31.8 - 66.2)	204.4 (147.7 - 299.8)	318 (232 - 429)	37.9 (-10.2 - 102.1)	11.2 (8.1 - 15.2)
Puerto Rico	16,056 (12,927 - 20,820)	-9.6 (-37.0 - 30.6)	362.9 (278.3 - 492.9)	9,134 (6,448 - 12,902)	5.8 (-38.9 - 74.4)	224.7 (145.0 - 350.7)	5,623 (4,249 - 7,628)	-39.2 (-61.4 - -10.1)	101.7 (75.7 - 140.4)	1,298 (653 - 2,639)	117.2 (-12.9 - 400.9)	36.5 (16.8 - 78.6)
Saint Lucia	657 (541 - 814)	-21.0 (-42.8 - 11.2)	397.6 (313.4 - 533.8)	332 (246 - 458)	-5.0 (-43.1 - 54.7)	216.0 (150.7 - 326.1)	298 (234 - 390)	-32.9 (-54.7 - -2.6)	167.3 (127.3 - 227.9)	27 (20 - 35)	-33.5 (-56.2 - -4.6)	14.3 (10.7 - 19.0)
Saint Vincent and the Grenadines	533 (435 - 676)	-11.2 (-38.1 - 26.2)	485.8 (383.4 - 656.1)	270 (196 - 396)	-5.2 (-45.7 - 57.3)	264.6 (181.0 - 421.6)	243 (191 - 312)	-16.0 (-42.1 - 18.5)	204.6 (157.8 - 266.9)	20 (15 - 25)	-8.0 (-35.4 - 26.2)	16.6 (12.8 - 21.3)
Suriname	3,450 (2,727 - 4,451)	-17.0 (-45.5 - 21.9)	672.3 (519.0 - 890.5)	2,044 (1,416 - 3,025)	-12.7 (-54.8 - 46.4)	409.4 (280.5 - 613.5)	1,269 (992 - 1,655)	-18.9 (-45.4 - 19.2)	238.4 (184.5 - 315.5)	137 (102 - 183)	-27.3 (-51.2 - 5.8)	24.6 (18.2 - 32.8)
Trinidad and Tobago	5,342 (4,147 - 6,965)	-22.7 (-46.0 - 7.3)	384.7 (294.3 - 499.1)	2,325 (1,615 - 3,244)	-8.0 (-47.1 - 41.3)	184.1 (123.8 - 261.3)	2,795 (2,026 - 3,873)	-30.5 (-54.7 - 4.0)	184.8 (135.1 - 255.3)	222 (157 - 312)	-40.1 (-62.0 - -8.1)	15.9 (11.2 - 22.3)
Virgin Islands	493 (401 - 613)	2.5 (-23.6 - 34.3)	382.2 (309.0 - 483.4)	243 (180 - 335)	31.3 (-13.0 - 92.2)	204.6 (147.5 - 285.8)	232 (179 - 296)	-17.0 (-44.5 - 17.0)	162.6 (126.4 - 212.0)	18 (14 - 24)	-15.3 (-44.3 - 23.5)	15.0 (11.3 - 19.9)
Central Latin America	1,000,574 (805,042 - 1,263,150)	-43.1 (-62.3 - -21.5)	417.5 (334.6 - 528.9)	582,299 (409,602 - 820,272)	-45.2 (-70.9 - -10.3)	243.1 (171.1 - 341.8)	373,590 (298,160 - 471,330)	-36.3 (-53.9 - -13.0)	156.9 (125.4 - 198.9)	44,685 (33,899 - 59,303)	-47.0 (-62.7 - -25.3)	17.5 (13.4 - 23.1)
Colombia	164,753 (126,822 - 218,526)	-35.7 (-58.1 - -6.1)	347.5 (261.2 - 477.3)	104,732 (71,151 - 157,052)	-20.5 (-58.5 - 33.8)	227.8 (152.7 - 354.5)	53,999 (42,462 - 70,009)	-48.7 (-65.2 - -26.7)	107.8 (83.3 - 147.3)	6,021 (4,531 - 7,976)	-66.7 (-77.8 - -52.5)	11.9 (8.9 - 15.8)
Costa Rica	10,492 (8,794 - 12,574)	-33.7 (-52.3 - -11.5)	231.4 (190.9 - 284.0)	5,012 (3,680 - 6,792)	-38.6 (-64.0 - -4.2)	112.5 (82.0 - 154.0)	4,842 (3,881 - 6,084)	-26.6 (-50.8 - 2.5)	105.8 (82.6 - 138.4)	638 (458 - 856)	-23.5 (-51.0 - 14.1)	13.2 (9.5 - 17.7)
El Salvador	24,202 (19,029 - 31,698)	-50.8 (-69.1 - -30.0)	420.8 (329.2 - 549.4)	13,892 (10,003 - 19,998)	-52.3 (-76.3 - -17.1)	243.1 (172.9 - 356.8)	9,176 (6,938 - 12,113)	-36.8 (-56.7 - -9.0)	159.1 (120.1 - 210.1)	1,134 (809 - 1,578)	-75.8 (-85.9 - -62.9)	18.5 (13.3 - 25.8)
Guatemala	113,157 (80,779 - 165,523)	-55.2 (-71.3 - -32.5)	711.7 (530.6 - 1,000.4)	79,558 (49,899 - 131,760)	-56.2 (-76.8 - -22.3)	476.9 (313.1 - 765.5)	30,169 (22,882 - 40,824)	-49.4 (-68.2 - -25.6)	213.4 (168.7 - 275.7)	3,430 (2,637 - 4,487)	-60.5 (-72.2 - -45.6)	21.4 (16.6 - 27.5)
Honduras	36,462 (26,744 - 50,694)	-54.0 (-70.2 - -32.1)	444.9 (338.0 - 599.1)	19,772 (12,588 - 31,605)	-56.6 (-78.4 - -22.8)	226.1 (151.1 - 353.5)	15,229 (11,207 - 22,070)	-48.9 (-67.7 - -19.4)	201.9 (154.7 - 279.2)	1,460 (1,077 - 1,958)	-45.9 (-63.6 - -22.7)	16.9 (12.4 - 22.7)
Mexico	483,584 (394,914 - 595,050)	-43.1 (-61.7 - -21.6)	410.7 (334.4 - 507.4)	256,767 (183,609 - 356,872)	-49.7 (-73.1 - -18.1)	217.5 (155.3 - 301.1)	201,946 (160,020 - 256,287)	-30.1 (-49.2 - -2.9)	173.5 (137.8 - 220.3)	24,871 (18,130 - 35,113)	-34.2 (-56.7 - -3.5)	19.8 (14.5 - 27.7)

Nicaragua	21,847 (16,087 - 29,952)	-64.5 (-80.2 - -44.3)	368.1 (278.5 - 496.1)	13,054 (8,352 - 20,278)	-70.3 (-87.3 - -43.0)	210.1 (135.0 - 321.9)	8,160 (6,079 - 11,107)	-47.2 (-65.6 - -23.4)	147.5 (112.6 - 194.2)	634 (466 - 844)	-59.8 (-72.9 - -43.7)	10.5 (7.7 - 13.9)
Panama	16,871 (12,701 - 23,220)	1.4 (-34.1 - 51.1)	454.0 (334.4 - 637.8)	11,196 (7,430 - 17,434)	22.1 (-36.1 - 117.0)	306.5 (200.6 - 483.8)	5,186 (4,006 - 6,824)	-20.4 (-45.3 - 13.3)	135.0 (103.5 - 180.6)	489 (357 - 680)	-40.0 (-63.1 - -8.1)	12.6 (9.2 - 17.5)
Venezuela	129,205 (95,363 - 177,151)	-34.7 (-59.1 - -2.1)	453.9 (333.6 - 629.6)	78,315 (49,861 - 121,175)	-36.0 (-67.6 - 14.2)	277.3 (176.9 - 427.8)	44,882 (33,368 - 59,916)	-29.5 (-53.9 - 2.4)	157.3 (117.8 - 212.4)	6,008 (4,353 - 8,237)	-28.2 (-54.3 - 6.1)	19.3 (14.1 - 26.3)
Tropical Latin America	901,156 (742,183 - 1,112,404)	-50.5 (-68.7 - -30.3)	444.9 (359.5 - 563.1)	561,056 (417,431 - 754,727)	-51.5 (-74.7 - -20.3)	286.1 (210.1 - 399.8)	302,502 (247,917 - 369,892)	-47.0 (-63.3 - -27.5)	141.9 (114.9 - 177.4)	37,598 (28,755 - 49,095)	-39.8 (-59.1 - -16.0)	16.9 (13.0 - 22.2)
Brazil	879,132 (724,533 - 1,085,691)	-50.5 (-68.8 - -29.9)	448.7 (361.5 - 569.6)	550,051 (409,273 - 741,234)	-51.3 (-74.7 - -19.7)	290.2 (212.9 - 405.4)	292,452 (239,614 - 357,255)	-47.3 (-63.6 - -27.9)	141.4 (114.1 - 177.1)	36,629 (28,009 - 47,963)	-40.2 (-59.4 - -16.7)	17.0 (13.0 - 22.3)
Paraguay	22,024 (17,524 - 28,248)	-49.6 (-65.8 - -31.0)	355.8 (288.3 - 449.2)	11,005 (7,718 - 15,303)	-58.7 (-76.3 - -35.1)	173.7 (124.3 - 238.3)	10,049 (7,456 - 13,573)	-35.5 (-57.9 - -7.4)	167.4 (127.7 - 219.7)	969 (700 - 1,313)	-7.7 (-39.2 - 33.1)	14.7 (10.8 - 19.8)
North Africa and Middle East	2,754,140 (2,135,558 - 3,695,975)	-49.4 (-65.5 - -30.5)	487.7 (386.1 - 638.6)	1,536,674 (1,045,124 - 2,345,616)	-54.9 (-74.5 - -25.0)	262.8 (181.9 - 391.9)	770,573 (576,151 - 1,052,928)	-52.2 (-69.8 - -30.9)	152.4 (118.6 - 200.1)	446,893 (252,263 - 832,038)	49.2 (-22.4 - 180.0)	72.5 (41.6 - 133.7)
Afghanistan	293,099 (220,265 - 393,897)	-58.7 (-71.5 - -42.2)	909.7 (724.2 - 1,167.9)	180,876 (124,528 - 262,461)	-65.2 (-79.2 - -47.0)	424.5 (308.9 - 608.6)	97,683 (64,536 - 154,769)	-47.4 (-73.0 - 2.6)	436.3 (321.7 - 645.5)	14,540 (11,071 - 18,739)	-43.3 (-62.5 - -2.3)	48.9 (37.6 - 61.8)
Algeria	106,560 (81,150 - 142,852)	-48.9 (-66.4 - -27.8)	271.6 (212.1 - 355.1)	49,813 (30,796 - 82,232)	-54.1 (-76.1 - -18.9)	124.1 (79.9 - 196.1)	50,238 (35,440 - 77,739)	-41.0 (-64.9 - -6.8)	131.3 (96.1 - 193.4)	6,509 (4,273 - 10,040)	-42.6 (-67.0 - -10.8)	16.2 (10.7 - 24.6)
Bahrain	1,869 (1,459 - 2,435)	-41.2 (-56.8 - -18.7)	221.7 (172.1 - 291.5)	893 (635 - 1,216)	-29.1 (-52.1 - 1.9)	108.9 (78.5 - 147.5)	831 (575 - 1,267)	-49.4 (-68.8 - -19.1)	101.8 (70.1 - 157.8)	145 (97 - 213)	-41.2 (-69.3 - -5.3)	10.9 (7.4 - 16.1)
Egypt	467,600 (322,255 - 724,128)	-58.1 (-75.7 - -34.9)	554.5 (405.6 - 788.0)	336,817 (194,144 - 593,182)	-61.0 (-81.7 - -28.1)	365.2 (226.0 - 602.3)	108,071 (83,599 - 147,609)	-49.8 (-67.5 - -28.6)	165.1 (131.9 - 210.7)	22,712 (15,478 - 32,744)	-34.6 (-62.2 - 1.3)	24.2 (16.7 - 34.7)
Iran	169,177 (136,717 - 214,911)	-52.6 (-67.1 - -34.1)	249.1 (199.7 - 317.4)	76,301 (54,116 - 104,927)	-53.1 (-73.3 - -24.6)	110.9 (79.6 - 154.9)	75,779 (55,808 - 110,402)	-42.0 (-65.8 - -4.7)	116.6 (84.1 - 173.4)	17,097 (12,196 - 23,717)	-71.5 (-84.5 - -53.2)	21.6 (15.5 - 30.1)
Iraq	231,157 (158,830 - 347,293)	-44.5 (-66.4 - -12.5)	495.6 (352.5 - 716.9)	83,711 (53,209 - 133,663)	-66.8 (-83.5 - -42.9)	161.5 (106.6 - 252.8)	43,625 (28,197 - 71,707)	-64.7 (-77.8 - -46.5)	115.3 (84.6 - 167.7)	103,821 (47,892 - 205,672)	255.3 (55.8 - 628.6)	218.8 (101.4 - 424.8)
Jordan	29,804 (20,898 - 45,904)	-51.6 (-68.5 - -26.5)	322.3 (239.4 - 465.2)	18,145 (11,020 - 30,788)	-48.4 (-73.9 - -5.7)	179.8 (114.1 - 293.8)	10,300 (6,614 - 17,395)	-54.1 (-72.3 - -25.3)	129.7 (92.6 - 195.8)	1,359 (979 - 1,869)	-46.1 (-66.3 - -16.9)	12.9 (9.3 - 17.6)
Kuwait	4,936 (3,813 - 6,592)	-63.4 (-78.1 - -43.8)	169.7 (128.3 - 231.9)	2,821 (1,906 - 4,317)	-16.4 (-54.5 - 43.3)	101.5 (67.6 - 159.2)	1,714 (1,234 - 2,571)	-44.7 (-66.2 - -10.5)	58.1 (42.2 - 87.4)	401 (262 - 612)	-95.4 (-98.1 - -90.7)	10.1 (6.7 - 15.1)
Lebanon	21,067 (15,550 - 28,949)	-46.7 (-63.9 - -23.9)	274.2 (209.1 - 365.5)	11,418 (6,976 - 18,680)	-36.3 (-66.7 - 11.2)	142.7 (91.1 - 226.1)	7,039 (5,161 - 9,959)	-49.0 (-67.5 - -23.4)	101.9 (76.1 - 138.7)	2,611 (1,347 - 5,332)	-62.3 (-84.3 - -15.7)	29.6 (15.8 - 58.8)
Libya	22,799 (17,160 - 30,707)	-15.5 (-43.5 - 19.4)	380.0 (296.4 - 497.1)	8,638 (5,724 - 13,285)	-39.8 (-67.5 - -2.2)	143.0 (97.5 - 208.8)	6,680 (5,043 - 9,000)	-26.1 (-53.1 - 6.3)	130.9 (99.7 - 171.7)	7,480 (3,859 - 14,523)	297.1 (82.7 - 706.1)	106.1 (55.4 - 205.5)
Morocco	126,025 (94,504 - 168,715)	-66.9 (-79.8 - -49.2)	401.2 (301.9 - 538.2)	64,529 (42,221 - 101,320)	-69.7 (-85.5 - -48.0)	205.5 (133.6 - 318.9)	55,395 (39,123 - 78,947)	-62.0 (-81.8 - -35.7)	178.0 (127.3 - 252.7)	6,101 (3,995 - 9,625)	-42.6 (-66.7 - -8.7)	17.6 (11.6 - 27.7)
Palestine	11,047 (8,390 - 14,854)	-47.3 (-61.7 - -27.9)	276.4 (226.6 - 347.5)	6,295 (4,331 - 9,319)	-48.9 (-69.5 - -20.4)	138.6 (101.8 - 194.4)	3,899 (2,767 - 5,862)	-30.0 (-52.5 - -0.9)	120.7 (93.3 - 162.2)	853 (553 - 1,319)	-76.2 (-89.0 - -55.7)	17.1 (11.4 - 25.5)
Oman	8,197 (6,262 - 11,008)	-49.2 (-65.0 - -28.5)	271.8 (212.1 - 350.3)	4,429 (3,164 - 6,380)	-51.3 (-70.9 - -22.9)	148.6 (107.5 - 210.8)	2,775 (1,840 - 4,306)	-42.8 (-65.1 - -9.7)	100.8 (71.5 - 147.0)	992 (520 - 1,876)	-50.2 (-79.6 - -1.6)	22.5 (12.3 - 40.7)
Qatar	2,544 (1,953 - 3,392)	-49.0 (-64.9 - -26.0)	202.2 (154.0 - 277.6)	1,188 (802 - 1,787)	-41.3 (-66.5 - -6.5)	91.4 (63.8 - 133.2)	930 (637 - 1,455)	-54.8 (-72.9 - -25.1)	94.6 (64.8 - 147.4)	425 (255 - 694)	-36.3 (-67.7 - 5.2)	16.3 (10.0 - 26.2)

Saudi Arabia	85,617 (62,527 - 124,210)	-43.6 (-64.1 - -12.7)	380.2 (288.3 - 538.9)	47,807 (29,828 - 82,875)	-46.5 (-72.0 - -3.1)	229.4 (153.1 - 379.2)	18,839 (13,289 - 27,730)	-48.4 (-69.1 - -20.4)	97.3 (72.5 - 134.6)	18,971 (10,773 - 32,935)	17.7 (-38.1 - 122.7)	53.6 (31.5 - 92.2)
Sudan	415,580 (273,439 - 669,632)	-46.8 (-68.7 - -15.2)	962.4 (651.8 - 1,486.9)	311,972 (179,340 - 569,655)	-40.9 (-72.3 - 11.0)	688.0 (403.1 - 1,218.5)	89,505 (55,837 - 145,577)	-53.2 (-74.9 - -24.1)	240.8 (172.0 - 348.4)	14,102 (9,672 - 19,504)	-63.8 (-77.1 - -45.4)	33.7 (23.2 - 46.9)
Syria	183,938 (89,427 - 364,110)	94.0 (-5.7 - 287.1)	1,084.5 (553.1 - 2,106.3)	19,555 (13,952 - 28,357)	-56.4 (-74.2 - -32.5)	130.3 (95.2 - 184.3)	11,593 (8,841 - 15,482)	-60.8 (-74.3 - -43.4)	91.7 (69.8 - 121.0)	152,790 (57,813 - 333,835)	4,573.3 (1,603.2 - 9,866.8)	862.5 (323.3 - 1,889.9)
Tunisia	22,699 (17,424 - 29,519)	-52.9 (-68.0 - -33.9)	215.6 (164.4 - 279.1)	10,479 (7,022 - 15,936)	-54.8 (-75.1 - -24.9)	101.4 (67.9 - 154.2)	9,947 (7,329 - 13,072)	-50.5 (-69.9 - -26.7)	94.4 (68.9 - 129.0)	2,272 (1,450 - 3,380)	-43.4 (-67.6 - -8.4)	19.8 (12.7 - 29.3)
Turkey	196,555 (154,220 - 252,272)	-65.5 (-76.9 - -51.0)	272.3 (208.9 - 359.8)	96,692 (64,515 - 141,273)	-70.3 (-83.7 - -49.9)	138.7 (89.6 - 207.6)	80,942 (61,749 - 111,130)	-61.9 (-76.7 - -40.0)	109.2 (79.6 - 162.3)	18,921 (11,249 - 33,286)	6.3 (-40.6 - 95.4)	24.4 (14.4 - 43.3)
United Arab Emirates	12,416 (9,515 - 15,848)	-17.9 (-41.4 - 11.2)	321.9 (250.8 - 416.3)	5,170 (3,617 - 7,382)	-11.5 (-45.8 - 38.9)	156.8 (103.7 - 231.5)	4,705 (3,418 - 6,233)	-22.1 (-48.9 - 10.5)	133.7 (98.8 - 182.4)	2,541 (1,538 - 4,024)	-18.4 (-58.9 - 43.3)	31.5 (19.4 - 49.2)
Yemen	338,884 (224,659 - 545,892)	-47.3 (-69.6 - -16.2)	1,017.6 (708.5 - 1,529.7)	197,690 (100,012 - 392,422)	-56.7 (-81.0 - -13.8)	533.1 (281.5 - 1,000.9)	89,362 (51,103 - 156,501)	-44.5 (-74.6 - 5.9)	326.7 (208.5 - 532.1)	51,832 (29,362 - 94,400)	206.4 (42.2 - 567.1)	157.9 (92.4 - 280.4)
South Asia	14,830,172 (11,479,725 - 19,802,861)	-46.4 (-63.2 - -23.3)	982.9 (763.2 - 1,348.8)	10,099,467 (7,120,659 - 15,144,208)	-45.1 (-67.6 - -11.1)	668.0 (467.5 - 1,030.3)	4,234,866 (3,304,735 - 5,491,136)	-48.9 (-65.6 - -28.1)	282.8 (224.6 - 357.4)	495,838 (359,615 - 674,720)	-30.1 (-52.9 - -2.2)	32.0 (23.5 - 43.8)
Bangladesh	1,147,722 (805,921 - 1,638,393)	-55.2 (-70.0 - -35.1)	820.2 (593.0 - 1,137.1)	780,190 (475,194 - 1,270,764)	-49.2 (-71.9 - -14.7)	546.3 (339.5 - 867.7)	347,180 (263,575 - 467,124)	-63.1 (-75.2 - -47.1)	259.7 (199.7 - 343.9)	20,352 (15,605 - 26,655)	-50.5 (-66.3 - -30.8)	14.2 (10.9 - 18.4)
Bhutan	4,413 (3,331 - 5,805)	-62.0 (-73.4 - -48.6)	565.1 (434.3 - 729.0)	2,568 (1,768 - 3,777)	-58.7 (-74.2 - -39.1)	331.7 (228.5 - 487.8)	1,665 (1,171 - 2,422)	-65.8 (-79.3 - -48.9)	211.0 (158.3 - 280.9)	180 (121 - 257)	-52.5 (-70.0 - -27.7)	22.4 (15.4 - 31.7)
India	11,341,313 (8,695,622 - 15,418,974)	-45.9 (-63.2 - -21.3)	992.2 (766.4 - 1,383.4)	8,027,453 (5,676,631 - 12,108,556)	-43.8 (-66.7 - -7.5)	703.0 (492.3 - 1,089.7)	2,898,369 (2,271,449 - 3,777,080)	-50.3 (-67.3 - -29.3)	254.7 (203.2 - 323.7)	415,492 (292,021 - 583,256)	-31.6 (-54.9 - -2.3)	34.6 (24.5 - 48.3)
Nepal	192,301 (147,466 - 253,169)	-61.6 (-73.6 - -45.7)	767.2 (594.9 - 1,005.1)	120,259 (84,614 - 178,684)	-62.7 (-77.5 - -40.4)	474.6 (335.5 - 701.4)	65,300 (47,891 - 91,237)	-59.8 (-76.1 - -37.3)	266.0 (204.7 - 354.9)	6,742 (4,743 - 9,421)	-29.9 (-53.8 - -0.9)	26.7 (18.7 - 36.7)
Pakistan	2,144,423 (1,622,391 - 2,896,891)	-44.8 (-63.4 - -21.5)	1,045.7 (817.0 - 1,384.5)	1,168,998 (768,704 - 1,855,594)	-52.3 (-74.4 - -18.6)	537.0 (364.4 - 849.2)	922,353 (634,468 - 1,356,782)	-32.3 (-57.1 - 4.9)	480.5 (359.7 - 661.4)	53,072 (38,305 - 71,720)	-3.2 (-33.7 - 32.9)	28.2 (20.4 - 37.4)
Southeast Asia, east Asia, and Oceania	7,137,538 (5,968,981 - 8,661,167)	-51.2 (-64.3 - -36.4)	367.4 (299.4 - 460.2)	4,034,964 (3,036,829 - 5,490,474)	-48.0 (-66.9 - -23.7)	225.5 (164.8 - 311.5)	2,769,998 (2,276,315 - 3,447,725)	-55.3 (-69.9 - -37.8)	126.5 (102.5 - 161.1)	332,576 (243,992 - 446,959)	-45.0 (-63.6 - -21.0)	15.4 (11.3 - 20.8)
East Asia	3,161,125 (2,623,108 - 3,889,553)	-64.1 (-74.4 - -51.5)	219.6 (176.7 - 278.9)	1,358,866 (992,720 - 1,957,728)	-66.4 (-80.0 - -46.4)	109.5 (76.8 - 160.8)	1,588,643 (1,281,016 - 2,026,216)	-62.1 (-75.1 - -44.7)	95.9 (76.0 - 124.5)	213,615 (149,505 - 300,013)	-43.3 (-63.9 - -14.7)	14.2 (10.0 - 20.0)
China	2,931,821 (2,430,160 - 3,620,068)	-65.3 (-75.3 - -53.1)	214.8 (171.6 - 274.4)	1,241,273 (902,338 - 1,805,717)	-67.8 (-81.2 - -48.4)	106.1 (73.7 - 157.0)	1,488,253 (1,200,034 - 1,901,952)	-63.1 (-75.7 - -45.9)	94.6 (75.1 - 123.0)	202,295 (140,882 - 284,130)	-43.8 (-64.3 - -15.5)	14.1 (10.0 - 19.9)
North Korea	89,754 (71,305 - 113,281)	-15.1 (-41.7 - 17.8)	402.8 (311.2 - 539.8)	36,475 (26,332 - 54,891)	-18.5 (-52.4 - 35.3)	192.1 (130.0 - 309.3)	48,230 (36,210 - 64,558)	-12.1 (-44.6 - 33.7)	190.5 (141.7 - 256.4)	5,048 (3,548 - 7,165)	26.3 (-22.5 - 92.2)	20.2 (14.3 - 28.3)
Taiwan (province of China)	88,627 (69,294 - 118,696)	-0.2 (-25.4 - 33.7)	273.9 (215.8 - 357.3)	59,227 (40,911 - 86,842)	45.2 (-8.4 - 116.6)	184.4 (130.4 - 261.6)	26,568 (20,805 - 35,228)	-33.1 (-51.2 - -8.3)	79.2 (62.2 - 103.0)	2,832 (1,991 - 4,074)	-60.8 (-79.3 - -37.8)	10.3 (7.3 - 14.6)
Oceania	120,380 (92,915 - 163,068)	-21.2 (-45.0 - 9.4)	1,117.9 (894.1 - 1,449.0)	69,562 (49,452 - 103,478)	-19.2 (-47.9 - 21.7)	600.2 (443.9 - 864.2)	45,842 (31,405 - 70,891)	-21.7 (-53.8 - 21.4)	475.7 (353.2 - 681.9)	4,976 (3,692 - 6,516)	-17.1 (-41.9 - 14.1)	42.0 (31.6 - 54.2)
American Samoa	228 (186 - 280)	-3.2 (-25.1 - 22.4)	518.7 (428.9 - 631.8)	133 (100 - 178)	2.2 (-28.5 - 41.6)	297.7 (226.9 - 395.3)	86 (66 - 113)	-7.6 (-34.4 - 27.0)	203.0 (157.7 - 265.1)	9 (6 - 12)	-15.2 (-44.4 - 25.8)	18.0 (13.1 - 24.4)

Federated States of Micronesia	429 (347 - 536)	-35.9 (-51.3 - -16.2)	553.0 (457.5 - 671.0)	204 (149 - 281)	-42.6 (-61.1 - -16.1)	245.1 (182.6 - 335.7)	204 (151 - 272)	-28.8 (-50.7 - -0.9)	284.6 (221.3 - 367.8)	21 (14 - 31)	-22.4 (-52.2 - 14.2)	23.3 (15.2 - 32.4)
Fiji	4,853 (3,751 - 6,377)	-3.6 (-29.5 - 37.2)	637.5 (493.6 - 835.1)	2,551 (1,812 - 3,693)	-13.8 (-44.3 - 31.5)	325.0 (233.6 - 466.8)	2,164 (1,510 - 3,189)	13.5 (-27.5 - 72.2)	296.2 (206.6 - 438.2)	139 (102 - 189)	-8.6 (-39.0 - 33.1)	16.4 (12.2 - 22.1)
Guam	651 (528 - 810)	3.6 (-23.3 - 37.6)	393.1 (317.8 - 491.0)	392 (289 - 538)	5.3 (-32.4 - 54.9)	237.9 (174.6 - 326.5)	235 (181 - 304)	3.7 (-27.5 - 44.1)	140.3 (108.0 - 182.0)	25 (17 - 35)	1.9 (-41.0 - 56.4)	14.9 (10.4 - 21.0)
Kiribati	810 (652 - 1,018)	-32.6 (-50.9 - -10.9)	919.1 (752.5 - 1,142.5)	403 (300 - 565)	-43.0 (-63.4 - -16.9)	425.7 (319.8 - 589.2)	384 (297 - 517)	-18.4 (-43.7 - 14.1)	471.5 (371.6 - 602.2)	23 (17 - 32)	-10.8 (-39.3 - 29.3)	21.9 (16.2 - 29.7)
Marshall Islands	285 (231 - 366)	-21.7 (-40.0 - -1.1)	678.5 (567.5 - 835.2)	142 (105 - 197)	-27.9 (-50.6 - 1.2)	311.2 (237.6 - 424.2)	129 (97 - 171)	-14.6 (-38.1 - 13.1)	338.2 (267.0 - 426.2)	14 (11 - 20)	-13.0 (-40.5 - 22.0)	29.2 (21.5 - 39.1)
Northern Mariana Islands	140 (112 - 176)	-18.9 (-42.3 - 11.3)	336.2 (271.0 - 420.3)	79 (58 - 110)	-14.7 (-47.0 - 28.7)	196.0 (142.8 - 267.8)	55 (42 - 74)	-22.3 (-47.6 - 12.4)	127.1 (98.6 - 164.7)	6 (4 - 7)	-21.2 (-51.3 - 15.4)	13.1 (9.3 - 17.8)
Papua New Guinea	100,223 (75,433 - 139,219)	-26.9 (-50.4 - 3.2)	1,301.6 (1,022.9 - 1,729.9)	58,168 (40,365 - 87,961)	-23.3 (-51.7 - 17.6)	704.1 (513.1 - 1,037.4)	37,895 (24,685 - 61,429)	-28.9 (-61.3 - 14.9)	549.3 (393.3 - 802.2)	4,159 (3,027 - 5,505)	-24.3 (-47.6 - 5.6)	48.2 (35.7 - 62.5)
Samoa	637 (509 - 801)	-19.4 (-38.3 - 3.4)	391.6 (321.5 - 480.1)	352 (255 - 503)	-19.3 (-44.5 - 14.0)	195.0 (146.7 - 269.5)	260 (201 - 330)	-17.7 (-40.5 - 9.6)	181.7 (142.8 - 226.3)	26 (18 - 35)	-27.9 (-52.6 - 2.9)	14.9 (10.7 - 20.0)
Solomon Islands	3,439 (2,706 - 4,319)	-31.8 (-51.5 - -9.4)	748.5 (618.4 - 922.7)	2,144 (1,548 - 2,969)	-37.5 (-59.3 - -10.0)	430.4 (328.8 - 576.9)	1,105 (862 - 1,416)	-21.2 (-43.4 - 5.5)	280.0 (225.7 - 347.8)	189 (139 - 250)	-17.9 (-45.5 - 20.3)	38.1 (27.7 - 50.5)
Tonga	508 (402 - 649)	-20.4 (-41.1 - 5.4)	570.3 (454.6 - 718.9)	293 (215 - 410)	-23.6 (-50.4 - 10.8)	312.9 (231.8 - 426.6)	200 (149 - 266)	-14.6 (-41.7 - 22.0)	241.5 (182.1 - 319.9)	15 (11 - 20)	-16.7 (-43.4 - 15.5)	15.9 (11.9 - 20.8)
Vanuatu	1,542 (1,130 - 2,126)	-24.5 (-45.2 - 2.9)	691.7 (527.3 - 917.2)	868 (586 - 1,350)	-34.2 (-56.8 - -0.6)	342.4 (237.4 - 523.4)	599 (446 - 782)	-11.4 (-35.7 - 18.2)	318.7 (245.6 - 399.6)	75 (49 - 109)	-1.3 (-35.0 - 45.1)	30.5 (20.3 - 43.6)
Southeast Asia	3,834,335 (3,114,076 - 4,825,671)	-39.6 (-56.3 - -19.0)	675.7 (547.1 - 847.8)	2,588,007 (1,889,258 - 3,498,271)	-34.7 (-58.0 - -3.3)	459.7 (336.8 - 626.0)	1,132,405 (920,723 - 1,443,364)	-45.8 (-64.6 - -23.8)	197.7 (160.9 - 251.4)	113,922 (87,558 - 147,132)	-51.1 (-65.7 - -32.9)	18.3 (14.0 - 23.4)
Cambodia	99,110 (79,571 - 125,665)	-57.9 (-70.3 - -44.0)	734.1 (605.4 - 900.2)	63,081 (46,480 - 85,360)	-54.5 (-69.5 - -35.8)	452.1 (342.3 - 602.2)	30,173 (23,710 - 40,041)	-62.5 (-79.9 - -42.2)	239.3 (196.1 - 300.8)	5,856 (3,881 - 8,430)	-48.9 (-66.0 - -26.7)	42.7 (28.2 - 61.5)
Indonesia	1,635,563 (1,282,430 - 2,128,315)	-44.2 (-62.1 - -23.8)	794.9 (623.9 - 1,047.5)	1,092,211 (764,774 - 1,577,531)	-40.9 (-64.8 - -6.0)	533.7 (368.0 - 786.2)	508,216 (405,580 - 664,095)	-47.2 (-68.1 - -22.5)	245.7 (199.8 - 312.6)	35,136 (26,754 - 45,616)	-55.2 (-69.6 - -36.8)	15.5 (11.8 - 20.0)
Laos	55,965 (41,810 - 78,731)	-57.6 (-71.3 - -38.4)	864.9 (676.7 - 1,169.2)	37,295 (24,906 - 58,154)	-52.7 (-71.2 - -24.1)	538.4 (372.5 - 821.4)	17,021 (12,325 - 24,541)	-62.7 (-78.9 - -41.5)	300.7 (233.9 - 398.2)	1,650 (1,191 - 2,163)	-58.0 (-70.6 - -41.3)	25.8 (19.1 - 33.6)
Malaysia	149,152 (115,814 - 195,214)	-1.7 (-30.1 - 35.0)	576.5 (450.8 - 753.1)	114,424 (83,368 - 158,943)	24.8 (-19.8 - 88.6)	443.5 (324.4 - 617.8)	29,779 (23,723 - 37,862)	-42.5 (-58.3 - -23.0)	116.5 (93.3 - 145.5)	4,950 (3,482 - 7,030)	-29.8 (-55.6 - 4.2)	16.4 (11.7 - 23.1)
Maldives	1,148 (814 - 1,660)	-72.3 (-85.4 - -55.7)	319.3 (231.4 - 454.6)	779 (485 - 1,275)	-50.1 (-74.1 - -13.5)	218.2 (138.4 - 346.3)	336 (238 - 522)	-85.7 (-93.9 - -74.9)	93.0 (71.1 - 130.0)	32 (23 - 50)	-67.1 (-79.0 - -48.8)	8.1 (5.7 - 12.0)
Mauritius	4,622 (3,684 - 6,001)	-24.2 (-45.7 - 7.1)	378.6 (294.6 - 498.1)	1,933 (1,423 - 2,590)	-22.7 (-55.3 - 25.4)	183.7 (128.3 - 267.8)	2,514 (1,811 - 3,635)	-24.4 (-51.7 - 12.9)	181.5 (130.9 - 262.7)	175 (123 - 243)	-11.5 (-47.6 - 35.0)	13.4 (9.5 - 18.9)
Myanmar	388,555 (304,598 - 494,445)	-45.2 (-60.5 - -27.5)	854.0 (673.6 - 1,075.3)	235,342 (167,794 - 321,143)	-44.7 (-64.0 - -18.7)	516.7 (368.1 - 711.2)	140,886 (105,195 - 209,929)	-44.3 (-63.4 - -18.9)	312.1 (239.4 - 441.5)	12,327 (9,115 - 16,243)	-49.5 (-64.3 - -28.6)	25.3 (18.7 - 33.4)
Philippines	763,194 (576,556 - 1,032,639)	-15.2 (-40.2 - 13.8)	834.6 (654.2 - 1,084.3)	560,710 (392,733 - 814,870)	-15.6 (-45.9 - 27.4)	595.5 (434.5 - 835.7)	184,905 (141,553 - 247,918)	-9.3 (-37.7 - 27.1)	221.3 (176.5 - 281.6)	17,580 (13,272 - 22,732)	-33.1 (-53.7 - -7.9)	17.8 (13.5 - 22.8)
Sri Lanka	82,724 (61,557 - 115,821)	-45.1 (-63.2 - -19.1)	413.2 (303.8 - 586.8)	54,502 (35,712 - 82,537)	-36.5 (-64.6 - 6.0)	278.6 (180.4 - 429.8)	24,778 (18,021 - 34,118)	-39.2 (-59.7 - -10.9)	118.9 (86.2 - 165.7)	3,444 (2,429 - 4,731)	-84.9 (-92.3 - -75.0)	15.7 (11.2 - 21.5)
Seychelles	499 (406 - 631)	-2.5 (-26.9 - 30.0)	528.9 (425.1 - 678.9)	322 (237 - 440)	12.3 (-27.2 - 64.3)	351.3 (257.2 - 489.1)	163 (130 - 209)	-21.9 (-45.1 - 5.8)	163.7 (130.0 - 211.8)	14 (11 - 20)	-14.0 (-43.1 - 26.2)	13.9 (10.3 - 18.9)
Thailand	325,519 (258,245 - 429,705)	-24.7 (-47.8 - 5.9)	484.9 (371.9 - 668.3)	230,771 (169,080 - 331,185)	-8.9 (-45.3 - 43.5)	365.3 (255.8 - 540.7)	82,168 (65,637 - 101,588)	-50.5 (-65.9 - -32.1)	102.1 (79.8 - 130.7)	12,580 (9,038 - 17,186)	-41.5 (-61.0 - -12.5)	17.5 (12.6 - 24.0)

Timor-Leste	10,371 (7,061 - 15,261)	-51.9 (-74.0 - -22.7)	877.8 (599.4 - 1,288.2)	6,098 (3,912 - 9,387)	-48.1 (-70.1 - -12.7)	478.7 (323.9 - 728.2)	4,098 (2,306 - 7,915)	-50.7 (-81.4 - 5.3)	384.1 (224.6 - 711.8)	175 (106 - 240)	-78.3 (-87.0 - -67.9)	15.0 (9.7 - 20.7)
Vietnam	312,868 (251,070 - 398,503)	-43.9 (-58.6 - -24.9)	370.7 (296.0 - 477.9)	187,134 (135,213 - 262,912)	-41.4 (-62.8 - -9.3)	224.3 (160.8 - 321.7)	105,880 (84,904 - 133,493)	-48.2 (-64.3 - -27.4)	125.3 (100.6 - 160.6)	19,854 (14,449 - 26,920)	-32.2 (-56.3 - -3.1)	21.2 (15.4 - 29.0)
Sub-Saharan Africa	16,681,190 (12,117,506 - 23,332,529)	-36.8 (-56.8 - -10.6)	1,527.2 (1,173.2 - 2,015.3)	13,220,961 (8,927,161 - 19,581,711)	-35.1 (-60.9 - 0.4)	1,130.1 (800.8 - 1,597.8)	3,176,954 (2,266,939 - 4,702,163)	-38.3 (-61.9 - -6.6)	365.1 (281.9 - 512.1)	283,275 (227,939 - 347,972)	-48.9 (-61.0 - -34.2)	31.9 (25.9 - 38.4)
Central sub-Saharan Africa	1,943,946 (1,399,701 - 2,791,560)	-31.8 (-53.8 - -2.1)	1,605.3 (1,223.5 - 2,174.1)	1,548,094 (1,026,683 - 2,351,889)	-30.6 (-58.6 - 8.7)	1,190.3 (827.2 - 1,743.1)	352,806 (254,875 - 495,764)	-33.0 (-56.8 - -3.6)	377.0 (291.9 - 500.7)	43,046 (32,728 - 56,341)	-30.6 (-50.3 - -4.8)	38.0 (29.7 - 48.6)
Angola	388,426 (269,922 - 569,174)	-48.9 (-65.4 - -25.4)	1,393.3 (1,042.2 - 1,895.9)	302,615 (194,584 - 479,936)	-44.9 (-67.5 - -13.1)	1,002.2 (693.3 - 1,504.7)	78,613 (53,338 - 121,365)	-52.3 (-74.6 - -22.6)	362.0 (268.2 - 520.1)	7,198 (5,297 - 9,827)	-72.1 (-81.0 - -58.0)	29.1 (21.8 - 39.1)
Central African Republic	101,514 (72,392 - 147,908)	-6.4 (-33.0 - 33.1)	2,365.1 (1,775.9 - 3,228.5)	75,749 (49,004 - 119,545)	-3.5 (-37.5 - 48.0)	1,673.7 (1,152.3 - 2,452.2)	19,728 (13,378 - 31,642)	-20.3 (-52.8 - 26.7)	557.3 (412.5 - 826.1)	6,038 (4,374 - 8,152)	95.3 (30.8 - 203.3)	134.1 (99.3 - 176.1)
Congo (Brazzaville)	59,031 (42,984 - 85,001)	-26.5 (-48.5 - 3.7)	1,397.7 (1,067.4 - 1,912.4)	45,663 (30,691 - 70,086)	-23.8 (-51.9 - 17.3)	1,032.5 (729.3 - 1,506.2)	12,134 (8,557 - 17,743)	-30.9 (-53.0 - -0.9)	335.8 (254.6 - 452.6)	1,233 (873 - 1,728)	-42.1 (-61.1 - -16.2)	29.4 (21.4 - 39.9)
DR Congo	1,361,764 (952,188 - 2,023,649)	-26.6 (-52.3 - 8.7)	1,666.2 (1,245.9 - 2,308.5)	1,097,977 (702,436 - 1,756,576)	-26.9 (-58.5 - 17.6)	1,250.9 (846.1 - 1,892.1)	235,819 (165,569 - 338,955)	-24.2 (-51.4 - 10.3)	378.7 (286.2 - 513.6)	27,968 (20,551 - 37,223)	-9.5 (-38.6 - 28.8)	36.6 (27.8 - 47.5)
Equatorial Guinea	17,778 (10,400 - 30,240)	-48.3 (-70.7 - -14.3)	1,429.9 (885.0 - 2,284.9)	14,638 (7,925 - 26,650)	-37.0 (-67.2 - 10.4)	1,116.9 (654.0 - 1,870.4)	2,920 (1,573 - 4,829)	-67.1 (-83.7 - -42.9)	293.4 (177.9 - 448.9)	220 (135 - 341)	-70.3 (-82.0 - -53.4)	19.6 (12.5 - 29.9)
Gabon	15,434 (11,250 - 21,446)	-35.1 (-57.0 - -8.7)	1,016.5 (772.3 - 1,366.9)	11,452 (7,233 - 17,684)	-35.3 (-62.8 - 2.6)	727.0 (488.4 - 1,082.8)	3,593 (2,664 - 4,967)	-32.6 (-52.5 - -8.3)	263.6 (205.9 - 341.2)	389 (271 - 528)	-36.3 (-58.4 - -7.2)	25.9 (18.4 - 34.5)
Eastern sub-Saharan Africa	4,618,230 (3,499,703 - 6,197,410)	-48.8 (-63.0 - -30.7)	1,236.7 (984.5 - 1,578.2)	3,460,813 (2,463,264 - 5,029,670)	-47.1 (-65.2 - -22.9)	857.9 (641.3 - 1,216.1)	1,054,014 (767,247 - 1,544,123)	-49.0 (-69.2 - -24.5)	345.0 (271.9 - 466.6)	103,403 (83,124 - 126,472)	-63.6 (-72.8 - -52.9)	33.7 (27.5 - 40.8)
Burundi	156,548 (108,596 - 227,692)	-45.0 (-63.7 - -21.2)	1,505.5 (1,149.1 - 2,007.8)	122,918 (78,952 - 191,121)	-38.0 (-63.2 - -3.7)	1,086.0 (762.8 - 1,571.8)	30,547 (21,480 - 42,973)	-56.9 (-76.4 - -35.2)	380.7 (293.4 - 512.2)	3,082 (2,360 - 3,963)	-35.7 (-53.8 - -13.3)	38.8 (30.2 - 49.9)
Comoros	5,073 (3,809 - 7,015)	-50.3 (-65.5 - -30.8)	835.2 (659.6 - 1,115.5)	3,474 (2,402 - 5,371)	-46.5 (-68.3 - -15.0)	547.1 (395.4 - 818.7)	1,452 (1,120 - 1,955)	-55.8 (-71.5 - -39.1)	262.3 (210.2 - 332.3)	147 (108 - 194)	-45.1 (-61.7 - -25.5)	25.8 (19.2 - 33.9)
Djibouti	8,864 (6,011 - 12,977)	-42.9 (-61.9 - -17.6)	900.2 (644.0 - 1,257.3)	5,637 (3,626 - 8,957)	-42.8 (-66.7 - -9.1)	546.9 (372.1 - 832.5)	3,012 (1,833 - 4,671)	-39.6 (-63.7 - -5.9)	327.4 (220.5 - 476.3)	215 (150 - 318)	-55.7 (-70.4 - -36.7)	25.9 (18.4 - 37.6)
Eritrea	68,399 (49,345 - 99,396)	-66.2 (-76.5 - -51.7)	1,439.3 (1,094.2 - 1,977.5)	45,608 (29,890 - 72,899)	-41.4 (-63.3 - -8.1)	918.6 (648.5 - 1,377.5)	21,057 (13,288 - 35,274)	-52.7 (-74.3 - -21.0)	479.2 (337.8 - 715.6)	1,734 (1,310 - 2,317)	-97.4 (-98.4 - -96.3)	41.4 (31.8 - 54.1)
Ethiopia	1,018,392 (743,662 - 1,470,742)	-59.5 (-72.1 - -43.4)	1,055.6 (831.7 - 1,419.5)	740,224 (501,571 - 1,174,922)	-53.9 (-68.7 - -27.8)	707.1 (515.0 - 1,052.4)	257,349 (176,970 - 414,142)	-64.9 (-81.8 - -41.4)	320.2 (242.1 - 480.4)	20,819 (16,491 - 25,761)	-75.7 (-82.4 - -67.7)	28.3 (22.3 - 34.8)
Kenya	594,293 (446,614 - 848,100)	-30.4 (-52.2 - -0.1)	1,382.9 (1,077.7 - 1,893.0)	466,278 (322,060 - 720,179)	-28.7 (-57.2 - 14.8)	1,029.7 (734.6 - 1,550.9)	118,497 (86,024 - 167,135)	-34.0 (-58.0 - -5.5)	326.1 (251.9 - 426.8)	9,518 (7,381 - 11,962)	-13.6 (-38.7 - 15.3)	27.2 (21.1 - 34.4)
Madagascar	395,322 (264,112 - 614,991)	-26.6 (-52.7 - 13.7)	1,479.5 (1,069.3 - 2,093.9)	294,840 (174,849 - 515,727)	-25.7 (-60.1 - 30.0)	992.8 (647.3 - 1,612.0)	95,193 (60,579 - 155,206)	-24.5 (-53.9 - 24.1)	459.6 (327.4 - 710.2)	5,288 (4,043 - 6,763)	-35.8 (-53.4 - -14.8)	27.2 (21.3 - 35.0)
Malawi	204,568 (155,399 - 276,982)	-54.5 (-70.2 - -32.9)	1,250.8 (986.5 - 1,618.6)	159,345 (112,182 - 227,390)	-57.5 (-74.5 - -33.7)	918.8 (678.6 - 1,266.4)	41,878 (31,112 - 59,215)	-41.1 (-62.3 - -1.0)	305.6 (244.8 - 395.1)	3,345 (2,548 - 4,288)	-37.6 (-60.4 - 8.8)	26.4 (20.3 - 33.1)
Mozambique	398,491 (298,453 - 540,421)	-42.0 (-60.0 - -18.9)	1,473.6 (1,167.7 - 1,934.1)	314,111 (216,877 - 462,223)	-38.8 (-62.7 - -8.3)	1,076.2 (793.1 - 1,515.4)	76,694 (55,499 - 107,507)	-47.0 (-66.3 - -20.9)	361.0 (285.1 - 468.7)	7,686 (6,020 - 9,654)	-49.9 (-62.6 - -33.9)	36.4 (28.5 - 45.4)
Rwanda	121,970 (88,220 - 169,639)	-59.9 (-72.6 - -44.5)	1,065.9 (817.1 - 1,415.4)	96,455 (63,648 - 142,055)	-57.8 (-74.5 - -36.1)	789.2 (549.4 - 1,115.1)	22,786 (16,566 - 32,679)	-61.7 (-75.0 - -46.0)	247.3 (198.4 - 317.6)	2,728 (2,017 - 3,673)	-75.3 (-83.4 - -65.5)	29.4 (21.9 - 38.8)

Somalia	227,477 (158,462 - 333,213)	-41.8 (-61.3 - -18.1)	1,475.0 (1,098.5 - 2,045.2)	149,874 (99,395 - 228,673)	-46.9 (-65.4 - -20.6)	844.7 (594.5 - 1,192.4)	62,407 (36,778 - 118,114)	-32.3 (-67.8 - 30.8)	528.0 (351.7 - 898.8)	15,196 (10,684 - 21,782)	-9.5 (-39.8 - 31.6)	102.3 (74.7 - 138.6)
South Sudan	224,282 (153,548 - 332,276)	-19.2 (-49.8 - 20.2)	2,042.2 (1,460.5 - 2,889.1)	164,160 (105,663 - 265,293)	-19.6 (-52.8 - 28.1)	1,329.2 (899.4 - 2,040.6)	48,708 (27,364 - 96,575)	-19.8 (-65.9 - 71.0)	595.2 (349.0 - 1,153.0)	11,413 (7,631 - 17,552)	55.9 (-3.8 - 153.0)	117.8 (82.9 - 170.6)
Tanzania	586,334 (446,736 - 765,326)	-46.7 (-62.3 - -26.7)	1,101.5 (886.5 - 1,384.9)	417,859 (291,349 - 585,037)	-50.8 (-68.5 - -29.3)	737.2 (546.3 - 993.5)	157,504 (112,450 - 227,955)	-33.7 (-59.3 - 6.5)	340.0 (260.0 - 457.1)	10,971 (8,299 - 14,509)	-28.5 (-51.0 - 7.3)	24.3 (18.8 - 30.7)
Uganda	427,454 (305,233 - 615,929)	-55.5 (-71.0 - -35.7)	1,105.9 (854.7 - 1,477.4)	337,654 (221,195 - 519,629)	-59.7 (-76.7 - -36.6)	801.2 (572.2 - 1,162.6)	82,134 (59,116 - 118,834)	-36.7 (-57.5 - -11.0)	277.0 (221.3 - 352.9)	7,666 (5,918 - 10,003)	-36.4 (-54.0 - -13.5)	27.7 (21.6 - 35.7)
Zambia	177,868 (131,791 - 250,122)	-50.6 (-64.7 - -31.8)	1,137.1 (914.4 - 1,478.0)	140,204 (97,402 - 207,068)	-50.2 (-67.9 - -26.2)	826.2 (617.3 - 1,170.9)	34,135 (24,699 - 46,704)	-50.9 (-67.1 - -31.5)	281.6 (230.4 - 343.0)	3,529 (2,745 - 4,480)	-41.9 (-58.8 - -18.4)	29.3 (23.3 - 36.8)
Southern sub-Saharan Africa	650,327 (495,568 - 907,168)	-27.5 (-55.6 - 7.1)	910.5 (708.7 - 1,256.8)	501,192 (352,108 - 758,376)	-25.5 (-61.1 - 21.1)	682.2 (484.6 - 1,019.1)	129,399 (103,735 - 166,278)	-28.1 (-48.7 - -3.4)	202.0 (164.2 - 251.5)	19,736 (14,980 - 26,162)	-40.0 (-60.2 - -16.6)	26.4 (20.3 - 34.5)
Botswana	16,938 (12,078 - 25,837)	-33.5 (-60.2 - 4.1)	860.1 (630.5 - 1,270.2)	13,730 (8,885 - 22,311)	-33.0 (-64.5 - 16.2)	667.4 (444.7 - 1,074.7)	2,851 (2,134 - 3,778)	-32.4 (-51.9 - -8.1)	175.5 (136.9 - 224.1)	358 (256 - 489)	-37.0 (-59.5 - -9.9)	17.2 (12.6 - 23.1)
Lesotho	25,200 (18,867 - 35,297)	-4.9 (-40.0 - 39.9)	1,483.9 (1,151.7 - 2,000.4)	19,063 (13,294 - 28,797)	-2.4 (-47.4 - 58.3)	1,066.0 (769.3 - 1,568.9)	5,272 (3,953 - 7,220)	-9.5 (-37.8 - 30.0)	370.0 (282.4 - 481.6)	865 (634 - 1,179)	19.4 (-22.4 - 72.8)	47.9 (35.5 - 64.4)
Namibia	19,893 (14,192 - 29,668)	-29.2 (-55.0 - 8.4)	931.7 (691.4 - 1,348.1)	15,584 (9,996 - 25,475)	-20.0 (-57.1 - 37.7)	701.5 (473.1 - 1,104.2)	3,812 (2,844 - 5,155)	-46.1 (-61.9 - -26.6)	207.1 (162.4 - 263.5)	497 (353 - 700)	-42.0 (-60.8 - -18.7)	23.1 (16.8 - 32.2)
South Africa	433,066 (323,827 - 610,601)	-33.5 (-62.2 - 4.0)	848.0 (641.6 - 1,197.7)	338,483 (228,357 - 523,622)	-30.7 (-66.7 - 19.5)	650.6 (444.6 - 1,011.0)	81,024 (64,137 - 104,995)	-35.6 (-54.6 - -12.2)	172.9 (138.8 - 218.7)	13,559 (9,930 - 18,595)	-50.6 (-68.4 - -29.2)	24.5 (18.2 - 33.2)
Swaziland (eSwatini)	11,897 (8,409 - 18,000)	-9.3 (-43.1 - 37.2)	1,225.2 (906.1 - 1,762.1)	9,530 (6,220 - 15,576)	-1.8 (-46.5 - 66.5)	910.4 (619.6 - 1,440.1)	1,983 (1,481 - 2,631)	-24.7 (-46.6 - 5.1)	277.4 (211.0 - 361.4)	384 (266 - 534)	-8.7 (-40.7 - 34.1)	37.4 (26.6 - 51.0)
Zimbabwe	143,333 (109,922 - 186,456)	-10.8 (-36.1 - 19.6)	1,118.1 (903.0 - 1,399.3)	104,803 (75,296 - 147,742)	-16.4 (-45.0 - 22.0)	765.0 (584.6 - 1,021.4)	34,458 (25,428 - 48,695)	6.0 (-28.1 - 47.3)	317.9 (252.9 - 415.6)	4,073 (3,183 - 5,214)	26.3 (-10.4 - 72.9)	35.2 (27.6 - 44.6)
Western sub-Saharan Africa	9,467,596 (6,579,715 - 13,529,599)	-32.9 (-57.0 - -1.1)	1,874.2 (1,375.1 - 2,547.0)	7,709,875 (5,004,337 - 11,789,161)	-32.0 (-61.2 - 11.1)	1,427.4 (971.2 - 2,094.6)	1,640,635 (1,109,058 - 2,527,368)	-32.9 (-61.6 - 6.2)	416.6 (296.3 - 632.0)	117,087 (91,625 - 145,911)	-27.1 (-45.9 - -4.8)	30.2 (24.1 - 36.9)
Benin	191,618 (131,435 - 281,788)	-33.4 (-57.3 - -1.1)	1,573.0 (1,149.8 - 2,180.4)	154,791 (98,266 - 242,478)	-26.9 (-58.5 - 19.8)	1,200.3 (795.2 - 1,784.4)	33,458 (23,718 - 48,741)	-47.5 (-65.7 - -22.9)	335.3 (258.9 - 447.6)	3,369 (2,416 - 4,608)	-34.1 (-53.2 - -8.9)	37.4 (27.4 - 50.5)
Burkina Faso	470,981 (309,661 - 724,673)	-31.0 (-55.8 - 4.4)	1,892.2 (1,358.7 - 2,667.1)	403,201 (248,496 - 652,562)	-29.0 (-58.9 - 15.9)	1,512.7 (998.6 - 2,277.2)	60,971 (43,625 - 86,282)	-36.8 (-60.8 - -8.5)	341.5 (267.0 - 447.5)	6,809 (5,169 - 9,049)	-24.5 (-45.9 - 0.2)	38.0 (29.8 - 47.5)
Cameroon	411,831 (284,250 - 621,754)	-33.5 (-56.8 - -0.1)	1,560.8 (1,160.0 - 2,193.5)	319,809 (201,033 - 525,507)	-31.8 (-61.1 - 14.1)	1,133.8 (766.5 - 1,723.1)	85,022 (57,761 - 133,777)	-36.2 (-61.2 - 0.5)	394.1 (288.9 - 580.2)	7,001 (5,138 - 9,051)	-22.2 (-45.3 - 5.3)	32.8 (24.7 - 42.3)
Cape Verde	2,288 (1,739 - 3,048)	-55.7 (-73.8 - -32.7)	467.7 (354.4 - 631.1)	1,484 (1,032 - 2,153)	-61.6 (-80.8 - -34.9)	302.5 (209.7 - 445.5)	736 (551 - 1,029)	-35.9 (-58.4 - -2.9)	152.0 (113.0 - 214.1)	68 (51 - 89)	-19.0 (-43.7 - 12.0)	13.1 (9.9 - 17.1)
Chad	404,810 (272,860 - 636,080)	-23.3 (-51.8 - 16.5)	2,198.6 (1,585.1 - 3,136.2)	317,470 (194,776 - 547,788)	-21.7 (-55.2 - 29.7)	1,535.2 (1,027.9 - 2,413.1)	81,962 (50,481 - 152,609)	-21.4 (-65.9 - 50.4)	623.3 (393.1 - 1,185.8)	5,378 (4,045 - 6,884)	-27.3 (-46.8 - -3.7)	40.1 (31.4 - 50.8)
Côte d'Ivoire	350,360 (256,040 - 492,404)	-31.6 (-53.3 - -3.0)	1,415.4 (1,103.7 - 1,864.8)	261,580 (175,234 - 398,906)	-32.8 (-59.1 - 2.6)	994.4 (709.8 - 1,430.4)	82,283 (58,025 - 120,059)	-26.7 (-53.8 - 10.2)	387.0 (295.4 - 535.4)	6,496 (4,933 - 8,356)	-19.6 (-40.8 - 4.6)	34.0 (26.4 - 41.9)
The Gambia	26,269 (18,774 - 35,612)	-38.1 (-58.4 - -10.2)	1,364.7 (1,023.4 - 1,833.5)	17,167 (11,057 - 25,347)	-42.7 (-64.8 - -10.6)	854.4 (595.7 - 1,223.0)	8,652 (5,729 - 13,281)	-25.4 (-58.6 - 22.1)	481.3 (339.6 - 716.9)	450 (318 - 612)	-15.5 (-41.2 - 17.9)	29.0 (21.4 - 38.6)

Ghana	363,585 (258,535 - 518,000)	-32.0 (-55.0 - 1.2)	1,274.6 (957.0 - 1,738.4)	291,711 (186,774 - 449,645)	-30.8 (-59.2 - 11.3)	983.1 (677.0 - 1,444.0)	64,539 (47,802 - 93,311)	-35.1 (-58.4 - -5.7)	260.5 (207.9 - 334.2)	7,335 (5,453 - 9,549)	-12.1 (-38.2 - 22.9)	31.0 (23.7 - 39.4)
Guinea	210,333 (153,162 - 290,140)	-37.4 (-57.6 - -12.7)	1,733.8 (1,340.3 - 2,275.1)	153,988 (103,239 - 237,295)	-35.3 (-59.0 - -2.8)	1,166.4 (829.0 - 1,680.7)	53,035 (35,991 - 84,653)	-39.1 (-69.2 - 3.3)	532.7 (380.3 - 831.9)	3,311 (2,523 - 4,227)	-26.5 (-45.1 - -1.5)	34.7 (27.2 - 43.5)
Guinea-Bissau	23,375 (16,935 - 33,683)	-44.7 (-61.2 - -23.4)	1,395.3 (1,105.4 - 1,848.5)	16,402 (10,988 - 25,807)	-43.6 (-63.7 - -14.3)	918.2 (673.8 - 1,330.4)	6,434 (4,470 - 9,323)	-45.1 (-67.0 - -17.2)	437.3 (332.5 - 580.8)	539 (409 - 705)	-39.8 (-56.4 - -18.6)	39.8 (31.0 - 50.2)
Liberia	70,230 (49,445 - 99,801)	-56.0 (-71.1 - -35.5)	1,552.3 (1,152.2 - 2,087.2)	53,516 (34,890 - 82,693)	-49.7 (-70.7 - -20.2)	1,123.8 (770.8 - 1,636.9)	15,903 (10,489 - 25,218)	-53.1 (-77.7 - -18.7)	405.6 (287.7 - 624.6)	810 (592 - 1,100)	-93.5 (-96.1 - -90.2)	23.0 (17.3 - 29.8)
Mali	568,495 (381,062 - 864,704)	-34.7 (-58.8 - 2.5)	2,219.1 (1,588.8 - 3,133.9)	458,818 (275,247 - 748,427)	-24.6 (-58.3 - 26.3)	1,684.3 (1,074.3 - 2,610.8)	101,115 (67,399 - 156,090)	-52.7 (-76.1 - -19.4)	494.5 (352.2 - 727.7)	8,562 (6,392 - 11,344)	-30.6 (-48.9 - -7.4)	40.3 (30.9 - 51.5)
Mauritania	46,747 (33,473 - 68,063)	-47.4 (-67.2 - -20.6)	1,285.7 (946.1 - 1,814.7)	32,867 (20,300 - 52,264)	-43.7 (-70.2 - -2.7)	861.9 (550.4 - 1,327.1)	13,096 (8,892 - 19,144)	-51.5 (-74.6 - -21.8)	399.6 (284.6 - 575.5)	784 (580 - 1,059)	-44.1 (-60.9 - -20.7)	24.2 (18.2 - 32.3)
Niger	568,605 (372,044 - 876,162)	-38.4 (-62.6 - -4.2)	2,223.9 (1,541.1 - 3,156.1)	506,854 (312,417 - 806,810)	-36.2 (-65.0 - 7.0)	1,864.9 (1,203.3 - 2,767.6)	55,625 (37,026 - 86,755)	-45.1 (-70.1 - -9.3)	326.7 (237.3 - 481.2)	6,127 (4,567 - 8,008)	-41.2 (-58.1 - -19.6)	32.3 (24.6 - 41.5)
Nigeria	5,333,767 (3,474,576 - 8,085,457)	-32.9 (-60.2 - 2.1)	2,068.5 (1,417.6 - 2,995.7)	4,394,358 (2,647,587 - 6,949,779)	-34.3 (-65.3 - 10.9)	1,591.2 (1,007.7 - 2,428.9)	886,118 (528,768 - 1,522,138)	-23.6 (-61.4 - 35.6)	451.0 (268.5 - 775.1)	53,291 (38,406 - 71,625)	-14.0 (-40.2 - 21.0)	26.3 (19.8 - 34.6)
São Tomé and Príncipe	1,150 (906 - 1,465)	-50.6 (-64.8 - -33.7)	720.6 (590.6 - 876.5)	729 (523 - 1,000)	-57.4 (-72.9 - -35.6)	434.9 (331.1 - 575.7)	389 (299 - 512)	-33.5 (-55.0 - -7.0)	265.9 (213.4 - 331.9)	32 (24 - 43)	-17.5 (-45.1 - 19.0)	19.9 (14.6 - 26.9)
Senegal	144,634 (106,245 - 202,477)	-53.8 (-69.7 - -33.6)	1,071.6 (828.7 - 1,420.4)	100,944 (66,851 - 155,555)	-55.8 (-74.2 - -28.7)	700.1 (492.5 - 1,028.7)	40,601 (29,860 - 57,088)	-48.4 (-68.1 - -23.2)	344.6 (269.1 - 454.5)	3,088 (2,291 - 4,196)	-28.5 (-48.5 - -2.3)	26.9 (20.4 - 35.7)
Sierra Leone	176,933 (119,166 - 273,868)	-32.4 (-58.7 - 4.4)	2,110.2 (1,524.0 - 3,033.7)	141,138 (86,457 - 239,051)	-27.6 (-61.0 - 20.9)	1,607.0 (1,050.9 - 2,534.5)	33,682 (23,405 - 51,856)	-41.3 (-69.3 - -2.0)	471.1 (350.8 - 682.4)	2,112 (1,592 - 2,778)	-27.0 (-49.4 - 2.5)	32.1 (24.9 - 41.4)
Togo	101,491 (70,537 - 150,056)	-34.5 (-57.6 - 0.4)	1,424.4 (1,059.7 - 2,003.1)	82,971 (52,599 - 134,991)	-29.6 (-59.7 - 17.0)	1,108.0 (748.5 - 1,705.4)	16,998 (11,945 - 24,174)	-46.8 (-66.0 - -24.1)	289.3 (224.6 - 378.5)	1,522 (1,152 - 1,970)	-26.5 (-46.8 - -0.2)	27.1 (20.6 - 34.1)

Abbreviations: DR=Democratic Republic. UI=uncertainty interval. USA=United States of America.

eTable 10. Global sepsis-related mortality and incidence by underlying cause for all ages and both sexes, 2017

Cause	Sepsis incidence				Sepsis-related mortality				
	**Sepsis incidence, no. (95% UI)**	**Percentage of age-standardised sepsis cases due to cause (95% UI)**	**Age-standardised sepsis incidence rate per 100,000 (95% UI)**	**Percentage change in age-standardised sepsis incidence rate, 1990-2017**	**Sepsis-related deaths, no. (95% UI)**	**Percentage of age-standardised cause deaths via sepsis (95% UI)**	**Percentage of age-standardised sepsis deaths due to cause (95% UI)**	**Sepsis-related age-standardised mortality rate per 100,000 (95% UI)**	**Percentage change in sepsis-related age-standardised mortality rate, 1990-2017**
HIV/AIDS	745,492 (354,311 - 1,341,261)	1.4 (0.6 - 2.7)	9.5 (4.5 - 17.1)	97.3 (-26.2 - 322.8)	398,695 (223,600 - 574,164)	41.8 (23.4 - 60.5)	3.4 (1.9 - 4.9)	5.1 (2.9 - 7.3)	53.4 (-22.5 - 168.0)
Sexually transmitted infections excluding HIV	173,646 (59,442 - 425,426)	0.4 (0.1 - 0.9)	2.4 (0.8 - 6.0)	9.5 (-75.5 - 224.3)	104,450 (42,979 - 188,034)	87.6 (76.7 - 94.8)	1.1 (0.4 - 2.0)	1.6 (0.6 - 2.8)	-24.6 (-37.3 - -9.9)
Tuberculosis	718,582 (330,445 - 1,433,619)	1.4 (0.6 - 2.8)	9.1 (4.2 - 18.3)	-50.5 (-84.5 - 26.1)	390,967 (200,285 - 653,896)	32.9 (17.3 - 54.7)	3.3 (1.7 - 5.4)	4.9 (2.5 - 8.2)	-62.0 (-85.7 - -15.0)
Lower respiratory infections	6,114,110 (3,498,903 - 11,130,544)	12.8 (7.2 - 21.8)	86.7 (49.3 - 158.8)	-37.6 (-73.6 - 25.3)	1,762,055 (1,307,597 - 2,117,334)	68.8 (52.4 - 82.5)	16.4 (12.8 - 19.4)	24.4 (18.1 - 29.3)	-53.4 (-65.9 - -39.6)
Upper respiratory infections	81,507 (27,667 - 170,109)	0.2 (0.1 - 0.4)	1.2 (0.4 - 2.5)	-64.6 (-90.5 - -9.0)	4,918 (2,215 - 8,034)	53.5 (32.9 - 71.7)	0.0 (0.0 - 0.1)	0.1 (0.0 - 0.1)	-81.0 (-89.1 - -68.0)
Otitis media	3,169 (1,361 - 6,822)	0.0 (0.0 - 0.0)	0.0 (0.0 - 0.1)	-75.8 (-93.1 - -39.3)	624 (387 - 1,019)	65.6 (48.5 - 79.9)	0.0 (0.0 - 0.0)	0.0 (0.0 - 0.0)	-81.4 (-88.0 - -71.7)
Diarrhoeal diseases	9,214,180 (3,563,782 - 20,937,368)	18.9 (8.4 - 36.2)	132.1 (50.8 - 301.8)	-38.4 (-83.7 - 71.7)	1,288,467 (900,028 - 1,846,888)	82.1 (62.5 - 94.3)	11.9 (8.6 - 16.3)	17.7 (12.6 - 25.1)	-59.3 (-70.6 - -44.8)
Typhoid and paratyphoid	2,662,565 (948,871 - 6,015,597)	5.5 (2.1 - 11.9)	37.7 (13.4 - 85.5)	-4.1 (-72.2 - 140.1)	105,116 (58,041 - 172,160)	77.4 (62.7 - 88.7)	1.0 (0.5 - 1.6)	1.5 (0.8 - 2.4)	-56.0 (-66.5 - -44.8)
Invasive non-typhoidal salmonella	414,810 (154,828 - 953,066)	0.9 (0.3 - 2.1)	6.0 (2.2 - 13.9)	94.5 (-34.6 - 368.9)	45,177 (24,495 - 76,954)	76.5 (61.1 - 88.1)	0.4 (0.2 - 0.7)	0.6 (0.3 - 1.1)	2.6 (-23.4 - 33.3)
Other intestinal infectious diseases					629 (302 - 1,152)	43.5 (24.5 - 62.9)	0.0 (0.0 - 0.0)	0.0 (0.0 - 0.0)	-77.4 (-91.2 - -51.2)
Malaria	3,374,871 (1,029,771 - 8,423,870)	7.2 (2.3 - 17.5)	49.0 (14.9 - 123.4)	30.4 (-75.2 - 316.1)	392,423 (212,638 - 603,702)	63.3 (38.6 - 83.7)	3.7 (2.0 - 5.7)	5.5 (3.0 - 8.5)	-32.1 (-62.5 - 8.9)
Chagas disease	708 (224 - 1,759)	0.0 (0.0 - 0.0)	0.0 (0.0 - 0.0)	-59.2 (-91.7 - 33.5)	458 (157 - 1,056)	5.8 (2.0 - 13.3)	0.0 (0.0 - 0.0)	0.0 (0.0 - 0.0)	-65.9 (-92.4 - 0.3)
Leishmaniasis	10,415 (5 - 60,865)	0.0 (0.0 - 0.1)	0.1 (0.0 - 0.9)	-98.1 (-99.5 - -94.2)	3,854 (3 - 18,029)	52.1 (27.5 - 73.9)	0.0 (0.0 - 0.2)	0.1 (0.0 - 0.3)	-98.8 (-99.6 - -97.3)
African trypanosomiasis	2,180 (235 - 10,344)	0.0 (0.0 - 0.0)	0.0 (0.0 - 0.1)	-90.7 (-99.4 - -57.6)	671 (104 - 2,592)	49.3 (22.4 - 75.5)	0.0 (0.0 - 0.0)	0.0 (0.0 - 0.0)	-94.5 (-99.2 - -78.0)
Schistosomiasis					1,517 (565 - 3,093)	17.1 (6.3 - 34.2)	0.0 (0.0 - 0.0)	0.0 (0.0 - 0.0)	-66.7 (-91.2 - -1.6)
Cysticercosis					173 (64 - 356)	23.1 (9.4 - 43.7)	0.0 (0.0 - 0.0)	0.0 (0.0 - 0.0)	-62.4 (-89.1 - -7.6)
Cystic echinococcosis	3,433 (1,156 - 8,506)	0.0 (0.0 - 0.0)	0.0 (0.0 - 0.1)	-61.4 (-91.5 - 16.1)	758 (425 - 1,095)	63.3 (41.0 - 83.4)	0.0 (0.0 - 0.0)	0.0 (0.0 - 0.0)	-76.4 (-87.6 - -61.0)
Dengue	644,105 (217,789 - 1,420,143)	1.3 (0.4 - 2.9)	8.9 (3.0 - 19.8)	194.8 (-43.2 - 789.6)	30,556 (12,224 - 40,273)	75.4 (59.0 - 87.3)	0.3 (0.1 - 0.4)	0.4 (0.2 - 0.5)	73.5 (-30.3 - 196.1)
Yellow fever	10,643 (1,027 - 45,109)	0.0 (0.0 - 0.1)	0.1 (0.0 - 0.6)	-44.6 (-93.8 - 123.1)	2,001 (357 - 6,360)	42.1 (21.3 - 66.2)	0.0 (0.0 - 0.1)	0.0 (0.0 - 0.1)	-72.9 (-89.0 - -46.2)

Rabies	21,297 (4,337 - 77,416)	0.0 (0.0 - 0.2)	0.3 (0.1 - 1.1)	-63.3 (-96.6 - 65.8)	3,893 (1,588 - 7,012)	33.4 (14.5 - 56.3)	0.0 (0.0 - 0.1)	0.1 (0.0 - 0.1)	-82.8 (-94.2 - - 61.3)
Intestinal nematode infections					1,210 (543 - 2,064)	37.7 (18.2 - 62.5)	0.0 (0.0 - 0.0)	0.0 (0.0 - 0.0)	-77.7 (-91.3 - - 51.5)
Ebola virus disease	29 (16 - 51)	0.0 (0.0 - 0.0)	0.0 (0.0 - 0.0)	-	7 (5 - 8)	90.8 (67.3 - 99.5)	0.0 (0.0 - 0.0)	0.0 (0.0 - 0.0)	-
Zika virus disease	66 (2 - 406)	0.0 (0.0 - 0.0)	0.0 (0.0 - 0.0)	-	5 (0 - 18)	26.6 (3.8 - 70.5)	0.0 (0.0 - 0.0)	0.0 (0.0 - 0.0)	-
Other neglected tropical diseases					9,248 (4,732 - 27,955)	73.2 (52.7 - 88.9)	0.1 (0.0 - 0.3)	0.1 (0.1 - 0.4)	-27.6 (-56.0 - 16.8)
Meningitis	723,883 (465,365 - 1,136,378)	1.6 (1.0 - 2.5)	10.4 (6.7 - 16.5)	-35.4 (-65.9 - 12.0)	184,976 (148,238 - 227,536)	64.4 (53.9 - 73.2)	1.8 (1.4 - 2.2)	2.6 (2.1 - 3.2)	-53.5 (-64.1 - - 39.4)
Encephalitis	236,221 (99,605 - 503,526)	0.5 (0.2 - 1.1)	3.3 (1.4 - 7.1)	-0.4 (-68.7 - 150.3)	58,006 (38,336 - 77,518)	62.8 (41.5 - 79.9)	0.5 (0.3 - 0.7)	0.8 (0.5 - 1.0)	-39.4 (-66.5 - - 2.9)
Diphtheria	6,119 (2,624 - 12,865)	0.0 (0.0 - 0.0)	0.1 (0.0 - 0.2)	-75.1 (-91.4 - - 43.3)	2,698 (1,485 - 4,694)	65.3 (47.7 - 77.6)	0.0 (0.0 - 0.0)	0.0 (0.0 - 0.1)	-82.6 (-91.0 - - 68.0)
Whooping cough	170,453 (55,392 - 394,601)	0.4 (0.1 - 0.9)	2.5 (0.8 - 5.9)	-26.0 (-84.2 - 117.0)	55,740 (23,024 - 108,855)	53.1 (30.9 - 72.0)	0.6 (0.2 - 1.1)	0.8 (0.3 - 1.6)	-48.3 (-79.7 - 7.5)
Tetanus	53,552 (17,500 - 117,513)	0.1 (0.0 - 0.3)	0.8 (0.2 - 1.7)	-88.3 (-97.3 - - 67.9)	15,620 (7,038 - 27,979)	41.1 (19.5 - 67.2)	0.1 (0.1 - 0.3)	0.2 (0.1 - 0.4)	-91.7 (-96.8 - - 82.2)
Measles	524,058 (117,226 - 1,502,979)	1.2 (0.3 - 3.4)	7.8 (1.7 - 22.4)	-78.3 (-94.4 - - 44.4)	82,855 (29,283 - 180,031)	76.0 (62.5 - 83.9)	0.8 (0.3 - 1.8)	1.2 (0.4 - 2.7)	-87.9 (-91.1 - - 84.2)
Varicella and herpes zoster	118,274 (52,287 - 239,206)	0.3 (0.1 - 0.5)	1.7 (0.8 - 3.5)	-20.5 (-75.5 - 77.1)	10,870 (8,276 - 13,316)	69.7 (53.4 - 83.5)	0.1 (0.1 - 0.1)	0.2 (0.1 - 0.2)	-58.8 (-70.1 - - 45.8)
Acute hepatitis	71,607 (34,325 - 133,533)	0.1 (0.1 - 0.3)	0.9 (0.5 - 1.8)	-44.5 (-77.5 - 18.0)	31,113 (16,794 - 50,785)	24.6 (14.0 - 38.5)	0.3 (0.1 - 0.4)	0.4 (0.2 - 0.6)	-58.1 (-79.0 - - 25.0)
Other unspecified infectious diseases					51,863 (34,907 - 65,555)	65.7 (48.6 - 80.3)	0.5 (0.3 - 0.6)	0.7 (0.5 - 0.9)	-45.8 (-61.9 - - 27.0)
Maternal disorders	5,655,987 (3,389,024 - 9,153,516)	10.8 (6.3 - 17.0)	72.7 (43.5 - 117.5)	-48.4 (-77.2 - - 0.7)	74,665 (63,113 - 87,367)	24.5 (21.0 - 28.2)	0.6 (0.5 - 0.8)	1.0 (0.8 - 1.1)	-60.5 (-69.0 - - 50.4)
Neonatal disorders	5,145,449 (2,917,740 - 8,864,110)	11.6 (6.5 - 19.6)	78.0 (44.2 - 134.4)	-6.4 (-58.7 - 85.4)	801,615 (627,191 - 996,840)	4.6 (3.6 - 5.7)	8.2 (6.4 - 10.2)	12.1 (9.5 - 15.1)	-49.6 (-63.1 - - 30.9)
Protein-energy malnutrition	176,617 (26,876 - 512,755)	0.4 (0.1 - 1.2)	2.6 (0.4 - 7.4)	-44.2 (-95.4 - 162.9)	77,819 (16,511 - 169,877)	33.6 (7.3 - 72.4)	0.8 (0.2 - 1.7)	1.1 (0.2 - 2.4)	-59.8 (-94.0 - 59.7)
Other nutritional deficiencies					8,013 (1,315 - 19,990)	20.7 (3.3 - 53.4)	0.1 (0.0 - 0.2)	0.1 (0.0 - 0.3)	-55.2 (-96.2 - 98.5)
Lip and oral cavity cancer	26,720 (13,276 - 47,429)	0.0 (0.0 - 0.1)	0.3 (0.2 - 0.6)	-12.3 (-68.3 - 83.5)	17,965 (9,359 - 30,493)	6.5 (3.4 - 11.0)	0.2 (0.1 - 0.3)	0.2 (0.1 - 0.4)	-25.6 (-70.4 - 56.4)
Nasopharynx cancer	12,034 (5,499 - 23,027)	0.0 (0.0 - 0.0)	0.1 (0.1 - 0.3)	-41.0 (-80.9 - 41.5)	6,813 (3,339 - 11,668)	8.8 (4.4 - 15.0)	0.1 (0.0 - 0.1)	0.1 (0.0 - 0.1)	-55.0 (-83.1 - - 4.9)
Other pharynx cancer	16,677 (7,309 - 30,329)	0.0 (0.0 - 0.1)	0.2 (0.1 - 0.4)	-8.8 (-67.3 - 111.3)	11,229 (5,264 - 19,954)	6.8 (3.3 - 11.7)	0.1 (0.0 - 0.2)	0.1 (0.1 - 0.2)	-22.1 (-69.6 - 70.2)
Oesophageal cancer	49,646 (23,768 - 93,715)	0.1 (0.0 - 0.2)	0.6 (0.3 - 1.2)	-47.7 (-80.5 - 15.6)	29,177 (15,044 - 52,439)	4.7 (2.4 - 8.4)	0.2 (0.1 - 0.4)	0.4 (0.2 - 0.7)	-60.3 (-83.4 - - 13.0)
Stomach cancer	103,307 (46,671 - 202,799)	0.2 (0.1 - 0.4)	1.3 (0.6 - 2.5)	-55.8 (-84.2 - 2.6)	58,108 (28,580 - 103,696)	4.7 (2.3 - 8.5)	0.5 (0.2 - 0.9)	0.7 (0.4 - 1.3)	-66.1 (-86.9 - - 27.5)
Colon and rectum cancer	134,967 (62,489 - 281,563)	0.3 (0.1 - 0.6)	1.7 (0.8 - 3.5)	-18.4 (-72.6 - 89.9)	65,858 (34,709 - 115,140)	5.2 (2.7 - 9.0)	0.6 (0.3 - 1.0)	0.8 (0.4 - 1.5)	-37.0 (-74.4 - 31.8)
Liver cancer	115,640 (85,297 - 157,856)	0.2 (0.1 - 0.3)	1.4 (1.1 - 1.9)	-33.6 (-56.4 - - 1.9)	57,932 (45,103 - 72,673)	6.3 (4.9 - 7.9)	0.5 (0.4 - 0.6)	0.7 (0.6 - 0.9)	-53.1 (-66.3 - - 35.1)
Gallbladder and biliary tract cancer	32,175 (14,384 - 60,218)	0.1 (0.0 - 0.1)	0.4 (0.2 - 0.8)	-26.2 (-73.1 - 61.9)	17,437 (9,107 - 29,429)	7.0 (3.7 - 11.8)	0.1 (0.1 - 0.3)	0.2 (0.1 - 0.4)	-40.1 (-75.8 - 24.0)
Pancreatic cancer	40,209 (18,074 - 80,105)	0.1 (0.0 - 0.2)	0.5 (0.2 - 1.0)	-2.1 (-68.1 - 131.5)	20,956 (10,290 - 37,704)	3.3 (1.6 - 5.9)	0.2 (0.1 - 0.3)	0.3 (0.1 - 0.5)	-23.4 (-70.8 - 64.5)
Larynx cancer	17,387 (8,139 - 32,061)	0.0 (0.0 - 0.1)	0.2 (0.1 - 0.4)	-39.9 (-77.4 - 40.1)	10,757 (5,351 - 18,219)	6.0 (3.0 - 10.2)	0.1 (0.0 - 0.2)	0.1 (0.1 - 0.2)	-50.5 (-81.3 - 8.5)
Tracheal, bronchus, and lung cancer	90,228 (38,931 - 168,157)	0.2 (0.1 - 0.3)	1.1 (0.5 - 2.1)	-29.8 (-78.4 - 75.9)	56,047 (26,061 - 101,645)	2.1 (1.0 - 3.9)	0.5 (0.2 - 0.8)	0.7 (0.3 - 1.3)	-43.8 (-81.7 - 24.2)

Malignant skin melanoma	3,099 (1,351 - 6,474)	0.0 (0.0 - 0.0)	0.0 (0.0 - 0.1)	-18.5 (-74.5 - 104.0)	1,698 (801 - 3,233)	1.9 (0.9 - 3.5)	0.0 (0.0 - 0.0)	0.0 (0.0 - 0.0)	-34.5 (-76.3 - 54.4)
Non-melanoma skin cancer	10,986 (4,678 - 22,911)	0.0 (0.0 - 0.0)	0.1 (0.1 - 0.3)	-8.9 (-71.2 - 128.5)	5,083 (2,539 - 8,907)	5.4 (2.7 - 9.5)	0.0 (0.0 - 0.1)	0.1 (0.0 - 0.1)	-33.4 (-74.7 - 43.0)
Breast cancer	56,655 (25,436 - 111,361)	0.1 (0.0 - 0.2)	0.7 (0.3 - 1.4)	-15.6 (-74.4 - 94.5)	33,882 (16,208 - 61,923)	3.9 (1.9 - 7.1)	0.3 (0.1 - 0.5)	0.4 (0.2 - 0.8)	-28.3 (-75.1 - 54.0)
Cervical cancer	46,859 (22,076 - 86,708)	0.1 (0.0 - 0.2)	0.6 (0.3 - 1.1)	-37.8 (-77.5 - 40.0)	28,974 (14,683 - 49,837)	7.9 (4.0 - 13.5)	0.2 (0.1 - 0.4)	0.4 (0.2 - 0.6)	-49.6 (-80.6 - 3.3)
Uterine cancer	12,220 (5,471 - 23,915)	0.0 (0.0 - 0.0)	0.2 (0.1 - 0.3)	-36.3 (-79.1 - 52.0)	6,367 (3,200 - 11,012)	5.2 (2.6 - 9.1)	0.1 (0.0 - 0.1)	0.1 (0.0 - 0.1)	-50.7 (-79.5 - 6.6)
Ovarian cancer	22,933 (10,169 - 45,130)	0.0 (0.0 - 0.1)	0.3 (0.1 - 0.6)	-5.3 (-70.9 - 127.9)	12,016 (5,966 - 20,800)	4.8 (2.3 - 8.4)	0.1 (0.0 - 0.2)	0.1 (0.1 - 0.3)	-20.8 (-70.1 - 71.5)
Prostate cancer	48,993 (22,749 - 93,434)	0.1 (0.0 - 0.2)	0.6 (0.3 - 1.2)	-17.7 (-72.6 - 103.9)	30,038 (14,867 - 51,521)	5.1 (2.5 - 8.7)	0.3 (0.1 - 0.5)	0.4 (0.2 - 0.7)	-30.3 (-73.2 - 56.3)
Testicular cancer	1,810 (818 - 3,349)	0.0 (0.0 - 0.0)	0.0 (0.0 - 0.0)	-35.2 (-78.3 - 51.0)	1,016 (504 - 1,725)	9.4 (4.8 - 15.9)	0.0 (0.0 - 0.0)	0.0 (0.0 - 0.0)	-48.3 (-79.3 - 7.0)
Kidney cancer	13,124 (5,467 - 27,456)	0.0 (0.0 - 0.1)	0.2 (0.1 - 0.3)	-2.4 (-70.7 - 157.2)	5,868 (2,804 - 10,402)	4.2 (2.0 - 7.4)	0.1 (0.0 - 0.1)	0.1 (0.0 - 0.1)	-25.7 (-71.1 - 73.7)
Bladder cancer	35,301 (16,290 - 75,722)	0.1 (0.0 - 0.1)	0.5 (0.2 - 1.0)	-25.1 (-74.4 - 83.7)	16,832 (8,609 - 30,484)	6.0 (3.1 - 10.8)	0.1 (0.1 - 0.3)	0.2 (0.1 - 0.4)	-42.6 (-76.6 - 25.8)
Brain and nervous system cancer	26,811 (11,355 - 53,994)	0.1 (0.0 - 0.1)	0.3 (0.1 - 0.7)	-12.7 (-73.5 - 114.8)	13,732 (6,405 - 23,882)	5.6 (2.6 - 9.6)	0.1 (0.1 - 0.2)	0.2 (0.1 - 0.3)	-33.2 (-76.3 - 40.6)
Thyroid cancer	4,481 (2,062 - 8,215)	0.0 (0.0 - 0.0)	0.1 (0.0 - 0.1)	-21.9 (-72.6 - 76.3)	2,780 (1,397 - 4,730)	5.3 (2.7 - 9.1)	0.0 (0.0 - 0.0)	0.0 (0.0 - 0.1)	-35.8 (-75.8 - 39.9)
Mesothelioma	2,377 (997 - 4,843)	0.0 (0.0 - 0.0)	0.0 (0.0 - 0.1)	-21.9 (-75.6 - 93.4)	1,329 (621 - 2,488)	3.1 (1.5 - 5.9)	0.0 (0.0 - 0.0)	0.0 (0.0 - 0.0)	-36.3 (-77.5 - 49.8)
Hodgkin lymphoma	16,512 (5,304 - 37,226)	0.0 (0.0 - 0.1)	0.2 (0.1 - 0.5)	-38.5 (-86.5 - 76.3)	8,373 (3,236 - 15,744)	25.7 (10.0 - 48.1)	0.1 (0.0 - 0.1)	0.1 (0.0 - 0.2)	-53.3 (-85.7 - 11.5)
Non-Hodgkin lymphoma	82,990 (26,281 - 192,575)	0.2 (0.0 - 0.4)	1.1 (0.3 - 2.5)	9.1 (-77.0 - 244.1)	44,008 (16,391 - 85,666)	17.6 (6.5 - 34.4)	0.4 (0.1 - 0.7)	0.6 (0.2 - 1.1)	-13.9 (-76.6 - 138.5)
Multiple myeloma	36,533 (11,194 - 90,429)	0.1 (0.0 - 0.2)	0.5 (0.1 - 1.1)	15.0 (-78.3 - 296.6)	16,831 (5,773 - 34,126)	11.0 (3.9 - 22.3)	0.1 (0.0 - 0.3)	0.2 (0.1 - 0.4)	-12.8 (-78.9 - 130.3)
Leukaemia	163,197 (90,147 - 270,670)	0.3 (0.2 - 0.6)	2.1 (1.2 - 3.6)	-24.2 (-66.2 - 39.8)	77,722 (49,490 - 113,434)	22.3 (14.4 - 32.2)	0.7 (0.4 - 1.0)	1.0 (0.6 - 1.5)	-40.6 (-68.3 - - 1.9)
Other malignant cancers	72,645 (32,052 - 139,268)	0.1 (0.1 - 0.3)	0.9 (0.4 - 1.8)	-14.2 (-70.8 - 94.1)	35,556 (18,426 - 60,656)	9.9 (5.1 - 16.7)	0.3 (0.2 - 0.5)	0.5 (0.2 - 0.8)	-34.4 (-73.9 - 40.9)
Other neoplasms	50,477 (24,197 - 98,582)	0.1 (0.0 - 0.2)	0.7 (0.3 - 1.3)	42.5 (-45.1 - 220.3)	17,860 (10,258 - 29,436)	17.1 (10.2 - 27.0)	0.2 (0.1 - 0.3)	0.2 (0.1 - 0.4)	4.1 (-49.3 - 87.7)
Rheumatic heart disease	56,058 (24,291 - 114,460)	0.1 (0.0 - 0.2)	0.7 (0.3 - 1.5)	-60.6 (-88.0 - - 1.1)	33,676 (15,707 - 63,566)	11.4 (5.4 - 21.6)	0.3 (0.1 - 0.5)	0.4 (0.2 - 0.8)	-67.3 (-89.0 - - 24.7)
Ischaemic heart disease	375,281 (143,655 - 802,235)	0.7 (0.3 - 1.6)	4.8 (1.8 - 10.2)	-31.0 (-82.3 - 92.1)	201,156 (83,017 - 410,582)	1.6 (0.6 - 3.1)	1.7 (0.7 - 3.5)	2.6 (1.1 - 5.3)	-44.8 (-83.2 - 43.9)
Stroke	1,067,730 (583,431 - 1,787,789)	2.0 (1.1 - 3.5)	13.7 (7.4 - 23.0)	-44.6 (-73.9 - 12.8)	597,394 (360,156 - 933,124)	9.5 (5.7 - 15.0)	5.2 (3.2 - 7.9)	7.7 (4.6 - 12.1)	-56.7 (-77.7 - - 21.2)
Hypertensive heart disease					18,216 (7,077 - 39,307)	1.4 (0.5 - 2.9)	0.2 (0.1 - 0.3)	0.2 (0.1 - 0.5)	-51.3 (-88.0 - 33.0)
Non-rheumatic valvular heart disease	11,486 (5,584 - 20,656)	0.0 (0.0 - 0.0)	0.2 (0.1 - 0.3)	-20.3 (-69.1 - 71.7)	7,858 (4,650 - 12,003)	3.7 (2.2 - 5.8)	0.1 (0.0 - 0.1)	0.1 (0.1 - 0.2)	-38.2 (-68.8 - 6.7)
Cardiomyopathy and myocarditis	18,258 (6,974 - 41,294)	0.0 (0.0 - 0.1)	0.2 (0.1 - 0.5)	-4.0 (-74.9 - 154.1)	20,261 (11,741 - 32,790)	5.5 (3.2 - 8.9)	0.2 (0.1 - 0.3)	0.3 (0.2 - 0.4)	-41.2 (-72.7 - 15.6)
Atrial fibrillation and flutter	56,643 (20,275 - 126,108)	0.1 (0.0 - 0.3)	0.8 (0.3 - 1.7)	7.8 (-73.7 - 199.7)	22,679 (9,567 - 43,529)	3.6 (1.5 - 6.9)	0.2 (0.1 - 0.4)	0.3 (0.1 - 0.6)	-24.9 (-76.2 - 89.6)
Aortic aneurysm					6,863 (2,868 - 13,466)	2.9 (1.2 - 5.6)	0.1 (0.0 - 0.1)	0.1 (0.0 - 0.2)	-38.5 (-82.6 - 51.0)
Peripheral vascular disease	48,331 (15,941 - 113,195)	0.1 (0.0 - 0.2)	0.6 (0.2 - 1.5)	71.0 (-56.1 - 351.4)	18,584 (7,799 - 39,734)	8.6 (4.4 - 14.2)	0.2 (0.1 - 0.4)	0.2 (0.1 - 0.5)	25.8 (-57.7 - 185.4)
Endocarditis	134,940 (64,377 - 258,233)	0.3 (0.1 - 0.5)	1.8 (0.8 - 3.4)	26.5 (-52.3 - 164.8)	39,795 (25,930 - 54,895)	47.2 (31.3 - 64.1)	0.4 (0.2 - 0.5)	0.5 (0.3 - 0.7)	-13.3 (-47.7 - 35.3)

Other cardiovascular and circulatory diseases					118,397 (63,537 - 189,178)	32.4 (17.3 - 51.2)	1.0 (0.5 - 1.6)	1.5 (0.8 - 2.4)	-39.4 (-73.0 - 16.2)
Chronic obstructive pulmonary disease	692,082 (187,442 - 1,838,769)	1.3 (0.3 - 3.4)	9.0 (2.4 - 23.9)	-40.5 (-90.6 - 90.4)	399,648 (122,352 - 907,700)	12.3 (3.9 - 27.8)	3.5 (1.1 - 7.6)	5.2 (1.6 - 11.8)	-54.9 (-91.4 - 35.5)
Pneumoconiosis	6,184 (2,885 - 12,266)	0.0 (0.0 - 0.0)	0.1 (0.0 - 0.2)	-45.1 (-81.9 - 28.8)	2,472 (1,315 - 4,256)	8.0 (4.3 - 13.9)	0.0 (0.0 - 0.0)	0.0 (0.0 - 0.1)	-62.6 (-84.8 - -23.2)
Asthma	99,074 (24,221 - 244,209)	0.2 (0.0 - 0.5)	1.3 (0.3 - 3.1)	-54.4 (-93.4 - 42.3)	60,156 (16,370 - 140,235)	11.7 (3.4 - 26.1)	0.5 (0.1 - 1.2)	0.8 (0.2 - 1.8)	-62.6 (-93.4 - 11.6)
Interstitial lung disease and pulmonary sarcoidosis	19,696 (5,446 - 50,280)	0.0 (0.0 - 0.1)	0.3 (0.1 - 0.6)	26.9 (-76.2 - 347.9)	14,341 (4,134 - 33,963)	9.3 (2.9 - 21.2)	0.1 (0.0 - 0.3)	0.2 (0.1 - 0.4)	10.6 (-77.9 - 253.6)
Other chronic respiratory diseases					12,787 (4,423 - 25,225)	24.3 (8.8 - 46.5)	0.1 (0.0 - 0.2)	0.2 (0.1 - 0.3)	-37.1 (-81.8 - 62.8)
Cirrhosis and other chronic liver diseases	1,091,373 (766,791 - 1,557,827)	2.0 (1.3 - 3.0)	13.5 (9.5 - 19.3)	-19.8 (-49.2 - 22.1)	455,347 (354,034 - 563,290)	33.6 (26.3 - 41.4)	3.8 (2.9 - 4.8)	5.7 (4.4 - 7.0)	-41.4 (-58.4 - -21.9)
Upper digestive system diseases	143,060 (57,614 - 287,428)	0.3 (0.1 - 0.6)	1.8 (0.7 - 3.7)	-54.2 (-85.3 - 15.1)	79,778 (36,222 - 134,122)	26.4 (11.8 - 43.9)	0.7 (0.3 - 1.2)	1.0 (0.5 - 1.7)	-65.1 (-87.0 - -21.2)
Appendicitis	104,245 (58,104 - 188,648)	0.2 (0.1 - 0.4)	1.4 (0.8 - 2.5)	-16.4 (-65.4 - 69.7)	36,479 (29,167 - 42,128)	81.1 (66.4 - 90.7)	0.3 (0.3 - 0.4)	0.5 (0.4 - 0.5)	-40.4 (-53.5 - -25.6)
Paralytic ileus and intestinal obstruction	258,804 (131,418 - 465,842)	0.5 (0.2 - 1.0)	3.5 (1.7 - 6.3)	-7.3 (-62.0 - 86.0)	127,951 (78,934 - 178,004)	52.6 (33.6 - 71.2)	1.1 (0.7 - 1.6)	1.7 (1.0 - 2.3)	-27.2 (-58.5 - 16.0)
Inguinal, femoral, and abdominal hernia	35,444 (18,846 - 59,296)	0.1 (0.0 - 0.1)	0.5 (0.2 - 0.8)	-35.4 (-71.7 - 18.8)	21,100 (12,910 - 29,791)	46.2 (28.9 - 65.5)	0.2 (0.1 - 0.3)	0.3 (0.2 - 0.4)	-45.4 (-70.7 - -8.5)
Inflammatory bowel disease	65,383 (22,868 - 145,938)	0.1 (0.0 - 0.3)	0.9 (0.3 - 1.9)	13.6 (-73.7 - 222.2)	17,166 (8,943 - 26,838)	43.0 (22.4 - 65.6)	0.2 (0.1 - 0.2)	0.2 (0.1 - 0.4)	-32.4 (-70.2 - 34.0)
Vascular intestinal disorders	54,633 (23,609 - 98,575)	0.1 (0.0 - 0.2)	0.7 (0.3 - 1.3)	-16.3 (-72.0 - 94.8)	36,457 (17,945 - 59,698)	36.7 (18.1 - 60.2)	0.3 (0.2 - 0.5)	0.5 (0.2 - 0.8)	-26.9 (-72.4 - 54.8)
Gallbladder and biliary diseases	359,142 (117,259 - 840,033)	0.7 (0.2 - 1.7)	4.7 (1.5 - 11.1)	10.0 (-74.5 - 214.8)	60,041 (33,416 - 86,748)	52.5 (28.9 - 76.3)	0.5 (0.3 - 0.8)	0.8 (0.4 - 1.1)	-39.8 (-68.7 - 1.1)
Pancreatitis	92,938 (31,895 - 219,873)	0.2 (0.1 - 0.4)	1.2 (0.4 - 2.8)	15.9 (-74.5 - 254.6)	34,806 (15,311 - 58,376)	33.3 (14.4 - 55.3)	0.3 (0.1 - 0.5)	0.4 (0.2 - 0.7)	-19.4 (-72.2 - 82.9)
Other digestive diseases					44,776 (24,762 - 65,503)	49.6 (27.0 - 72.7)	0.4 (0.2 - 0.6)	0.6 (0.3 - 0.9)	-37.9 (-70.5 - 15.0)
Alzheimer's disease and other dementias	530,711 (225,247 - 1,063,445)	1.1 (0.5 - 2.2)	7.3 (3.1 - 14.7)	-7.2 (-71.1 - 131.0)	204,316 (107,400 - 349,599)	2.6 (1.4 - 4.5)	1.9 (1.0 - 3.2)	2.8 (1.5 - 4.8)	-37.1 (-74.6 - 34.9)
Parkinson's disease	76,507 (33,262 - 157,926)	0.2 (0.1 - 0.3)	1.0 (0.4 - 2.1)	-6.8 (-70.7 - 112.9)	31,354 (16,016 - 54,876)	5.7 (2.9 - 9.9)	0.3 (0.1 - 0.5)	0.4 (0.2 - 0.7)	-35.2 (-73.9 - 40.5)
Epilepsy	59,494 (24,801 - 127,288)	0.1 (0.0 - 0.3)	0.8 (0.3 - 1.7)	-24.3 (-78.8 - 95.4)	18,249 (9,296 - 31,646)	14.0 (7.2 - 23.4)	0.2 (0.1 - 0.3)	0.2 (0.1 - 0.4)	-48.3 (-79.5 - 7.4)
Multiple sclerosis	40,480 (14,229 - 96,116)	0.1 (0.0 - 0.2)	0.5 (0.2 - 1.2)	22.4 (-76.8 - 253.3)	2,932 (1,586 - 4,895)	8.9 (4.9 - 14.5)	0.0 (0.0 - 0.0)	0.0 (0.0 - 0.1)	-31.6 (-70.0 - 44.1)
Motor neuron disease	2,654 (1,071 - 5,572)	0.0 (0.0 - 0.0)	0.0 (0.0 - 0.1)	12.3 (-68.9 - 187.4)	1,001 (458 - 1,842)	2.9 (1.3 - 5.4)	0.0 (0.0 - 0.0)	0.0 (0.0 - 0.0)	-14.9 (-70.8 - 96.2)
Other neurological disorders					6,462 (3,254 - 11,090)	12.0 (6.2 - 20.6)	0.1 (0.0 - 0.1)	0.1 (0.0 - 0.1)	-31.9 (-74.1 - 44.4)
Eating disorders	207 (62 - 513)	0.0 (0.0 - 0.0)	0.0 (0.0 - 0.0)	68.7 (-71.5 - 443.3)	18 (9 - 31)	3.8 (1.9 - 6.5)	0.0 (0.0 - 0.0)	0.0 (0.0 - 0.0)	-4.3 (-61.9 - 95.7)
Alcohol use disorders	77,214 (14,702 - 256,367)	0.1 (0.0 - 0.5)	0.9 (0.2 - 3.1)	48.7 (-88.8 - 496.0)	14,968 (3,941 - 37,515)	5.7 (1.5 - 14.1)	0.1 (0.0 - 0.3)	0.2 (0.0 - 0.5)	-15.3 (-88.2 - 173.2)
Drug use disorders	20,056 (4,313 - 62,994)	0.0 (0.0 - 0.1)	0.3 (0.1 - 0.8)	154.3 (-75.5 - 969.8)	6,297 (2,640 - 13,010)	3.8 (1.6 - 7.8)	0.1 (0.0 - 0.1)	0.1 (0.0 - 0.2)	-32.2 (-81.6 - 74.3)
Diabetes mellitus	677,499 (272,608 - 1,393,219)	1.3 (0.5 - 2.7)	8.5 (3.4 - 17.6)	15.7 (-63.0 - 197.1)	277,017 (132,141 - 470,513)	20.0 (9.5 - 34.0)	2.4 (1.1 - 4.1)	3.5 (1.7 - 6.0)	-14.2 (-65.9 - 73.9)

Chronic kidney disease	489,053 (298,346 - 770,903)	0.9 (0.5 - 1.5)	6.2 (3.8 - 9.9)	-12.8 (-53.9 - 50.3)	230,774 (152,270 - 324,752)	18.4 (12.2 - 25.9)	2.0 (1.3 - 2.8)	2.9 (1.9 - 4.1)	-34.1 (-60.1 - 3.6)
Acute glomerulonephritis	12,113 (2,864 - 32,582)	0.0 (0.0 - 0.1)	0.2 (0.0 - 0.4)	-46.9 (-91.7 - 68.1)	2,104 (756 - 4,165)	18.8 (7.0 - 36.7)	0.0 (0.0 - 0.0)	0.0 (0.0 - 0.1)	-73.7 (-92.4 - -30.8)
Bacterial skin diseases	404,770 (206,789 - 701,437)	0.8 (0.4 - 1.4)	5.3 (2.7 - 9.2)	106.3 (-6.4 - 275.7)	68,012 (42,784 - 85,325)	89.2 (82.9 - 93.8)	0.6 (0.4 - 0.8)	0.9 (0.6 - 1.1)	24.3 (8.5 - 44.0)
Decubitus ulcer	44,446 (21,706 - 87,126)	0.1 (0.0 - 0.2)	0.6 (0.3 - 1.2)	16.7 (-50.3 - 145.6)	15,518 (9,991 - 23,617)	74.3 (59.5 - 85.5)	0.1 (0.1 - 0.2)	0.2 (0.1 - 0.3)	-13.8 (-37.6 - 19.7)
Other skin and subcutaneous diseases	4,486 (2,152 - 9,459)	0.0 (0.0 - 0.0)	0.1 (0.0 - 0.1)	13.9 (-49.3 - 128.6)	1,778 (1,045 - 3,249)	44.8 (30.6 - 60.3)	0.0 (0.0 - 0.0)	0.0 (0.0 - 0.0)	-15.4 (-48.0 - 36.6)
Rheumatoid arthritis	36,352 (16,541 - 76,444)	0.1 (0.0 - 0.1)	0.5 (0.2 - 1.0)	-12.9 (-69.9 - 95.7)	12,475 (7,171 - 19,215)	23.5 (14.1 - 35.6)	0.1 (0.1 - 0.2)	0.2 (0.1 - 0.2)	-37.8 (-68.9 - 11.7)
Other musculoskeletal disorders					22,279 (13,826 - 32,759)	26.7 (16.8 - 39.6)	0.2 (0.1 - 0.3)	0.3 (0.2 - 0.4)	-11.9 (-51.8 - 47.1)
Congenital anomalies	81,628 (53,649 - 122,088)	0.2 (0.1 - 0.3)	1.2 (0.8 - 1.9)	-23.3 (-58.5 - 25.0)	127,788 (96,691 - 162,327)	20.7 (15.6 - 26.1)	1.3 (1.0 - 1.7)	1.9 (1.4 - 2.4)	-47.7 (-64.8 - -24.9)
Urinary diseases and male infertility	1,209,705 (609,607 - 2,243,660)	2.4 (1.2 - 4.5)	16.0 (8.0 - 29.7)	57.4 (-42.4 - 230.8)	195,772 (163,711 - 224,069)	71.7 (59.8 - 81.8)	1.7 (1.4 - 2.0)	2.6 (2.1 - 2.9)	-3.6 (-22.7 - 16.3)
Gynaecological diseases	14,352 (6,427 - 28,180)	0.0 (0.0 - 0.1)	0.2 (0.1 - 0.4)	-3.1 (-67.9 - 109.1)	4,017 (2,435 - 5,383)	34.9 (21.7 - 46.0)	0.0 (0.0 - 0.0)	0.1 (0.0 - 0.1)	-33.5 (-63.8 - 2.3)
Haemoglobinopathies and haemolytic anaemias	1,083 (405 - 2,540)	0.0 (0.0 - 0.0)	0.0 (0.0 - 0.0)	-8.5 (-69.6 - 131.2)	28,680 (17,294 - 45,384)	27.0 (19.1 - 35.5)	0.3 (0.2 - 0.4)	0.4 (0.2 - 0.6)	-48.4 (-67.2 - -22.9)
Endocrine, metabolic, blood, and immune disorders					18,935 (10,236 - 31,173)	13.1 (7.1 - 21.3)	0.2 (0.1 - 0.3)	0.2 (0.1 - 0.4)	-32.7 (-70.3 - 37.7)
Sudden infant death syndrome					41 (10 - 121)	0.0 (0.0 - 0.0)	0.0 (0.0 - 0.0)	0.0 (0.0 - 0.0)	-45.9 (-85.4 - 47.0)
Road injuries	457,495 (282,177 - 715,774)	0.9 (0.5 - 1.5)	5.9 (3.6 - 9.3)	-32.6 (-67.4 - 20.6)	145,520 (100,480 - 200,090)	11.7 (8.2 - 16.1)	1.3 (0.9 - 1.7)	1.9 (1.3 - 2.6)	-50.6 (-70.7 - -22.8)
Other transport injuries	68,984 (29,042 - 137,531)	0.1 (0.1 - 0.3)	0.9 (0.4 - 1.8)	-14.4 (-74.5 - 116.6)	17,392 (8,795 - 28,867)	18.9 (9.9 - 30.9)	0.1 (0.1 - 0.2)	0.2 (0.1 - 0.4)	-40.5 (-73.5 - 19.7)
Falls	261,818 (117,982 - 536,230)	0.5 (0.2 - 1.0)	3.4 (1.5 - 7.1)	-10.9 (-72.2 - 126.7)	104,939 (54,473 - 175,951)	14.9 (7.8 - 25.2)	0.9 (0.5 - 1.5)	1.4 (0.7 - 2.3)	-34.9 (-72.1 - 36.7)
Drowning	19,325 (4,094 - 64,216)	0.0 (0.0 - 0.1)	0.3 (0.1 - 0.9)	-46.0 (-95.9 - 138.2)	5,121 (1,364 - 13,697)	1.8 (0.5 - 4.7)	0.0 (0.0 - 0.1)	0.1 (0.0 - 0.2)	-66.6 (-95.8 - 24.6)
Fire, heat, and hot substances	58,848 (24,484 - 117,093)	0.1 (0.0 - 0.2)	0.8 (0.3 - 1.6)	-46.4 (-83.4 - 25.5)	21,385 (10,759 - 35,634)	17.7 (9.3 - 28.9)	0.2 (0.1 - 0.3)	0.3 (0.1 - 0.5)	-59.8 (-84.1 - -21.3)
Poisonings	9,905 (2,922 - 24,608)	0.0 (0.0 - 0.1)	0.1 (0.0 - 0.3)	-29.6 (-89.2 - 130.4)	3,586 (1,222 - 7,919)	5.1 (1.8 - 11.3)	0.0 (0.0 - 0.1)	0.0 (0.0 - 0.1)	-52.2 (-89.7 - 39.0)
Exposure to mechanical forces	98,517 (42,365 - 196,715)	0.2 (0.1 - 0.4)	1.3 (0.6 - 2.6)	-30.5 (-76.3 - 57.5)	30,084 (17,336 - 46,729)	22.1 (12.9 - 33.9)	0.3 (0.2 - 0.4)	0.4 (0.2 - 0.6)	-53.5 (-77.0 - -16.0)
Adverse effects of medical treatment	123,678 (61,577 - 225,545)	0.2 (0.1 - 0.5)	1.6 (0.8 - 3.0)	-17.0 (-66.8 - 79.0)	45,564 (27,898 - 66,063)	37.3 (23.7 - 53.2)	0.4 (0.2 - 0.6)	0.6 (0.4 - 0.9)	-37.3 (-64.5 - 5.0)
Animal contact	172,197 (55,420 - 364,883)	0.3 (0.1 - 0.8)	2.3 (0.7 - 5.0)	-16.9 (-77.2 - 106.4)	18,818 (8,680 - 30,325)	23.2 (14.4 - 34.8)	0.2 (0.1 - 0.3)	0.2 (0.1 - 0.4)	-52.0 (-76.6 - -12.6)
Foreign body	48,159 (16,013 - 118,676)	0.1 (0.0 - 0.3)	0.7 (0.2 - 1.7)	-37.7 (-87.1 - 72.5)	13,886 (5,614 - 29,551)	11.3 (4.5 - 23.4)	0.1 (0.1 - 0.3)	0.2 (0.1 - 0.4)	-55.3 (-86.9 - 16.4)
Environmental heat and cold exposure	22,630 (5,684 - 60,719)	0.0 (0.0 - 0.1)	0.3 (0.1 - 0.8)	-35.3 (-90.8 - 98.0)	9,139 (2,696 - 20,612)	17.0 (5.2 - 36.9)	0.1 (0.0 - 0.2)	0.1 (0.0 - 0.3)	-51.7 (-89.4 - 24.7)
Exposure to forces of nature	3,913 (1,331 - 9,753)	0.0 (0.0 - 0.0)	0.1 (0.0 - 0.1)	-84.7 (-97.2 - -51.1)	475 (223 - 869)	5.0 (2.4 - 9.1)	0.0 (0.0 - 0.0)	0.0 (0.0 - 0.0)	-88.0 (-95.7 - -70.5)
Other unintentional injuries	17,888 (7,559 - 35,450)	0.0 (0.0 - 0.1)	0.2 (0.1 - 0.5)	-46.5 (-82.5 - 24.2)	7,260 (3,572 - 13,189)	7.7 (3.8 - 13.8)	0.1 (0.0 - 0.1)	0.1 (0.0 - 0.2)	-61.1 (-85.6 - -16.4)

Self-harm	59,673 (15,045 - 178,337)	0.1 (0.0 - 0.3)	0.8 (0.2 - 2.2)	-16.4 (-88.3 - 210.7)	19,615 (6,061 - 49,783)	2.0 (0.6 - 5.1)	0.2 (0.1 - 0.4)	0.2 (0.1 - 0.6)	-44.4 (-90.3 - 88.8)
Interpersonal violence	50,794 (24,919 - 95,795)	0.1 (0.0 - 0.2)	0.7 (0.3 - 1.3)	-31.7 (-74.9 - 44.7)	16,663 (9,684 - 26,112)	4.1 (2.4 - 6.5)	0.1 (0.1 - 0.2)	0.2 (0.1 - 0.3)	-51.7 (-77.6 - - 8.7)
Conflict and terrorism	391,097 (171,771 - 810,606)	0.8 (0.3 - 1.5)	5.3 (2.3 - 11.0)	75.4 (-38.4 - 296.2)	66,182 (44,078 - 89,639)	51.1 (34.5 - 67.4)	0.6 (0.4 - 0.8)	0.9 (0.6 - 1.2)	-32.8 (-56.6 - - 4.6)
Executions and police conflict	436 (179 - 891)	0.0 (0.0 - 0.0)	0.0 (0.0 - 0.0)	105.9 (-46.7 - 464.2)	208 (96 - 401)	1.3 (0.6 - 2.5)	0.0 (0.0 - 0.0)	0.0 (0.0 - 0.0)	86.2 (-45.1 - 355.5)

Abbreviations: ASMR=age-standardised mortality rate. UI=uncertainty interval.
We did not produce sepsis estimates within causes for which the GBD study does not make incidence estimates.[2] In these cases, the incidence data cells are left blank. There were no recorded hospitalisations or deaths with Zika or Ebola virus disease in 1990, and thus we were unable to produce estimates for the percentage change in age-standardised sepsis incidence rate or percentage change in sepsis-associated age-standardised mortality rate from 1990 to 2017.

eTable 11. Sepsis-related mortality by location for all ages, both sexes, and all underlying causes, 1990 and 2017

	All underlying causes			Underlying infection			Underlying non-communicable disease			Underlying injury		
**Location**	**Percentage change in sepsis ASMR (95% UI), 1990-2017**	**Sepsis ASMR per 100,000 population (95% UI), 2017**	**Sepsis-related deaths (95% UI), 2017**	**Percentage change in sepsis ASMR (95% UI), 1990–2017**	**Sepsis ASMR per 100,000 population (95% UI), 2017**	**Sepsis-related deaths (95% UI), 2017**	**Percentage change in sepsis ASMR (95% UI), 1990–2017**	**Sepsis ASMR per 100,000 population (95% UI), 2017**	**Sepsis-related deaths (95% UI), 2017**	**Percentage change in sepsis ASMR (95% UI), 1990–2017**	**Sepsis ASMR per 100,000 population (95% UI), 2017**	**Sepsis-related deaths (95% UI), 2017**
Central Europe, eastern Europe, and central Asia	-42.2 (-51.0 - -32.6)	66.6 (59.5 - 74.8)	364,724 (324,651 - 413,095)	-38.3 (-51.8 - -22.8)	25.3 (20.8 - 29.5)	122,996 (101,385 - 144,130)	-43.5 (-55.5 - -28.9)	39.5 (34.1 - 46.3)	233,937 (201,823 - 277,803)	-51.8 (-65.2 - -35.6)	3.3 (2.6 - 4.2)	16,924 (13,266 - 21,602)
Central Asia	-40.6 (-49.3 - -30.8)	104.0 (92.4 - 116.9)	78,345 (70,237 - 87,921)	-49.2 (-59.5 - -37.4)	41.1 (34.5 - 47.5)	33,776 (28,264 - 39,028)	-30.4 (-46.2 - -11.8)	61.3 (52.1 - 72.5)	42,888 (36,508 - 50,256)	-55.6 (-67.1 - -41.4)	3.5 (2.8 - 4.3)	3,067 (2,437 - 3,806)
Armenia	-50.7 (-58.2 - -42.5)	68.0 (61.2 - 76.2)	2,596 (2,325 - 2,910)	-50.4 (-60.3 - -39.0)	24.1 (20.1 - 28.1)	859 (715 - 1,007)	-46.3 (-58.0 - -32.9)	43.1 (37.0 - 50.3)	1,709 (1,463 - 1,994)	-81.2 (-86.2 - -74.5)	2.0 (1.6 - 2.5)	71 (58 - 89)
Azerbaijan	-46.7 (-56.6 - -35.5)	107.0 (91.9 - 123.7)	8,641 (7,471 - 9,939)	-58.1 (-68.8 - -44.3)	45.1 (35.9 - 55.3)	3,706 (2,977 - 4,547)	-29.4 (-45.9 - -8.5)	62.6 (52.4 - 74.8)	4,963 (4,176 - 5,924)	-69.7 (-77.9 - -59.2)	2.3 (1.8 - 3.0)	230 (182 - 293)
Georgia	-28.4 (-40.5 - -13.7)	78.6 (69.0 - 89.3)	4,285 (3,744 - 4,923)	-48.1 (-60.1 - -32.6)	21.7 (18.0 - 25.4)	1,091 (904 - 1,280)	-13.9 (-34.1 - 10.6)	54.8 (46.6 - 65.2)	3,122 (2,642 - 3,748)	-17.8 (-42.5 - 15.5)	4.2 (3.4 - 5.4)	195 (155 - 248)
Kazakhstan	-43.8 (-53.0 - -33.7)	79.9 (69.7 - 90.8)	13,046 (11,418 - 14,748)	-45.6 (-57.8 - -31.9)	27.3 (22.1 - 32.4)	4,672 (3,802 - 5,527)	-41.1 (-54.5 - -24.6)	50.5 (42.4 - 60.3)	7,965 (6,713 - 9,457)	-52.2 (-65.9 - -35.7)	4.1 (3.2 - 5.3)	757 (581 - 977)
Kyrgyzstan	-54.9 (-62.7 - -46.1)	89.3 (78.2 - 102.2)	4,456 (3,943 - 5,005)	-64.5 (-71.3 - -56.3)	29.5 (24.9 - 33.9)	1,661 (1,411 - 1,911)	-46.1 (-60.5 - -30.3)	57.5 (48.1 - 68.8)	2,646 (2,228 - 3,124)	-61.4 (-71.6 - -48.9)	3.7 (2.9 - 4.6)	214 (170 - 270)
Mongolia	-61.2 (-66.8 - -54.2)	149.4 (131.7 - 170.8)	3,518 (3,100 - 3,990)	-72.3 (-77.4 - -65.6)	48.4 (41.3 - 56.3)	1,284 (1,083 - 1,514)	-53.1 (-62.6 - -40.9)	95.2 (80.2 - 114.9)	2,025 (1,722 - 2,428)	-50.5 (-67.1 - -31.7)	7.5 (5.7 - 9.8)	244 (183 - 318)
Tajikistan	-40.6 (-49.8 - -30.5)	154.2 (134.8 - 176.6)	11,898 (10,162 - 13,980)	-48.8 (-59.4 - -36.6)	73.0 (60.3 - 85.9)	6,847 (5,497 - 8,276)	-28.1 (-43.8 - -10.5)	79.7 (66.9 - 95.3)	4,845 (3,970 - 5,893)	-53.9 (-67.5 - -37.3)	3.6 (2.8 - 4.6)	302 (236 - 386)
Turkmenistan	-51.3 (-59.7 - -41.5)	106.9 (91.5 - 123.9)	4,418 (3,776 - 5,087)	-61.4 (-70.1 - -51.3)	40.6 (33.5 - 48.4)	1,872 (1,531 - 2,253)	-39.7 (-54.5 - -21.2)	66.0 (54.2 - 81.3)	2,508 (2,073 - 3,056)	-63.3 (-73.3 - -51.0)	2.8 (2.2 - 3.5)	134 (105 - 169)
Uzbekistan	-31.7 (-43.7 - -17.5)	109.8 (93.5 - 128.0)	25,422 (21,758 - 29,183)	-43.6 (-55.7 - -28.8)	43.0 (34.9 - 51.0)	11,784 (9,532 - 14,025)	-17.3 (-38.8 - 9.0)	65.2 (52.5 - 80.3)	13,042 (10,702 - 15,789)	-52.2 (-65.9 - -34.0)	3.1 (2.4 - 4.0)	921 (707 - 1,200)
Central Europe	-59.6 (-65.7 - -52.2)	42.3 (37.7 - 47.5)	85,807 (75,687 - 97,307)	-60.6 (-71.1 - -47.7)	13.5 (10.6 - 16.7)	26,331 (20,509 - 32,590)	-59.0 (-67.2 - -49.0)	28.6 (24.7 - 33.3)	59,934 (51,557 - 70,630)	-69.0 (-78.6 - -56.1)	1.8 (1.4 - 2.3)	3,015 (2,295 - 4,021)
Albania	-68.2 (-75.5 - -58.9)	42.4 (34.2 - 51.8)	1,557 (1,239 - 1,930)	-79.0 (-85.8 - -68.6)	14.3 (10.3 - 18.8)	497 (358 - 667)	-54.0 (-65.7 - -37.7)	30.3 (23.9 - 39.1)	1,159 (903 - 1,506)	-64.7 (-75.3 - -50.7)	1.5 (1.1 - 2.0)	49 (37 - 64)
Bosnia and Herzegovina	-52.6 (-61.7 - -42.2)	53.7 (45.5 - 63.2)	2,899 (2,443 - 3,428)	-64.5 (-75.2 - -46.0)	11.4 (8.9 - 15.4)	594 (460 - 820)	-49.6 (-61.4 - -36.3)	43.3 (36.0 - 52.2)	2,382 (1,968 - 2,907)	-63.5 (-75.8 - -45.1)	1.8 (1.4 - 2.5)	87 (64 - 123)
Bulgaria	-49.8 (-59.2 - -39.2)	50.4 (44.1 - 58.6)	6,861 (5,908 - 8,138)	-59.5 (-70.8 - -44.8)	15.9 (12.4 - 19.8)	1,978 (1,514 - 2,501)	-42.1 (-55.9 - -24.7)	36.4 (30.6 - 44.4)	5,256 (4,352 - 6,487)	-54.5 (-67.7 - -37.8)	1.8 (1.4 - 2.4)	187 (144 - 245)
Croatia	-57.2 (-64.1 - -49.1)	30.4 (26.9 - 34.6)	2,639 (2,328 - 3,033)	-64.5 (-74.4 - -52.2)	8.0 (6.5 - 9.6)	672 (540 - 814)	-57.7 (-66.4 - -47.6)	21.4 (18.4 - 25.2)	1,905 (1,626 - 2,260)	-59.7 (-74.0 - -39.6)	1.6 (1.1 - 2.2)	122 (83 - 181)
Czech Republic	-60.4 (-67.5 - -51.9)	31.5 (27.6 - 36.2)	6,397 (5,575 - 7,363)	-44.2 (-61.5 - -22.3)	12.3 (9.2 - 15.8)	2,476 (1,845 - 3,209)	-65.6 (-72.7 - -57.0)	19.4 (16.7 - 22.5)	3,993 (3,433 - 4,663)	-72.1 (-83.3 - -57.3)	1.4 (1.0 - 1.8)	238 (174 - 321)
Hungary	-62.9 (-68.7 - -56.1)	37.8 (33.4 - 42.8)	7,044 (6,181 - 8,087)	-64.2 (-72.6 - -54.1)	8.8 (7.2 - 10.4)	1,572 (1,272 - 1,862)	-61.8 (-69.2 - -52.8)	28.4 (24.4 - 33.1)	5,394 (4,619 - 6,319)	-76.8 (-86.1 - -64.2)	1.7 (1.2 - 2.3)	286 (203 - 402)

Montenegro	-40.4 (-54.3 - -23.4)	38.1 (31.7 - 47.1)	358 (296 - 446)	-43.4 (-60.1 - -20.4)	7.3 (5.6 - 9.4)	66 (49 - 84)	-38.5 (-55.3 - -16.8)	30.4 (24.4 - 38.6)	292 (234 - 373)	-50.9 (-63.8 - -34.2)	1.6 (1.2 - 2.0)	13 (10 - 16)
North Macedonia	-62.6 (-69.8 - -53.6)	41.9 (35.8 - 49.4)	1,271 (1,076 - 1,519)	-77.2 (-83.8 - -67.0)	7.8 (6.1 - 10.3)	214 (165 - 298)	-57.4 (-67.8 - -45.0)	34.3 (28.4 - 42.0)	1,071 (882 - 1,311)	-56.0 (-69.3 - -38.4)	1.3 (1.0 - 1.7)	37 (28 - 48)
Poland	-62.0 (-67.9 - -55.3)	38.2 (33.7 - 43.3)	25,833 (22,657 - 29,362)	-55.5 (-68.1 - -40.8)	12.9 (9.7 - 16.3)	8,685 (6,453 - 11,091)	-65.3 (-72.0 - -57.3)	24.7 (21.3 - 28.7)	16,906 (14,511 - 19,906)	-70.8 (-80.2 - -58.6)	1.8 (1.4 - 2.4)	1,036 (767 - 1,409)
Romania	-59.3 (-66.6 - -50.8)	54.1 (47.3 - 62.3)	18,185 (15,800 - 21,007)	-61.1 (-71.8 - -48.4)	20.2 (15.7 - 24.8)	6,128 (4,788 - 7,502)	-58.3 (-67.6 - -47.1)	33.5 (28.4 - 39.6)	12,119 (10,193 - 14,492)	-68.0 (-77.1 - -55.9)	2.0 (1.6 - 2.6)	557 (434 - 714)
Serbia	-43.8 (-54.3 - -31.7)	53.8 (46.7 - 63.3)	8,155 (7,004 - 9,677)	-42.4 (-56.8 - -24.3)	11.9 (9.3 - 14.7)	1,754 (1,360 - 2,181)	-42.3 (-55.1 - -25.4)	42.7 (35.8 - 52.3)	6,565 (5,498 - 8,089)	-58.5 (-70.1 - -43.1)	1.6 (1.3 - 2.1)	209 (163 - 273)
Slovakia	-59.3 (-66.1 - -50.9)	41.5 (35.9 - 47.5)	3,491 (3,024 - 3,997)	-62.1 (-74.6 - -45.6)	15.7 (11.4 - 20.1)	1,316 (956 - 1,691)	-57.3 (-65.6 - -46.7)	25.7 (22.3 - 30.1)	2,187 (1,890 - 2,571)	-67.4 (-78.4 - -51.5)	1.8 (1.3 - 2.4)	135 (99 - 188)
Slovenia	-63.4 (-69.7 - -56.2)	25.1 (21.8 - 28.6)	1,116 (960 - 1,279)	-63.1 (-75.3 - -46.6)	8.3 (6.0 - 11.0)	380 (266 - 508)	-63.0 (-70.3 - -53.9)	15.9 (13.7 - 18.4)	706 (604 - 821)	-67.8 (-79.8 - -51.7)	1.5 (1.1 - 2.1)	60 (40 - 86)
Eastern Europe	-31.1 (-43.5 - -17.7)	64.6 (57.3 - 73.6)	200,572 (176,901 - 231,220)	-3.8 (-25.2 - 21.5)	22.3 (18.3 - 26.6)	62,889 (51,521 - 75,147)	-38.6 (-53.4 - -20.7)	39.5 (33.7 - 47.8)	131,114 (111,187 - 160,466)	-43.6 (-60.8 - -23.6)	4.1 (3.2 - 5.2)	10,842 (8,435 - 13,797)
Belarus	-45.3 (-55.5 - -33.7)	42.8 (37.1 - 49.9)	6,375 (5,484 - 7,500)	-40.9 (-54.1 - -24.4)	11.2 (9.0 - 13.5)	1,528 (1,220 - 1,854)	-45.5 (-59.1 - -28.9)	29.7 (24.9 - 36.4)	4,631 (3,844 - 5,716)	-51.0 (-66.6 - -31.2)	2.9 (2.2 - 3.8)	378 (284 - 503)
Estonia	-61.6 (-69.3 - -53.1)	29.5 (25.1 - 34.0)	745 (630 - 863)	-49.8 (-62.9 - -33.6)	9.2 (7.0 - 11.5)	210 (160 - 263)	-62.0 (-71.7 - -51.2)	19.5 (16.2 - 23.1)	526 (433 - 626)	-79.0 (-86.1 - -70.2)	1.6 (1.2 - 2.1)	31 (22 - 41)
Latvia	-46.8 (-56.9 - -35.6)	43.4 (37.4 - 50.6)	1,643 (1,402 - 1,954)	-30.5 (-46.5 - -10.4)	13.4 (10.7 - 16.4)	445 (355 - 546)	-48.3 (-61.5 - -33.3)	28.2 (23.4 - 34.6)	1,156 (941 - 1,446)	-67.7 (-77.9 - -54.5)	2.7 (2.1 - 3.5)	78 (59 - 104)
Lithuania	-27.8 (-39.9 - -14.0)	46.0 (40.4 - 52.5)	2,486 (2,161 - 2,869)	5.3 (-21.0 - 35.5)	15.4 (12.3 - 18.7)	754 (596 - 916)	-34.0 (-48.7 - -16.6)	29.0 (24.7 - 34.7)	1,683 (1,419 - 2,049)	-51.9 (-68.2 - -31.6)	3.1 (2.3 - 4.1)	131 (96 - 176)
Moldova	-44.3 (-53.2 - -33.3)	81.9 (72.5 - 92.6)	4,089 (3,626 - 4,633)	-36.2 (-50.9 - -19.9)	31.6 (25.7 - 37.7)	1,430 (1,177 - 1,706)	-46.9 (-58.8 - -31.9)	47.4 (40.2 - 56.2)	2,524 (2,127 - 3,009)	-58.0 (-70.0 - -42.6)	3.8 (3.0 - 4.9)	184 (142 - 237)
Russia	-31.8 (-44.0 - -18.3)	66.8 (59.5 - 76.0)	141,660 (125,207 - 163,131)	-3.1 (-24.5 - 22.0)	23.5 (19.2 - 28.0)	45,890 (37,383 - 54,590)	-39.7 (-54.2 - -22.1)	40.5 (34.6 - 49.1)	91,020 (77,309 - 111,214)	-45.0 (-61.8 - -25.0)	4.2 (3.3 - 5.4)	7,870 (6,115 - 10,013)
Ukraine	-23.5 (-39.6 - -6.4)	65.1 (57.0 - 74.9)	43,575 (37,504 - 51,070)	9.2 (-16.3 - 38.5)	21.5 (17.6 - 25.9)	12,633 (10,216 - 15,238)	-32.5 (-49.9 - -12.1)	40.7 (34.1 - 49.6)	29,575 (24,540 - 36,760)	-31.5 (-52.4 - -5.1)	4.0 (3.1 - 5.1)	2,169 (1,698 - 2,757)
High-income	-45.1 (-53.8 - -35.0)	30.3 (26.7 - 34.4)	660,422 (573,766 - 759,927)	-36.0 (-55.4 - -10.6)	12.9 (9.8 - 16.4)	291,210 (215,484 - 376,347)	-49.2 (-58.3 - -38.3)	17.0 (15.0 - 19.4)	367,098 (319,466 - 424,767)	-57.9 (-71.0 - -41.5)	1.2 (0.9 - 1.5)	20,167 (14,918 - 27,291)
Australasia	-46.3 (-55.5 - -36.0)	20.1 (17.6 - 23.2)	10,380 (8,990 - 12,125)	-32.9 (-54.5 - -5.9)	6.8 (5.1 - 8.7)	3,615 (2,668 - 4,738)	-50.1 (-59.9 - -38.1)	13.0 (11.1 - 15.2)	6,628 (5,656 - 7,846)	-55.2 (-70.9 - -33.2)	0.8 (0.6 - 1.2)	367 (257 - 528)
Australia	-44.7 (-54.7 - -33.6)	19.9 (17.2 - 23.1)	8,702 (7,457 - 10,197)	-23.8 (-47.4 - 6.2)	6.8 (5.0 - 8.8)	3,079 (2,238 - 4,084)	-50.6 (-60.4 - -38.8)	12.7 (10.8 - 15.0)	5,515 (4,664 - 6,544)	-53.3 (-69.8 - -31.1)	0.8 (0.6 - 1.1)	307 (212 - 447)
New Zealand	-51.9 (-60.3 - -42.3)	21.5 (19.0 - 24.5)	1,678 (1,475 - 1,918)	-57.7 (-72.3 - -39.0)	6.6 (5.1 - 8.4)	537 (406 - 693)	-47.1 (-57.4 - -34.2)	14.3 (12.4 - 16.7)	1,113 (952 - 1,310)	-61.0 (-75.0 - -42.2)	1.0 (0.7 - 1.3)	59 (42 - 85)
High-income Asia Pacific	-61.0 (-69.3 - -50.7)	23.1 (19.2 - 27.5)	118,401 (95,294 - 143,547)	-54.9 (-72.0 - -31.9)	11.9 (8.1 - 16.3)	63,713 (41,909 - 88,341)	-64.5 (-71.5 - -56.5)	10.8 (9.5 - 12.5)	53,558 (46,247 - 62,989)	-72.9 (-81.4 - -60.6)	0.8 (0.6 - 1.1)	2,806 (2,112 - 3,813)
Brunei	-44.1 (-55.3 - -32.1)	77.7 (67.1 - 90.5)	207 (181 - 239)	-24.7 (-48.3 - 1.3)	35.8 (27.4 - 44.5)	90 (70 - 109)	-52.9 (-64.2 - -39.6)	40.8 (34.0 - 49.8)	111 (93 - 135)	-58.5 (-71.0 - -42.1)	2.6 (2.0 - 3.3)	10 (8 - 13)
Japan	-53.6 (-64.6 - -39.1)	22.1 (18.0 - 26.7)	95,616 (75,738 - 117,393)	-49.9 (-70.3 - -22.0)	11.8 (7.9 - 16.2)	53,361 (34,513 - 74,829)	-55.9 (-64.6 - -45.9)	10.0 (8.8 - 11.6)	41,743 (35,960 - 49,727)	-57.0 (-71.7 - -37.1)	0.7 (0.5 - 0.9)	1,891 (1,390 - 2,594)
South Korea	-77.2 (-81.6 - -72.2)	26.8 (23.0 - 31.1)	20,552 (17,552 - 23,755)	-67.6 (-77.9 - -55.9)	11.7 (8.4 - 15.2)	8,800 (6,255 - 11,437)	-81.1 (-85.2 - -76.1)	14.3 (12.2 - 16.9)	11,150 (9,468 - 13,137)	-84.4 (-89.3 - -77.1)	1.2 (0.9 - 1.6)	877 (657 - 1,203)
Singapore	-64.1 (-74.2 - -51.4)	31.4 (23.6 - 39.8)	2,026 (1,523 - 2,574)	-52.3 (-70.9 - -26.4)	22.7 (15.1 - 31.0)	1,463 (973 - 1,998)	-77.6 (-82.6 - -71.7)	8.5 (7.3 - 10.0)	554 (473 - 648)	-75.9 (-84.2 - -64.5)	0.4 (0.3 - 0.6)	28 (21 - 40)

Appendix: Global Burden of Sepsis

High-income North America	-28.9 (-40.6 - -16.0)	33.8 (30.0 - 38.5)	205,979 (181,163 - 235,725)	-26.5 (-48.2 - 1.6)	13.5 (10.6 - 16.9)	83,851 (64,609 - 106,052)	-29.3 (-42.5 - -13.5)	19.7 (17.2 - 22.8)	119,605 (103,387 - 139,219)	-36.1 (-56.8 - -9.6)	1.5 (1.1 - 2.0)	7,482 (5,488 - 10,131)
Canada	-40.6 (-51.4 - -28.7)	23.1 (20.2 - 26.2)	16,023 (13,838 - 18,446)	-30.5 (-54.0 - 1.7)	8.8 (6.6 - 11.2)	6,376 (4,696 - 8,244)	-44.6 (-54.8 - -32.8)	13.8 (12.0 - 16.1)	9,428 (8,158 - 11,038)	-49.8 (-67.2 - -24.9)	1.0 (0.7 - 1.4)	567 (398 - 826)
Greenland	-50.9 (-58.4 - -42.5)	88.9 (77.2 - 100.8)	51 (44 - 57)	-43.1 (-56.4 - -28.0)	31.5 (25.0 - 37.3)	17 (14 - 20)	-52.0 (-62.1 - -40.3)	53.5 (45.9 - 62.7)	31 (27 - 36)	-65.4 (-76.5 - -49.7)	5.6 (4.3 - 7.5)	3 (3 - 4)
USA	-27.7 (-39.6 - -14.5)	35.1 (31.0 - 39.9)	189,623 (166,669 - 216,828)	-25.9 (-47.3 - 2.1)	14.1 (11.0 - 17.5)	77,456 (59,889 - 97,597)	-27.9 (-41.3 - -11.4)	20.3 (17.7 - 23.5)	109,865 (94,867 - 128,333)	-34.7 (-56.1 - -7.4)	1.5 (1.2 - 2.1)	6,912 (5,070 - 9,293)
Southern Latin America	-39.3 (-48.6 - -29.8)	83.3 (72.9 - 94.3)	67,612 (58,772 - 76,747)	-14.8 (-34.7 - 8.2)	42.9 (33.8 - 51.4)	35,189 (27,486 - 42,294)	-52.6 (-60.8 - -42.6)	40.2 (34.6 - 46.1)	32,481 (27,903 - 37,479)	-57.6 (-69.5 - -42.3)	3.0 (2.4 - 3.8)	2,248 (1,772 - 2,845)
Argentina	-29.0 (-41.0 - -17.3)	97.1 (83.4 - 111.6)	51,697 (44,188 - 59,603)	13.8 (-13.4 - 45.1)	52.9 (41.0 - 64.1)	28,471 (21,929 - 34,625)	-50.3 (-59.6 - -40.0)	44.3 (37.8 - 51.8)	23,471 (19,916 - 27,368)	-52.3 (-66.2 - -34.2)	3.4 (2.6 - 4.3)	1,676 (1,302 - 2,152)
Chile	-64.9 (-70.6 - -58.8)	52.6 (45.7 - 59.8)	11,834 (10,292 - 13,509)	-68.7 (-76.4 - -59.1)	21.7 (17.4 - 26.3)	4,920 (3,929 - 5,956)	-60.1 (-67.5 - -51.4)	30.0 (25.4 - 35.2)	6,751 (5,698 - 7,936)	-72.9 (-81.3 - -62.2)	1.9 (1.5 - 2.5)	415 (317 - 541)
Uruguay	-36.5 (-46.2 - -25.2)	72.6 (63.7 - 83.0)	4,074 (3,562 - 4,682)	-15.1 (-33.2 - 5.3)	31.4 (25.5 - 37.0)	1,796 (1,451 - 2,140)	-46.2 (-56.1 - -34.2)	40.1 (34.4 - 46.9)	2,253 (1,919 - 2,667)	-42.3 (-60.4 - -21.1)	3.4 (2.6 - 4.3)	157 (121 - 197)
Western Europe	-49.8 (-58.0 - -40.7)	25.7 (22.8 - 29.1)	258,042 (226,588 - 296,610)	-40.1 (-58.3 - -15.3)	10.0 (7.5 - 12.7)	104,830 (77,923 - 135,657)	-53.3 (-61.7 - -43.2)	15.7 (13.7 - 18.1)	154,834 (134,275 - 179,600)	-66.2 (-77.8 - -50.7)	0.9 (0.6 - 1.2)	7,263 (5,172 - 10,234)
Andorra	-41.6 (-55.8 - -24.1)	24.1 (19.4 - 29.3)	36 (29 - 45)	-38.4 (-63.2 - -1.0)	10.8 (7.2 - 14.8)	17 (11 - 24)	-42.9 (-55.9 - -27.0)	13.4 (11.1 - 16.1)	19 (16 - 23)	-49.8 (-68.7 - -23.1)	0.7 (0.5 - 1.0)	1 (1 - 1)
Austria	-58.0 (-64.5 - -50.5)	19.0 (16.9 - 21.4)	3,554 (3,134 - 4,032)	-63.7 (-73.8 - -50.4)	4.5 (3.5 - 5.5)	844 (661 - 1,063)	-55.9 (-64.1 - -45.8)	14.2 (12.3 - 16.5)	2,679 (2,291 - 3,120)	-63.7 (-77.0 - -45.6)	0.9 (0.7 - 1.3)	150 (106 - 214)
Belgium	-39.8 (-50.9 - -26.8)	29.6 (25.0 - 34.6)	7,675 (6,421 - 9,089)	-12.3 (-41.4 - 28.6)	13.9 (10.0 - 18.2)	3,791 (2,686 - 5,020)	-52.1 (-61.5 - -40.9)	15.6 (13.6 - 18.1)	3,889 (3,360 - 4,551)	-56.0 (-71.1 - -36.0)	1.2 (0.9 - 1.6)	254 (179 - 358)
Cyprus	-64.9 (-70.6 - -58.1)	26.2 (22.8 - 30.4)	490 (424 - 569)	-54.4 (-66.5 - -38.9)	9.6 (7.3 - 12.1)	181 (138 - 229)	-68.1 (-74.7 - -60.1)	16.2 (13.7 - 19.1)	304 (254 - 361)	-75.5 (-83.4 - -65.0)	1.1 (0.8 - 1.4)	18 (13 - 24)
Denmark	-37.4 (-49.2 - -24.2)	30.6 (26.2 - 35.7)	3,629 (3,091 - 4,269)	-19.2 (-47.5 - 19.4)	12.4 (9.0 - 16.3)	1,550 (1,107 - 2,061)	-43.5 (-54.8 - -29.6)	18.0 (15.5 - 21.1)	2,073 (1,769 - 2,449)	-67.9 (-80.6 - -51.3)	0.8 (0.6 - 1.1)	87 (61 - 123)
Finland	-64.3 (-70.4 - -56.4)	18.4 (16.0 - 21.0)	2,293 (1,993 - 2,656)	-77.3 (-84.4 - -67.1)	4.6 (3.5 - 5.7)	582 (447 - 740)	-54.0 (-63.8 - -42.4)	13.3 (11.4 - 15.8)	1,674 (1,419 - 2,014)	-66.8 (-79.5 - -49.1)	1.0 (0.7 - 1.4)	102 (67 - 155)
France	-57.8 (-64.3 - -50.2)	23.7 (20.9 - 26.9)	37,255 (32,363 - 42,696)	-55.2 (-68.6 - -37.7)	9.0 (6.8 - 11.6)	15,040 (11,063 - 19,698)	-58.2 (-65.4 - -49.3)	14.8 (12.9 - 17.0)	22,637 (19,533 - 26,425)	-71.8 (-81.8 - -58.2)	1.2 (0.9 - 1.6)	1,584 (1,103 - 2,244)
Germany	-43.5 (-53.2 - -32.5)	27.1 (23.7 - 31.3)	54,666 (47,302 - 63,388)	-24.6 (-47.4 - 6.7)	9.7 (7.3 - 12.5)	20,122 (14,976 - 26,020)	-49.0 (-58.9 - -36.9)	17.5 (14.8 - 20.6)	35,091 (29,662 - 41,400)	-60.5 (-75.2 - -40.6)	0.9 (0.6 - 1.3)	1,525 (1,043 - 2,212)
Greece	-40.2 (-51.3 - -27.3)	24.5 (21.0 - 28.4)	6,525 (5,502 - 7,673)	-19.1 (-47.0 - 18.1)	8.6 (5.9 - 11.6)	2,436 (1,643 - 3,332)	-46.0 (-56.7 - -31.8)	15.5 (13.4 - 18.2)	4,082 (3,471 - 4,869)	-60.1 (-73.9 - -42.3)	1.0 (0.7 - 1.3)	174 (128 - 234)
Iceland	-48.0 (-59.0 - -34.5)	18.9 (16.0 - 22.1)	109 (92 - 130)	-47.3 (-69.6 - -13.4)	7.7 (5.2 - 10.7)	47 (31 - 66)	-46.9 (-56.9 - -34.9)	10.9 (9.5 - 12.6)	62 (53 - 72)	-57.5 (-72.3 - -37.6)	0.7 (0.5 - 1.0)	4 (3 - 5)
Ireland	-64.4 (-71.7 - -55.4)	24.3 (20.6 - 28.6)	1,794 (1,510 - 2,131)	-65.3 (-78.4 - -45.5)	10.1 (7.0 - 13.8)	762 (528 - 1,048)	-62.5 (-70.7 - -53.3)	14.2 (12.2 - 16.8)	1,038 (887 - 1,231)	-73.8 (-83.1 - -61.4)	0.6 (0.4 - 0.8)	38 (27 - 54)
Israel	-39.7 (-49.8 - -28.8)	34.9 (30.4 - 40.0)	4,244 (3,677 - 4,868)	-0.2 (-30.3 - 37.4)	15.9 (11.8 - 20.0)	1,982 (1,459 - 2,494)	-51.6 (-60.6 - -40.8)	18.9 (16.4 - 21.8)	2,252 (1,951 - 2,618)	-74.6 (-82.9 - -61.7)	1.1 (0.8 - 1.4)	115 (87 - 155)
Italy	-55.5 (-62.6 - -47.5)	18.5 (16.3 - 21.0)	30,073 (26,179 - 34,465)	-46.5 (-62.3 - -25.1)	5.9 (4.5 - 7.4)	9,450 (7,027 - 12,228)	-57.2 (-65.4 - -48.0)	12.6 (10.9 - 14.5)	20,639 (17,671 - 24,086)	-68.5 (-79.1 - -54.1)	0.7 (0.5 - 0.9)	896 (658 - 1,223)
Luxembourg	-52.2 (-61.1 - -41.7)	24.1 (20.6 - 28.4)	247 (210 - 292)	-39.6 (-60.1 - -12.3)	9.6 (7.0 - 12.6)	100 (71 - 133)	-57.4 (-66.0 - -46.6)	15.0 (12.7 - 17.7)	153 (129 - 181)	-67.4 (-79.1 - -51.3)	0.9 (0.7 - 1.3)	8 (6 - 12)
Malta	-45.7 (-55.4 - -34.8)	31.2 (27.0 - 36.0)	272 (235 - 315)	-28.5 (-50.8 - 0.7)	14.0 (10.3 - 18.1)	125 (92 - 163)	-54.1 (-62.8 - -43.7)	17.2 (15.0 - 19.8)	148 (128 - 172)	-57.2 (-73.0 - -35.0)	0.8 (0.6 - 1.1)	6 (4 - 9)
Netherlands	-38.2 (-49.3 - -25.8)	26.8 (23.1 - 31.1)	9,400 (8,073 - 10,984)	-18.2 (-43.7 - 14.6)	12.0 (8.9 - 15.5)	4,305 (3,162 - 5,593)	-47.6 (-57.6 - -34.8)	14.8 (12.8 - 17.2)	5,116 (4,379 - 5,983)	-49.5 (-68.9 - -21.4)	0.8 (0.5 - 1.1)	248 (163 - 380)

Norway	-51.1 (-61.5 - -39.1)	22.9 (19.6 - 26.6)	2,440 (2,064 - 2,882)	-48.7 (-68.1 - -21.5)	10.3 (7.5 - 13.5)	1,172 (839 - 1,550)	-51.0 (-60.7 - -39.2)	12.2 (10.6 - 14.2)	1,239 (1,066 - 1,455)	-65.0 (-78.0 - -46.5)	0.8 (0.6 - 1.2)	76 (51 - 113)
Portugal	-57.9 (-66.0 - -48.3)	38.2 (32.7 - 44.3)	10,035 (8,456 - 11,812)	-32.8 (-54.5 - -5.5)	17.7 (12.8 - 22.9)	4,790 (3,383 - 6,280)	-66.7 (-73.7 - -58.3)	20.5 (17.7 - 23.7)	5,306 (4,549 - 6,188)	-78.7 (-85.2 - -70.1)	1.1 (0.8 - 1.4)	204 (157 - 268)
Spain	-57.0 (-64.1 - -49.3)	24.0 (21.3 - 27.2)	26,832 (23,399 - 30,814)	-46.6 (-60.9 - -27.3)	9.2 (7.3 - 11.6)	10,611 (8,192 - 13,594)	-60.4 (-68.0 - -51.2)	14.9 (13.0 - 17.3)	16,495 (14,153 - 19,367)	-75.2 (-83.7 - -63.6)	0.7 (0.5 - 0.9)	570 (409 - 789)
Sweden	-44.5 (-54.8 - -32.0)	21.1 (18.4 - 24.3)	4,936 (4,265 - 5,722)	-43.0 (-63.0 - -16.4)	8.6 (6.4 - 11.1)	2,110 (1,562 - 2,758)	-45.3 (-55.5 - -32.8)	12.4 (10.7 - 14.3)	2,816 (2,407 - 3,283)	-51.2 (-68.2 - -26.3)	0.8 (0.6 - 1.1)	157 (110 - 226)
Switzerland	-48.7 (-58.5 - -37.7)	18.0 (15.7 - 20.6)	3,409 (2,945 - 3,912)	-49.2 (-66.3 - -24.1)	6.7 (5.1 - 8.6)	1,333 (980 - 1,723)	-45.7 (-56.5 - -33.6)	11.1 (9.7 - 12.8)	2,060 (1,774 - 2,411)	-65.4 (-80.5 - -43.5)	0.7 (0.5 - 1.1)	125 (80 - 203)
United Kingdom	-40.2 (-52.0 - -26.5)	34.8 (29.8 - 40.6)	47,860 (40,515 - 56,244)	-31.3 (-55.8 - 4.1)	16.1 (11.7 - 21.1)	23,370 (16,627 - 31,154)	-45.6 (-56.0 - -32.6)	19.0 (16.6 - 22.0)	24,901 (21,538 - 28,943)	-51.3 (-67.9 - -28.3)	0.8 (0.6 - 1.1)	912 (640 - 1,296)
Latin America and Caribbean	-55.7 (-60.8 - -50.1)	100.7 (92.0 - 110.3)	553,044 (506,128 - 605,965)	-56.7 (-64.2 - -49.4)	44.9 (38.1 - 51.0)	244,159 (207,344 - 277,855)	-53.5 (-61.1 - -44.5)	53.0 (46.6 - 60.5)	292,182 (257,209 - 333,139)	-63.5 (-72.5 - -52.4)	4.7 (3.8 - 5.9)	27,452 (22,191 - 34,067)
Andean Latin America	-65.7 (-70.1 - -60.5)	107.7 (95.9 - 121.4)	59,991 (53,527 - 67,519)	-66.7 (-73.8 - -58.9)	53.2 (43.2 - 62.6)	29,966 (24,416 - 35,270)	-62.4 (-68.8 - -55.7)	51.1 (44.8 - 58.7)	27,974 (24,540 - 32,034)	-76.7 (-82.7 - -68.8)	4.9 (3.8 - 6.2)	2,859 (2,235 - 3,609)
Bolivia	-64.5 (-70.5 - -57.7)	189.8 (160.3 - 221.2)	16,794 (14,237 - 19,646)	-68.4 (-75.4 - -60.4)	82.9 (66.5 - 101.3)	7,537 (6,020 - 9,465)	-59.1 (-67.7 - -49.5)	101.6 (83.3 - 121.1)	8,683 (7,147 - 10,361)	-71.7 (-80.6 - -60.1)	8.2 (6.0 - 10.8)	821 (583 - 1,080)
Ecuador	-57.2 (-62.8 - -50.6)	100.6 (88.8 - 113.9)	14,639 (12,917 - 16,513)	-62.7 (-71.0 - -54.7)	39.7 (31.8 - 47.5)	5,770 (4,645 - 6,889)	-51.7 (-60.0 - -42.1)	57.0 (49.2 - 66.2)	8,223 (7,095 - 9,548)	-60.7 (-72.0 - -46.5)	5.5 (4.2 - 7.0)	870 (669 - 1,109)
Peru	-68.9 (-74.4 - -62.4)	88.8 (75.0 - 104.1)	28,559 (24,096 - 33,428)	-66.2 (-74.5 - -56.8)	51.3 (40.2 - 62.0)	16,658 (13,101 - 20,141)	-69.3 (-74.9 - -62.6)	35.0 (29.5 - 41.5)	11,068 (9,302 - 13,098)	-83.5 (-88.3 - -77.1)	3.6 (2.7 - 4.7)	1,168 (867 - 1,540)
Caribbean	-42.4 (-49.5 - -35.3)	140.7 (127.0 - 156.2)	65,922 (59,639 - 73,177)	-43.3 (-53.3 - -33.5)	66.9 (57.2 - 77.3)	30,278 (25,875 - 34,858)	-40.3 (-49.8 - -29.8)	69.0 (60.7 - 78.0)	33,387 (29,388 - 37,772)	-46.2 (-59.9 - -30.5)	7.8 (6.3 - 9.7)	3,729 (2,986 - 4,560)
Antigua and Barbuda	-32.9 (-43.2 - -20.8)	83.5 (73.0 - 95.0)	78 (69 - 89)	-19.8 (-40.1 - 5.9)	39.1 (30.4 - 47.3)	36 (28 - 44)	-39.4 (-51.7 - -26.0)	45.8 (38.7 - 54.4)	43 (37 - 51)	-51.4 (-64.4 - -35.7)	2.6 (2.1 - 3.3)	2 (2 - 3)
The Bahamas	-32.3 (-42.0 - -21.2)	103.2 (90.8 - 115.8)	356 (313 - 399)	-25.3 (-42.5 - -4.8)	48.4 (39.4 - 57.5)	165 (133 - 199)	-36.1 (-47.2 - -23.3)	54.0 (46.6 - 62.1)	187 (161 - 215)	-42.4 (-58.3 - -23.2)	4.3 (3.4 - 5.3)	16 (13 - 20)
Barbados	-27.0 (-39.0 - -13.6)	95.3 (83.4 - 109.5)	428 (375 - 494)	-8.3 (-27.4 - 14.0)	51.2 (41.6 - 61.3)	227 (183 - 275)	-40.1 (-54.1 - -25.1)	44.7 (38.0 - 53.9)	206 (173 - 246)	-47.5 (-63.0 - -27.6)	2.3 (1.8 - 3.0)	9 (7 - 12)
Belize	-29.1 (-38.0 - -19.0)	137.6 (124.0 - 153.1)	383 (344 - 427)	-32.4 (-45.4 - -17.8)	61.5 (51.5 - 71.1)	175 (146 - 204)	-24.4 (-38.1 - -9.7)	73.0 (62.7 - 84.5)	195 (169 - 225)	-33.7 (-50.5 - -12.4)	7.6 (6.1 - 9.3)	25 (21 - 31)
Bermuda	-62.9 (-69.3 - -56.2)	32.8 (28.8 - 37.7)	39 (34 - 45)	-57.3 (-70.2 - -41.7)	12.7 (9.6 - 16.0)	15 (11 - 19)	-64.7 (-71.3 - -57.4)	20.6 (17.6 - 23.9)	25 (22 - 30)	-72.8 (-81.2 - -62.0)	1.0 (0.8 - 1.3)	1 (1 - 1)
Cuba	-37.4 (-47.8 - -25.1)	53.6 (45.9 - 62.4)	9,831 (8,348 - 11,550)	-27.2 (-49.1 - -0.8)	23.3 (17.1 - 29.5)	4,268 (3,052 - 5,447)	-40.1 (-51.5 - -26.7)	28.9 (24.7 - 34.0)	5,327 (4,508 - 6,322)	-58.5 (-72.2 - -38.9)	2.6 (1.9 - 3.6)	451 (320 - 640)
Dominica	-10.7 (-23.3 - 4.7)	130.8 (116.1 - 148.3)	109 (98 - 123)	2.5 (-18.6 - 26.8)	58.0 (47.9 - 69.4)	46 (38 - 54)	-17.2 (-32.7 - 0.4)	74.2 (64.3 - 85.3)	65 (56 - 75)	-32.3 (-49.9 - -11.8)	4.6 (3.7 - 5.7)	4 (3 - 4)
Dominican Republic	-49.3 (-57.4 - -39.8)	110.5 (95.1 - 126.9)	10,489 (8,993 - 12,120)	-47.5 (-58.4 - -34.9)	50.0 (39.9 - 60.4)	4,844 (3,847 - 5,892)	-50.7 (-60.9 - -38.4)	56.8 (47.4 - 66.8)	5,245 (4,380 - 6,185)	-45.9 (-63.6 - -22.9)	6.3 (4.6 - 8.6)	640 (464 - 872)
Grenada	-38.9 (-48.0 - -29.2)	134.6 (119.3 - 152.3)	210 (187 - 240)	-29.8 (-45.5 - -12.9)	60.6 (49.5 - 70.9)	96 (78 - 113)	-43.4 (-54.7 - -30.8)	73.8 (63.2 - 87.1)	115 (98 - 138)	-52.9 (-65.4 - -37.7)	4.8 (3.9 - 5.9)	7 (5 - 8)
Guyana	-35.0 (-44.9 - -23.8)	193.1 (168.7 - 219.6)	1,164 (1,016 - 1,325)	-27.2 (-39.8 - -13.7)	78.6 (66.1 - 90.2)	478 (403 - 552)	-38.6 (-51.3 - -23.7)	109.5 (91.5 - 130.0)	648 (543 - 770)	-38.7 (-54.0 - -19.7)	9.9 (7.9 - 12.3)	66 (52 - 82)
Haiti	-48.9 (-56.5 - -40.6)	384.0 (332.9 - 435.6)	31,342 (27,076 - 35,740)	-53.0 (-61.4 - -44.7)	162.1 (138.8 - 188.6)	15,343 (12,695 - 18,264)	-43.0 (-53.6 - -30.1)	211.2 (177.2 - 245.8)	14,639 (12,285 - 16,987)	-56.1 (-68.0 - -41.2)	21.3 (16.3 - 27.8)	2,015 (1,537 - 2,625)
Jamaica	-21.8 (-36.3 - -5.7)	105.1 (88.7 - 123.7)	2,954 (2,499 - 3,488)	-26.3 (-41.6 - -9.3)	35.2 (28.9 - 42.4)	972 (801 - 1,154)	-20.1 (-39.7 - 2.9)	69.1 (55.6 - 86.6)	1,960 (1,573 - 2,463)	7.4 (-21.8 - 44.4)	3.2 (2.5 - 4.0)	95 (73 - 120)

Puerto Rico	-50.2 (-58.8 - -41.0)	48.5 (42.5 - 55.9)	3,228 (2,792 - 3,758)	-47.7 (-62.0 - -31.1)	22.2 (17.0 - 27.0)	1,473 (1,126 - 1,824)	-53.5 (-63.3 - -42.1)	24.6 (20.7 - 29.5)	1,684 (1,393 - 2,041)	-35.4 (-58.6 - -0.7)	2.7 (1.9 - 4.0)	138 (100 - 197)
Saint Lucia	-46.1 (-54.4 - -36.7)	104.1 (92.6 - 117.5)	202 (181 - 228)	-37.8 (-50.5 - -23.8)	42.6 (35.3 - 50.2)	80 (67 - 94)	-49.8 (-60.4 - -38.6)	62.1 (52.7 - 73.1)	123 (104 - 146)	-56.3 (-67.5 - -42.3)	3.9 (3.1 - 4.9)	8 (6 - 10)
Saint Vincent and the Grenadines	-26.3 (-37.2 - -13.3)	135.9 (120.8 - 152.5)	175 (155 - 196)	-21.8 (-36.7 - -3.9)	56.6 (48.1 - 65.7)	71 (60 - 82)	-28.5 (-44.1 - -11.5)	79.3 (67.2 - 93.0)	104 (88 - 123)	-29.0 (-46.7 - -8.7)	5.1 (4.2 - 6.2)	7 (5 - 8)
Suriname	-33.4 (-42.3 - -23.3)	164.4 (147.4 - 183.3)	879 (786 - 981)	-29.9 (-41.9 - -17.0)	69.9 (60.4 - 79.5)	367 (318 - 416)	-34.1 (-46.3 - -20.1)	89.4 (76.6 - 103.6)	482 (413 - 558)	-45.5 (-60.7 - -26.1)	8.1 (6.4 - 10.3)	46 (36 - 58)
Trinidad and Tobago	-44.9 (-56.9 - -30.0)	96.9 (80.2 - 118.7)	1,556 (1,263 - 1,932)	-41.7 (-54.5 - -25.6)	31.5 (24.9 - 38.4)	484 (384 - 592)	-44.4 (-60.0 - -25.1)	64.2 (50.1 - 81.7)	1,057 (811 - 1,372)	-53.7 (-67.6 - -36.0)	4.0 (3.0 - 5.2)	61 (46 - 81)
Virgin Islands	-37.0 (-48.1 - -25.7)	77.4 (66.8 - 89.0)	127 (107 - 147)	-25.0 (-41.1 - -4.6)	30.2 (24.5 - 36.1)	47 (38 - 57)	-41.6 (-54.1 - -28.3)	47.0 (39.1 - 55.3)	80 (65 - 94)	-49.1 (-63.3 - -31.2)	3.1 (2.4 - 3.9)	5 (4 - 6)
Central Latin America	-57.4 (-62.5 - -51.6)	88.8 (80.5 - 98.3)	204,516 (185,367 - 226,776)	-62.6 (-68.9 - -56.1)	33.6 (28.9 - 38.3)	77,764 (66,871 - 88,597)	-51.6 (-60.5 - -40.8)	52.4 (45.4 - 61.1)	119,627 (103,622 - 139,346)	-66.9 (-75.2 - -56.9)	4.2 (3.4 - 5.2)	10,556 (8,509 - 13,073)
Colombia	-62.3 (-67.4 - -55.9)	56.5 (49.8 - 64.2)	29,293 (25,814 - 33,157)	-56.8 (-65.0 - -46.9)	23.3 (19.2 - 27.5)	11,851 (9,750 - 13,931)	-63.2 (-70.2 - -54.8)	32.2 (27.7 - 37.7)	16,948 (14,520 - 19,853)	-79.6 (-84.8 - -73.1)	2.4 (1.9 - 3.1)	1,273 (1,004 - 1,622)
Costa Rica	-43.6 (-51.2 - -34.9)	50.2 (45.4 - 55.6)	2,413 (2,175 - 2,682)	-47.9 (-59.2 - -34.5)	15.9 (13.3 - 18.6)	766 (640 - 901)	-39.6 (-50.3 - -27.3)	32.8 (28.7 - 37.6)	1,569 (1,376 - 1,807)	-51.0 (-66.1 - -29.7)	2.8 (2.2 - 3.6)	138 (107 - 177)
El Salvador	-56.4 (-63.6 - -48.0)	112.7 (94.9 - 132.3)	6,505 (5,484 - 7,620)	-53.6 (-64.1 - -41.2)	50.2 (39.0 - 61.4)	2,905 (2,250 - 3,556)	-52.0 (-62.0 - -39.3)	59.2 (48.0 - 72.3)	3,397 (2,759 - 4,154)	-82.7 (-87.6 - -75.7)	5.2 (3.9 - 6.9)	313 (235 - 413)
Guatemala	-65.4 (-70.7 - -59.2)	193.3 (168.0 - 219.6)	23,818 (20,646 - 27,179)	-69.9 (-76.0 - -64.3)	99.1 (81.7 - 114.6)	12,631 (10,379 - 14,938)	-56.5 (-67.8 - -42.9)	88.3 (74.5 - 104.5)	10,291 (8,698 - 12,142)	-71.5 (-78.3 - -63.3)	8.0 (6.4 - 9.8)	1,136 (913 - 1,398)
Honduras	-48.7 (-57.7 - -38.4)	152.7 (127.8 - 178.1)	9,875 (8,371 - 11,481)	-57.3 (-65.5 - -48.6)	53.4 (43.5 - 64.9)	3,608 (2,966 - 4,365)	-40.1 (-52.4 - -24.9)	94.9 (77.4 - 112.3)	5,878 (4,871 - 6,905)	-59.5 (-71.5 - -45.2)	7.2 (5.4 - 9.4)	560 (422 - 730)
Mexico	-55.4 (-61.2 - -48.4)	94.2 (84.6 - 106.1)	105,955 (95,151 - 119,093)	-64.1 (-70.8 - -57.9)	31.5 (27.0 - 36.0)	35,685 (30,586 - 40,798)	-47.9 (-58.7 - -35.1)	59.5 (50.6 - 70.7)	66,185 (56,481 - 78,681)	-60.7 (-71.3 - -47.1)	4.6 (3.7 - 5.9)	5,660 (4,494 - 7,180)
Nicaragua	-62.6 (-68.4 - -56.5)	77.5 (67.8 - 89.2)	3,884 (3,406 - 4,430)	-71.9 (-77.7 - -65.0)	27.8 (23.1 - 32.7)	1,500 (1,243 - 1,781)	-52.0 (-61.7 - -41.1)	47.8 (40.3 - 56.9)	2,273 (1,919 - 2,707)	-71.7 (-79.7 - -60.8)	2.9 (2.3 - 3.8)	161 (125 - 206)
Panama	-41.5 (-50.1 - -31.6)	68.0 (60.3 - 77.1)	2,655 (2,357 - 3,007)	-26.3 (-42.1 - -9.3)	31.9 (26.2 - 38.2)	1,233 (1,014 - 1,466)	-48.2 (-57.7 - -37.0)	35.1 (30.0 - 40.9)	1,380 (1,180 - 1,609)	-64.5 (-74.6 - -51.2)	2.4 (1.9 - 3.1)	95 (74 - 122)
Venezuela	-50.9 (-58.7 - -41.6)	74.0 (64.0 - 86.5)	20,012 (17,351 - 23,360)	-54.1 (-63.9 - -43.2)	28.0 (22.8 - 33.8)	7,586 (6,180 - 9,167)	-47.3 (-58.6 - -34.3)	43.5 (36.0 - 52.3)	11,599 (9,610 - 13,958)	-57.4 (-71.0 - -40.5)	4.0 (3.1 - 5.2)	1,221 (934 - 1,590)
Tropical Latin America	-54.6 (-60.2 - -48.1)	103.5 (93.6 - 113.8)	223,404 (201,979 - 246,053)	-52.0 (-61.0 - -42.2)	50.0 (41.8 - 57.4)	106,542 (88,504 - 122,514)	-56.1 (-63.6 - -46.6)	51.2 (44.9 - 58.5)	111,559 (97,819 - 127,952)	-57.8 (-68.6 - -44.3)	4.5 (3.6 - 5.6)	10,363 (8,325 - 12,877)
Brazil	-54.9 (-60.5 - -48.5)	103.4 (93.5 - 113.8)	217,618 (196,489 - 239,599)	-52.2 (-61.2 - -42.6)	50.2 (42.0 - 57.6)	104,269 (86,471 - 120,087)	-56.5 (-64.0 - -47.0)	50.9 (44.7 - 58.3)	108,243 (95,045 - 123,927)	-58.3 (-69.0 - -44.8)	4.5 (3.7 - 5.7)	10,059 (8,083 - 12,578)
Paraguay	-38.9 (-49.5 - -26.2)	107.6 (93.0 - 126.7)	5,786 (5,001 - 6,812)	-44.8 (-56.0 - -32.7)	40.8 (33.3 - 48.8)	2,273 (1,854 - 2,745)	-34.5 (-49.0 - -16.6)	63.8 (52.1 - 77.7)	3,316 (2,720 - 4,016)	-28.6 (-49.9 - -2.5)	5.0 (3.8 - 6.5)	304 (232 - 395)
North Africa and Middle East	-62.2 (-66.7 - -57.2)	105.5 (95.7 - 116.7)	497,164 (453,733 - 544,402)	-68.0 (-74.1 - -62.3)	39.2 (33.8 - 44.0)	189,894 (165,335 - 213,292)	-60.6 (-68.1 - -52.1)	55.7 (48.1 - 64.5)	240,288 (208,371 - 278,806)	-28.2 (-49.5 - -1.2)	13.3 (10.0 - 16.8)	77,467 (57,663 - 98,177)
Afghanistan	-56.1 (-64.3 - -48.4)	414.1 (363.5 - 467.5)	93,640 (80,710 - 107,796)	-70.0 (-82.1 - -61.8)	144.2 (124.2 - 164.6)	44,588 (37,666 - 52,485)	-36.3 (-51.1 - -10.6)	251.3 (213.0 - 294.8)	43,285 (36,046 - 51,118)	-49.5 (-64.7 - -11.8)	27.6 (21.7 - 34.3)	6,832 (5,409 - 8,466)
Algeria	-62.2 (-68.1 - -55.5)	60.5 (53.5 - 69.3)	19,665 (17,407 - 22,458)	-62.2 (-70.9 - -52.7)	19.4 (15.9 - 23.0)	6,219 (5,111 - 7,383)	-60.4 (-68.7 - -50.6)	39.9 (34.0 - 47.3)	12,795 (10,910 - 15,371)	-67.4 (-77.9 - -52.5)	3.7 (2.6 - 5.4)	1,407 (988 - 2,030)
Bahrain	-65.2 (-72.0 - -56.8)	62.1 (52.2 - 76.5)	399 (339 - 483)	-59.0 (-68.1 - -47.5)	22.2 (17.7 - 26.7)	131 (108 - 156)	-67.5 (-76.2 - -55.7)	39.4 (30.5 - 52.7)	253 (198 - 336)	-73.2 (-82.7 - -61.3)	1.8 (1.4 - 2.5)	23 (17 - 31)
Egypt	-63.4 (-69.7 - -55.8)	140.9 (120.8 - 165.2)	80,662 (70,098 - 93,411)	-69.3 (-76.7 - -60.8)	64.4 (49.9 - 80.3)	39,487 (31,149 - 47,879)	-54.3 (-64.3 - -41.8)	73.6 (61.2 - 89.9)	37,688 (31,618 - 44,943)	-63.3 (-76.0 - -44.2)	6.4 (4.6 - 8.8)	5,226 (3,815 - 7,136)

Iran	-66.0 (-71.3 - -59.8)	54.7 (48.8 - 61.6)	34,810 (31,163 - 39,052)	-60.8 (-70.0 - -50.8)	17.7 (14.1 - 21.1)	11,162 (8,916 - 13,224)	-63.4 (-71.6 - -54.3)	35.5 (30.1 - 41.7)	22,128 (18,864 - 25,913)	-81.1 (-86.9 - -73.4)	3.9 (3.0 - 5.1)	3,058 (2,346 - 4,012)
Iraq	-52.4 (-61.1 - -41.1)	115.1 (97.9 - 135.9)	41,387 (33,879 - 50,445)	-70.2 (-76.4 - -63.5)	23.0 (19.7 - 26.7)	9,237 (7,675 - 11,109)	-67.3 (-74.7 - -58.9)	49.3 (41.4 - 58.6)	13,599 (11,270 - 16,347)	102.6 (29.1 - 203.4)	44.7 (29.3 - 62.4)	19,015 (12,313 - 26,588)
Jordan	-63.1 (-69.4 - -54.8)	58.0 (50.1 - 67.7)	3,525 (3,021 - 4,127)	-59.9 (-70.1 - -48.5)	20.0 (15.8 - 24.0)	1,295 (1,021 - 1,576)	-63.6 (-71.9 - -53.5)	37.5 (31.1 - 46.0)	2,131 (1,753 - 2,600)	-72.2 (-80.7 - -61.7)	2.1 (1.7 - 2.7)	190 (147 - 241)
Kuwait	-62.2 (-70.9 - -51.1)	33.2 (26.9 - 39.7)	751 (622 - 884)	-23.0 (-49.9 - 14.1)	18.7 (12.9 - 24.7)	393 (276 - 518)	-58.6 (-67.7 - -48.1)	13.9 (11.8 - 16.6)	327 (278 - 389)	-95.2 (-97.1 - -92.2)	1.4 (1.1 - 2.0)	53 (39 - 73)
Lebanon	-74.8 (-79.4 - -69.4)	34.8 (29.9 - 40.6)	2,056 (1,768 - 2,388)	-68.0 (-77.2 - -57.3)	12.0 (9.1 - 15.3)	694 (527 - 882)	-74.1 (-79.9 - -67.0)	21.5 (17.9 - 25.9)	1,240 (1,031 - 1,488)	-87.8 (-91.9 - -81.7)	2.4 (1.7 - 3.2)	185 (131 - 256)
Libya	-41.9 (-53.0 - -29.1)	78.0 (67.0 - 90.2)	3,647 (3,140 - 4,204)	-46.8 (-60.9 - -30.9)	23.2 (18.1 - 29.6)	1,021 (800 - 1,291)	-47.5 (-59.7 - -32.6)	44.1 (36.4 - 53.6)	1,864 (1,543 - 2,259)	37.5 (-17.7 - 106.0)	12.9 (8.9 - 17.6)	864 (587 - 1,191)
Morocco	-63.3 (-70.5 - -54.6)	97.8 (80.8 - 116.7)	28,173 (23,266 - 33,599)	-72.1 (-78.6 - -64.3)	34.0 (27.2 - 41.8)	9,746 (7,852 - 12,060)	-53.8 (-64.7 - -39.7)	61.9 (49.3 - 77.2)	17,639 (14,123 - 21,919)	-63.6 (-76.0 - -45.9)	5.0 (3.4 - 7.4)	1,692 (1,164 - 2,522)
Palestine	-49.1 (-58.0 - -39.6)	90.3 (79.6 - 102.9)	2,448 (2,174 - 2,770)	-47.7 (-58.7 - -35.2)	30.7 (25.7 - 37.3)	848 (718 - 990)	-43.1 (-56.8 - -28.1)	58.3 (48.8 - 69.8)	1,498 (1,262 - 1,798)	-79.6 (-85.8 - -71.2)	3.9 (3.1 - 4.8)	159 (125 - 199)
Oman	-64.9 (-71.9 - -56.0)	58.3 (47.7 - 70.4)	1,125 (930 - 1,350)	-61.7 (-71.8 - -49.4)	28.2 (20.4 - 36.0)	506 (390 - 635)	-64.9 (-74.0 - -53.6)	28.4 (22.7 - 35.1)	517 (416 - 634)	-74.9 (-86.8 - -57.1)	4.0 (2.5 - 6.2)	147 (88 - 237)
Qatar	-69.7 (-77.5 - -60.8)	42.9 (34.4 - 52.7)	297 (244 - 362)	-57.6 (-73.4 - -39.3)	15.6 (10.9 - 20.7)	95 (70 - 122)	-74.0 (-82.1 - -63.8)	25.6 (19.5 - 33.6)	156 (123 - 197)	-69.3 (-80.6 - -53.6)	2.4 (1.6 - 3.4)	52 (35 - 76)
Saudi Arabia	-64.8 (-71.2 - -57.6)	66.5 (56.4 - 77.8)	10,233 (8,721 - 12,022)	-61.0 (-71.0 - -50.0)	32.5 (25.3 - 39.8)	4,450 (3,462 - 5,384)	-68.8 (-76.2 - -60.2)	28.5 (23.7 - 34.2)	3,837 (3,187 - 4,725)	-59.9 (-74.4 - -40.5)	6.9 (4.9 - 9.2)	2,146 (1,514 - 2,942)
Sudan	-63.8 (-69.8 - -57.0)	182.6 (154.9 - 211.6)	55,750 (46,514 - 66,774)	-68.7 (-76.2 - -60.7)	75.9 (60.9 - 91.6)	25,698 (20,154 - 32,616)	-54.6 (-65.1 - -43.4)	100.8 (81.9 - 122.6)	27,271 (22,147 - 34,214)	-74.6 (-81.9 - -63.9)	11.1 (8.2 - 15.2)	3,789 (2,838 - 5,083)
Syria	-7.8 (-34.9 - 28.1)	155.0 (112.7 - 208.9)	23,234 (15,957 - 32,552)	-61.9 (-70.8 - -51.9)	22.3 (18.0 - 27.2)	2,725 (2,188 - 3,334)	-64.8 (-73.3 - -54.4)	37.1 (30.2 - 46.4)	4,288 (3,497 - 5,357)	1,219.1 (613.0 - 2,004.1)	97.3 (55.9 - 147.4)	16,424 (9,424 - 25,817)
Tunisia	-63.6 (-71.5 - -55.0)	54.6 (44.8 - 66.5)	5,862 (4,776 - 7,169)	-60.4 (-71.1 - -47.4)	17.7 (13.5 - 22.7)	1,874 (1,426 - 2,393)	-63.5 (-73.3 - -52.7)	34.7 (27.2 - 43.4)	3,708 (2,903 - 4,649)	-67.1 (-78.6 - -50.1)	4.2 (3.0 - 5.8)	504 (352 - 697)
Turkey	-78.4 (-81.9 - -74.6)	45.0 (39.1 - 51.2)	36,418 (31,602 - 41,683)	-81.3 (-85.9 - -76.3)	14.2 (11.3 - 17.2)	11,463 (8,953 - 13,927)	-76.9 (-81.5 - -71.3)	29.2 (24.6 - 34.6)	23,692 (19,930 - 28,191)	-66.3 (-76.8 - -53.6)	2.9 (2.2 - 3.8)	2,426 (1,797 - 3,156)
United Arab Emirates	-46.2 (-58.5 - -32.1)	98.2 (78.7 - 120.0)	2,681 (2,134 - 3,301)	-40.1 (-59.1 - -13.5)	41.8 (23.2 - 59.1)	807 (602 - 1,096)	-48.7 (-62.2 - -31.8)	49.8 (39.0 - 62.6)	1,372 (1,065 - 1,710)	-53.1 (-72.9 - -22.7)	7.3 (4.8 - 10.6)	529 (345 - 766)
Yemen	-56.7 (-66.4 - -44.1)	224.6 (183.2 - 280.7)	49,846 (40,147 - 62,208)	-72.4 (-83.3 - -62.3)	69.6 (53.7 - 90.6)	17,277 (12,765 - 23,630)	-50.8 (-64.3 - -31.2)	115.4 (89.7 - 149.8)	20,685 (15,788 - 27,118)	51.0 (-10.1 - 173.2)	45.4 (33.2 - 58.5)	12,670 (9,073 - 16,624)
South Asia	-55.3 (-61.4 - -48.9)	289.6 (258.7 - 326.3)	3,655,232 (3,302,940 - 4,072,196)	-59.3 (-65.6 - -52.1)	157.2 (132.9 - 192.0)	1,975,574 (1,711,005 - 2,333,894)	-49.0 (-61.1 - -35.4)	122.6 (102.9 - 148.7)	1,537,091 (1,314,435 - 1,821,887)	-45.6 (-60.5 - -27.7)	13.4 (10.4 - 16.9)	185,971 (147,483 - 228,778)
Bangladesh	-75.6 (-79.7 - -70.2)	184.3 (159.5 - 215.5)	213,628 (184,739 - 246,668)	-79.8 (-84.7 - -72.3)	83.2 (69.4 - 108.2)	97,199 (81,605 - 122,290)	-69.7 (-76.8 - -61.7)	98.2 (80.5 - 118.1)	112,200 (92,972 - 134,030)	-70.3 (-77.9 - -60.4)	5.4 (4.3 - 6.7)	6,974 (5,587 - 8,696)
Bhutan	-72.7 (-77.8 - -67.1)	171.0 (141.3 - 201.5)	1,043 (869 - 1,221)	-75.6 (-80.7 - -70.1)	78.8 (61.0 - 98.8)	490 (384 - 615)	-68.8 (-77.0 - -58.9)	86.2 (68.8 - 106.1)	508 (407 - 620)	-70.2 (-80.0 - -56.9)	8.9 (6.2 - 12.3)	62 (43 - 84)
India	-54.2 (-60.9 - -47.3)	297.7 (264.9 - 336.2)	2,942,916 (2,638,480 - 3,289,401)	-57.4 (-64.1 - -49.9)	168.6 (142.1 - 206.1)	1,648,322 (1,417,402 - 1,965,217)	-48.7 (-62.2 - -33.3)	118.3 (98.8 - 147.4)	1,172,987 (993,007 - 1,424,466)	-46.6 (-61.9 - -27.5)	14.3 (11.0 - 18.1)	155,628 (122,127 - 194,027)
Nepal	-64.4 (-69.8 - -58.1)	265.2 (229.4 - 303.1)	54,638 (47,275 - 62,325)	-70.8 (-76.4 - -64.4)	133.4 (103.7 - 163.5)	27,555 (21,856 - 33,549)	-54.2 (-65.4 - -39.9)	121.8 (98.9 - 148.9)	24,707 (20,138 - 29,641)	-45.7 (-63.3 - -23.9)	13.1 (9.3 - 17.5)	2,964 (2,149 - 3,983)
Pakistan	-41.4 (-51.1 - -30.5)	318.9 (273.4 - 370.1)	443,007 (381,689 - 510,976)	-56.0 (-64.1 - -45.7)	132.8 (109.8 - 167.1)	202,008 (167,763 - 247,475)	-22.6 (-39.7 - -5.1)	179.1 (148.9 - 210.6)	226,690 (188,288 - 264,572)	-19.1 (-42.3 - 9.8)	13.0 (9.8 - 16.7)	20,343 (15,087 - 26,555)

Southeast Asia, east Asia, and Oceania	-69.9 (-75.3 - -64.6)	80.6 (72.3 - 91.4)	1,781,344 (1,596,126 - 2,021,415)	-70.5 (-75.6 - -65.4)	34.5 (29.5 - 39.0)	721,104 (621,537 - 821,069)	-69.0 (-77.5 - -59.1)	43.6 (36.8 - 52.5)	1,001,984 (841,707 - 1,213,873)	-69.1 (-77.2 - -58.1)	3.6 (2.8 - 4.6)	82,690 (65,019 - 105,159)
East Asia	-79.6 (-84.2 - -74.5)	44.9 (38.4 - 53.7)	774,765 (663,248 - 927,166)	-83.3 (-87.1 - -78.3)	13.7 (10.9 - 16.8)	219,065 (175,648 - 266,922)	-77.2 (-84.5 - -68.1)	29.3 (23.9 - 37.3)	524,637 (427,308 - 671,667)	-74.1 (-81.9 - -63.5)	2.4 (1.8 - 3.2)	41,723 (31,472 - 55,245)
China	-80.6 (-85.0 - -75.8)	43.3 (37.0 - 51.8)	709,315 (606,462 - 849,843)	-84.4 (-88.1 - -79.7)	12.9 (10.2 - 15.9)	196,016 (156,779 - 240,061)	-78.1 (-85.1 - -69.3)	28.6 (23.3 - 36.5)	484,305 (394,243 - 620,462)	-74.9 (-82.6 - -64.5)	2.4 (1.8 - 3.2)	38,835 (29,186 - 51,650)
North Korea	-12.4 (-36.9 - 18.5)	135.0 (110.1 - 168.9)	35,442 (28,731 - 44,796)	-19.4 (-38.1 - 2.4)	39.9 (32.4 - 48.8)	9,485 (7,750 - 11,842)	-9.3 (-42.5 - 37.8)	90.4 (68.9 - 123.6)	24,666 (18,823 - 33,689)	13.2 (-25.1 - 61.8)	6.3 (4.8 - 8.4)	1,730 (1,279 - 2,335)
Taiwan (province of China)	-58.0 (-65.7 - -49.4)	46.8 (39.7 - 54.5)	17,527 (14,765 - 20,505)	-42.2 (-59.3 - -22.7)	26.4 (19.7 - 33.4)	10,036 (7,428 - 12,799)	-68.4 (-74.7 - -60.7)	19.5 (16.8 - 22.9)	7,215 (6,160 - 8,505)	-80.6 (-87.2 - -71.2)	1.5 (1.1 - 1.9)	486 (370 - 646)
Oceania	-26.2 (-37.8 - -12.8)	451.4 (392.9 - 518.5)	35,884 (31,211 - 41,497)	-30.1 (-40.4 - -18.1)	201.8 (173.1 - 237.8)	17,186 (14,495 - 20,494)	-21.6 (-40.8 - 2.3)	237.5 (193.1 - 289.1)	17,126 (14,041 - 20,655)	-29.0 (-47.1 - -5.7)	20.1 (16.0 - 24.9)	2,126 (1,685 - 2,670)
American Samoa	-25.2 (-38.1 - -11.9)	161.9 (141.9 - 183.8)	60 (53 - 69)	-18.2 (-33.0 - -2.5)	71.8 (60.8 - 82.5)	26 (22 - 30)	-28.8 (-46.3 - -9.0)	89.0 (72.8 - 107.8)	33 (27 - 40)	-38.0 (-55.7 - -15.1)	5.6 (4.4 - 7.0)	2 (2 - 3)
Federated States of Micronesia	-38.7 (-49.6 - -24.1)	279.9 (239.2 - 331.0)	184 (155 - 221)	-43.4 (-56.5 - -18.2)	102.9 (79.1 - 151.4)	71 (53 - 115)	-35.1 (-50.2 - -18.3)	172.2 (139.7 - 208.2)	107 (86 - 130)	-39.6 (-58.8 - -16.2)	10.9 (7.7 - 14.5)	9 (6 - 12)
Fiji	-13.5 (-32.4 - 11.0)	247.0 (203.7 - 308.0)	1,630 (1,338 - 2,034)	-25.5 (-39.0 - -9.8)	92.8 (78.5 - 107.5)	602 (514 - 699)	-3.7 (-34.2 - 35.6)	157.7 (116.4 - 215.6)	1,041 (767 - 1,421)	-25.1 (-46.7 - 1.5)	6.2 (4.8 - 7.9)	49 (38 - 63)
Guam	-17.0 (-30.0 - -2.7)	87.0 (77.2 - 99.0)	145 (129 - 164)	-15.5 (-34.3 - 5.5)	39.2 (32.2 - 46.0)	65 (53 - 76)	-17.6 (-35.5 - 1.9)	46.3 (39.5 - 54.4)	78 (67 - 91)	-19.2 (-43.7 - 11.5)	3.3 (2.6 - 4.3)	6 (4 - 7)
Kiribati	-27.3 (-39.7 - -14.1)	530.9 (459.8 - 618.6)	380 (328 - 441)	-40.6 (-51.7 - -28.2)	206.0 (170.6 - 250.7)	149 (126 - 177)	-14.8 (-34.5 - 5.6)	320.9 (262.4 - 385.8)	224 (186 - 267)	-18.0 (-41.8 - 13.2)	13.1 (10.0 - 17.2)	13 (10 - 17)
Marshall Islands	-29.8 (-41.3 - -16.3)	307.6 (264.6 - 357.1)	98 (85 - 115)	-34.4 (-44.9 - -21.6)	112.3 (93.9 - 142.8)	38 (31 - 48)	-26.1 (-42.6 - -7.1)	189.5 (157.4 - 227.0)	57 (47 - 68)	-29.4 (-49.2 - -4.6)	13.2 (10.2 - 16.8)	6 (4 - 7)
Northern Mariana Islands	-37.4 (-49.6 - -23.4)	74.9 (65.0 - 87.1)	31 (27 - 36)	-34.5 (-50.7 - -14.8)	32.9 (26.1 - 39.7)	13 (11 - 16)	-38.4 (-53.2 - -20.1)	40.8 (34.0 - 49.6)	17 (14 - 21)	-46.4 (-63.0 - -26.4)	2.7 (2.1 - 3.5)	1 (1 - 2)
Papua New Guinea	-30.1 (-42.4 - -16.0)	540.7 (458.9 - 636.7)	28,839 (24,735 - 33,939)	-33.7 (-44.4 - -21.5)	247.1 (207.9 - 299.9)	14,096 (11,734 - 17,122)	-25.3 (-46.5 - 1.4)	278.8 (219.0 - 353.7)	13,371 (10,705 - 16,511)	-35.1 (-52.8 - -13.1)	23.4 (18.3 - 29.2)	1,775 (1,380 - 2,248)
Samoa	-25.7 (-38.5 - -9.4)	180.7 (155.2 - 208.2)	241 (208 - 277)	-26.9 (-41.4 - -10.0)	68.5 (56.4 - 86.4)	95 (78 - 120)	-24.0 (-41.9 - -3.0)	110.2 (90.2 - 133.4)	142 (117 - 171)	-35.7 (-54.2 - -14.0)	6.4 (4.8 - 8.4)	10 (7 - 13)
Solomon Islands	-27.0 (-38.7 - -14.0)	407.9 (360.5 - 460.4)	1,439 (1,264 - 1,636)	-31.7 (-43.8 - -19.0)	201.0 (171.2 - 229.1)	736 (620 - 860)	-20.8 (-38.6 - 0.8)	190.2 (158.1 - 227.2)	623 (519 - 740)	-24.9 (-49.1 - 5.5)	22.3 (16.6 - 29.1)	96 (72 - 123)
Tonga	-31.0 (-43.7 - -16.4)	206.0 (175.8 - 242.4)	162 (139 - 191)	-34.8 (-46.8 - -23.1)	80.5 (67.1 - 94.0)	64 (54 - 75)	-27.5 (-46.3 - -5.0)	122.8 (98.7 - 153.8)	95 (77 - 118)	-36.5 (-54.1 - -15.1)	6.2 (4.9 - 7.7)	5 (4 - 7)
Vanuatu	-19.8 (-36.0 - 0.0)	389.4 (318.2 - 466.9)	696 (557 - 863)	-29.3 (-43.7 - -11.1)	145.9 (116.2 - 185.8)	282 (219 - 368)	-12.5 (-34.4 - 13.2)	235.9 (184.5 - 288.6)	392 (302 - 490)	-8.9 (-36.7 - 28.1)	17.3 (11.7 - 24.3)	38 (26 - 54)
Southeast Asia	-55.3 (-60.7 - -49.2)	181.1 (164.6 - 200.1)	965,385 (877,884 - 1,066,136)	-57.9 (-65.2 - -50.5)	91.0 (78.5 - 103.1)	481,445 (419,444 - 544,407)	-50.4 (-59.6 - -39.2)	86.0 (73.9 - 99.9)	458,385 (395,645 - 532,624)	-65.1 (-73.8 - -53.3)	6.4 (5.2 - 8.1)	38,704 (31,074 - 48,341)
Cambodia	-59.8 (-65.2 - -54.0)	309.1 (277.5 - 347.6)	33,778 (30,004 - 37,946)	-62.9 (-68.9 - -56.9)	158.4 (136.5 - 180.9)	17,458 (14,959 - 19,991)	-54.3 (-62.9 - -44.5)	131.9 (114.0 - 154.5)	13,910 (12,084 - 16,214)	-60.1 (-72.0 - -45.3)	22.4 (15.3 - 31.2)	2,789 (1,916 - 3,804)
Indonesia	-51.3 (-57.5 - -44.0)	231.5 (203.7 - 259.8)	422,140 (375,836 - 471,454)	-58.8 (-66.3 - -50.3)	111.1 (90.3 - 130.7)	200,420 (168,039 - 233,914)	-38.4 (-50.6 - -22.9)	116.9 (99.2 - 137.4)	213,583 (182,293 - 250,525)	-64.4 (-74.9 - -50.3)	6.6 (5.2 - 8.5)	14,044 (11,231 - 17,677)
Laos	-63.9 (-69.8 - -57.6)	305.6 (266.8 - 347.4)	14,952 (12,968 - 17,149)	-68.3 (-74.3 - -61.0)	139.3 (115.1 - 166.2)	7,449 (6,146 - 9,191)	-57.6 (-66.5 - -46.8)	158.7 (134.5 - 186.6)	6,998 (5,954 - 8,209)	-67.6 (-76.3 - -55.9)	12.4 (9.6 - 15.8)	708 (537 - 909)
Malaysia	-36.4 (-52.0 - -22.2)	143.8 (115.4 - 169.7)	31,997 (25,707 - 37,391)	-7.3 (-43.3 - 22.5)	95.3 (68.2 - 118.9)	21,027 (15,077 - 26,130)	-59.7 (-67.7 - -49.8)	47.5 (40.5 - 56.3)	10,581 (9,126 - 12,484)	-63.2 (-74.7 - -48.9)	3.9 (3.0 - 5.1)	1,090 (845 - 1,436)

Maldives	-83.6 (-86.3 - -80.5)	45.4 (40.5 - 52.4)	126 (113 - 143)	-81.4 (-85.4 - -77.1)	17.3 (14.6 - 20.2)	49 (42 - 57)	-84.4 (-88.1 - -79.9)	27.4 (23.2 - 33.2)	74 (63 - 88)	-84.9 (-89.2 - -78.3)	1.8 (1.4 - 2.6)	6 (5 - 9)
Mauritius	-45.6 (-55.9 - -33.9)	86.2 (73.4 - 102.9)	1,244 (1,047 - 1,507)	-53.1 (-63.6 - -41.4)	27.8 (22.6 - 32.8)	373 (302 - 444)	-40.9 (-56.3 - -22.0)	57.0 (44.9 - 73.3)	851 (663 - 1,107)	-44.8 (-61.8 - -23.6)	3.0 (2.3 - 3.8)	44 (34 - 56)
Myanmar	-64.2 (-70.0 - -56.9)	277.9 (243.5 - 319.9)	115,652 (101,106 - 132,711)	-68.2 (-74.1 - -61.3)	126.8 (109.2 - 145.1)	53,545 (45,986 - 61,351)	-59.1 (-68.7 - -47.3)	142.6 (117.4 - 173.5)	58,033 (48,135 - 70,153)	-64.4 (-74.8 - -50.8)	11.4 (8.7 - 14.5)	5,322 (4,079 - 6,779)
Philippines	-28.8 (-41.0 - -15.2)	242.5 (208.5 - 277.0)	161,259 (139,929 - 185,364)	-32.6 (-47.2 - -16.0)	143.0 (117.7 - 167.8)	92,732 (77,837 - 108,761)	-19.2 (-36.2 - 0.7)	96.0 (79.9 - 115.7)	65,089 (54,699 - 77,384)	-45.7 (-58.6 - -30.2)	5.6 (4.5 - 6.9)	4,880 (3,968 - 5,994)
Sri Lanka	-70.4 (-76.3 - -63.5)	69.5 (57.3 - 83.0)	14,865 (12,159 - 17,866)	-72.0 (-79.7 - -61.6)	30.8 (23.7 - 39.3)	6,541 (5,056 - 8,234)	-63.8 (-72.8 - -52.8)	36.4 (28.5 - 46.0)	7,830 (6,068 - 9,881)	-87.9 (-91.8 - -82.6)	3.6 (2.7 - 4.8)	805 (602 - 1,073)
Seychelles	-43.4 (-52.1 - -34.4)	133.6 (115.8 - 151.0)	129 (113 - 146)	-39.3 (-53.7 - -22.8)	78.4 (61.2 - 93.7)	75 (59 - 89)	-48.4 (-57.0 - -38.0)	55.2 (48.6 - 63.4)	54 (48 - 63)	-51.2 (-64.9 - -33.8)	3.6 (2.8 - 4.6)	4 (3 - 5)
Thailand	-59.8 (-67.7 - -52.2)	87.3 (74.2 - 100.9)	76,970 (64,908 - 89,371)	-45.3 (-64.2 - -29.2)	50.8 (39.1 - 61.9)	44,330 (33,857 - 54,571)	-70.0 (-75.5 - -63.0)	34.3 (29.4 - 40.1)	30,996 (26,232 - 36,309)	-72.1 (-80.1 - -61.8)	3.4 (2.6 - 4.3)	2,771 (2,161 - 3,547)
Timor-Leste	-61.2 (-67.5 - -54.1)	229.8 (199.2 - 262.6)	2,040 (1,765 - 2,359)	-65.9 (-72.6 - -54.7)	105.0 (86.0 - 139.4)	1,004 (826 - 1,330)	-51.6 (-62.7 - -39.3)	121.8 (101.3 - 144.2)	999 (835 - 1,173)	-81.5 (-87.3 - -74.8)	6.7 (4.7 - 8.9)	66 (44 - 88)
Vietnam	-61.4 (-67.8 - -53.5)	104.5 (91.5 - 119.9)	88,651 (77,605 - 101,988)	-63.4 (-71.7 - -53.9)	41.9 (34.9 - 49.6)	35,810 (29,818 - 42,486)	-59.4 (-68.4 - -48.3)	58.0 (48.2 - 70.2)	48,474 (40,231 - 58,335)	-59.3 (-72.8 - -41.2)	6.7 (5.0 - 8.9)	6,123 (4,653 - 8,133)
Sub-Saharan Africa	-47.0 (-52.0 - -41.4)	470.2 (430.9 - 514.4)	3,492,058 (3,180,006 - 3,818,514)	-49.0 (-55.5 - -41.7)	308.7 (273.2 - 349.8)	2,498,546 (2,230,269 - 2,787,405)	-39.9 (-48.9 - -30.0)	151.2 (132.9 - 171.0)	905,957 (793,853 - 1,021,394)	-56.7 (-64.9 - -46.3)	16.1 (13.5 - 19.1)	114,375 (96,741 - 133,719)
Central sub-Saharan Africa	-41.6 (-48.8 - -33.1)	553.8 (501.3 - 627.1)	453,447 (404,474 - 506,559)	-44.1 (-52.8 - -33.5)	355.5 (309.4 - 420.1)	315,610 (274,412 - 363,262)	-35.3 (-46.1 - -23.0)	184.6 (159.4 - 213.2)	122,443 (103,510 - 144,330)	-39.9 (-54.1 - -21.4)	19.7 (15.9 - 24.1)	18,440 (14,762 - 22,903)
Angola	-57.1 (-63.9 - -49.8)	482.3 (421.8 - 546.3)	85,518 (73,704 - 99,534)	-56.6 (-65.4 - -47.5)	313.1 (264.7 - 368.1)	59,986 (49,975 - 71,036)	-53.9 (-63.5 - -40.5)	160.0 (135.3 - 186.6)	23,231 (19,357 - 27,745)	-75.1 (-81.5 - -65.2)	14.9 (11.6 - 19.1)	2,892 (2,229 - 3,769)
Central African Republic	-12.3 (-25.6 - 2.0)	1,081.4 (942.9 - 1,238.3)	35,644 (30,521 - 41,545)	-16.1 (-35.2 - 3.4)	684.7 (571.0 - 804.0)	23,953 (19,811 - 28,679)	-12.4 (-28.1 - 9.2)	328.7 (275.1 - 390.0)	8,748 (7,082 - 10,697)	65.0 (20.9 - 140.6)	76.9 (61.8 - 93.5)	3,128 (2,484 - 3,805)
Congo (Brazzaville)	-44.3 (-53.2 - -34.4)	515.3 (444.4 - 593.4)	15,727 (13,522 - 18,414)	-44.5 (-55.6 - -33.0)	333.2 (277.5 - 396.4)	11,005 (9,054 - 13,289)	-42.2 (-53.7 - -28.8)	173.4 (144.6 - 204.7)	4,326 (3,579 - 5,149)	-52.8 (-66.4 - -33.5)	15.2 (11.5 - 19.6)	549 (404 - 725)
DR Congo	-36.9 (-46.3 - -25.3)	559.6 (492.5 - 647.8)	310,088 (267,665 - 359,172)	-40.8 (-51.2 - -27.3)	358.9 (305.7 - 434.4)	216,221 (183,044 - 256,976)	-28.3 (-42.1 - -13.4)	188.1 (157.9 - 222.9)	84,266 (68,985 - 101,242)	-23.6 (-44.6 - 2.3)	18.6 (14.7 - 23.4)	11,651 (9,129 - 14,810)
Equatorial Guinea	-71.6 (-79.2 - -62.6)	336.2 (245.6 - 448.9)	2,611 (1,849 - 3,557)	-68.7 (-77.9 - -57.9)	236.1 (167.0 - 320.3)	2,023 (1,376 - 2,812)	-75.2 (-82.2 - -65.8)	96.9 (67.6 - 133.2)	547 (365 - 783)	-83.6 (-89.7 - -75.5)	7.3 (4.8 - 10.9)	62 (39 - 93)
Gabon	-47.0 (-54.4 - -38.1)	331.8 (295.3 - 374.0)	3,858 (3,373 - 4,359)	-49.5 (-58.9 - -38.9)	198.8 (168.8 - 233.1)	2,423 (2,029 - 2,889)	-41.5 (-52.3 - -29.2)	125.9 (108.3 - 147.5)	1,324 (1,132 - 1,537)	-49.5 (-64.6 - -29.2)	12.0 (9.0 - 15.5)	159 (116 - 211)
Eastern sub-Saharan Africa	-55.7 (-60.0 - -51.2)	474.9 (437.1 - 516.5)	1,206,487 (1,116,649 - 1,300,234)	-58.2 (-63.7 - -51.8)	295.0 (262.3 - 334.7)	818,947 (738,427 - 906,235)	-47.3 (-55.1 - -38.3)	166.0 (145.6 - 185.5)	349,701 (306,990 - 391,690)	-67.9 (-74.1 - -60.5)	18.7 (15.5 - 22.2)	45,623 (38,837 - 53,032)
Burundi	-50.0 (-57.9 - -41.3)	618.4 (529.4 - 707.5)	41,731 (35,757 - 48,533)	-49.4 (-59.1 - -38.4)	393.3 (323.7 - 466.8)	28,620 (23,953 - 33,931)	-51.3 (-60.6 - -39.5)	207.6 (175.4 - 242.9)	11,896 (9,825 - 14,352)	-46.8 (-60.6 - -28.4)	23.5 (18.4 - 30.4)	1,468 (1,157 - 1,861)
Comoros	-51.6 (-58.8 - -43.2)	359.3 (314.0 - 409.9)	1,719 (1,497 - 1,983)	-51.3 (-59.7 - -41.8)	194.3 (162.4 - 232.1)	957 (801 - 1,150)	-51.7 (-61.0 - -41.0)	154.4 (129.7 - 181.0)	705 (596 - 831)	-53.5 (-66.3 - -37.5)	14.6 (11.1 - 18.8)	74 (56 - 97)
Djibouti	-42.8 (-56.5 - -25.8)	356.8 (273.3 - 453.4)	2,432 (1,815 - 3,202)	-41.6 (-57.6 - -23.2)	201.5 (151.1 - 256.7)	1,518 (1,119 - 1,977)	-42.1 (-57.6 - -22.4)	145.4 (109.8 - 193.7)	841 (607 - 1,177)	-58.6 (-71.1 - -42.0)	14.1 (10.1 - 20.0)	98 (68 - 141)
Eritrea	-71.8 (-76.3 - -66.8)	626.6 (530.5 - 724.5)	19,562 (16,457 - 23,088)	-53.2 (-63.6 - -41.8)	376.1 (304.9 - 454.5)	12,579 (10,174 - 15,312)	-52.1 (-63.0 - -38.7)	231.9 (186.9 - 274.5)	6,265 (5,104 - 7,575)	-97.3 (-98.0 - -96.5)	25.0 (19.6 - 32.2)	870 (674 - 1,130)
Ethiopia	-68.8 (-72.4 - -64.7)	420.2 (384.6 - 458.6)	268,383 (245,079 - 293,270)	-70.7 (-75.5 - -64.3)	254.2 (223.0 - 295.7)	176,253 (157,082 - 196,243)	-62.3 (-68.6 - -54.6)	152.8 (134.0 - 170.9)	84,011 (72,701 - 95,229)	-78.4 (-83.3 - -72.3)	16.9 (13.6 - 20.8)	9,724 (7,982 - 11,648)

Kenya	-29.8 (-37.5 - -21.9)	409.9 (368.2 - 452.9)	117,126 (104,804 - 130,053)	-31.6 (-41.8 - -20.5)	267.7 (229.6 - 308.8)	83,288 (71,437 - 96,859)	-25.9 (-37.9 - -12.5)	133.2 (113.9 - 153.4)	31,098 (26,850 - 35,513)	-26.7 (-47.9 - -3.0)	12.7 (10.3 - 15.6)	3,549 (2,892 - 4,338)
Madagascar	-34.8 (-45.3 - -23.7)	524.8 (449.7 - 608.9)	92,124 (76,341 - 109,889)	-35.7 (-46.1 - -23.7)	301.3 (253.6 - 360.7)	60,233 (48,965 - 73,726)	-32.3 (-48.3 - -12.7)	215.9 (173.7 - 264.4)	30,347 (23,724 - 39,002)	-42.1 (-56.6 - -25.6)	15.6 (12.3 - 19.8)	2,414 (1,904 - 3,021)
Malawi	-56.1 (-62.4 - -49.3)	506.6 (451.2 - 567.4)	59,903 (52,460 - 68,640)	-60.4 (-68.8 - -52.1)	331.6 (283.6 - 385.9)	42,232 (35,844 - 48,997)	-42.5 (-55.5 - -18.8)	164.0 (139.0 - 190.6)	16,460 (13,896 - 19,435)	-47.7 (-65.3 - -11.8)	15.0 (12.0 - 18.8)	1,534 (1,223 - 1,915)
Mozambique	-42.6 (-50.5 - -33.1)	638.5 (552.8 - 736.3)	120,126 (102,270 - 140,383)	-40.4 (-50.3 - -28.9)	425.5 (353.9 - 502.5)	89,109 (73,101 - 107,535)	-44.6 (-55.2 - -31.4)	197.8 (168.7 - 231.4)	28,186 (23,625 - 32,823)	-56.5 (-66.1 - -43.9)	20.9 (16.8 - 25.9)	3,490 (2,838 - 4,255)
Rwanda	-69.0 (-73.7 - -64.0)	380.1 (337.0 - 430.7)	29,022 (25,638 - 33,107)	-70.5 (-76.0 - -64.0)	225.8 (192.1 - 267.8)	18,495 (15,713 - 21,849)	-63.9 (-70.8 - -56.1)	142.9 (121.3 - 165.0)	9,598 (8,186 - 11,239)	-78.9 (-84.4 - -71.8)	15.7 (11.9 - 20.8)	1,166 (883 - 1,564)
Somalia	-40.2 (-52.3 - -26.2)	744.7 (599.7 - 927.7)	82,919 (65,205 - 106,881)	-45.8 (-61.9 - -28.0)	419.8 (328.1 - 532.3)	53,637 (40,816 - 69,868)	-31.1 (-50.4 - -3.2)	271.6 (213.6 - 339.7)	22,275 (16,779 - 28,605)	-17.7 (-38.9 - 11.4)	60.3 (48.1 - 74.5)	7,474 (6,019 - 9,118)
South Sudan	-28.6 (-40.7 - -13.3)	706.2 (579.1 - 855.8)	54,817 (45,593 - 66,505)	-31.8 (-48.3 - -12.5)	441.0 (351.0 - 547.4)	38,166 (30,738 - 46,816)	-28.2 (-46.9 - -2.8)	215.4 (168.6 - 269.4)	12,499 (9,714 - 16,003)	26.3 (-7.8 - 73.5)	54.9 (43.9 - 65.7)	4,358 (3,452 - 5,259)
Tanzania	-53.4 (-59.7 - -46.7)	399.8 (358.1 - 444.8)	149,449 (130,949 - 172,317)	-59.1 (-66.9 - -51.3)	245.8 (210.2 - 285.4)	97,257 (82,106 - 115,016)	-39.4 (-50.4 - -24.9)	146.0 (124.9 - 169.3)	49,002 (41,107 - 58,441)	-42.5 (-58.8 - -17.3)	12.6 (10.0 - 15.8)	4,370 (3,476 - 5,429)
Uganda	-62.9 (-70.3 - -55.0)	441.9 (395.0 - 498.9)	109,604 (96,445 - 123,987)	-69.9 (-77.1 - -61.6)	275.6 (236.2 - 320.5)	75,914 (64,399 - 88,633)	-38.8 (-50.3 - -25.4)	155.2 (133.4 - 178.7)	31,013 (26,208 - 36,268)	-43.0 (-58.1 - -24.8)	15.9 (12.5 - 20.7)	3,350 (2,647 - 4,288)
Zambia	-48.4 (-56.0 - -40.3)	533.2 (475.0 - 600.4)	56,755 (48,834 - 66,028)	-51.0 (-60.0 - -39.8)	342.3 (294.0 - 400.9)	40,173 (33,864 - 47,976)	-42.0 (-52.5 - -29.0)	177.8 (154.3 - 204.4)	15,230 (12,832 - 17,927)	-46.1 (-60.8 - -26.8)	17.6 (14.2 - 21.9)	1,655 (1,358 - 2,031)
Southern sub-Saharan Africa	-11.2 (-25.4 - 4.7)	330.4 (285.8 - 380.3)	208,310 (177,537 - 243,598)	-3.2 (-25.0 - 21.5)	219.9 (177.6 - 267.8)	146,086 (115,494 - 181,087)	-19.8 (-33.8 - -2.8)	103.5 (89.4 - 119.8)	56,814 (49,368 - 64,901)	-43.8 (-58.9 - -25.1)	10.8 (8.7 - 13.5)	7,564 (6,043 - 9,512)
Botswana	-44.0 (-56.1 - -30.2)	258.1 (212.8 - 314.2)	4,079 (3,246 - 5,228)	-43.3 (-59.8 - -23.9)	169.6 (128.4 - 222.7)	2,928 (2,138 - 4,069)	-43.6 (-56.4 - -27.7)	85.6 (71.1 - 103.9)	1,082 (910 - 1,302)	-55.6 (-68.3 - -39.7)	5.9 (4.6 - 7.5)	108 (83 - 136)
Lesotho	20.9 (0.8 - 44.6)	695.0 (591.3 - 814.2)	9,988 (8,405 - 11,851)	30.1 (4.2 - 63.5)	443.2 (361.0 - 535.5)	6,895 (5,512 - 8,475)	7.9 (-18.2 - 40.0)	233.0 (186.2 - 290.5)	2,739 (2,208 - 3,346)	14.1 (-23.3 - 57.8)	25.9 (19.4 - 33.9)	432 (322 - 570)
Namibia	-42.3 (-53.1 - -30.3)	327.2 (274.8 - 388.7)	5,626 (4,650 - 6,800)	-34.1 (-50.5 - -15.8)	214.7 (169.7 - 269.7)	3,914 (3,059 - 5,022)	-52.1 (-62.1 - -39.6)	107.0 (88.0 - 129.7)	1,584 (1,308 - 1,904)	-59.6 (-70.9 - -44.4)	9.7 (7.4 - 12.7)	184 (138 - 246)
South Africa	-14.0 (-29.3 - 3.6)	283.3 (238.3 - 334.8)	134,680 (110,363 - 162,718)	0.2 (-25.2 - 30.9)	192.8 (146.8 - 244.7)	96,032 (71,651 - 123,917)	-29.0 (-41.8 - -13.7)	84.3 (72.4 - 98.4)	35,058 (30,124 - 40,655)	-56.4 (-68.9 - -40.7)	8.9 (7.0 - 11.3)	4,721 (3,671 - 6,072)
Swaziland (eSwatini)	-7.8 (-25.5 - 11.1)	485.2 (407.6 - 574.8)	3,606 (2,985 - 4,267)	4.5 (-19.1 - 30.3)	304.7 (245.3 - 370.5)	2,526 (2,003 - 3,095)	-22.7 (-42.2 - 2.2)	168.7 (133.2 - 209.8)	955 (778 - 1,157)	-24.9 (-48.7 - 6.4)	16.8 (12.3 - 22.1)	152 (111 - 202)
Zimbabwe	8.1 (-19.6 - 34.9)	537.1 (470.7 - 608.6)	50,295 (44,185 - 56,988)	-3.3 (-34.8 - 28.7)	335.3 (281.5 - 392.6)	33,791 (28,738 - 39,114)	35.9 (9.3 - 67.8)	193.5 (164.6 - 226.1)	15,361 (12,824 - 18,113)	38.0 (0.1 - 84.4)	20.1 (15.9 - 25.1)	1,966 (1,561 - 2,467)
Western sub-Saharan Africa	-44.4 (-50.2 - -37.3)	482.6 (430.9 - 542.5)	1,623,733 (1,442,962 - 1,832,406)	-46.7 (-53.6 - -38.5)	331.8 (288.5 - 383.9)	1,217,818 (1,058,701 - 1,391,678)	-38.0 (-48.0 - -26.7)	143.7 (123.3 - 166.9)	377,002 (322,007 - 439,733)	-41.0 (-53.6 - -24.9)	14.6 (12.0 - 17.6)	42,749 (35,228 - 50,766)
Benin	-39.8 (-49.1 - -29.7)	496.5 (431.6 - 575.6)	40,074 (34,122 - 47,830)	-40.6 (-51.0 - -29.0)	318.7 (267.6 - 378.3)	27,681 (22,777 - 33,890)	-37.4 (-49.5 - -23.1)	164.8 (139.5 - 194.6)	11,264 (9,365 - 13,483)	-42.1 (-58.0 - -21.5)	21.5 (15.8 - 28.8)	1,544 (1,141 - 2,068)
Burkina Faso	-46.8 (-53.9 - -38.8)	582.7 (511.9 - 660.8)	95,694 (79,684 - 114,013)	-51.2 (-59.0 - -42.0)	391.5 (332.3 - 453.7)	70,458 (56,576 - 85,878)	-33.4 (-44.7 - -21.0)	182.0 (154.8 - 210.4)	23,384 (19,317 - 28,282)	-38.3 (-54.1 - -18.3)	21.0 (16.9 - 25.7)	2,878 (2,282 - 3,548)
Cameroon	-33.9 (-43.5 - -23.7)	529.2 (460.9 - 607.3)	91,943 (78,497 - 105,743)	-34.0 (-45.3 - -22.4)	354.4 (300.0 - 415.1)	67,011 (55,739 - 79,290)	-33.3 (-45.8 - -18.4)	166.8 (138.6 - 200.6)	23,023 (19,287 - 27,253)	-33.0 (-50.2 - -10.7)	18.2 (14.0 - 23.2)	3,074 (2,314 - 3,913)
Cape Verde	-50.5 (-57.7 - -43.2)	132.5 (116.7 - 148.3)	623 (547 - 700)	-54.6 (-63.5 - -44.3)	75.3 (62.5 - 88.0)	359 (296 - 422)	-43.3 (-54.2 - -31.2)	58.4 (50.5 - 67.7)	269 (232 - 311)	-43.5 (-58.2 - -25.9)	4.3 (3.4 - 5.3)	21 (17 - 26)

Chad	-30.7 (-39.1 - -20.2)	677.1 (601.6 - 761.5)	86,201 (74,900 - 99,328)	-33.4 (-42.7 - -22.6)	459.2 (401.4 - 528.1)	64,481 (55,136 - 75,479)	-23.1 (-37.7 - -6.3)	204.6 (173.8 - 237.3)	19,906 (16,205 - 23,970)	-30.0 (-45.2 - -12.0)	23.6 (18.7 - 29.8)	2,422 (1,939 - 2,980)
Côte d'Ivoire	-34.6 (-45.5 - -23.5)	552.8 (488.3 - 629.3)	92,448 (80,661 - 105,931)	-39.2 (-52.2 - -25.8)	354.4 (303.7 - 414.2)	64,027 (54,143 - 75,743)	-23.2 (-38.0 - -5.5)	187.7 (159.5 - 219.1)	26,309 (22,317 - 30,984)	-27.7 (-44.3 - -7.1)	20.4 (16.4 - 24.9)	3,133 (2,526 - 3,841)
The Gambia	-34.9 (-44.7 - -23.0)	426.6 (375.0 - 483.8)	5,538 (4,788 - 6,425)	-39.3 (-49.6 - -27.5)	252.5 (215.1 - 297.9)	3,447 (2,861 - 4,160)	-26.7 (-41.5 - -8.9)	167.3 (138.7 - 200.7)	1,985 (1,612 - 2,441)	-23.0 (-44.4 - 4.4)	15.8 (12.0 - 20.3)	194 (144 - 255)
Ghana	-39.7 (-48.1 - -30.4)	414.6 (370.9 - 466.7)	80,422 (70,612 - 91,546)	-42.9 (-52.8 - -32.5)	270.1 (233.1 - 311.5)	55,322 (47,203 - 64,670)	-32.6 (-46.7 - -16.7)	136.5 (115.7 - 159.2)	23,228 (19,732 - 27,352)	-29.2 (-48.5 - -5.4)	15.2 (12.0 - 18.9)	2,946 (2,341 - 3,679)
Guinea	-36.7 (-44.5 - -27.9)	623.4 (553.9 - 692.7)	54,448 (47,371 - 62,019)	-40.2 (-48.5 - -30.4)	399.9 (349.9 - 454.0)	37,645 (32,359 - 43,735)	-28.6 (-41.1 - -13.8)	212.5 (182.2 - 244.8)	15,727 (13,277 - 18,313)	-30.5 (-46.2 - -10.5)	21.2 (16.7 - 26.1)	1,654 (1,325 - 2,021)
Guinea-Bissau	-44.2 (-52.0 - -34.6)	692.5 (609.1 - 782.8)	7,932 (6,885 - 9,105)	-45.9 (-54.5 - -35.2)	423.8 (359.3 - 496.8)	5,220 (4,377 - 6,205)	-40.0 (-50.9 - -27.0)	255.6 (217.8 - 298.3)	2,516 (2,080 - 3,070)	-46.1 (-59.8 - -27.9)	25.7 (20.2 - 31.8)	286 (222 - 360)
Liberia	-58.2 (-64.1 - -51.1)	493.9 (436.3 - 567.6)	15,261 (13,164 - 17,898)	-54.0 (-61.6 - -44.2)	333.4 (284.1 - 397.4)	11,132 (9,258 - 13,443)	-45.9 (-56.1 - -33.8)	155.4 (132.5 - 180.5)	3,923 (3,260 - 4,653)	-92.8 (-94.6 - -90.2)	13.1 (10.1 - 16.6)	369 (283 - 473)
Mali	-54.8 (-60.8 - -48.1)	513.8 (451.6 - 583.4)	92,171 (78,059 - 109,770)	-56.5 (-63.4 - -48.3)	333.6 (283.8 - 400.0)	64,893 (53,674 - 78,722)	-50.4 (-60.0 - -39.2)	171.1 (143.0 - 201.1)	25,202 (20,220 - 31,491)	-52.1 (-62.6 - -37.0)	17.7 (13.9 - 22.2)	2,823 (2,284 - 3,428)
Mauritania	-57.1 (-63.5 - -50.4)	301.1 (261.3 - 343.1)	7,676 (6,618 - 8,860)	-59.0 (-66.2 - -51.3)	176.8 (147.9 - 210.0)	4,719 (3,904 - 5,646)	-53.4 (-62.4 - -43.3)	118.9 (99.9 - 138.5)	2,778 (2,357 - 3,287)	-58.2 (-69.2 - -42.2)	11.5 (8.9 - 14.7)	300 (235 - 385)
Niger	-52.1 (-58.5 - -44.5)	588.5 (516.1 - 666.5)	101,711 (85,894 - 120,109)	-55.1 (-62.1 - -46.8)	416.8 (355.2 - 488.0)	80,555 (66,692 - 96,650)	-41.8 (-52.9 - -28.8)	162.5 (135.5 - 190.8)	19,318 (15,649 - 23,242)	-48.4 (-60.8 - -32.6)	17.9 (13.9 - 23.1)	2,507 (1,968 - 3,099)
Nigeria	-48.0 (-56.4 - -37.3)	431.0 (349.1 - 532.8)	753,357 (617,579 - 919,896)	-49.6 (-58.5 - -38.7)	311.7 (250.3 - 390.0)	594,159 (481,097 - 733,512)	-43.5 (-57.0 - -25.7)	114.6 (87.3 - 152.5)	148,604 (114,181 - 192,408)	-38.7 (-56.6 - -15.2)	10.5 (8.0 - 13.9)	15,483 (11,875 - 19,642)
São Tomé and Príncipe	-39.2 (-48.0 - -30.0)	320.1 (282.3 - 362.1)	370 (328 - 414)	-45.8 (-55.0 - -36.0)	174.6 (148.4 - 200.9)	207 (177 - 236)	-27.3 (-42.2 - -10.5)	147.8 (125.5 - 174.1)	162 (138 - 189)	-28.6 (-49.9 - 0.5)	10.0 (7.3 - 13.3)	14 (10 - 18)
Senegal	-46.1 (-53.3 - -37.6)	408.6 (363.7 - 460.4)	38,905 (34,220 - 44,514)	-53.0 (-60.4 - -44.2)	238.9 (204.6 - 282.2)	24,060 (20,607 - 28,171)	-31.6 (-43.2 - -17.9)	161.1 (137.0 - 186.4)	13,884 (11,781 - 16,125)	-33.2 (-49.6 - -11.6)	15.6 (12.2 - 20.0)	1,470 (1,145 - 1,941)
Sierra Leone	-40.1 (-48.9 - -30.0)	611.9 (529.6 - 694.6)	35,466 (29,952 - 41,931)	-42.3 (-51.9 - -31.4)	416.5 (350.6 - 488.1)	25,628 (20,823 - 31,069)	-34.1 (-47.0 - -17.9)	188.3 (161.0 - 222.0)	9,300 (7,773 - 11,246)	-34.3 (-52.4 - -11.0)	17.4 (13.7 - 21.8)	910 (710 - 1,142)
Togo	-36.2 (-45.5 - -25.7)	486.1 (424.3 - 557.5)	23,476 (20,088 - 27,367)	-35.6 (-46.8 - -23.0)	325.0 (272.6 - 385.6)	16,804 (13,857 - 20,164)	-37.2 (-49.6 - -23.4)	153.8 (128.2 - 183.0)	6,217 (5,189 - 7,443)	-34.7 (-51.8 - -12.9)	15.5 (12.0 - 19.5)	722 (558 - 919)

Abbreviations: DR=Democratic Republic. UI=uncertainty interval. USA=United States of America.

eFigure 1. **Age-standardised sepsis-related in-hospital case fatality by year and region, both sexes, 1990–2017**

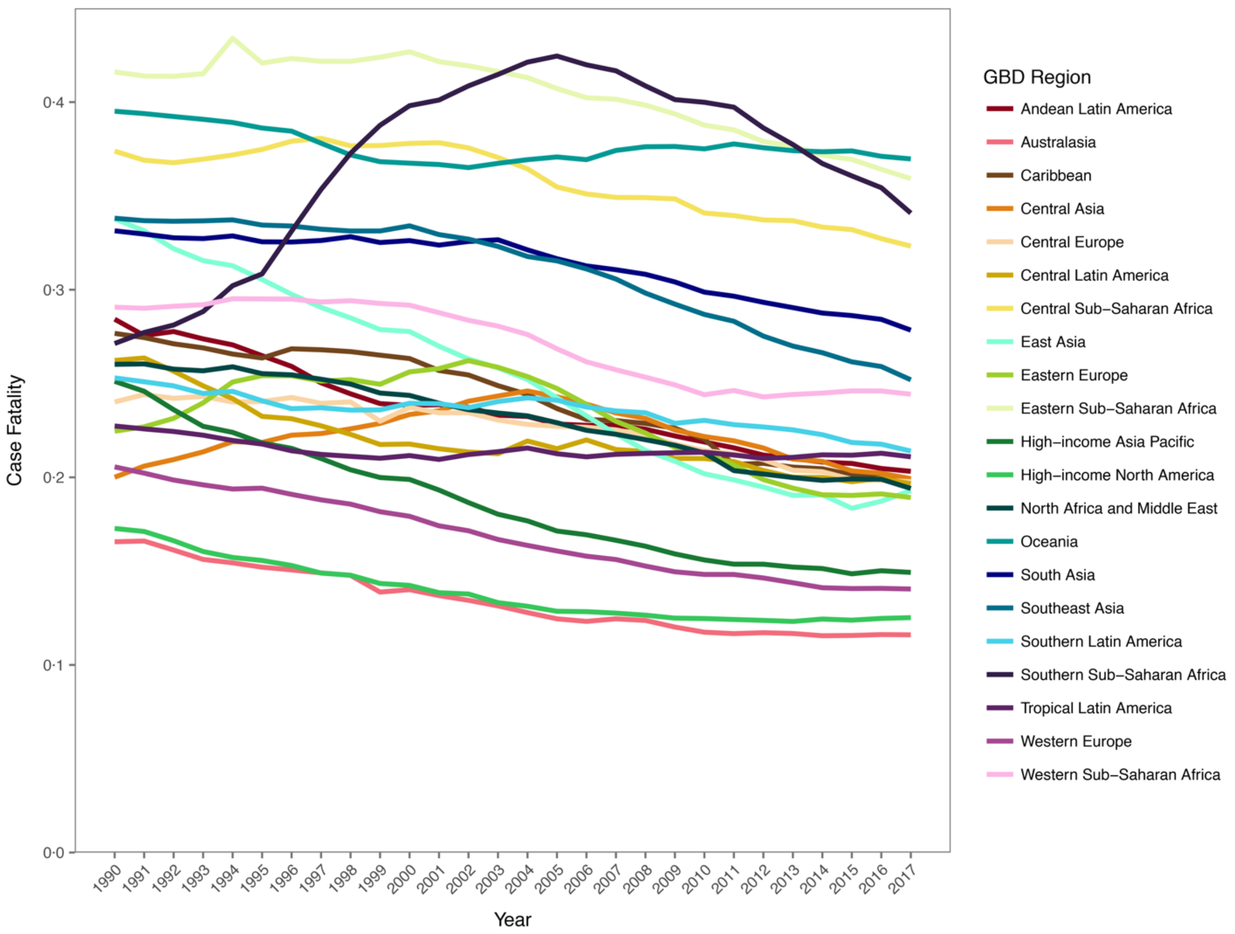

eFigure 2. Incident sepsis cases by age group and underlying cause category, both sexes, all locations, 2017

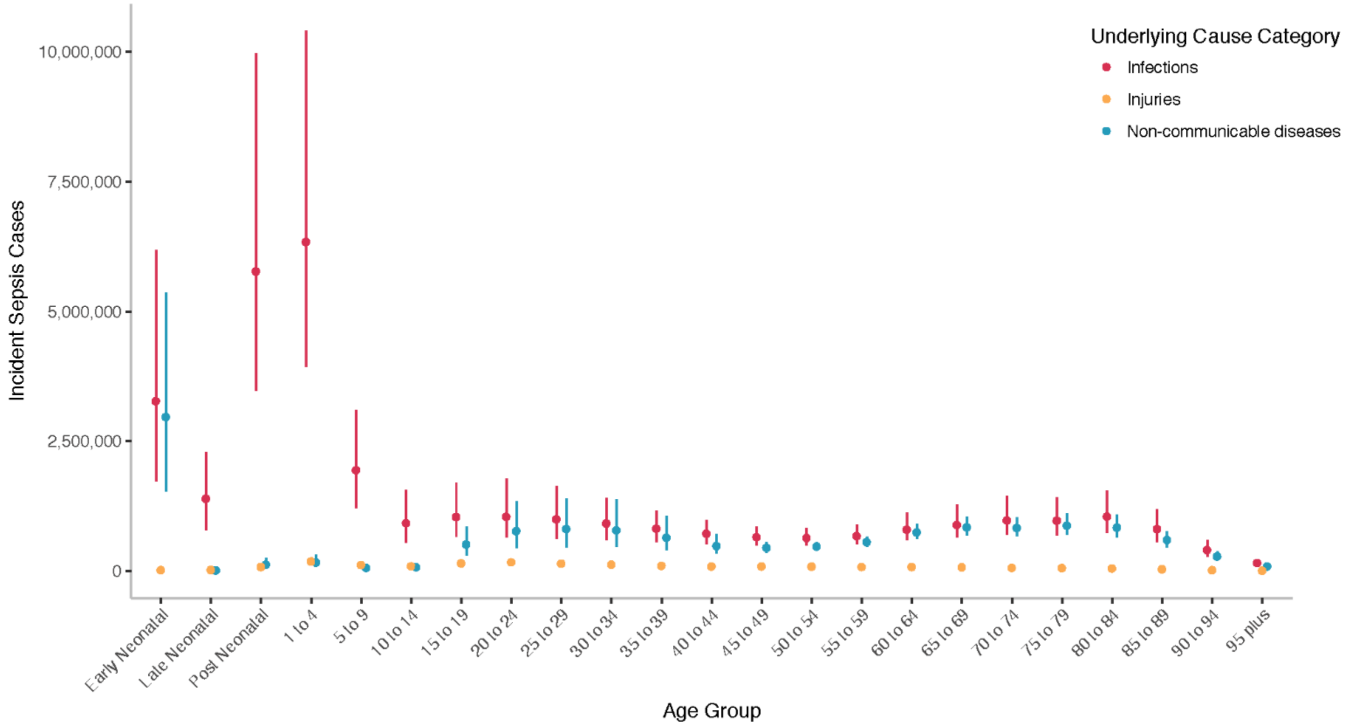

Error bars represent 95% uncertainty intervals.

eFigure 3. Age-standardised sepsis incidence rate per 100,000 population, both sexes, 1990 (panel A) and percentage of all deaths related to sepsis, age-standardised, both sexes, 1990 (panel B)

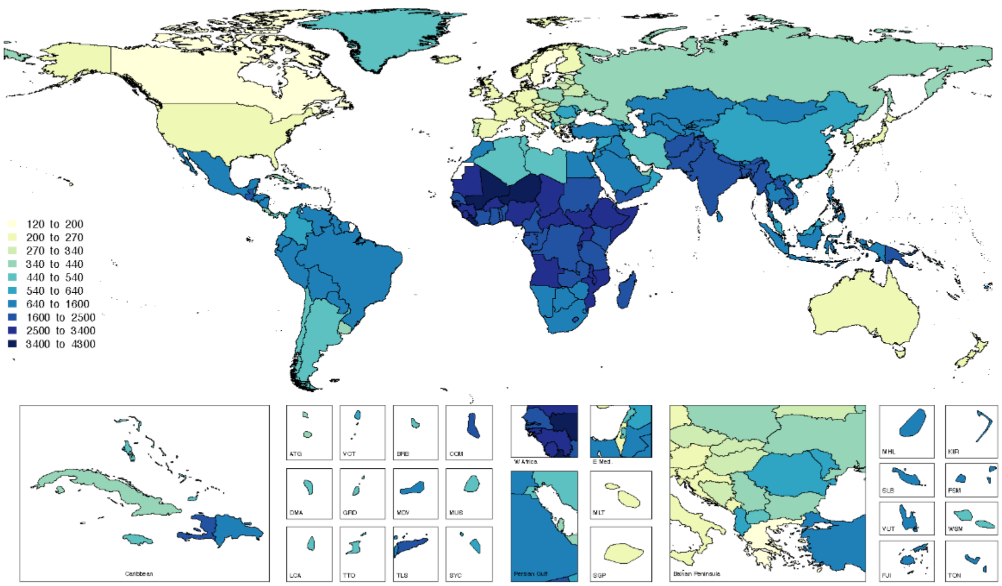

Panel A. Age-standardised sepsis incidence rate per 100,000 population, both sexes, 1990

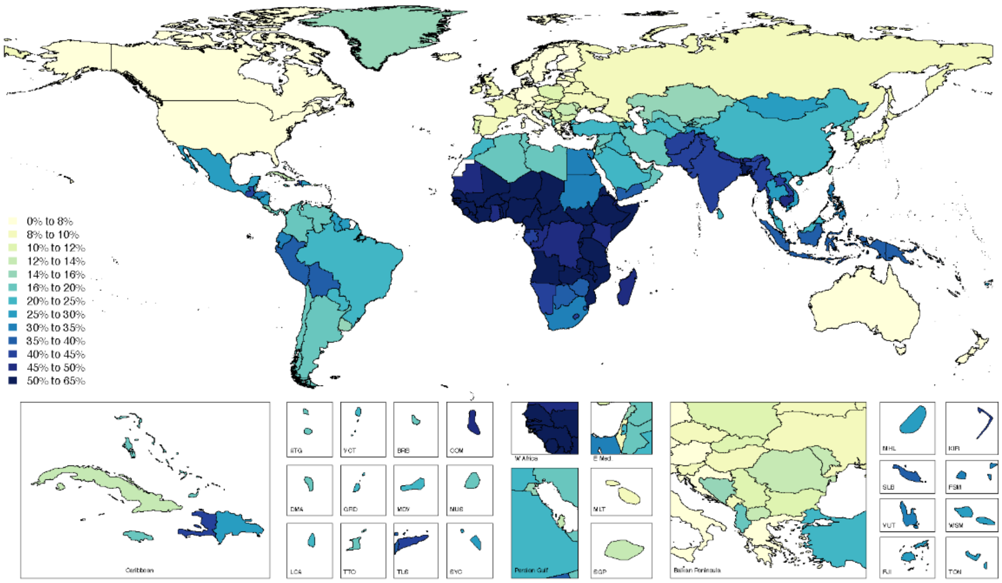

Panel B. Percentage of all deaths related to sepsis, age-standardised, both sexes, 1990

Abbreviations: ATG, Antigua and Barbuda; BRB, Barbados; COM, Comoros; DMA, Dominica; E Med, Eastern Mediterranean; FJI, Fiji; FSM, Federated States of Micronesia; GRD, Grenada; KIR, Kiribati; LCA, Saint Lucia; MDV, Maldives; MHL, Marshall Islands; MLT, Malta; MUS, Mauritius; SGP, Singapore; SLB, Solomon Islands; SYC,

Seychelles; TLS, Timor-Leste; TON, Tonga; TTO, Trinidad and Tobago; VCT, Saint Vincent and the Grenadines; VUT, Vanuatu; W Africa, West Africa; WSM, Samoa.

eFigure 4. Ranking of sepsis-related mortality for each year 1990–2017, by Global Burden of Disease region

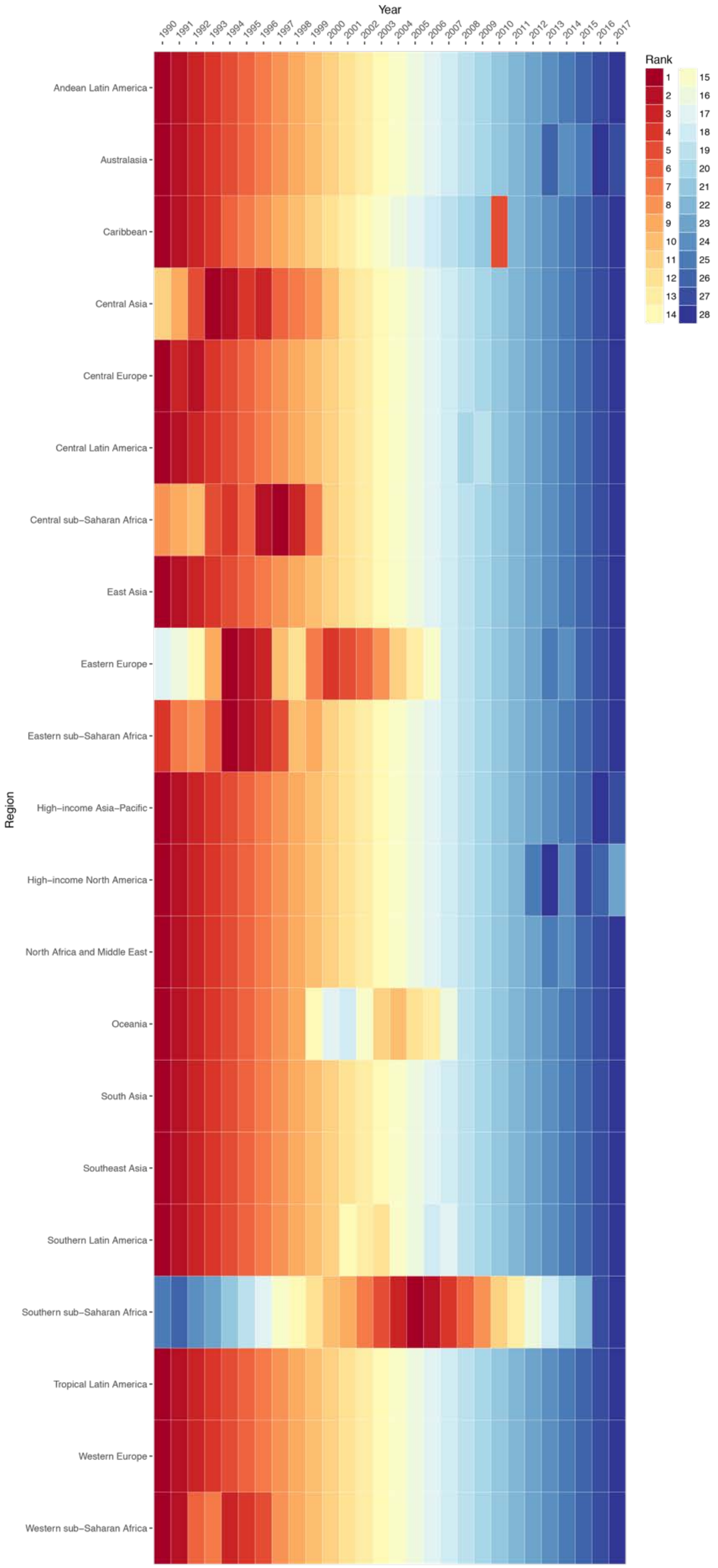

eFigure 5. Percentage of global deaths related to sepsis, age-standardised, both sexes, by underlying cause category, 1990–2017

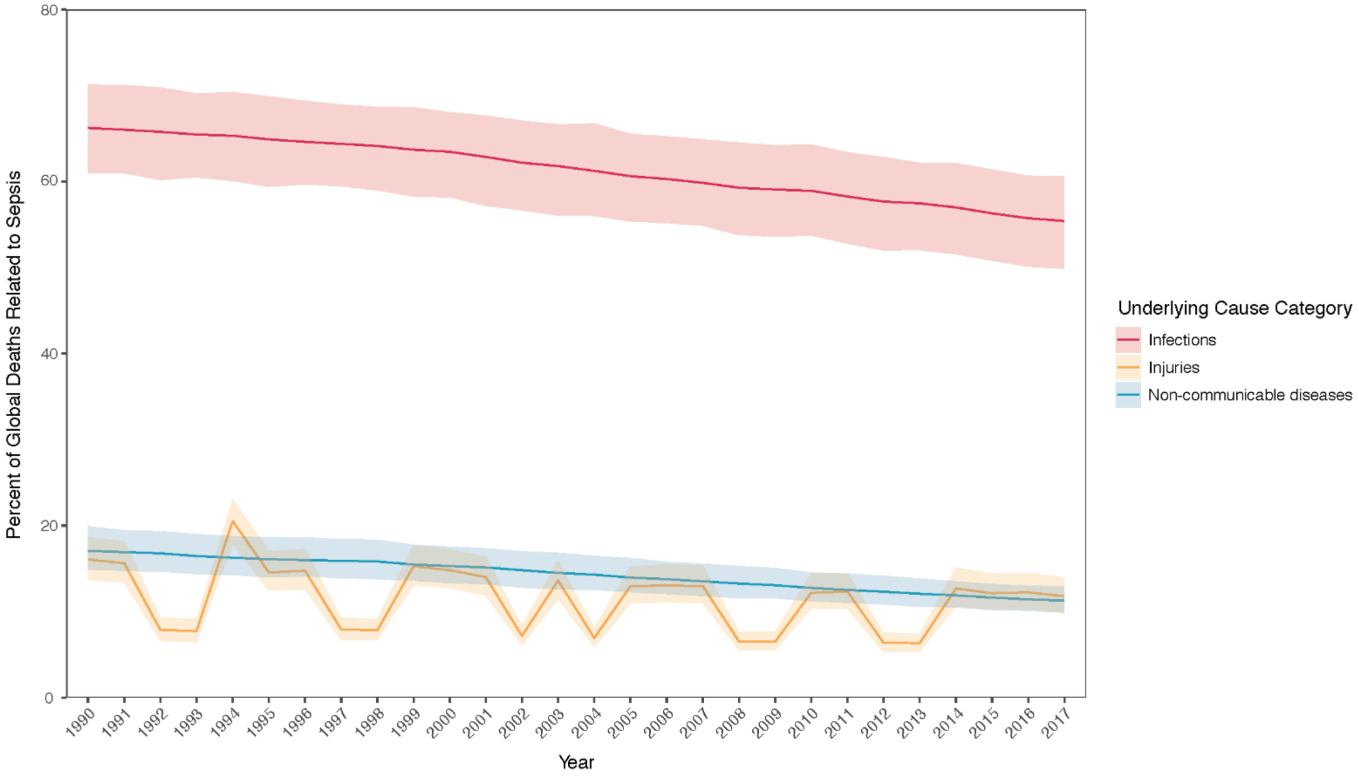

High variability in the percentage of global injury deaths which are related to sepsis over time is due to events such as wars and genocides. For example, the peak in the percentage of global injury deaths which are related to sepsis in 1994 reflects the Rwandan genocide.

eFigure 6. Percentage of sepsis deaths by underlying cause category and quintile of Socio-demographic Index, age-standardised, both sexes, 2017

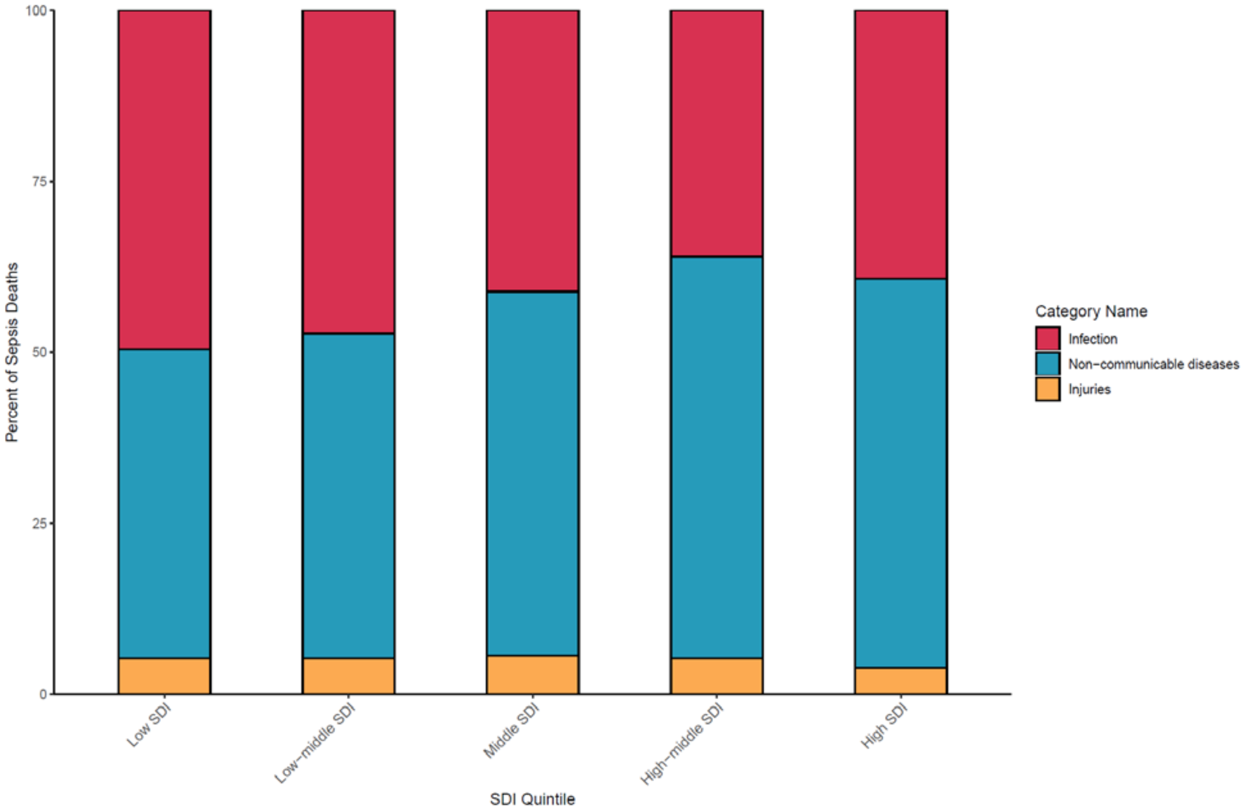

Abbreviations: SDI=Socio-demographic Index.
All 195 locations worldwide are categorised according to Socio-demographic Index (SDI), a summary measure that identifies where countries or other geographical areas sit on the spectrum of development. Expressed on a scale of 0 to 1, SDI is a composite average of the ranking of the incomes per capita, average educational attainment, and fertility rates of all areas in the GBD study. Locations are then categorised within SDI quintiles, termed low, low-middle, middle, high-middle, and high SDI. Socio-demographic index is specific by country and year, and thus the categorisation of a specific country can change over time.
